# Untargeted Diversity-Oriented Synthesis for the Discovery of New Antitumor Agents: An Integrated Approach of Inverse Virtual Screening, Bioinformatics, and Omics for Target Deconvolution

**DOI:** 10.1021/acs.jmedchem.5c01344

**Published:** 2025-07-24

**Authors:** Tania Ciaglia, Valeria Napolitano, Maria Rosaria Miranda, Danila La Gioia, Simona Musella, Aniello Schiano Moriello, Fabrizio Merciai, Veronica Di Sarno, Simona De Vita, Ester Colarusso, Gerardina Smaldone, Francesca Di Matteo, Eduardo Maria Sommella, Poulami Kumar, Marco Allarà, Alessia Ligresti, Isabel M. Gomez-Monterrey, Giuseppe Bifulco, Gianluigi Lauro, Pietro Campiglia, Carmine Ostacolo, Vincenzo Vestuto, Alessia Bertamino

**Affiliations:** † Department of Pharmacy, 19028 University of Salerno, Via G. Paolo II 132, 84084 Fisciano, Salerno, Italy; ‡ Institute of Biomolecular Chemistry, Consiglio Nazionale delle Ricerche, Comprensorio Olivetti, Via Campi Flegrei, 34, 80078 Pozzuoli, Naples, Italy; § Department of Pharmacy, University Federico II of Naples, Via D. Montesano 49, 80131 Naples, Italy; ∥ PhD Program in Drug Discovery and Development, University of Salerno, Via G. Paolo II 132, 84084 Fisciano, Salerno, Italy

## Abstract

Molecular diversity is one of the most pursued objectives in drug discovery, and diversity-oriented synthesis (DOS) perfectly responds to the achievement of this goal. In this paper, we describe a DOS approach applied to the antitumor field with the aim of identifying new anticancer structures and their associated targets. To accomplish this ambitious project, after an initial stage of phenotypic evaluation, we set up an integrated platform of inverse virtual screening (IVS), bioinformatics, and omics to predict the biological targets of the most promising compounds **31** and **63**. Several proteins emerged from this study, and the most interesting ones were assessed by biophysical and in cellulo experiments, leading to the validation of six targets involved in calcium regulation, endoplasmic reticulum stress, and apoptosis. This work allowed us to identify two hit compounds with an interesting antitumor mechanism, but principally, to validate our platform as a fruitful tool for untargeted DOS campaigns.

## Graphical abstract

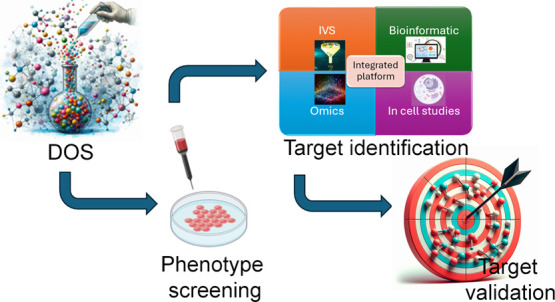

## Introduction

The drug discovery process has been a very fruitful practice for several decades, allowing the identification of an arsenal of agents capable of acting in the principal diseases and representing the pillar of the current pharmacological treatments.^1–3^ However, most drug discovery campaigns are addressed toward a specific target (target-oriented synthesis, TOS),^4–7^ which needs specific chemical requirements for its engagement, thus limiting the molecular diversity among its modulators. This is why conventional medicinal chemistry could be seen as a shrinking of the countless possibilities offered by the chemical space in the identification of biologically active molecules.

Again, the availability of crystal structures of macromolecules considered as pharmacological targets can bias the computer-aided design of new therapeutics, being based on specific binding pockets interacting with well-defined chemical functions. This circumscribes the design of new drugs to already discovered interaction sites, reflecting itself in a further restriction of the myriads of new chemical combinations resulting in a new compound.

The need to explore more broadly the chemical space to obtain high complexity and high diversity in chemical libraries is perfectly satisfied by the concept of diversity-oriented synthesis (DOS).^8,9^

The aim of DOS is principally focused on the achievement of high molecular diversity covering a wide region of chemical space, starting from similar reactants. This can be reached by considering appendage diversity, stereochemical diversity, or skeletal diversity.^8^ The first element of diversity is shared with combinatorial chemistry, which paved the way for the birth of the DOS technique.^10^ It is based on molecular scaffolds that have different points of decoration and can generate a collection of reaction intermediates that can themselves be further derivatized with different appendages, originating a huge library of different members.^11–13^

Stereochemical diversity is considered as important as molecular diversity, because of the tridimensional orientation of the pharmacophoric groups, which must be correctly directed toward their sites of interaction depending on molecular configuration. This is why many DOS approaches, to arrange of all the possible compound’s configurations, use stereoselective reactants to obtain enantio- or diastereoisomeric pure products.^14–17^

In the end, skeletal diversity is the most pursued approach, because, among all, it is responsible for the higher degree of diversity. It could be based on two different strategies, which are substrate-based or reagent-based skeletal diversity. The substrate-based approach uses different starting materials which are converted into different skeletons by the same reagents.^18–20^ The second one consists of using different reagents to transform a substrate having several dissimilar points of reactivity in different compounds characterized by wide skeleton variety.^21–23^

A restricted application of the DOS approach is directed toward privileged scaffolds which have been first proposed by Evans in 1988 ^24^ as chemical cores that, properly derivatized, can provide ligands for several receptors, and this concept is still very actual.^25–27^

A very noteworthy aspect of the DOS approach is that the high degree of chemical diversity achieved through this technique offers the possibility of discovering ligands for previously undruggable targets.^28–30^

It is widely recognized that DOS strategies have pleiotropic possibilities in the medicinal chemistry field, and this is also explained by the rising number of papers that appeared around this topic. However, the main drawback of this powerful approach is represented by the downstream processes, consisting of identifying the molecular target(s) involved in the compounds’ mechanism of action. The latter could be considered as “looking for a needle in a haystack” and usually the available tools for pursuing this objective are insufficient. This is why many DOS campaigns are specifically addressed toward an established target, and they are called “biological-oriented synthesis” (BIOS).^31–34^ In this kind of approach, a huge number of compounds are synthesized following the DOS principles and screened against one specific target. This procedure is definitely much easier, but it takes the risk of losing some off-target mechanisms, preventing the complete pharmacological characterization of the obtained compounds.

In this paper, we describe an approach of untargeted diversity-oriented synthesis (UnDOS) addressed to the identification of new antitumor agents, using the reagent-based approach in order to obtain different privileged scaffolds. We chose a *L*-amino acid as a common reagent because of its easy accessibility, high chemical versatility, and safe profile. We selected *L*-Leu and *L*-Phe as building blocks to build all the scaffolds, which were further derivatized with aliphatic or aromatic side chains to preliminarily explore the suitability of these kinds of generic appendages. Using different reaction conditions and substrates, we obtained 10 diverse privileged scaffolds (Figure 1),^35–45^ which were subsequently further derivatized. All the synthesized molecules were then phenotypically screened to evaluate their cytotoxicity against a broad panel of tumor cell lines using a nontumorigenic cell line as a safety control. To establish the target(s) involved in the anticancer mechanism of the most cytotoxic molecules, we set up an integrated platform consisting of omic studies, inverse virtual screening (IVS), in silico-based calculations, and bioinformatic simulations. All the inputs coming from these investigations were strictly supported by in cellulo biological experiments and finally the most promising targets, suggested by the integration of all the obtained results, were validated by specific biochemical tests.

**Figure 1:**
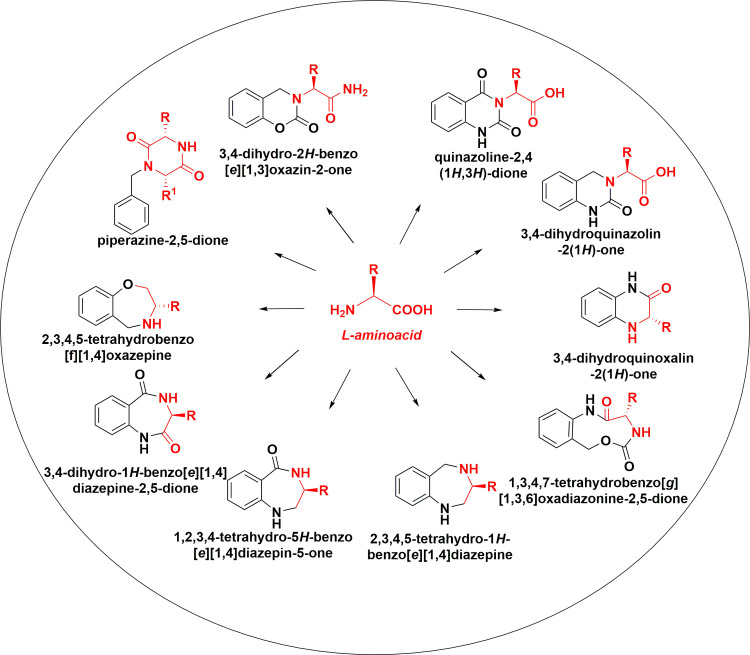
Graphical representation of the present reagent-based UnDOS strategy.

Together with the discovery of new antitumor compounds, the main objective of this article is focused on the design, validation, and implementation of our integrated screening platform as a helpful tool for a complete biological characterization of new chemical entities obtained through the DOS approach.

## Results and Discussion

### Chemistry

Compounds **7**, **8**, **13**, and **14** were obtained according to Scheme 1.

**Scheme 1:**
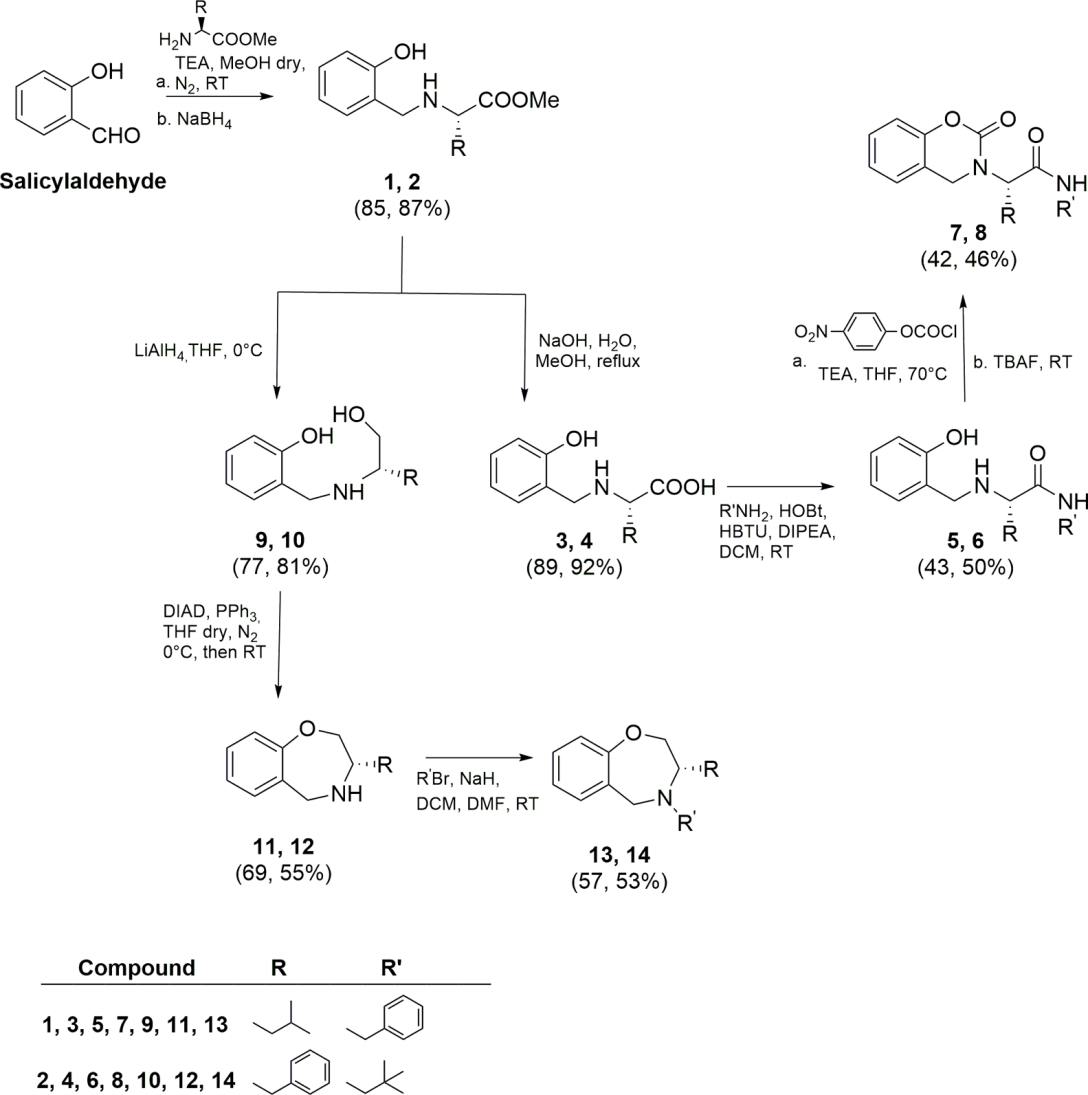
Synthesis of Compounds **7**, **8**, **13**, and **14**

Salicylaldehyde was subjected to reductive amination with commercially available *L*-leucine methyl ester or *L*-phenylalanine methyl ester furnishing, respectively, intermediates **1** and **2** in 85 and 87% yield. The following hydrolysis of methyl ester groups by an aqueous solution of NaOH gave, in almost quantitative yield, compounds **3** and **4**. These compounds were subjected to a coupling reaction with benzylamine or *tert*-butylamine, using HOBt and HBTU as coupling agents and DIPEA as the base, affording intermediates **5** (43% of yield) and **6** (50% of yield), respectively. The final cyclization employing 4-nitrophenyl chloroformate as the carbonyl source and tetrabutylammonium fluoride (TBAF) gave the desired products **7** and **8** in 42 and 46% of yield.

Intermediates **1** and **2** were further reduced to the alcohol intermediates **9** and **10** (77 and 81% of yield, respectively) employing LiAlH_4_ in THF. Then, an intramolecular cyclization under Mitsunobu reaction conditions using diisopropyl azodicarboxylate (DIAD) and triphenylphosphine in dry THF afforded the derivatives **11** and **12**. The alkylation with the proper alkyl bromide in basic medium yielded the final tetrahydrobenzo­[*f*]­[1,4]­oxazepinic compounds **13** and **14** in 57 and 53% yield, respectively.

The quinazoline-2,4-(1*H*,3*H*)-diones **22** and **23** were synthesized according to Scheme 2. 2-Aminobenzoic acid was protected with a Boc group, by reaction with Boc anhydride, and coupled with the proper amino acid under the same conditions described above, giving intermediates **16** and **17** in good yield (80 and 90%, respectively). The following hydrolysis in alkaline medium and esterification with isopropyl alcohol or benzyl alcohol led to intermediates **18** (65% yield) and **19** (58% yield), respectively. These compounds were then subjected to Boc removal using DCM/TFA (3:1, v:v) and triisopropylsilane (TIS) as the scavenger, yielding **20** and **21** almost quantitatively. The final products **22** and **23** were obtained by a microwave-assisted reaction using di-*tert*-butyl dicarbonate and 4-(dimethylamino)­pyridine (DMAP) in acetonitrile (ACN) with an isolated yield of 42 and 44%, respectively.

**Scheme 2:**
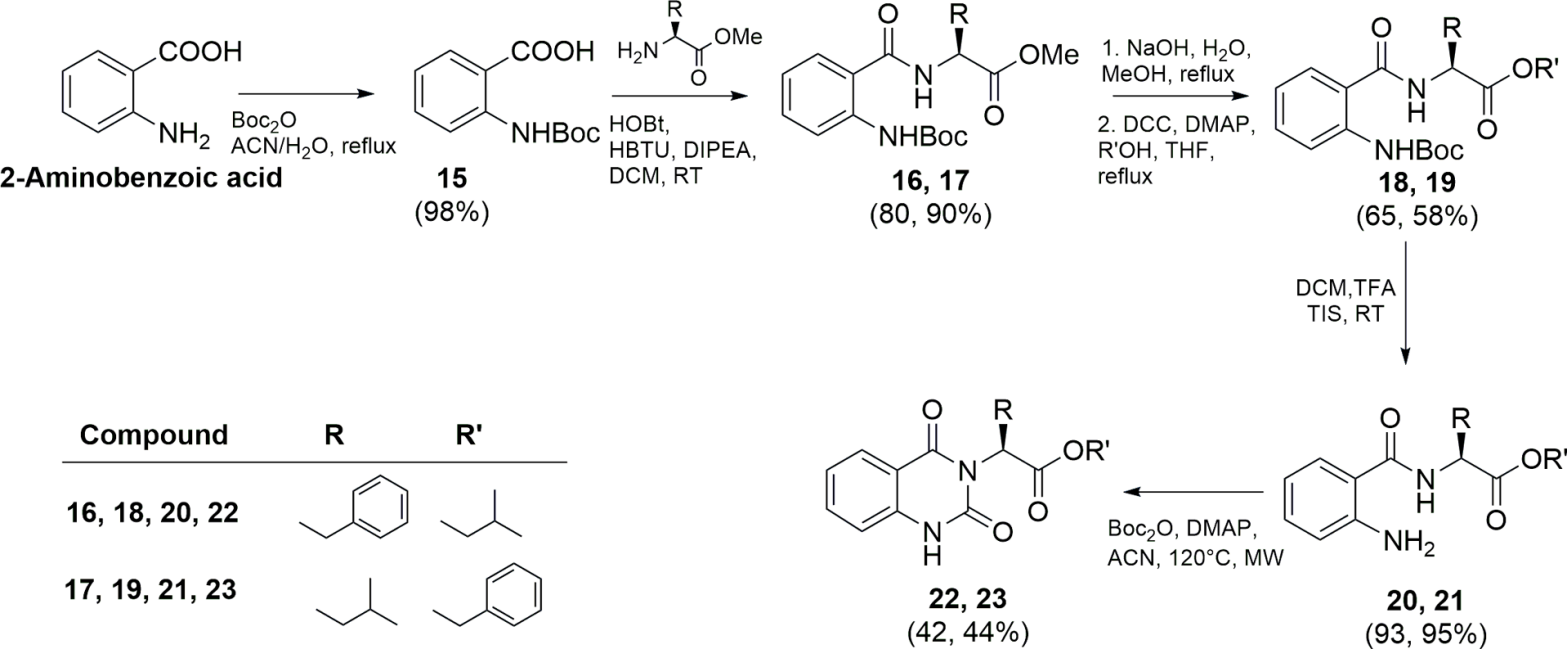
Synthesis of Compounds **22** and **23**

The synthesis of final derivatives **28–31**, **33**, **34**, **37,** and **38** is shown in Scheme 3.

**Scheme 3:**
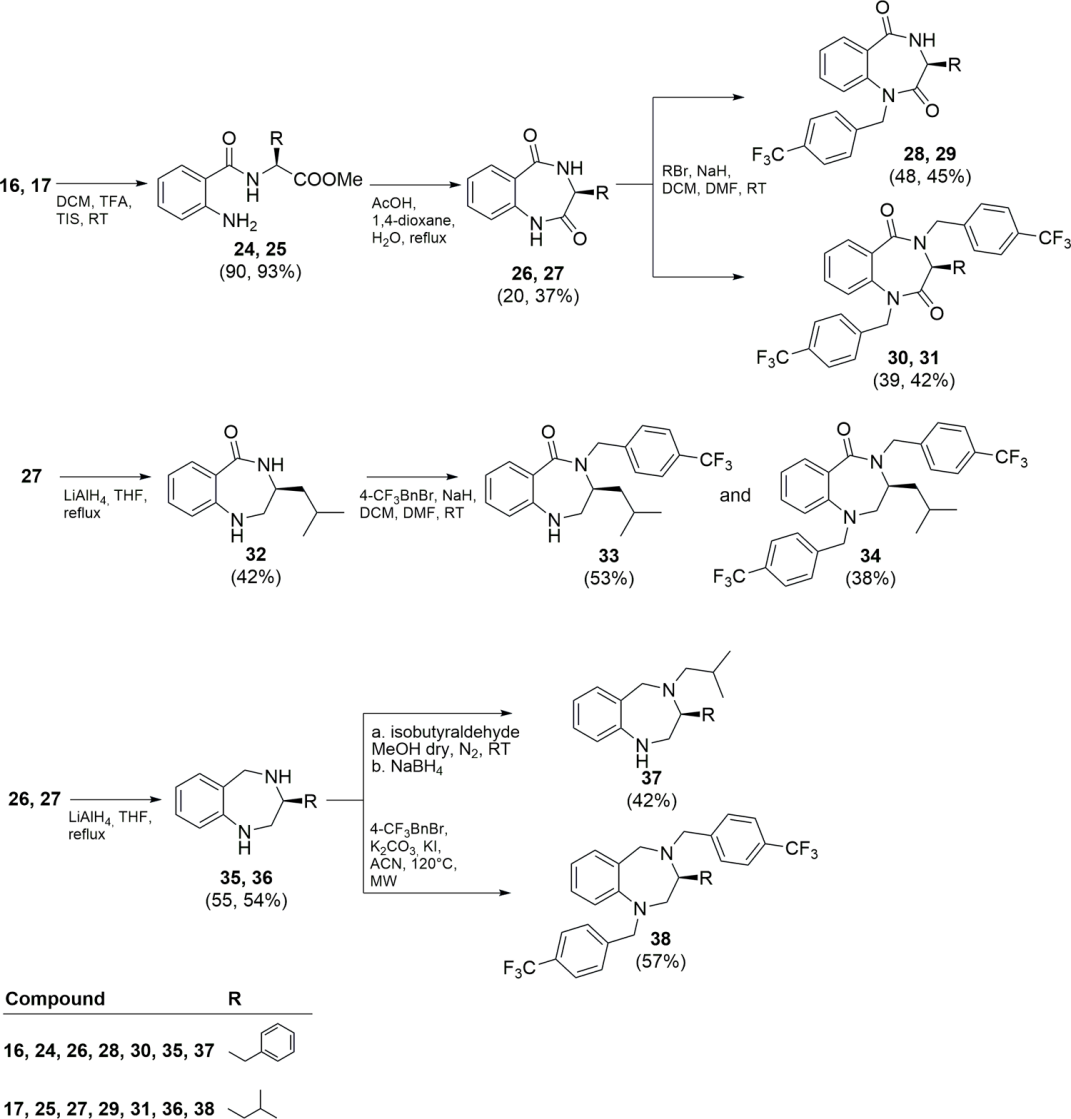
Synthesis of Compounds **28–31**, **33**, **34**, **37**, and **38**

Intermediates **16** and **17** were deprotected on the amino group, as described above, and subjected to an intramolecular acid-catalyzed cyclization affording intermediates **26** and **27** (20 and 37%, respectively). The following *N*-alkylation with the proper alkyl bromide and NaH as the base in DCM/DMF (4:1, v:v) furnished the final compounds **28–31** (39–48% yield).

The reduction reaction employing LiAlH_4_ in THF of intermediates **26** and **27** gave monoreduced **32** (42% yield) and bireduced derivatives **35** and **36** (55 and 54% yield, respectively). 4-(Trifluoromethyl)­benzyl bromide was then reacted with **32** in basic medium affording **33** and **34** in 53 and 38% yield, respectively. The reductive amination of intermediate **35** with isobutyraldehyde afforded the final compound **37** in 42% of yield, while intermediate **36** was subjected to a microwave-assisted *N*-alkylation reaction with 4-(trifluoromethyl)­benzyl bromide, K_2_CO_3_, and KI giving the final product **38** (57% yield).

Synthesis of the diketopiperazine derivative **41** is depicted in Scheme 4.

**Scheme 4:**
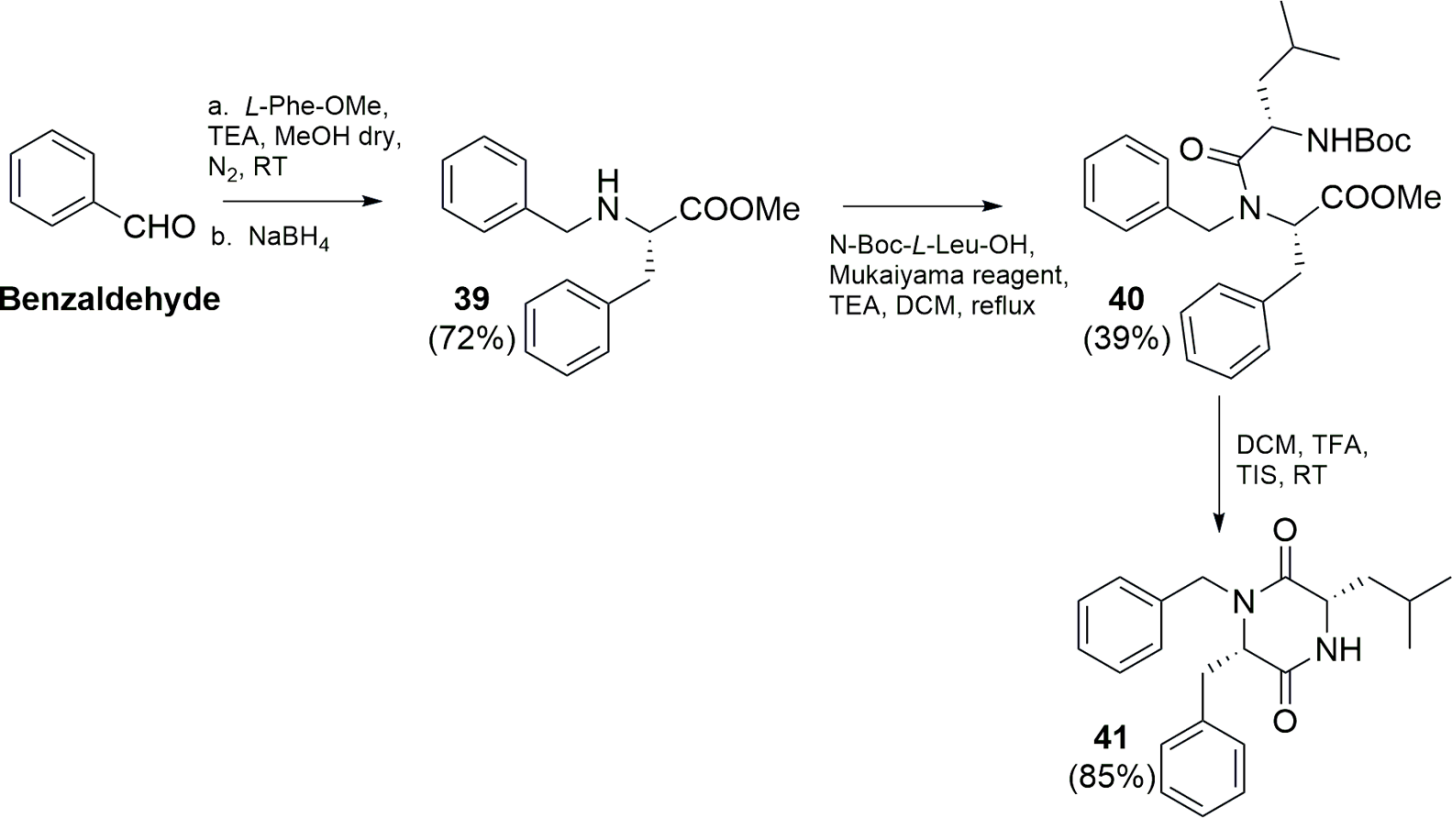
Synthesis of Compound **41**

Commercially available benzaldehyde was reacted with *L*-phenylalanine methyl ester under reductive amination conditions, giving **39** in 72% of yield. A subsequent coupling reaction with *N*-Boc-*L*-Leu-OH using 2-chloro-1-methylpyridinium iodide (Mukaiyama reagent) and TEA in DCM afforded intermediate **40** (39% yield). Compound **40** after treatment with a mixture of DCM/TFA (3:1, v:v) spontaneously cyclized to the final derivative **41**, almost quantitatively.

Scheme 5 depicts the synthesis of dihydroquinoxalinic derivatives **48** and **49** using 2-iodoaniline as the starting material. It was coupled with *N*-Boc-protected *L*-leucine or *L*-phenylalanine and deprotected at the amino group as described above, affording intermediates **44** and **45**. A palladium/copper cocatalyzed C–N coupling reaction under microwave irradiation in a mixture of DMF/water (10:1, v:v), in the presence of K_2_CO_3_ and TEA, yielded intermediates **46** and **47** (45 and 32% yield, respectively) that were reacted with 1-bromo-2-methylpropane or benzyl bromide affording final derivatives **48** (38% yield) and **49** (44% yield), respectively.

**Scheme 5:**
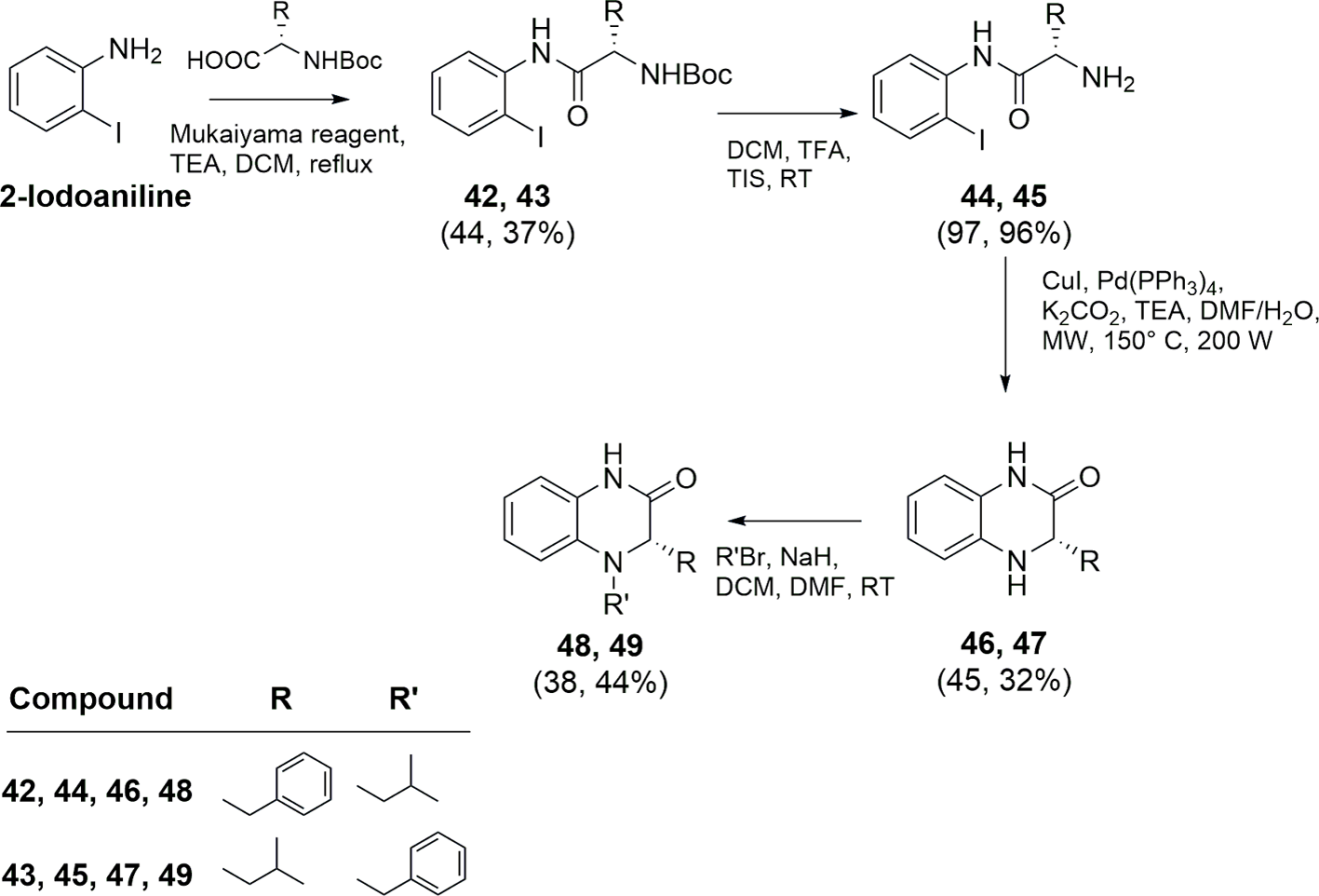
Synthesis of Compounds **48** and **49**

The synthetic route followed for the synthesis of final derivatives **52**, **63,** and **64** is reported in Scheme 6.

**Scheme 6:**
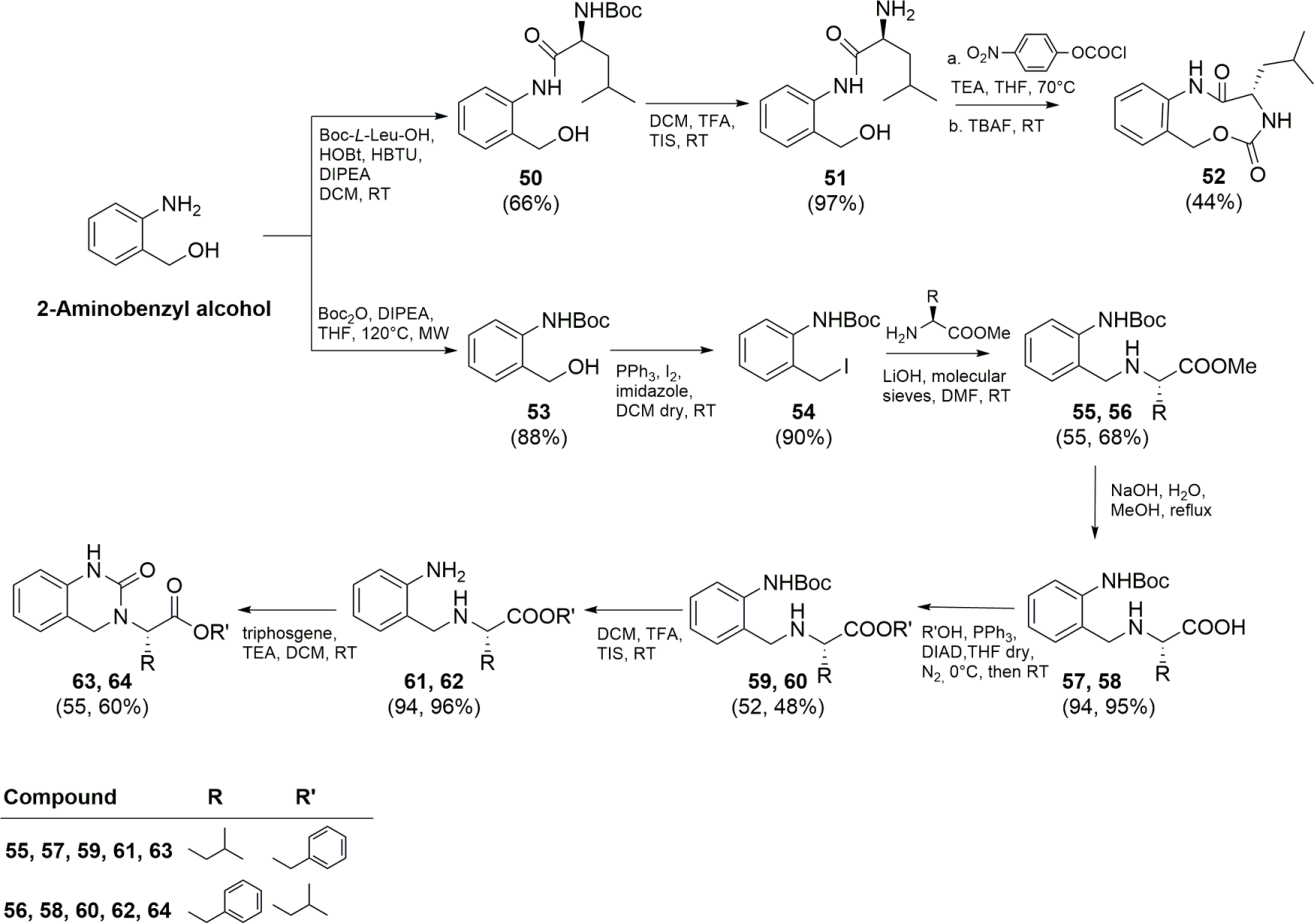
Synthesis of Compounds **52**, **63,** and **64**

The 2-aminobenzyl alcohol was coupled with Boc-*L*-Leu-OH giving intermediate **50** in 66% of yield. This intermediate was deprotected and subjected to intramolecular cyclization, following the same reaction conditions mentioned above, leading to compound **52** in 44% of isolated yield.

For the synthesis of compounds **63** and **64**, 2-aminobenzyl alcohol was first converted into the *N*-Boc-protected derivative **53**, in almost quantitative yield, and then into the corresponding benzyl iodide **54** using iodine, triphenylphosphine, and imidazole in dry DCM (90% yield). Intermediate **54** was reacted with *L*-leucine methyl ester or *L*-phenylalanine methyl ester using LiOH as the base in DMF affording intermediates **55** and **56** (55 and 68% isolated yield) that were subjected to hydrolysis giving intermediates **57** and **58** almost quantitatively. Subsequent esterification with benzyl or isopropyl alcohol and Boc removal in acid medium afforded intermediates **61** and **62**. Final urea-based compounds **63** and **64** were obtained by an intramolecular cyclization using triphosgene and TEA as the base in DCM (55 and 60% of yield).

### In Cellulo Cytotoxicity Evaluation

Initially, we phenotypically screened the synthesized compounds over a panel of tumor cell lines composed of HEPG2, A375, SHSY5Y, MCF7, and A549 derived from liver tumor, melanoma, neuroblastoma, breast cancer, and lung adenocarcinoma, respectively. We also considered the HaCaT cell line, coming from keratinocytes, as a healthy control line. As reference, cytotoxic chemotherapeutic agent 5-fluorouracil (5-FU) has been used. The results obtained performing the MTT (3-(4,5-dimethylthiazolyl-2)-2,5-diphenyltetrazolium bromide) assay are summarized in Table 1.

**Table 1:**
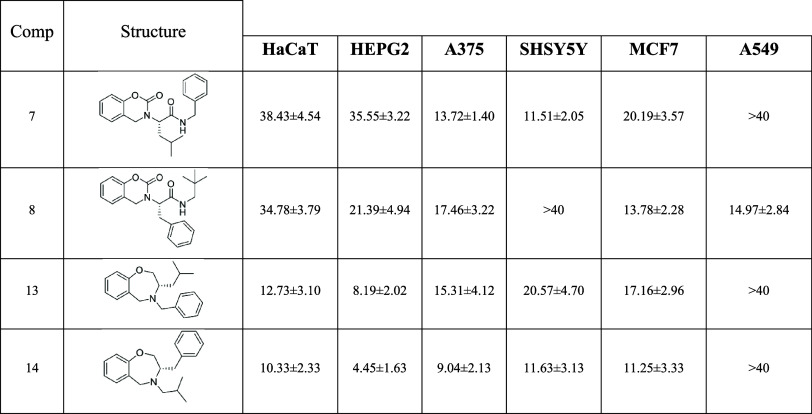

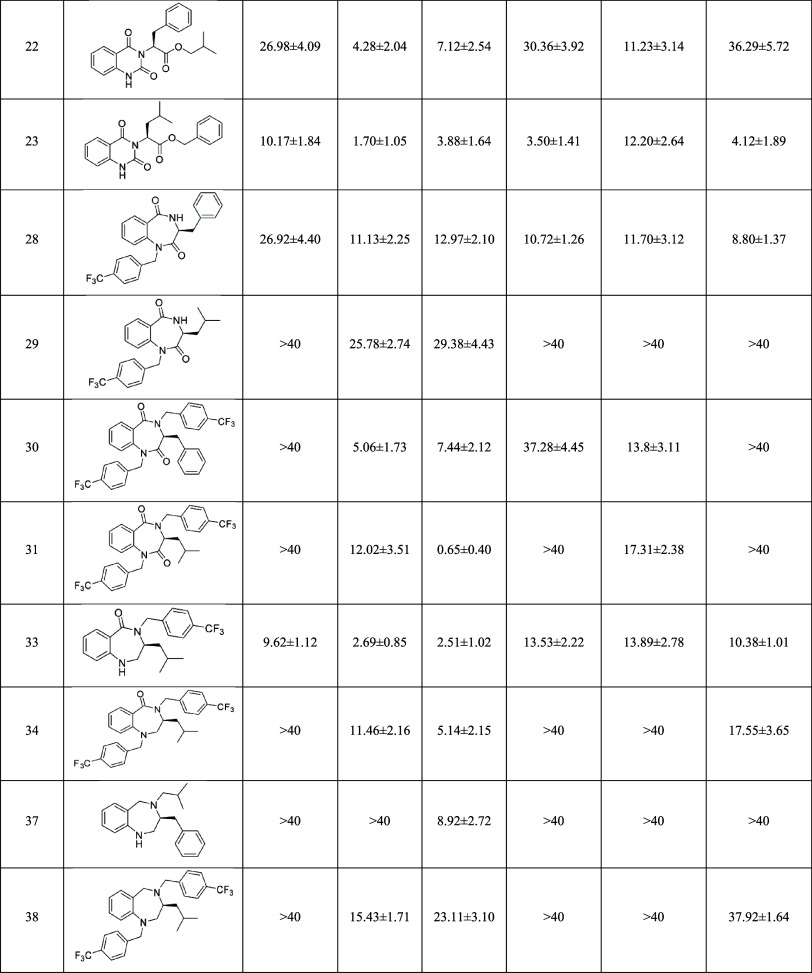

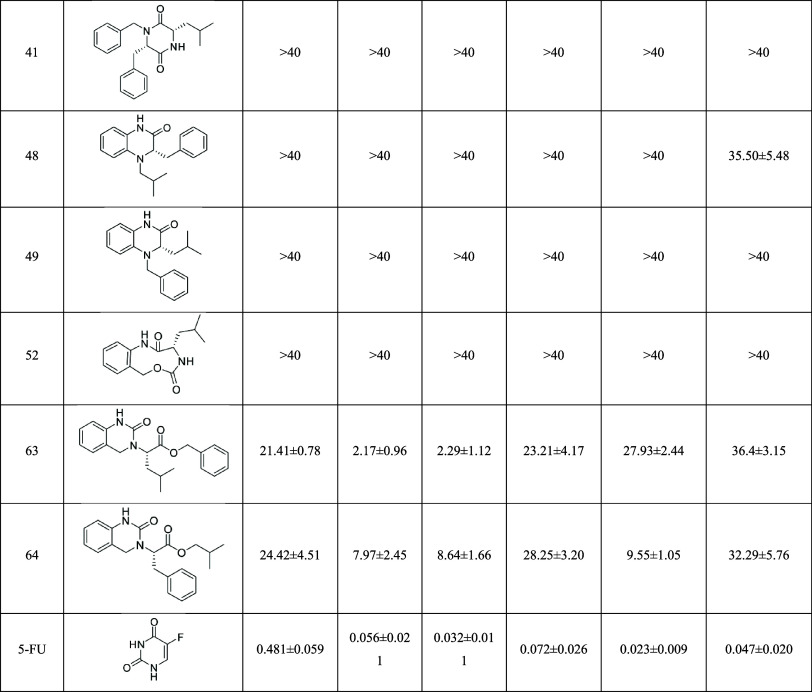
In Vitro Cytotoxicity of Synthesized Compounds^a^

a Data are reported as EC_50_ ± SD (μM).

This first evaluation allowed us to discard noncytotoxic compounds as well as those lacking selectivity over the cancer lines with respect to the healthy one.

Collectively, we found that most of the compounds were characterized by a remarkable cytotoxic activity over the cancer cell lines used, except piperazine-2,5-dione (**41**), 3,4-dihydroquinoxalin-2­(1*H*)-one derivatives (**48**, **49**), and 1,3,4,7-tetrahydrobenzo­[*g*]­[1,3,6]­oxadiazonine-2,5-dione (**52**) showing no activity (Table 1). Moreover, some compounds, namely 3,4-dihydro-2*H*-benzo­[*e*]­[1,3]­oxazin-2-one (**7**, **8**) and 2,3,4,5-tetrahydrobenzo­[*f*]­[1,4]­oxazepine (**13**, **14**), exhibited cytotoxicity effect also over the HaCaT cell lines. Also, other derivatives, characterized by different scaffolds, namely **23**, **28**, **33**, and **64**, although with high cytotoxicity over tumor cell lines, were discarded for further study, because of their lack of selectivity.

Based on these results, we selected two derivatives, compounds **31** and **63**, for further biological characterization, as they exhibited micromolar cytotoxicity and preferential activity against cancer cells. To quantify this selectivity, we calculated the selectivity index (SI), defined as the ratio of EC_50_ in healthy HaCaT cells to that in A375 melanoma cells. Compound **31** showed the highest SI value among all tested derivatives (SI = 61.54), indicating strong selectivity toward tumor cells. Compound **63**, despite being more cytotoxic on HaCaT cells than compound **31**, also displayed a favorable SI (SI = 9.87), supporting its preferential activity against A375 cells. These two compounds presented the highest SI values in the series, justifying their selection for further investigations. Accordingly, A375 cells, on which compounds **31** and **63** showed the greatest activity and selectivity, were chosen for additional biological studies.

### IVS for Target Identification

The IVS computational protocol was used to predict the interacting molecular targets of compounds **31** and **63** underlying their biological activity. This technique is advantageous in target identification because it allows quickly testing a small set of compounds through molecular docking calculations on a wide range of targets involved, for example, in a pathological event (e.g., cancer, inflammation, etc.) and singles out the most promising interacting macromolecules.^46,47^

The compounds were docked against a panel of cancer-related proteins containing nearly seven thousand structures (see Table S5, SI). Afterward, the obtained energies were normalized using decoy molecules, and to identify the most promising targets for the two compounds, we employed a funnel approach to refine the list of potential targets systematically (see Figure 2 and Experimental Section for full details).

**Figure 2:**
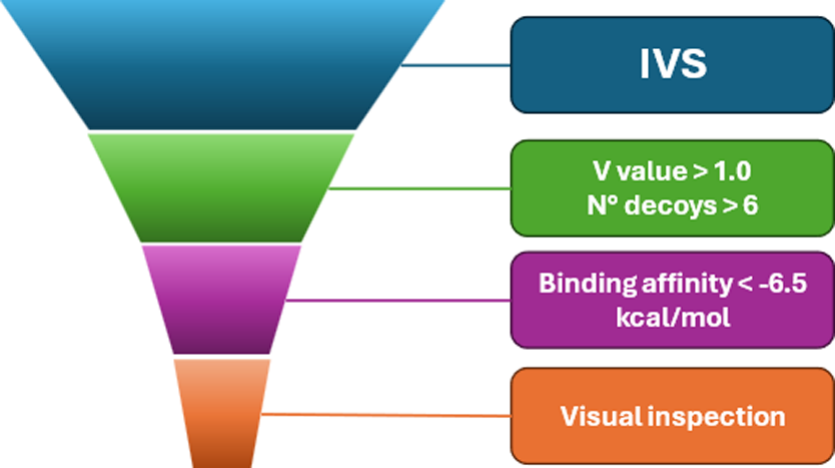
Funnel filtering approach used to narrow down the IVS results.

Finally, the two lists reported in Tables 2 and 3 were obtained. The comparison of the proteins identified for the two test compounds highlighted three items as common putative partners for both **31** and **63**, which are discussed below. Further details regarding the discussion of the remaining predicted ligand/protein complexes are reported in the Supporting Information (see SI Section 3: Interacting targets of compounds **31** and **63** predicted by IVS and the related Figures S62 and S63).

**Table 2:** IVS Results Highlighting the Most Promising Interacting Targets of Compound **31**

name	UniProt	Best V	Best docking score (kcal/mol)
receptor tyrosine-protein kinase erbB-2	ERBB2_HUMAN (P04626)	2.26	–9.19
17-beta-hydroxysteroid dehydrogenase 13	DHB13_HUMAN (Q7Z5P4)	2.09	–11.83
E3 ubiquitin-protein ligase Mdm2	MDM2_HUMAN (Q00987)	1.81	–7.99
Poly [ADP-ribose] polymerase tankyrase-1	TNKS1_HUMAN (O95271)	1.78	–8.13
bromodomain-containing protein 4	BRD4_HUMAN (O60885)	1.63	–8.04
retinoic acid receptor RXR-alpha	RXRA_HUMAN (P19793)	1.54	–8.60
transient receptor potential cation channel subfamily M member 8	TRPM8_HUMAN (Q7Z2W7)	1.21	–7.22
Poly [ADP-ribose] polymerase 1	PARP1_HUMAN (P09874)	1.21	–6.73
serine/threonine-protein kinase B-raf	BRAF_HUMAN (P15056)	1.17	–7.94
cannabinoid receptor 2	CNR2_HUMAN (P34972)	1.15	–11.47

**Table 3:** IVS Results Highlighting the Most Promising Interacting Targets of Compound **63**

name	UniProt	Best V	Best docking score(kcal/mol)
aurora kinase A	AURKA_HUMAN (O14965)	2.30	–7.25
protein-tyrosine kinase 6	PTK6_HUMAN (Q13882)	2.20	–7.95
transient receptor potential cation channel subfamily M member 2	TRPM2_HUMAN (O94759)	2.01	–7.39
Poly [ADP-ribose] polymerase tankyrase-1	TNKS1_HUMAN (O95271)	1.60	–7.05
bromodomain-containing protein 4	BRD4_HUMAN (O60885)	1.57	–7.56
GTPase KRas	RASK_HUMAN (P01116)	1.32	–6.61
transient receptor potential cation channel subfamily M member 8	TRPM8_HUMAN (Q7Z2W7)	1.05	–6.28
cannabinoid receptor 2	CNR2_HUMAN (P34972)	1.00	–9.90

### Binding Mode of Compounds **31** and **63** against the In-Common Identified Most Promising Targets

#### Cannabinoid Receptor 2

The cannabinoid receptor 2 (CB2R) is a G protein-coupled receptor (GPCR) expressed mainly in peripheral tissues and, especially in neuroinflammatory conditions, in the central nervous system. Agonists of the cannabinoid system were found to reduce cell proliferation and migration and promote autophagy, apoptosis, and cell cycle arrest.^48^ Key residues, for example, Phe87, Phe94, His95, Ile110, Val113, and Phe183 seem crucial for the interaction.^49^ The reference structure used in this case is 6PT0 ^50^ containing the agonist WIN 55, 212-2.

From the analysis of the poses, it emerged that both **31** and **63** proficiently bind CB2R, making interactions with key amino acids. In detail, as shown in Figure 3A,B, compound **31** interacts with Phe183 and Trp194 through direct π–π stacking, while hydrophobic interactions are established with Phe87 and Val113. Moreover, an extra π–π stacking interaction with His95 contributes to the positioning of **31** within the binding site. On the other hand, compound **63** exhibits a double π–π stacking with Trp194, representing the only interaction with an amino acid considered essential for binding, suggesting a less effective binding than **31** (Figure 3C,D).

**Figure 3:**
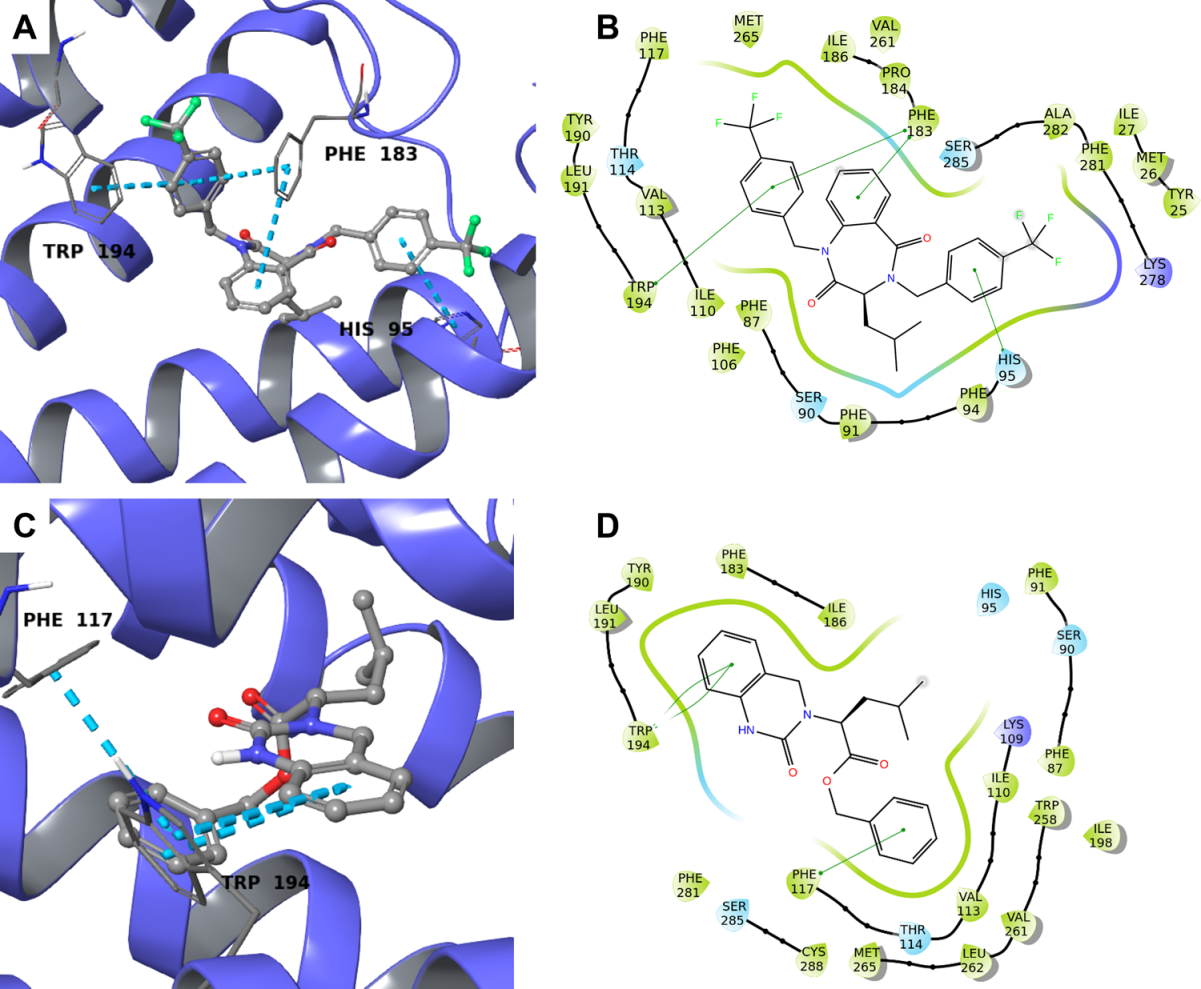
Predicted binding between 31 and 63 and CB2R (violet ribbon, PDB: 6PT0). (A,C) 3D view. Cyan dotted lines indicate π–π stacking and the interacting amino acids are labeled. (B,D) 2D view. Green lines represent π–π stacking, polar amino acids are in cyan, hydrophobic ones are in green, and positively charged residues are in blue.

#### Transient Receptor Potential Melastatin 8

The protein structure used for IVS calculations contains the human transient receptor potential Melastatin 8 (TRPM8) protein (PDB code:8BDC).^51^ According to Palchevskyi et al., Arg842 and Lys856 are required for TRPM8 channel voltage regulation, while Arg842, Tyr745, and Tyr1005 interact with menthol, a known TRPM8 ligand.

Despite having a higher docking score compared to **63**, the interactions shown in Figure 4A,B, involving only a single amino acid (Arg1008) suggest that the binding of **31** with TRPM8 is not optimal.

**Figure 4:**
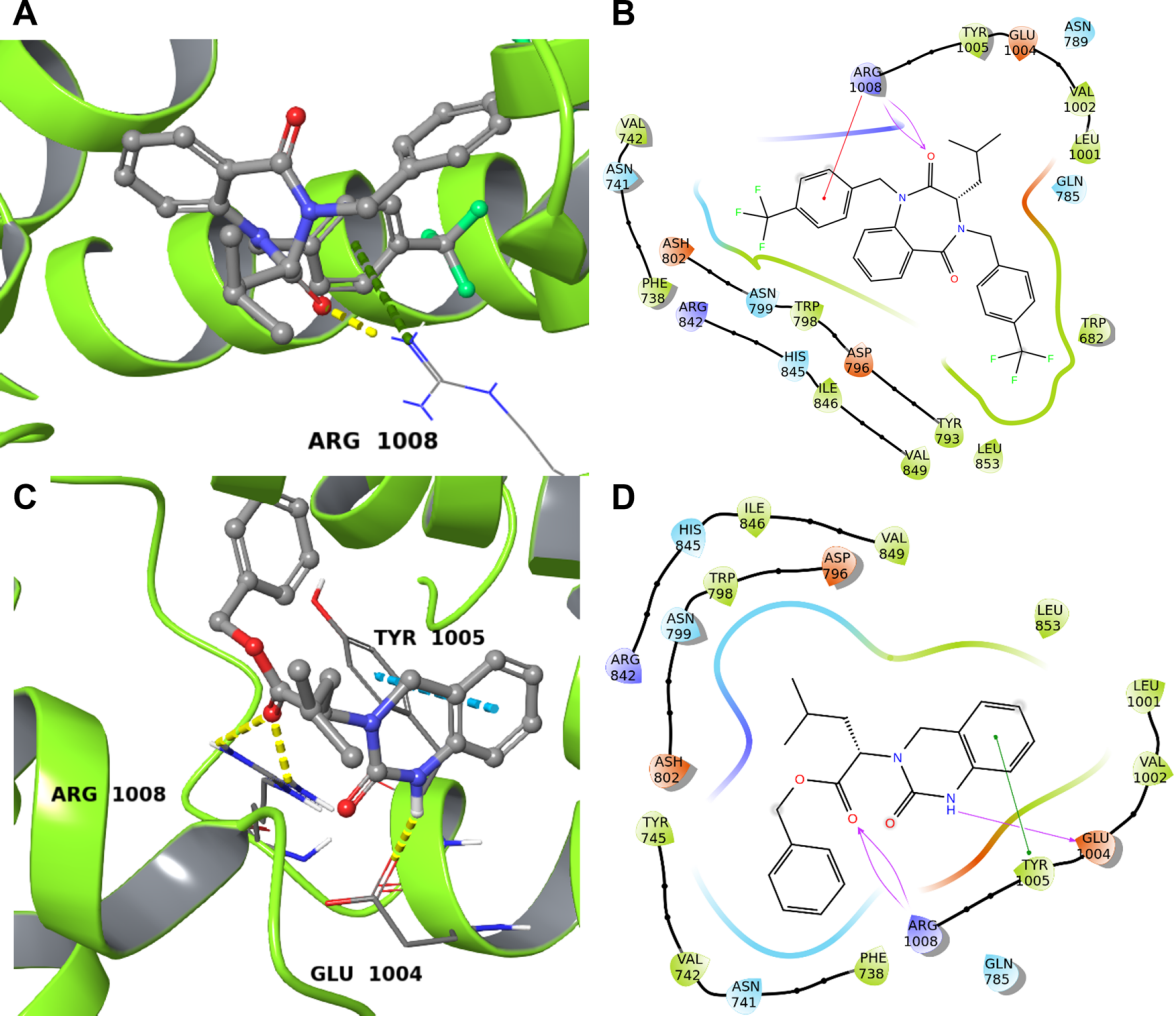
Predicted binding between **31** and **63** and TRPM8 (lime ribbon, PDB ID: 8BDC). (A,C) 3D view. Cyan dotted lines indicate π–π stacking and yellow dotted lines represent hydrogen bonds. The interacting amino acids are labeled. (B,D) 2D view. Green lines represent π–π stacking, pink lines are hydrogen bonds, and π–cation interactions are reported with red lines. Polar amino acids are in cyan, hydrophobic ones are in green, and positively charged residues are in blue.

In contrast, **63** exhibits a more favorable interaction pattern. It forms a π–π stacking interaction with Tyr1005, an amino acid considered essential for binding. Additionally, it maintains hydrogen bonds with Arg1008 and forms an additional hydrogen bond with Glu1004 (Figure 4C,D).

#### Bromodomain-Containing Protein 4

Bromodomain-containing protein 4 (BRD4) is a member of the Bromodomain protein family, responsible for the recognition of methylated lysine residues on histones.^52^ Dysregulations in BRD4 activity were found in several cancers, making it extensively studied.^53^

As reported in the literature,^54^ Asn140 represents a critical amino acid in blocking the activity of BRD4. Moreover, Trp81 belongs to a WPF shelf that is included in the active site of this reader. Both molecules, therefore, make proficient interactions with this target, showing a similar predicted binding energy, and indicating a promising activity against this target (Figure 5).

**Figure 5:**
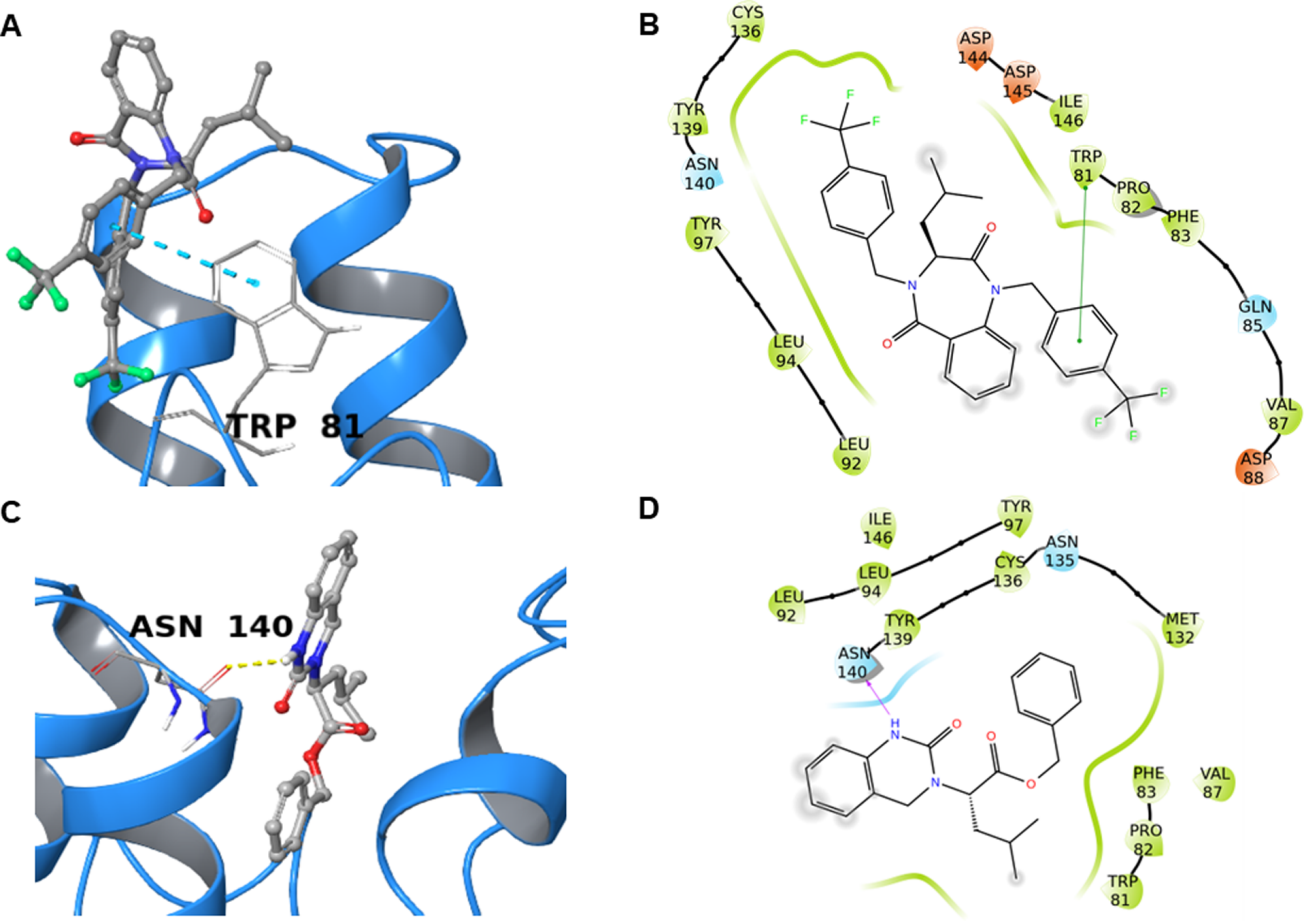
Predicted binding between **31** and **63** and BRD4 (blue ribbon, PDB ID: 6DJC and 7MRA, respectively). (A,C) 3D view. Cyan dotted lines indicate π–π stacking, and yellow dotted lines represent hydrogen bonds. The interacting amino acids are labeled. (B,D) 2D view. Green lines represent π–π stacking, and pink lines are hydrogen bonds. Polar amino acids are in cyan, hydrophobic ones are in green, and negatively charged residues are in red.

### Bioinformatic Studies for Target Identification

Bioinformatic studies were performed using Swiss Target Prediction (STP)^55^ and Super Pred (SP)^56^ databases to shed further light on the molecular mechanism underlying compounds **31** and **63** biological activity. These tools use different scoring systems: STP provides probability scores (typically below 0.1), where values closer to 1 indicate a higher likelihood of interaction based on chemical similarity to known ligands. SP, instead, reports the likelihood as a percentage, reflecting the confidence level of the prediction grounded in structural similarity to bioactive compounds in its database. Both methods were employed to complement each other’s limitations, improving the overall reliability of target predictions.

To prioritize these candidates, mRNA expression data from the Protein Atlas transcriptomic database (Human Protein Atlas proteinatlas.org) were analyzed. The A375 melanoma cell line was specifically selected for this analysis because it showed the most favorable EC_50_ values in our cytotoxicity assays, making it the most relevant model to investigate the potential molecular targets of compounds. Specifically, targets with high mRNA expression levels in A375 melanoma cells and no detectable expression in HaCaT keratinocytes were selected, as these were considered potentially involved in tumor-specific cytotoxicity. The filtered results are presented in Tables 4 and 5. The full lists of predicted targets prior to transcriptomic filtering, along with corresponding mRNA expression data and classification based on the applied criteria, are provided in Supplementary Tables S1 and S2 for compound **31**, and in Supplementary Tables S3 and S4 for compound **63**.

**Table 4:** Identified Targets for **31** Cross Bioinformatics Tools (SP, STP) and Transcriptomics Data (Protein Atlas)^a^

target name (SP)	probability (SP)	nTPM A375 (Protein Atlas)	nTPM HaCaT (Protein Atlas)
cannabinoid receptor 1	95%	3.4	0
transient receptor potential cation channel subfamily A member 1	87%	1.2	0
aminopeptidase N	81%	58.1	0

**target name (STP)**	**probability (STP)**	**nTPM A375 (Protein Atlas)**	**nTPM HaCaT (Protein Atlas)**
cannabinoid receptor 1	0.095623787	3.4	0
transient receptor potential cation channel subfamily M member 8	0.095623787	5.4	0
vasopressin V2 receptor	0.095623787	1.4	0

a nTPM, normalized transcripts per million.

**Table 5:** Identified Targets for **63** Cross Bioinformatics Tools (SP, STP) and Transcriptomics Data (Protein Atlas)^a^

target name (SP)	probability (SP)	nTPM A375 (Protein Atlas)	nTPM HaCaT (Protein Atlas)
Cathepsin K	91.2%	15.8	0
G-protein coupled receptor 55	84.8%	1.9	0

**target name (STP)**	**probability (STP)**	**nTPM A375 (Protein Atlas)**	**nTPM HaCaT (Protein Atlas)**
adenosine A1 receptor (by homology)	0.109339753	10.5	0
fibroblast growth factor receptor 1	0.109339753	10.2	0
vascular endothelial growth factor receptor 2	0.109339753	2.4	0
platelet-derived growth factor receptor beta	0.109339753	1.9	0
G-protein coupled receptor 55	0.109339753	1.9	0
vascular endothelial growth factor receptor 1	0.109339753	11.3	0
thromboxane-A synthase	0.109339753	3.0	0
phosphodiesterase 10A	0.109339753	1.1	0

a nTPM, normalized transcripts per million.

### Multiomics Characterization of Compounds **31** and **63** in A375 Cells

An integrated multiomics approach was employed to unravel the effects of **31** and **63** in A375 cells. In this regard, label-free quantification (LFQ) proteomic profiling of cells treated with compound **31** evidenced a partial separation in the principal component analysis (PCA) score plot (Figure 6A), with differential analyses resulting in 76 statistically significant proteins (Figure 6B). String network (Figure 6C) and enrichment (Figure 6D) evidenced that the pathway with the highest strength in the network was related to the heterocycle metabolic process; on the other hand, the most involved cellular compartment resulted in endoplasmic reticulum protein-containing complex, while Reactome pointed out proteins involved in post-translational modifications. Additionally, gene enrichment (Figure 6E) pointed out heme biosynthesis and porphyrin metabolism as well transcription of E2F targets. On the contrary, compound **63** showed only 25 differentially regulated proteins, and a high degree of overlap with nontreated cells, probably due to higher biological variability, hence the proteome modulation was not significant for **63**, despite a potential involvement of proteins involved in acetylation processes (Figure S64A-D).

**Figure 6:**
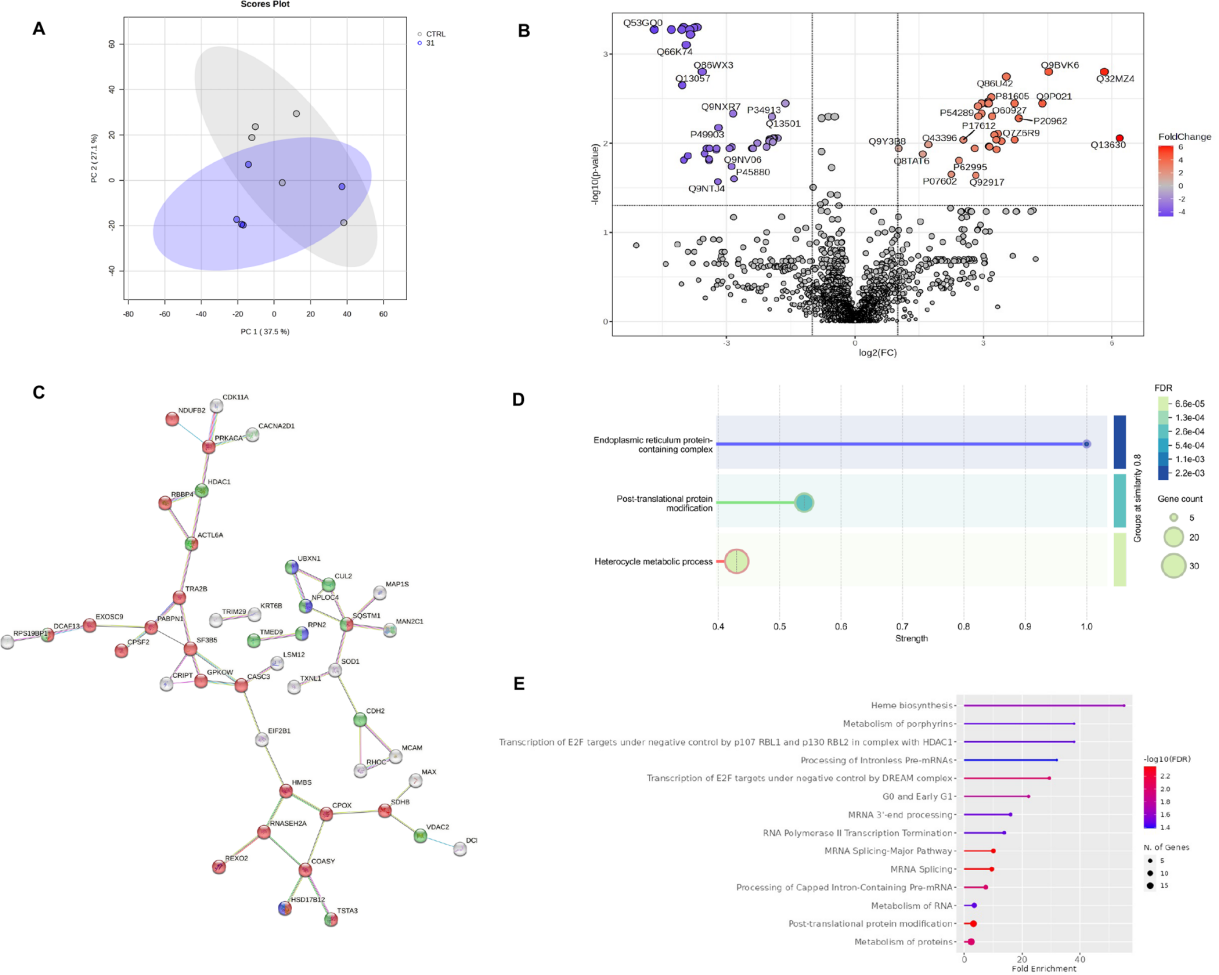
LFQ Proteomics results: Principal component analysis (A) showing clustering of **31** vs ctrl and (B) volcano plot of differentially expressed proteins, (C) STRING network and enrichment (D) of statistically significant proteins, and (E) gene ontology.

At the lipidomics level, interestingly, both compounds **31** and **63** showed a net lipid remodeling (Figure 7A,B), resulting in 49 and 74 differentially modulated lipids, respectively (Figure 7C,D), in particular, diacylglycerols (DGs), that resulted significantly reduced in treated-cells, as well as cholesterol esters (CEs), whereas lysophosphatidylcholines (LPCs) and lysophosphatidylethanolamines (LPEs) were notably increased in the treatments (Figure 7E,F). In this regard, CEs are important lipid droplet (LD) constituents; they serve as the storage form of cholesterol and are produced by the enzyme acyl-CoA acyltransferase (ACAT). Free cholesterol plays a crucial role in maintaining membrane fluidity. Abnormal buildup of CEs in LDs is a key focus in the reprogramming of tumor metabolism. Elevated CE levels are a distinct metabolic hallmark observed in several cancers and unfavorable patient outcomes.^57^ Additionally, the reduction of DGs could be related to an acyl-CoA diacylglycerol acyltransferase (DGAT) inhibition, which is an essential enzyme to convert DGs to triacylglycerols (TGs), major constituents of LDs. In this context, targeting LDs in melanoma cells has been recently associated with metabolic vulnerability.^58^ Finally, an overall increase of ceramides levels (Cer), potentially linked to a pro-apoptotic effect,^59^ has been observed with both compounds.

**Figure 7:**
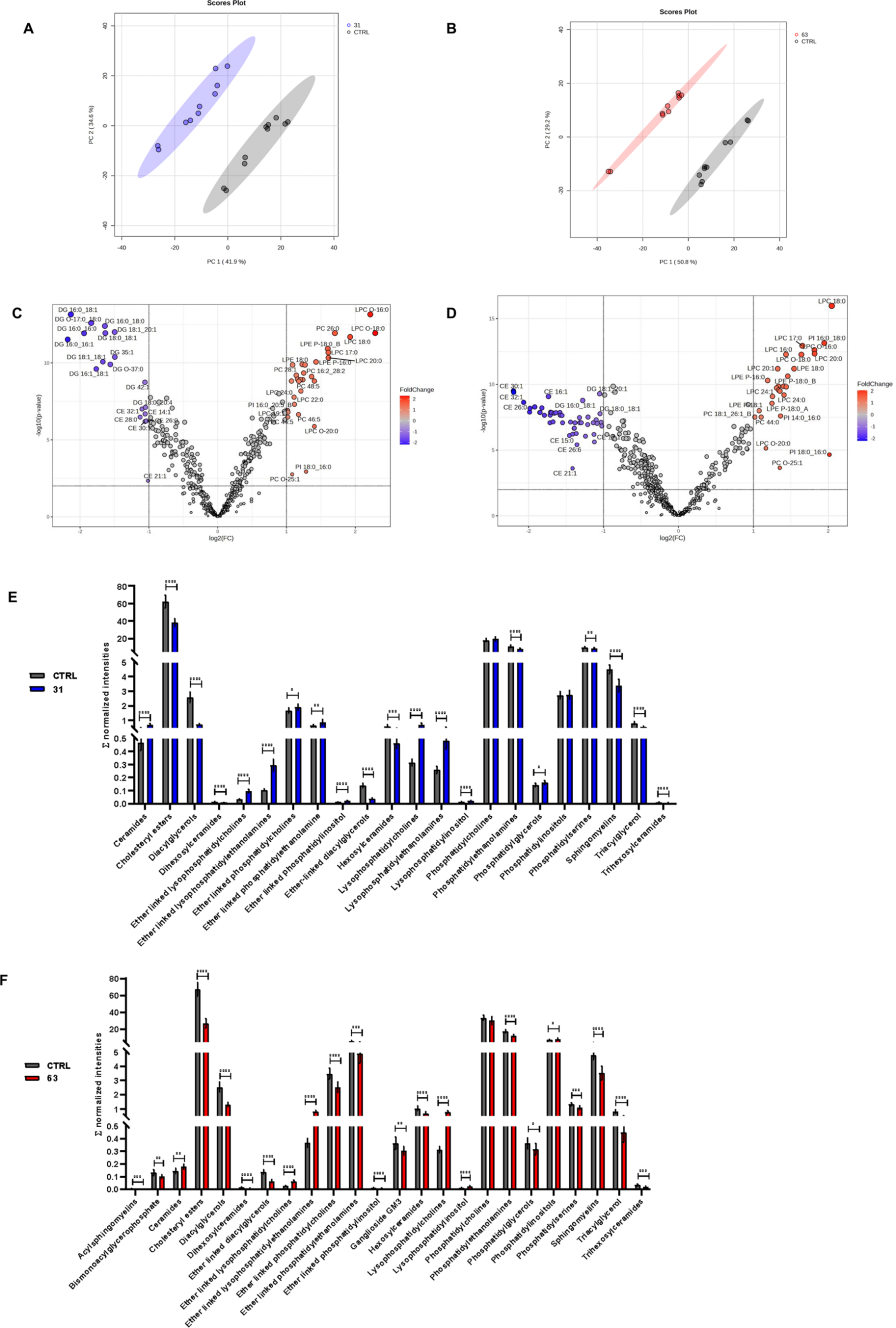
Untargeted lipidomics results: Principal component analysis (A,B) showing clustering of **31** and **63** vs CTRL and (C,D) volcano plot of differentially modulated lipids, and (E,F) subclass analysis with their average abundance between treatments and ctrl.

Untargeted metabolomics showed that both **31** and **63** influence cellular metabolism as can be appreciated by the score plots of PCA in Figure 8A,B, with 47 and 23 differentially abundant metabolites with *p*-value < 0.05 and FC > ±2 (Figure 8C,D). The HMDB codes of statistically significant metabolites were used to build the enrichment based on SMPDB (The Small Molecule Pathway Database, Figure 8E,F). Compound **31** showed as main enriched terms methionine (*p* = 9.88 × 10^–04^) and purine metabolism (*p* = 0.00208), while compound **63** showed purine metabolism (*p* = 3.39 × 10^–04^), glutamate (*p* = 7.89 × 10^–04^), and glutathione metabolism (*p* = 0.0155). Interestingly, cells treated with compound **31** show an increase in the metabolite 5′-S-methyl-5′-thioadenosine (MTA). MTA is a bioproduct of the S-adenosylmethionine (SAM), and it is utilized by methylthioadenosine phosphorylase that synthesizes methionine in the methionine salvage pathway. In this regard accumulation of MTA in cellulo has been associated with proapoptotic and generally chemopreventive effects,^60^ and also with the inhibition of proliferation in melanoma cells.^61,62^

**Figure 8:**
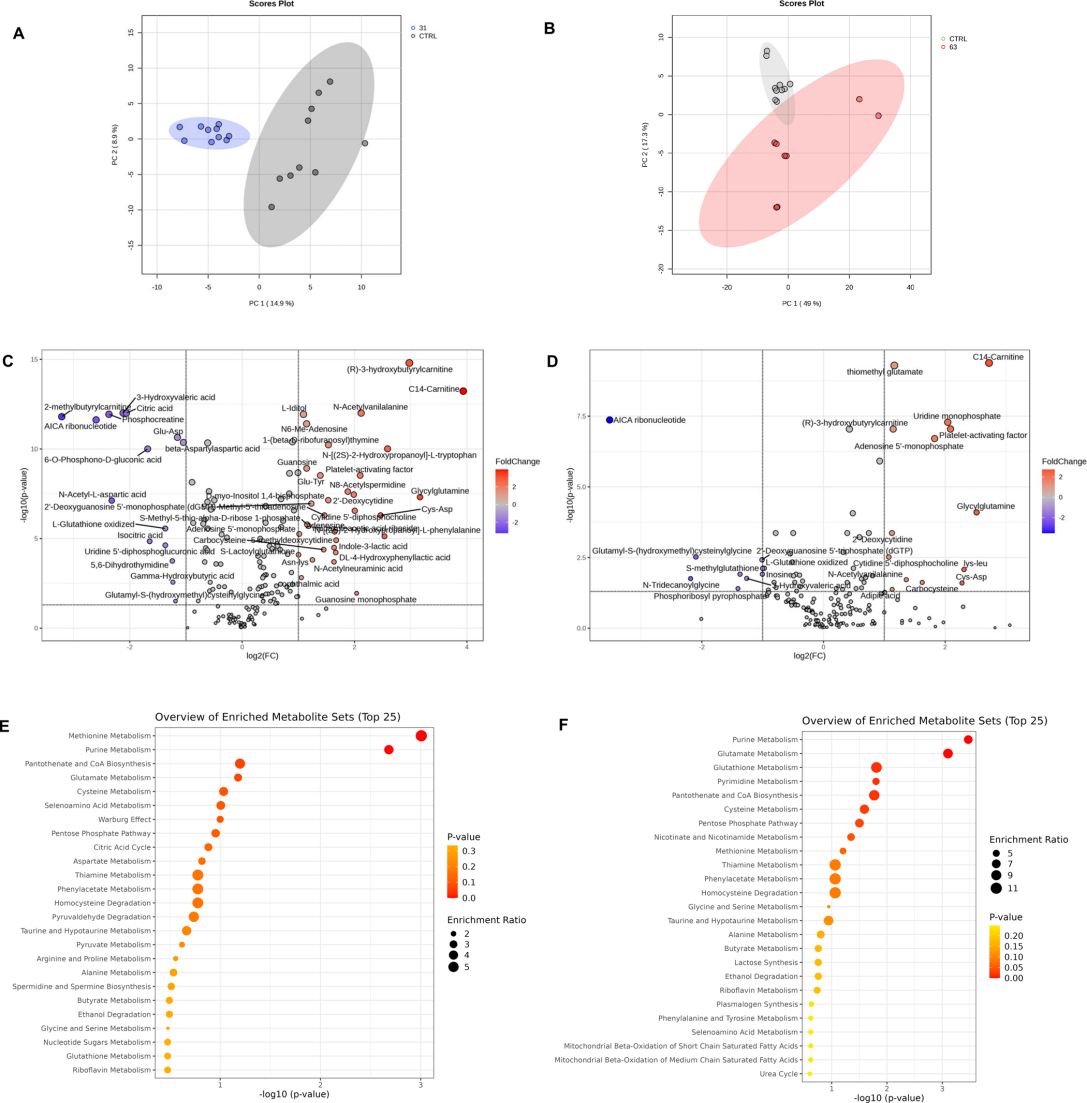
Untargeted metabolomics results: Principal component analysis (A,B) showing clustering of **31** and **63** vs ctrl and (C,D) volcano plot of differentially modulated metabolites, (E,F) SMPDB enrichments of **31** and **63**.

### Cellular Assays

Considering that omics showed an activation of oxidative stress pathways by compounds **31** and **63** on A375 cells, the intracellular production of reactive oxygen species (ROS) following treatment was assessed, using 6-carboxy-2′,7′-dichlorodihydrofluorescein diacetate (DCFH-DA). Cells were treated with compounds **31** and **63** (10, 5, and 2.5 μM) for 2–8 h, and ROS levels were subsequently measured. As a result, a significant concentration-dependent increase in ROS levels was observed, at 2 h (10 and 5 μM, *p* < 0.001 vs Ctrl; 2.5 μM, *p* < 0.01 vs Ctrl) and 4 h (*p* < 0.01 vs Ctrl) for compound **31**, while for compound **63**, a greater increase was observed at 4 h (10 and 5 μM, *p* < 0.001 vs Ctrl; 2.5 μM, *p* < 0.05 vs Ctrl) and 8 h (10 μM, *p* < 0.001 vs Ctrl; 5 and 2.5 μM, *p* < 0.01 vs Ctrl), which may indicate a delayed and more moderate effect than compound **31** (Figure 9A,B).

**Figure 9:**
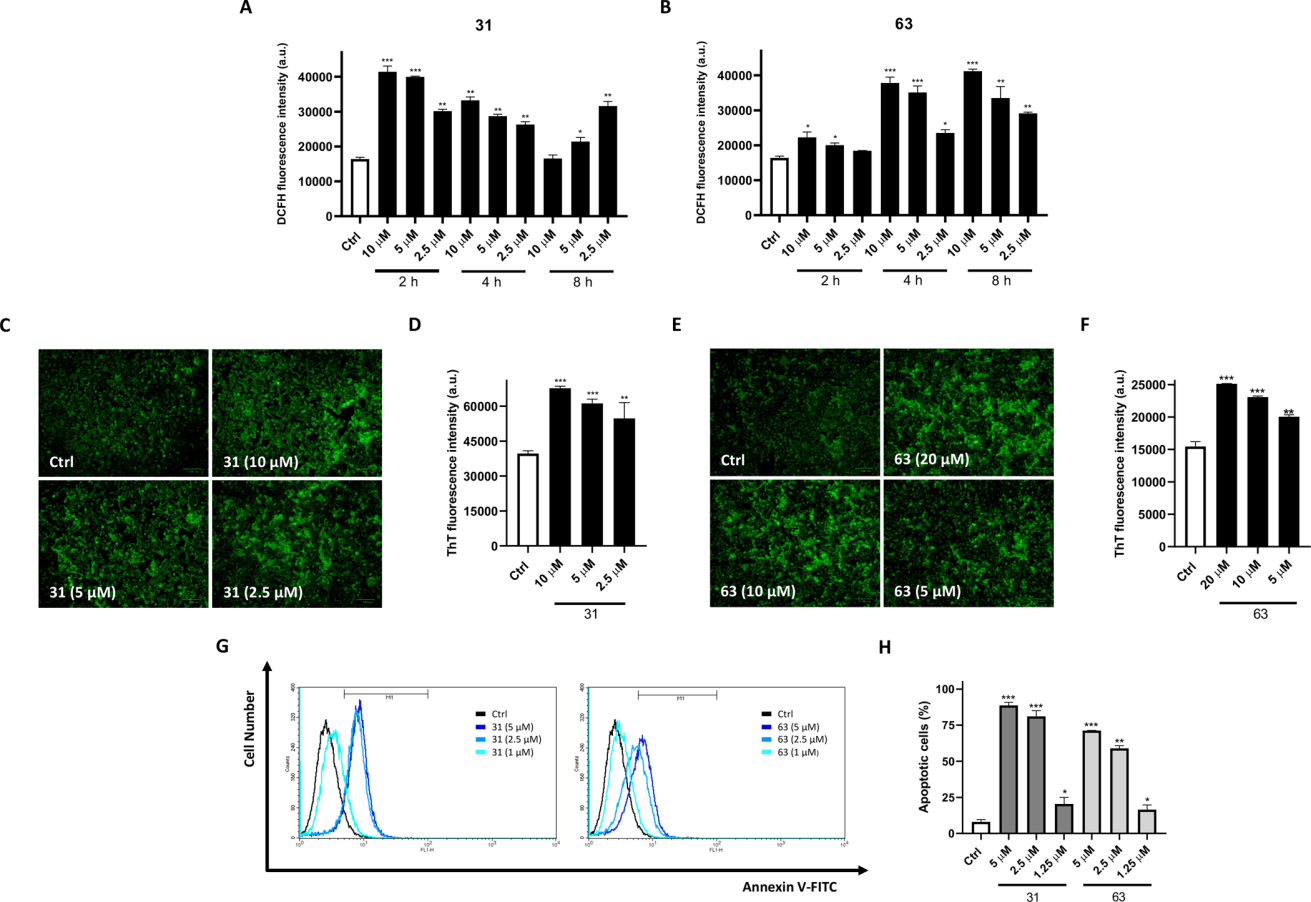
The administration of **31** and **63** induces oxidative stress, protein misfolding and apoptosis. ROS assay revealed oxidative stress triggered by compounds **31** and **63**. Quantitative analysis of ROS production was reported, after the exposure of compounds (A) **31** and (B) **63** (10, 5, 2.5 μM) for 2 to 8 h. (C,E) ThT test reported protein misfolding levels after exposure to compounds **31** (10, 5,2.5 μM) and **63** (20, 10, 5 μM) for 24 h. The fluorescence was observed by fluorescence microscope (ZOE Fluorescent Cell Imager microscope, Biorad. Magnification, 20×. Scale bar: 100 μm). (D,F) Quantitative analysis was reported. (G) Representative flow cytometry graphs with Annexin V-FITC staining for pro-apoptotic activity on A375 cells treated for 72 h with compounds **31** and **63** at 5, 2.5, and 1.25 μM. (H) Relevant quantitative analyses are shown. Results are expressed as the percentage of apoptotic cells. Data are expressed as mean ± SD of three different experiments performed in triplicate. *p < 0.05 vs Ctrl; **p < 0.01 vs Ctrl; ***p < 0.001 vs Ctrl.

To further corroborate the cellular effects of compounds revealed in proteomics analysis, a thioflavin T (ThT) assay was assessed to measure the misfolded protein levels in A375 cells. Cells were treated with compound **31** (10, 5, and 2.5 μM) and **63** (20, 10, and 5 μM) for 24h. ThT assay revealed a significant signal of fluorescence intensity that decreased in a concentration-dependent manner. Compound **31** caused the maximum accumulation of misfolded proteins even at 10 μM (*p* < 0.001 vs Ctrl), while compound **63** showed the greatest increase in fluorescence at 20 μM (*p* < 0.001 vs Ctrl) (Figure 9C–F). These effects are in line with cell viability data, as compound **31** is more cytotoxic than compound **63** (Table 1). The ThT assay results indicate that compound administration may impair protein homeostasis, potentially leading to increased misfolding and aggregation.

Given the cytotoxic role of the compounds, an annexin V-FITC staining was performed to evaluate their effects on cell apoptosis. Flow cytometric analysis revealed that administration of compounds **31** and **63** significantly promoted apoptosis at 5 μM compared with untreated controls (**31**: 88.68 ± 2.10%, *p* < 0.001 vs Ctrl; **63**: 71.25 ± 0.11%, *p* < 0.001 vs Ctrl). This trend was less pronounced but still evident at 2.5 μM (**31**: 81.10 ± 3.89%, *p* < 0.001 vs Ctrl; **63**: 58.90 ± 1.89%, *p* < 0.01 vs Ctrl), whereas, at 1.25 μM (**31**: 20.40 ± 4.56%, *p* < 0.05 vs Ctrl; **63**: 16.52 ± 3.24%, *p* < 0.05 vs Ctrl), minimal but significant effects were observed. These results demonstrate that the administration of compounds **31** and **63** induces apoptosis in A375 cells in a concentration-dependent manner (Figure 9G,H).

### In Vitro Validation

The introduced integrated platform suggested several targets suitable for the interaction with both compounds **31** and **63**. This strategy allowed us to tremendously narrow the field of investigation for targets deconvolution and gave us the possibility to validate each protein proposed by IVS and bioinformatic tools. Thus, we performed in vitro experiments, testing the selected compounds over the proposed targets. Among all the targets predicted by IVS and bioinformatic, we considered only those whose mRNA levels were significantly higher in A375 cells than in HaCaT.

The first six entries of Table 6 report the successfully validated targets through biophysical and in cellulo experiments, while in the remaining rows are the other unengaged proteins.

**Table 6:** Target Validation for Compounds **31** and **63**

target	method	31 (EC_50_ or IC_50_ μM)	63 (EC_50_ or IC_50_ μM)
isocitrate dehydrogenase	IDH1 enzyme assay (R132H; BPS Bioscience, USA)	14.92 ± 2.12	not soluble
protein-tyrosine kinase 6	BRK kinase assay (BPS Bioscience, USA)	18.96 ± 3.04	29.17 ± 4.53
transient receptor potential A1	Ca^2+^-imaging assay exploiting calcium-sensitive fluorescent probe Fluo-4 AM	0.20 ± 0.09	2.54 ± 1.20
transient receptor potential M8	Ca^2+^-imaging assay exploiting calcium-sensitive fluorescent probe Fluo-4 AM	>30	0.36 ± 0.08
cannabinoid receptor 2	Binding assays evaluating the displacement of the high-affinity radioligand [^3^H]-CP-55,940 (Perkinelmer, Italy)	9.70 ± 0.67	6.91 ± 0.45
bromodomain-containing protein 4	BRD4 (BD1) Inhibitor Screening Assay Kit	1.19 ± 0.29	0.11 ± 0.09
Cathepsin K	Cathepsin K Inhibitor Screening Assay Kit (BPS Bioscience, USA)	>30	>30
peroxisome proliferator-activated receptor gamma	PPARγ-LBD Ligand Screening Assay Kit (Cayman, USA)	>30	>30
Poly [ADP-ribose] polymerase 1	PARP1 Colorimetric Assay Kit (BPS Bioscience, USA)	>30	>30
Poly [ADP-ribose] polymerase tankyrase-1	TNKS1 (PARP5A) Colorimetric Assay Kit (BPS Bioscience, USA)	>30	>30
serine/threonine-protein kinase B-raf	B-Raf kinase assay (V600E; BPS Bioscience, USA)	≥30	≥30
cannabinoid receptor 1	Binding assays evaluating the displacement of the high-affinity radioligand [^3^H]-CP-55,940 (Perkinelmer, Italy)	>30	>30
Vascular Endothelial Growth Factor Receptor-1 Flt-1 (VEGFR1) (FLT1)	Kinase Enzymatic Radiometric [10 uM ATP] KinaseProfiler LeadHunter Assay - FR	≥30	≥30
Fibroblast Growth Factor Receptor 1 (FGFR1)	Kinase Enzymatic Radiometric [10 uM ATP] KinaseProfiler LeadHunter Assay - FR	≥30	≥30
A1 Human Adenosine GPCR	Binding Antagonist Radioligand LeadHunter Assay - FR	≥30	≥30
A1 Human Adenosine GPCR	Binding Agonist Radioligand LeadHunter Assay - FR	≥30	≥30
Thromboxane Synthase	Enzymatic LeadHunter Assay–FR	≥30	≥30
Platelet-Derived Growth Factor Receptor-Beta (PDGFRB)	Kinase Enzymatic Radiometric [10 uM ATP] KinaseProfiler LeadHunter Assay - FR	≥30	≥30
Vascular Endothelial Growth Factor Receptor (VEGFR-2)/KDR	Kinase Enzymatic Radiometric [10 uM ATP] KinaseProfiler LeadHunter Assay - FR	≥30	≥30
Phosphodiesterase 10A2	Enzymatic LeadHunter Assay - FR	≥30	≥30
G-protein coupled receptor 55	Cell Based Antagonist Arrestin LeadHunter Assay - US	≥30	≥30
G-protein coupled receptor 55	Cell Based Agonist Arrestin LeadHunter Assay - US	≥30	≥30
V2 Human Vasopressin/Oxytocin GPCR	Binding Agonist Radioligand LeadHunter Assay - FR	≥30	≥30

These results highlight how compound **31** works as a potent agonist of Transient Receptor Potential Cation Channel Subfamily A Member 1 (TRPA1) with an EC_50_ of 0.20 ± 0.09 μM. It also inhibits BRD4 with low micromolar potency (IC_50_ = 1.19 ± 0.29 μM), while it shows moderate abilities in modulating isocitrate dehydrogenase (IDH), protein-tyrosine kinase 6 (BRK) and CB2R, with an IC_50_/EC_50_ between 9.7 ± 0.67 and 18.96 ± 3.04 μM. Regarding derivative **63**, we noticed a very remarkable inhibition of TRPM8 (IC_50_ = 0.36 ± 0.08 μM) and BRD4 (IC_50_ = 0.11 ± 0.09 μM), associated with a micromolar activation of CB2R (EC_50_ = 6.9 ± 0.45 μM), activation of TRPA1 (EC_50_ = 2.54 ± 1.20 μM), and a weak inhibition of BRK (IC_50_ = 29.17 ± 4.53 μM).

Concerning the interesting binding affinity over CB2R from compound **63**, we felt we should investigate it further to shed light on its functional activity, using the cAMP Hunter assay enzyme fragment complementation chemiluminescent detection kit. Interestingly, instead of reducing NKH-477-induced cAMP levels as a classical agonist would, **63** demonstrated a trend toward increasing cAMP levels, a behavior indicative of inverse agonism (Figure 10A). Moreover, in the presence of JWH133 (Figure 10B), a selective CB2R agonist, **63**, in a dose-dependent manner, displaced it and significantly elevated NKH-477-induced cAMP levels beyond the baseline. These results confirm the functional inverse agonist activity of **63** at CB2R.

**Figure 10:**
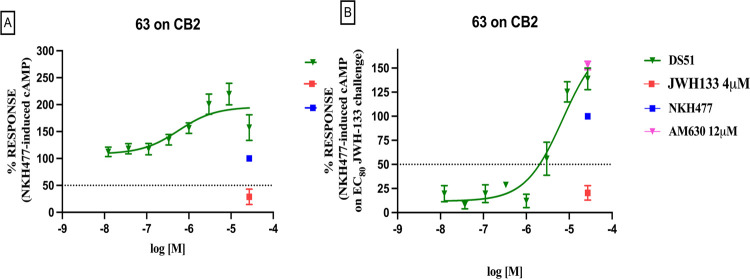
Concentration–response curves of compound **63** in the cAMP Hunter assay enzyme fragment complementation chemiluminescent detection kit. The curves show the effect of increasing concentrations of the compound on NKH-477-induced cAMP levels in stable CHO cells expressing the human CB2R. The effect of JWH-133 is reported as a reference compound. Data are reported as means ± standard error of three independent experiments conducted in triplicate and normalized considering the NKH-477 stimulus alone as 100% of the response as indicated in the experimental session. EC_50_ was determined by GraphPad Prism 9 as the concentration that provokes the response halfway between the baseline (bottom) and maximum response (top) of the fitted dose–response.

## Discussion

The selective cytotoxicity observed for compounds **31** and **63** against A375 melanoma cells underscores their potential in the development of selective anticancer agents. To elucidate the molecular mechanisms underlying this selective activity, we employed a comprehensive multidisciplinary approach integrating IVS, bioinformatic predictions, and biochemical as well as omics data profiling with functional in cellulo or in vitro validation.

The integration of these techniques with experimental validation has highlighted TRP channels, BRD4 and CB2R as the most involved targets, justifying the compounds **31** and **63** antiproliferative activity. However, despite the consistency of the overall target profile, some notable discrepancies emerged between computational predictions and experimental outcomes, warranting critical evaluation.

TRPM8, a transient receptor potential cation channel, plays a crucial role in calcium homeostasis and cellular signaling, processes often dysregulated in cancer and, particularly, in melanoma.^63–65^ TRPM8 was identified by both IVS (Tables 2, 3 and Figure 4A–D) and transcriptomic data (Table 4) as a top target for compound **31**, exhibiting elevated mRNA expression in melanoma cells with respect to HaCaT cells. However, functional assays confirmed potent inhibition predominantly by compound **63**, while compound **31** showed limited TRPM8 modulation, highlighting the limitations of relying solely on in silico affinity predictions and gene expression data. In parallel, TRPA1 emerged as an important complementary target, particularly for **31**, supported by bioinformatics (Table 4) and validated through functional assays (Table 6). For compound **63**, TRPA1 was not investigated in the early IVS but predicted during bioinformatic analyses, that were confirmed by functional assays, in which compound **63** showed EC_50_ in the micromolar range. Based on its structural and functional similarity to TRPM8, a retrospective docking study confirmed a plausible binding mode for **63** to TRPA1 (see SI Section 2: Predicted binding between **31** and **63** and TRPA1 and the related Figure S61).

Considering the involvement of these targets in regulating cellular calcium levels and oxidative stress, they may exert a complementary role in the action of compounds **31** and **63**.^66,67^ Furthermore, like TRPM8, TRPA1 expression is known to be altered in various cancers, including melanoma, and has been linked to increased tumor cell migration and invasiveness.^68,69^

The observed lipidomic alterations, including reduced CEs and DGs, both linked to TRP-regulated signaling, support these findings. The depletion of these lipid species, which accumulate in metastatic melanoma, suggests that TRP channel modulation may disrupt lipid-dependent oncogenic signaling such as Wnt/β-catenin.^70^

Proteomic analysis confirmed the involvement of calcium and redox signaling pathways. Downregulation of proteins involved in antioxidant defense (e.g., SOD1, TXNL1) and endoplasmic reticulum (ER) stress responses (e.g., NUCB1) aligned with ROS accumulation and disruption of protein folding, as seen in ROS assays and protein misfolding markers. TRP inhibition-induced calcium imbalance also impacted mitochondrial homeostasis and autophagy, as reflected in altered expression of CDH2, SNCA, and components of the unfolded protein response (UPR).^71–73^ This is further supported by the increase of ceramides, which can trigger apoptosis by calcium overload both in the mitochondria and ER.^74^

In HaCaT cells, which express negligible levels of TRPM8 and TRPA1, these stress responses were not activated, consistent with their resistance to treatment. The damage triggered by the interaction with TRP channels are corroborated by the cellular assays that confirm an important effect on production of ROS (Figure 9A,B) and induction of protein misfolding (Figure 9C–F) for both derivatives **31** and **63** in A375 melanoma cells.

Similarly, CB2R emerged as a shared target in both IVS and bioinformatic analyses. CB2R is known to modulate signaling pathways associated with cell proliferation, immune evasion, and apoptosis, making it a compelling target for anticancer therapeutics.^75–77^ Furthermore, the ability of **31** and **63** to act on TRP channels suggests a strong likelihood that they also function on cannabinoid receptors due to similarities in their ligand-binding pockets. Both TRPM8 and CB receptors possess hydrophobic domains that interact with lipophilic ligands, such as cannabinoids. This overlap in binding properties aligns with growing evidence that TRP channels can be considered ionotropic cannabinoid receptors.^78^

Targeting CB2R in melanoma represents a promising therapeutic approach due to its overexpression and its role in multiple key antitumor pathways.^79–81^ Unfortunately, no studies about CB2R inverse agonists in melanoma cells have been carried out, this is why no correlations between this kind of modulation and the protein intracellular rearrangement can be discussed. It could be plausible that, considering the CB2R overexpression in the A375 melanoma cell line,^79^ the inverse modulation of this receptor may counteract its function in cancer progression.

Although the CB1 receptor was initially predicted as a potential target for compound **31** based on bioinformatic analyses, it was not supported by IVS. Subsequent biological assays confirmed that CB1 does not show any appreciable interaction or modulation by the tested compounds (Table 6), excluding it from the list of relevant targets. This discrepancy highlights the limitations of relying solely on gene expression-based prediction tools, especially for promiscuous or widely expressed targets such as CB1.

BRD4, which was highlighted as a potential target in the IVS analysis (Tables 2 and 3), was also confirmed as an interacting target through functional assays, further supporting its relevance in the anticancer activity of the test compounds.

BRD4 plays a crucial role in regulating transcription by binding to acetylated lysines on histones, which facilitate an open chromatin structure and promote gene expression.^82^ When BRD4 is inhibited, this interaction is disrupted, leading to chromatin compaction, reduced transcription, and the accumulation of faulty or incomplete RNA. This is further supported by downregulation of cyclin-kinase dependent kinase 9 (P50750_CDK9) and upregulation of splicing and RNA-processing factors (e.g., CASC3, LSM12, RNase P).^83,84^ Proteomic changes further showed alterations in histone acetylation dynamics (e.g., increased HDAC1, decreased RBBP4), suggesting compensatory chromatin remodeling. These changes reflect a cellular attempt to maintain transcriptional homeostasis under BRD4 inhibition-induced stress.^85–89^ Furthermore, a key confirmation of BRD4 inhibition by the compounds, particularly **63**, is the strong downregulation of CDK9, a critical BRD4-dependent kinase for transcriptional elongation.^90,91^

Beyond transcription and RNA processing, BRD4 inhibition triggers significant changes in proteostasis, as demonstrated by the upregulation of autophagic and ubiquitin–proteasome components (e.g., Sequestosome-1, Cullin-2, DCAF13), reinforcing a convergence of stress pathways induced by multiple targets.^92–94^

Finally, IVS highlighted potential activities for other proteins involved in tumorigenesis, including IDH, BRK, and serine/threonine-protein kinase B-raf (B-Raf) (Tables 2 and 3). While these targets were identified as promising, based on docking simulations, experimental validation revealed a more limited effectiveness (Table 6).

For IDH, weak activity was observed following treatment with compound **31** in enzymatic assays (IC_50_ = 14.92 ± 2.12 μM). This suggests that compound **31** interacts effectively with IDH, an enzyme involved in cellular metabolism and known for its role in oncogenic transformations, such as in tumors with IDH mutations.^95^

In contrast, BRK and B-Raf were also identified as potential targets through IVS (Tables 2 and 3), but the enzymatic assays showed less pronounced activity (Table 6). This may suggest that, while these proteins are involved in critical cellular signaling pathways, the interactions with the tested compounds may be less efficient or that other variables, such as the specificity of molecular interaction or the need for a particular cellular context, are required for full activation. Specifically, B-Raf, which is known for its activating mutations in many cancers, may require more specific conditions to be fully inhibited or modulated.^96^

This study illustrates a novel and integrative framework for target deconvolution in phenotypic screening, combining IVS, bioinformatic predictions, transcriptomic profiling, and orthogonal experimental validation. The simultaneous application of these methodologies enabled robust prioritization and significantly enhanced confidence in target assignment. Unlike conventional linear approaches, our strategy provides a chemically guided, multidimensional workflow that better captures the complexity of ligand–target interactions in phenotypic screening. This integration of diverse data layers is particularly valuable in the context of DOS-based libraries, where chemical diversity and unknown modes of action demand robust target deconvolution strategies.

Crucially, the intersection of computational and transcriptomic data allowed us to identify convergent targets, such as TRPM8, TRPA1, CB2R, and BRD4, which were successfully validated in functional assays. However, several other targets, despite being prioritized by IVS and supported by either transcriptomics or combined bioinformatic layers, did not translate into biological activity upon testing (Table 6). This outcome highlights a critical limitation of the integrated strategy itself: while it improves specificity and prioritization, it cannot fully exclude false positives, that is why specific engagement assays are mandatory to confirm or discard the predicted results.

Additional limitations must be acknowledged. The prioritization process can be biased by target overexpression, particularly in transcriptomics, leading to apparent relevance that is not functionally supported. IVS remains restricted by the structural completeness and quality of protein models, which may exclude relevant targets lacking 3D structures or functional annotation. Transcriptomics, even if powerful, does not capture post-transcriptional regulation, protein localization, or activity, all of which are essential for biological validation.

Despite these limitations, the proposed framework offers a reproducible and scalable strategy for hit prioritization in phenotypic screens. The integration of orthogonal data layers, followed by biological testing, represents a valuable advancement toward rational and mechanism-driven target deconvolution.

## Conclusions

In this paper, a new UnDOS approach is discussed. The new structures, obtained via the skeleton-diversity strategy, were phenotypically screened against a broad panel of tumor cell lines, and two compounds, **31** and **63,** were selected because of their activity and selectivity against the A375 melanoma cell line. The novelty of the present paper regards the setting up of a platform integrating the principal approaches currently used for the target deconvolution, namely Inverse Virtual Screening, bioinformatics, cellular assays, and omics studies. This implementation in pharmacological investigation allowed us to collect a complete overview of the cellular activity of derivatives **31** and **63**. Pointing on the best outcomes coming from the single studies, we identified different molecular targets for each compound. Taking advantage of more than one prediction and being supported by cellular studies, we were able to widen the field of exploration including a higher number of proteins potentially involved in the antitumor mechanism of our derivatives. The downstream validation of a considerable number of suggested targets highlighted the suitability of our integrated platform as a powerful tool for target deconvolution in untargeted-oriented scientific programs. In this pilot study, an important contribution was made by omics experiments. Omics allow reporting the punctual protein, lipid, and metabolite rearrangement following the compound administration, and so they represent the result of a specific cellular response to an external perturbation. Deeply analyzing the cellular modifications coming out of omics profiles, we were capable of interconnecting the plethora of pathways affected by compounds administration, pulling the string of the tremendous complexity of the cellular biochemistry.

One of the biggest advantages of this approach could be seen as the possibility of identifying new hit compounds acting on emerging targets. In this regard, our attempt to demonstrate the validity of this approach led to the identification of BRD4 as a target for both compounds, especially for compound **63**, characterized by a submicromolar inhibitory potency against it. This encouraging data paves the way for a future chemical optimization, starting from the **63** chemical structure, aimed to improve compound activity, as against the isolated protein, as well as in the cellular environment also considering its pharmacokinetic property amelioration. The other targets involved in the compound's mechanism of action, such as TRPM8 and TRPA1, are very actual too, in the antitumor field, so our derivatives, also in these cases, deserve to be implemented for their binding with the respective proteins. Again, the data related to CB2R functional activity evidenced an inverse agonism for compound **63**, that is an underexplored behavior worthy to be deeply investigated in melanoma cancer.

Moreover, it is worth highlighting how an UnDOS approach can be conveniently exploited for identifying small molecules endowed not only with anticancer activity but spanning different biological properties, using a systematic and multidisciplinary approach for target deconvolution.

Besides the identification of novel antitumor agents, one of the most relevant impacts of this work is to provide a generic workflow that can be appropriately modulated depending on the biological activity being studied. Based on the diseases of interest, a suitable phenotypic assay should be identified. This screening needs to quickly and clearly highlight a key change in phenotype, for example, effect on bacterial colony growth for potential antibacterial agents, reduction or modulation of inflammation for potential anti-inflammatory molecules. The choice of this assay is central for the identification of the most promising compounds. Then, the accurate analysis of the results obtained by the integration of multiple approaches, that are IVS, bioinformatics and omics studies, is able to shed light on the putative biological counterpart(s) that, in the end, needs to be properly validated.

Overall, with this investigation, we demonstrated how an UnDOS project can be proficiently carried out exploiting the integration of complementary methods for a complete target deconvolution, but also, we identified two interesting hit compounds suitable for a subsequent program of chemical optimization targeting several high-value antitumor targets.

## Experimental Section

### General

All the reagents for the synthesis were purchased from Merck (Milan, Italy) unless otherwise described, all solvents and additives were purchased by Merck (Darmstadt, Germany) and were reagent-grade for synthetic purposes while LC-MS grade for analytical purposes. Reactions were carried out with magnetic stirring in round-bottom flasks except for microwave-assisted reactions that were conducted using glass vials and microwave closed vessel apparatus (CEM, Discover 2.0, Charlotte, North Carolina). Moisture-sensitive reactions were conducted in oven-dried glassware under nitrogen stream, using freshly distilled solvents. TLC analysis of reaction mixtures was performed on precoated glass silica gel plates (F254, 0.25 mm, VWR International), while crude products were purified with the Isolera Spektra One automated flash chromatography (FC) system (Biotage, Uppsala, Sweden), using commercial silica gel cartridges (SNAP KP-Sil, Biotage). NMR spectra were recorded on a Bruker Avance 400 MHz apparatus, at room temperature. Chemical shifts were reported in δ values (ppm) relative to internal Me_4_Si for ^1^H and ^13^C NMR. *J* values were reported in hertz (Hz). ^1^H NMR peaks were described using the following abbreviations: s (singlet), bs (broad singlet), d (doublet), t (triplet), and m (multiplet). HR-MS spectra were recorded using an LTQ-Orbitrap-XL-ETD mass spectrometer (Thermo Scientific, Bremen, Germany), equipped with an ESI source. The purity of final compounds was assessed by ultrahigh-performance liquid-chromatography (UHPLC) analyses, performed on a Jasco Extrema LC 4000 (Jasco, Japan) consisting of an LC-Net CG cable controller, quaternary flow pump system PU-4285, a DG-4000-04 degasser, a UV-4075 detector, and an AS-4250 autosampler. Purity assessment UHPLC runs were carried out on an EVO C18 150 mm × 2.1 mm × 2.6 μm (100 Å) column (Phenomenex, Bologna, Italy). The optimal mobile phase consisted of 0.1% HCOOH/H_2_O v/v (A) and 0.1% HCOOH/ACN v/v (B). Analysis was performed in gradient elution as follows: 0–10.00 min, 5–95% B; 10–12.00 min, 95–95% B; 12–15.00 min, isocratic to 5% B. Flow rate was 0.5 mL min^–1^. The injection volume was set at 5 μL. Data acquisition was set in the range 190–800 nm and chromatograms were monitored at 254 nm. All the final compounds showed a purity ≥ 95% by HPLC analysis. Optical rotations were measured on an Atago Polax 2-L polarimeter at a concentration of 0.1 g/100 mL.

#### General Procedure A: Reductive Amination (**1**, **2**, **37**, **39**)

*L*-Leu-OMe, *L*-Phe-OMe, or intermediate **35** (1.2 equiv) was dissolved in dry MeOH, and salicylaldehyde, benzaldehyde, or isobutyraldehyde (1 equiv) was added. The mixture was allowed to react for 12 h under a nitrogen stream, at room temperature. Then, NaBH_4_ (2.5 equiv) was added portionwise, and the mixture was stirred for a further 3 h. The reaction was quenched using 10% aqueous solution of citric acid, the solvent was evaporated in vacuo, and the residue was dissolved in dichloromethane and washed with water (3 × 50 mL). The organic layer was separated, dried over anhydrous Na_2_SO_4_, filtered, and evaporated in vacuo. The crude products were purified by column chromatography using mixtures of *n*-hexane/ethyl acetate as eluent.

#### General Procedure B: Hydrolysis (**3**, **4**, **57**, **58**)

1.0 mmol of intermediate **1**, **2**, **16**, **17**, **55** or **56** was dissolved in MeOH (10 mL), added with a 2 M NaOH aqueous solution (1 mL) and stirred at reflux until complete conversion of the starting material. The reaction was quenched with a 2 M aqueous solution of HCl until pH 7, and the mixture was extracted three times with ethyl acetate (3 × 20 mL), dried over sodium sulfate, and concentrated under vacuum. The obtained intermediates were used in the next step without further purification.

#### General Procedure C: Coupling Reaction Employing HOBt and HBTU (**5**, **6**, **16**, **17**, **50**)

One mmol of the intermediate **3**, **4**, **15** or Boc-*L*-Leu-OH was dissolved in dichloromethane and HOBt (1.2 equiv), HBTU (1.2 equiv), DIPEA (2.4 equiv), and the proper amine (1.2 equiv) were added. The mixture was stirred at room temperature for 12 h. Then, the solvent was evaporated under reduced pressure, and the residue was dissolved in dichloromethane and washed with water (3 × 20 mL), a saturated solution of NaHCO_3_ (3 × 20 mL), and a solution of citric acid (10% w:w, 3 × 20 mL). The organic phase was extracted, dried over Na_2_SO_4_, filtered, and concentrated in vacuo. The crude products were purified by flash chromatography using mixtures of *n*-hexane/ethyl acetate as the mobile phase.

#### General Procedure D: Cyclization Employing 4-Nitrophenyl Chloroformate (**7**, **8**, **52**)

The proper intermediate **5**, **6,** or **51** was dissolved in THF, and 4-nitrophenyl chloroformate (1.1 equiv) and TEA (2 equiv) were added. The mixture was refluxed at 70 °C overnight. Then, the crude product was added with TBAF (3 equiv) and stirred for 3 h at room temperature. The mixture was evaporated, diluted with DCM, and washed with a saturated aqueous solution of sodium bicarbonate. The organic layer was dried over anhydrous sodium sulfate, filtered, and concentrated in vacuo. The desired intermediates or final compounds were isolated using mixtures dichloromethane/ethyl acetate as the eluent.

#### General Procedure E: Reduction of Ester (**9**, **10**)

The ester intermediate **1** or **2** (1 equiv) dissolved in THF (15 mL) was added to a suspension of LiAlH_4_ (3 equiv) in THF at 0 °C. The reaction mixture was stirred at 0 °C for 15 min. Then, the mixture was quenched by dropwise addition of ethyl acetate followed by a solution of citric acid (10% w:w) until pH 7. The aqueous layer was extracted with ethyl acetate (3 × 20 mL), and the organic layer was dried over anhydrous Na_2_SO_4_, filtered, and concentrated in vacuo. The desired intermediates were purified by flash chromatography using mixtures of *n*-hexane/ethyl acetate as the mobile phase.

#### General Procedure F: Mitsunobu reaction (**11**, **12**, **59**, **60**)

Intermediate **9**, **10**, **57** or **58** (1 equiv), the proper alcohol (1.2 equiv), and triphenylphosphine (1 equiv) were dissolved in anhydrous THF under a nitrogen atmosphere. Then, DIAD (1 equiv, in THF) was added dropwise at 0 °C. The reaction mixture was stirred at 0 °C for 1 h. Then, it was allowed to warm to room temperature and was stirred overnight. The organic solvent was removed in vacuo, the crude product was dissolved in ethyl acetate, and a saturated aqueous solution of K_2_CO_3_ was added. The organic layer was extracted, dried on Na_2_SO_4_, filtered, and evaporated under vacuum. Flash chromatography of the crude products over silica gel using mixtures of *n*-hexane and ethyl acetate as the mobile phase furnished the desired compounds.

#### General Procedure G: *N*-Alkylation (**13**, **14**, **28**–**31**, **33**, **34**, **48**, **49**)

The proper intermediate **11**, **12, 26**, **27**, **32**, **46**, or **47** (1.0 equiv) was dissolved in a mixture of DCM/DMF (4:1, v:v) and stirred at 0 °C. To this solution, NaH (1.5 equiv), benzyl bromide, 1-bromo-2-methylpropane, or 4-(trifluoromethyl)­benzyl bromide (1.5 equiv) in DMF was added dropwise and the reaction mixture was warmed to room temperature and maintained under stirring for 5–7 h. The reaction mixture was quenched with 10% aqueous solution of citric acid and washed with brine. The organic layer was separated, dried over anhydrous Na_2_SO_4_, filtered, and evaporated in vacuo. Crude products were purified by flash chromatography using mixtures of *n*-hexane/ethyl acetate or dichloromethane/ethyl acetate as the eluent.

#### General Procedure H: Esterification Employing DCC and DMAP (**18**, **19**)

To a solution of the carboxylic acid (1.0 equiv) in THF, DMAP (0.7 equiv), *N*,*N*-dicyclohexylcarbodiimide (DCC, 1.2 equiv), and the proper alcohol (3.0 equiv) were added. After stirring at reflux temperature overnight, the urea byproduct was filtered off. The organic solvent was removed under reduced pressure, and the mixture was reconstituted in DCM and washed with water (3 × 20 mL), a saturated solution of NaHCO_3_ (3 × 20 mL), and a solution of citric acid (10% w:w, 3 × 20 mL). The combined organic layers were dried over anhydrous Na_2_SO_4_, filtered, and evaporated in vacuo. The desired intermediates were isolated by flash chromatography using mixtures of *n*-hexane/ethyl acetate as the mobile phase.

#### General Procedure I: Boc Removal (**20**, **21**, **24**, **25**, **41**, **44**, **45**, **51**, **61**, **62**)

The *N*-Boc protected intermediate **16–19**, **40**, **42**, **43**, **50**, **59** or **60** (0.2 mmol) was dissolved in a mixture of TFA/DCM (1:3, v:v) and added with TIS (0.25 equiv). The reaction mixture was stirred at room temperature for 1–2 h. After the completion of the reaction, monitored by TLC, a solution of NaOH (2 N) was added until pH 8. The mixture was diluted with water and dichloromethane, and the organic phase was extracted, dried over Na_2_SO_4_, filtered, and concentrated under vacuum. The desired intermediates were isolated by a precipitation step employing dichloromethane and diethyl ether.

#### General Procedure J: Synthesis of Quinazoline-2,4­(1*H*,3*H*)-Dione Compounds (**22**, **23**)

A 10 mL vial charged with the proper aminic intermediate **20** or **21** (1.0 mmol), di-*tert*-butyl dicarbonate (3 equiv), and DMAP (0.7 equiv) in ACN (5 mL) was heated under microwave radiation at 120 °C for 1 h. After cooling to room temperature, the mixture was evaporated to dryness, reconstituted in DCM, washed with brine, dried over anhydrous Na_2_SO_4_, and filtered. After concentrating in vacuo, crude products were purified by flash chromatography using mixtures of *n*-hexane/ethyl acetate as the eluent.

#### General Procedure K: Synthesis of 3,4-Dihydro-1*H*-benzo­[*e*]­[1,4]­diazepine-2,5-dione Compounds (**26**, **27**)

Intermediate **24** or **25** (1 mmol) was dissolved in 8 mL of ACN, 8 mL of water and 13 mL of acetic acid and the mixture was refluxed for 12 h. Then the reaction mixture was quenched with NaOH 2N and extracted three times with dichloromethane. The organic phase was dried over Na_2_SO_4_, filtered, and concentrated under vacuum. The crude products were purified by flash chromatography using *n*-hexane and ethyl acetate as the mobile phase.

#### General Procedure L: Synthesis of 1,2,3,4-Tetrahydro-5*H*-benzo­[*e*]­[1,4]­diazepin-5-one and 2,3,4,5-Tetrahydro-1*H*-benzo­[*e*]­[1,4]­diazepine Compounds (**32**, **35**, **36**)

To a stirring solution of the 3,4-dihydro-1*H*-benzo­[*e*]­[1,4]­diazepine-2,5-dione compound **26** or **27** in THF at 0 °C, 2 equiv of 2 M LiAlH_4_ solution in THF was added. The mixture was warmed at 80 °C, refluxed overnight, and subsequently quenched by dropwise addition of ethyl acetate followed by a saturated solution of potassium carbonate. The organic layer was separated, dried over Na_2_SO_4_, filtered, and concentrated under vacuum. The mono and bireduced intermediates were isolated by flash chromatography using mixtures of *n*-hexane/ethyl acetate as the eluent.

#### General Procedure M: Coupling Reaction Employing Mukaiyama Reagent (**40**, **42**, **43**)

To a stirring solution of Boc-*L*-Phe-OH or Boc-*L*-Leu-OH (2 equiv), Mukaiyama reagent (2.5 equiv), and TEA (2.5 equiv) in DCM, 2-iodoaniline or intermediate **39** (0.1 mmol) was added. The solution was refluxed for 12 h. Then, the crude product was washed with water (3 × 50 mL), a 10% aqueous solution of citric acid (3 × 50 mL), and a saturated aqueous solution of sodium bicarbonate (3 × 50 mL). The organic phase was extracted, and the combined organic layers were dried over anhydrous sodium sulfate, filtered, concentrated, and purified by flash chromatography employing mixtures of *n*-hexane/ethyl acetate as the mobile phase.

#### General Procedure N: Synthesis of 3,4-Dihydroquinoxalin-2­(1*H*)-one (**46**, **47**)

Intermediate **44** or **45** (1 mmol) was dissolved in H_2_O (0.2 mL), and DMF (2 mL), Pd­(PPh_3_)_4_ (0.05 equiv), CuI (0.05 equiv), K_2_CO_3_ (1 equiv), and TEA (3 equiv) were added. The mixture was subjected to microwave irradiation for 1 h at 170 °C. Then, the reaction mixture was washed three times with a saturated aqueous solution of K_2_CO_3_ (3 × 50 mL). The organic phase was extracted, dried over Na_2_SO_4_, filtered, and concentrated under vacuum. The crude products were purified by flash chromatography using mixtures of ethyl acetate/methanol as the mobile phase.

#### General Procedure O: *N*-alkylation of Amino Acid Methyl Ester (**55**, **56**)

To a suspension of *L*-Leu-OMe or *L*-Phe-OMe (1 mmol) and molecular sieves (1.0 g) in DMF, lithium hydroxide monohydrate (2 equiv) was added. After stirring at room temperature for 20 min, intermediate **54** (1 equiv) was added and the mixture was stirred overnight. Then, the organic layer was washed with a saturated aqueous solution of K_2_CO_3_ (3 × 20 mL) dried over anhydrous sodium sulfate, filtered, and concentrated under reduced pressure. The residue was purified by flash chromatography using mixtures of *n*-hexane/ethyl acetate as the mobile phase.

#### General Procedure P: Synthesis of 3,4-Dihydroquinazolin-2­(1*H*)-one (**63**, **64**)

A solution of diamine intermediate **61** or **62** (0.1 mmol), triphosgene (0.4 equiv), and TEA (2 equiv) in DCM was stirred at room temperature for 30 min. Then, water was added, and the mixture was extracted with dichloromethane (3 × 20 mL); the organic layer was dried over Na_2_SO_4_, filtered, and evaporated. Flash chromatography using *n*-hexane/ethyl acetate as the mobile phase furnished the corresponding urea compounds.

##### Methyl (2-Hydroxybenzyl)-*L*-leucinate (**1**)

Synthesized from salicylaldehyde and *L*-Leu-OMe as an off-white solid in 85% yield using general procedure A.

Precipitated from DCM/diethyl ether. ^1^H NMR (400 MHz, CD_3_OD) δ: 0.90 (dd, 6H, C*H*_3_, *J*′ = 6.6 Hz, *J*″ = 12.6 Hz); 1.53 (t, 2H, C*H*_2_, *J* = 7.2 Hz); 1.65–1.74 (m, 1H, C*H*); 3.34–3.37 (m, 1H, C*H*); 3.69–3.72 (m, 4H, C*H* and C*H*_3_); 3.92 (d, 1H, C*H*, *J* = 13.6 Hz); 6.76–6.80 (m, 2H, aryl); 7.05 (d, 1H, aryl, *J* = 7.0 Hz); 7.12 (d, 1H, aryl, *J* = 6.1 Hz);. HR-MS *m*/*z* calcd for C_14_H_21_NO_3_ [(M + H)]^+^: 252.1594; found 252.1544.

##### Methyl (2-Hydroxybenzyl)-*L*-phenylalaninate (**2**)

Intermediate **2** was synthesized from salicylaldehyde and *L*-Phe-OMe as pale-yellow oil using general procedure A. FC in *n*-hexane/ethyl acetate 3/1, *R*_f_: 0.35 (87% yield).

^1^H NMR (400 MHz, CDCl_3_) δ: 2.98 (dd, 1H, C*H*, *J*′ = 7.4 Hz; *J*″ = 13.7 Hz); 3.10 (dd, 1H, C*H*, *J*′ = 5.8 Hz; *J*″ = 13.7 Hz); 3.66 (t, 1H, C*H*, *J* = 7.3 Hz); 3.74 (d, 1H, C*H*, *J* = 13.6 Hz); 3.77 (s, 3H, C*H*_3_); 4.05 (d, 1H, C*H*, *J* = 13.6 Hz); 6.78 (t, 1H, aryl, *J* = 7.4 Hz); 6.86 (d, 1H, aryl, *J* = 8.1 Hz); 6.96 (d, 1H, aryl, *J* = 6.6 Hz); 7.17–7.22 (m, 3H, aryl); 7.28–7.37 (m, 3H, aryl). HR-MS *m*/*z* calcd for C_17_H_19_NO_3_ [(M + H)]^+^: 286.1438; found 286.1469.

##### (2-Hydroxybenzyl)-*L*-leucine (**3**)

Intermediate **3** was synthesized starting from **1** following general procedure B. Off-white powder (89% yield).

^1^H NMR (400 MHz, CD_3_OD) δ: 0.86 (s, 9H, C*H*_3_); 2.82 (dd, 1H, C*H*_2_, *J*′ = 8.0 Hz, *J*″ = 13.5 Hz); 2.97–3.02 (m, 3H, C*H* and C*H*_2_). 4.46 (dd, 1H, C*H*, *J*′ = 6.4 Hz; *J*″ = 8 Hz); 3.61 (d, 1H, C*H*, *J* = 13.3 Hz); 3.84 (d, 1H, C*H*, *J* = 13.2 Hz); 6.71–6.76 (m, 2H, aryl); 6.98 (d, 1H, aryl, *J* = 6.1 Hz); 7.09 (t, 1H, aryl, *J* = 6.1 Hz); 7.19–7.29 (m, 5H, aryl). HR-MS *m*/*z* calcd for C_13_H_19_NO_3_ [(M + H)]^+^: 238.1438; found 238.1433.

##### (2-Hydroxybenzyl)-*L*-phenylalanine (**4**)

Intermediate **4** was synthesized starting from **2** following general procedure B. Off-white powder (92% yield).

^1^H NMR (400 MHz, CD_3_OD) δ: 3.31 (d, 2H, C*H*_2_, *J* = 6.9 Hz); 4.19 (t, 1H, C*H*, *J* = 6.8 Hz); 4.28 (dd, 2H, C*H*_2_, *J*′ = 13.0 Hz, *J*″ = 15.2 Hz); 6.91 (d, 2H, aryl, *J* = 7.8 Hz); 7.26–7.40 (m, 7H, aryl). HR-MS *m*/*z* calcd for C_16_H_17_NO_3_ [(M + H)]^+^: 272.1281; found 272.1276.

##### (*S*)-*N*-Benzyl-2-((2-hydroxybenzyl)­amino)-4-methylpentanamide (**5**)

Intermediate **5** was synthesized from intermediate **3** and benzylamine according to general procedure C. FC in *n*-hexane/ethyl acetate 1/1, *R*_f_: 0.47. Yellowish oil (43%).

^1^H NMR (400 MHz, CD_3_OD) δ: 0.99 (t, 6H, C*H*_3_, *J* = 6.6 Hz); 1.53–1.60 (m, 1H, C*H*); 1.81–1.88 (m, 1H, C*H*); 1.94–2.02 (m, 1H, C*H*); 4.48 (d, 1H, C*H*, *J* = 14.6 Hz); 4.60 (d, 1H, C*H*, *J* = 14.6 Hz); 4.91–4–95 (m, 1H, C*H*); 7.04 (d, 1H, aryl, *J* = 8.2 Hz); 7.18 (t, 1H, aryl, *J* = 7.6 Hz); 7.27 (d, 1H, aryl, *J* = 7.3 Hz); 7.32 (t, 1H, aryl, *J* = 6.9 Hz). HR-MS *m*/*z* calcd for C_20_H_26_N_2_O_2_ [(M + H)]^+^: 327.2067; found 327.2094.

##### (*S*)-2-((2-Hydroxybenzyl)­amino)-*N*-neopentyl-3-phenylpropanamide (**6**)

Intermediate **6** was synthesized from intermediate **4** and *tert*-butylamine according to general procedure C. FC in *n*-hexane/ethyl acetate 2/3, *R*_f_: 0.44. Yellowish oil (50%).

^1^H NMR (400 MHz, CD_3_OD) δ: 0.99 (dd, 6H, C*H*_3_, *J*′ = 6.6 Hz, *J*″ = 8.8 Hz); 1.50–1.60 (m, 1H, C*H*); 1.76–1.83 (m, 1H, C*H*); 1.87–1.95 (m, 1H, C*H*); 4.39 (dd, 2H, C*H*_2_, *J*′ = 14.9 Hz, *J*″ = 25.0 Hz; 4.51 (d, 1H, C*H*, *J* = 14.8 Hz); 4.60 (d, 1H, C*H*, *J* = 14.8 Hz); 5.00 (dd, 1H, C*H*, *J*′ = 5.6 Hz, *J*″ = 10.1 Hz); 7.03 (d, 1H, aryl, *J* = 8.1 Hz); 7.15–7.34 (m, 8H, aryl). ^13^C NMR (100 MHz, CD_3_OD) δ: 20.6, 22.0, 24.7, 36.5, 42.6, 42.8, 57.3, 118.4, 124.4, 125.6, 126.8, 127.0, 128.1, 128.6, 138.5, 149.4, 152.6, 171.1. HR-MS *m*/*z* calcd for C_21_H_28_N_2_O_2_ [(M + H)]^+^: 341.2224; found 341.2244.

##### (*S*)-*N*-Benzyl-4-methyl-2-(2-oxo-2*H*-benzo­[*e*]­[1,3]­oxazin-3­(4*H*)-yl)­pentanamide (**7**)

Final derivative **7** was synthesized according to general procedure D, starting from intermediate **5**. FC in dichloromethane/ethyl acetate 9.8/0.2, *R*_f_ *:* 0.43. Yellowish oil (42%). [α]^25^_D_: −12.72 ± 0.25 (c = 0.10, MeOH).

^1^H NMR (400 MHz, CD_3_OD) δ: 0.99 (dd, 6H, C*H*_3_, *J*′ = 6.6 Hz, *J*″ = 8.8 Hz); 1.50–1.60 (m, 1H, C*H*); 1.76–1.83 (m, 1H, C*H*); 1.87–1.95 (m, 1H, C*H*); 4.39 (dd, 2H, C*H*_2_, *J*′ = 14.9 Hz, *J*″ = 25.0 Hz); 4.50 (d, 1H, C*H*, *J* = 14.8 Hz); 4.60 (d, 1H, C*H*, *J* = 14.8 Hz); 5.00 (dd, 1H, C*H*, *J*′ = 5.6 Hz, *J*″ = 10.1 Hz); 7.03 (d, 1H, aryl, *J* = 8.1 Hz); 7.15–7.34 (m, 8H, aryl). ^13^C NMR (100 MHz, CD_3_OD) δ: 20.6, 22.0, 24.7, 36.5, 42.6, 42.8, 57.3, 118.4, 124.4, 125.6, 126.8, 127.0, 128.1, 128.6, 138.5, 149.4, 152.6, 171.1. HR-MS *m*/*z* calcd for C_21_H_24_N_2_O_3_ [(M + Na)]^+^: 375.1679; found 375.1677.

##### (*S*)-*N*-Neopentyl-2-(2-oxo-2*H*-benzo­[*e*]­[1,3]­oxazin-3­(4*H*)-yl)-3-phenylpropanamide (**8**)

Final derivative **8** was synthesized according to general procedure D, starting from intermediate **6**. FC in dichloromethane/ethyl acetate 9.8/0.2, *R*_f_: 0.47. Yellowish oil (46%). [α]^25^_D_: −4.72 ± 0.46 (c = 0.10, MeOH).

^1^H NMR (400 MHz, CD_3_OD) δ: 0.65 (s, 9H, C*H*_3_); 3.05–3.14 (m, 3H, C*H* and C*H*_2_); 3.31 (dd, 1H, C*H*, *J*′ = 4.6 Hz, *J*″ = 14.8 Hz); 4.14 (t, 1H, C*H*, *J* = 4.2 Hz); 4.50 (d, 1H, C*H*, *J* = 14.7 Hz); 4.93 (d, 1H, C*H*, *J* = 14.7 Hz); 6.81–6.87 (m, 2H, aryl); 7.14–7.27 (m, 7H, aryl). ^13^C NMR (100 MHz, CD_3_OD) δ: 26.8, 33.5, 40.2, 49.6, 60.0, 115.0, 119.4, 121.8, 126.8, 128.1, 129.2, 129.7, 130.6, 134.6, 155.7, 157.9, 173.5. HR-MS *m*/*z* calcd for C_22_H_26_N_2_O_3_ [(M + H)]^+^: 367.2016; found 367.2017.

##### (*S*)-2-(((1-Hydroxy-4-methylpentan-2-yl)­amino)­methyl)­phenol (**9**)

Intermediate **9** was synthesized according to general procedure E, starting from **1**. White powder (77% yield). FC *n*-hexane/ethyl acetate 0.5/9.5, *R*_f_: 0.42.

^1^H NMR (400 MHz, CD_3_OD) δ: 0.90 (dd, 6H, C*H*_3_, *J*′ = 6.6 Hz, *J*″ = 12.9 Hz); 1.29–1.42 (m, 2H, C*H*_2_); 1.67–1.75 (m, 1H, C*H*); 2.68–2.74 (m, 1H, C*H*); 3.48 (dd, 1H, C*H*, *J*′ = 5.7 Hz, *J*″ = 11.2 Hz); 3.66 (dd, 1H, C*H*, *J*′ = 4.4 Hz, *J*″ = 11.2 Hz); 3.93 (dd, 2H, C*H*_2_, *J*′ = 13.5 Hz, *J*″ = 21.4 Hz); 6.74–6.77 (m, 2H, aryl); 7.08–7.11 (m, 2H, aryl). HR-MS *m*/*z* calcd for C_13_H_21_NO_2_ [(M + H)]^+^: 224.1645; found 224.1658.

##### (*S*)-2-(((1-Hydroxy-3-phenylpropan-2-yl)­amino)­methyl)­phenol (**10**)

Intermediate **10** was synthesized according to general procedure E, starting from **2**. White powder (81% yield). FC *n*-hexane/ethyl acetate 1/9, *R*_f_: 0.44.

^1^H NMR (400 MHz, CD_3_OD) δ: 2.76 (dd, 1H, C*H*, *J*′ = 7.4 Hz, *J*″ = 13.3 Hz); 2.83–2.94 (m, 2H, C*H*); 3.48 (dd, 1H, C*H*, *J*′ = 5.6 Hz, *J*″ = 11.4 Hz); 3.59 (dd, 1H, C*H*, *J*′ = 4.5 Hz, *J*″ = 11.2 Hz); 3.91 (dd, 2H, C*H*_2_, *J*′ = 13.5 Hz; *J*″ = 19.0 Hz); 6.72–6.78 (m, 2H, aryl); 7.06 (d, 1H, aryl, *J* = 6.2 Hz); 7.10 (t, 1H, aryl, *J* = 6.2 Hz); 7.19–7.22 (m, 3H, aryl); 7.27–7.30 (m, 2H, aryl). HR-MS *m*/*z* calcd for C_16_H_19_NO_2_ [(M + H)]^+^: 258.1489; found 258.1462.

##### (*S*)-3-Isobutyl-2,3,4,5-tetrahydrobenzo­[*f*]­[1,4]­oxazepane (**11**)

Intermediate **11** was synthesized according to general procedure F, starting from intermediate **9**. FC in *n*-hexane/ethyl acetate 3/1, *R*_f_: 0.48. White powder (69% yield).

^1^H NMR (400 MHz, CD_3_OD) δ: 0.81 (dd, 1H, C*H*, *J*′ = 6.7 Hz, *J*″ = 15.2 Hz); 1.13–1.20 (m, 1H, C*H*); 1.25–1.32 (m, 1H, C*H*); 1.36 (d, 1H, C*H*, *J* = 6.5 Hz); 1.48–1.54 (m, 1H, C*H*); 1.60 (d, 1H, C*H*, *J* = 6.5 Hz); 3.44 (d, 1H, C*H*, *J* = 13.8 Hz); 6.68 (t, 1H, aryl, *J* = 8.5 Hz); 6.80 (t, 2H, aryl, *J* = 7.0 Hz); 7.09 (t, H, aryl, *J* = 7.6 Hz). HR-MS *m*/*z* calcd for C_13_H_19_NO [(M + H)]^+^: 206.1539; found 206.1555.

##### (*S*)-3-Benzyl-2,3,4,5-tetrahydrobenzo­[*f*]­[1,4]­oxazepine (**12**)

Intermediate **12** was synthesized according to general procedure F, starting from intermediate **10**. FC in *n*-hexane/ethyl acetate 4/1, *R*_f_: 0.43. White powder (55% yield).

^1^H NMR (400 MHz, CDCl_3_) δ: 1.53 (d, 1H, C*H*, *J* = 6.4 Hz); 1.86 (d, 1H, C*H*, *J* = 3.8 Hz); 1.87–1.93 (m, 1H, C*H*); 2.74 (dd, 1H, C*H*, *J*′ = 5.7 Hz, *J*″ = 14.5 Hz); 2.86 (dd, 1H, C*H*, *J*′ = 6.1 Hz, *J*″ = 14.5 Hz); 3.48 (d, 1H, C*H*, *J* = 13.8 Hz); 3.77 (d, 1H, C*H*, *J* = 13.8 Hz); 6.80 (t, 1H, aryl, *J* = 6.3 Hz); 6.89 (d, 1H, aryl, *J* = 7.4 Hz); 6.93 (d, 1H, aryl, *J* = 8.1 Hz); 7.19–7.26 (m, 4H, aryl); 7.28–7.32 (m, 2H, aryl). HR-MS *m*/*z* calcd for C_16_H_17_NO [(M + H)]^+^: 240.1383; found 240.1355.

##### (*S*)-4-Benzyl-3-isobutyl-2,3,4,5-tetrahydrobenzo­[*f*]­[1,4]­oxazepine (**13**)

Final derivative **14** was synthesized from **12** as an off-white powder in 57% yield using general procedure G. FC in *n*-hexane/ethyl acetate 4/1, *R*_f_: 0.37. [α]^25^_D_: + 5.12 ± 0.15 (c = 0.10, MeOH).

^1^H NMR (400 MHz, CDCl_3_) δ: 0.99 (dd, 6H, C*H*_3_, *J*′ = 4.0 Hz, *J*″ = 6.6 Hz); 1.19–1.26 (m, 1H, C*H*); 1.44 (d, 1H, C*H*, *J* = 6.3 Hz); 1.46–1.58 (m, 3H, C*H* and C*H*_2_); 1.64 (d, 1H, C*H*, *J* = 3.3 Hz); 1.68–1.78 (m, 1H, C*H*); 3.45 (d, 1H, C*H*, *J* = 14.4 Hz); 3.66 (d, 1H, C*H*, *J* = 14.4 Hz); 5.10 (s, 2H, C*H*, *J*′ = 5.6 Hz, *J*″ = 10.1 Hz); 6.93 (d, 1H, aryl, *J* = 8.1 Hz); 7.01 (t, 1H, aryl, *J* = 7.4 Hz); 7.24 (t, 1H, aryl, *J* = 8.0 Hz); 7.35–7.46 (m, 5H, aryl); 7.57 (d, 1H, aryl, *J* = 7.6 Hz). ^13^C NMR (100 MHz, CDCl_3_) δ: 22.4, 22.9, 27.2, 34.4, 38.4, 42.2, 58.8, 69.9, 111.3, 120.8, 127.2, 127.8, 128.46, 128.52, 129.2, 137.3, 156.0. HR-MS *m*/*z* calcd for C_20_H_25_NO [(M + H)]^+^: 296.2009; found 296.2012.

##### (*S*)-3-Benzyl-4-isobutyl-2,3,4,5-tetrahydrobenzo­[*f*]­[1,4]­oxazepine (**14**)

Final derivative **13** was synthesized from **11** as an off-white powder in 53% yield using general procedure G. FC in *n*-hexane/ethyl acetate 3/1, *R*_f_: 0.41. [α]^25^_D_: −15.82 ± 0.44 (c = 0.10, MeOH).

^1^H NMR (400 MHz, CD_3_OD) δ: 0.92 (dd, 6H, C*H*_3_, *J*′ = 2.2 Hz, *J*″ = 6.7 Hz); 1.41 (d, 1H, C*H*, *J* = 6.0 Hz); 1.70–1.74 (m, 1H, C*H*); 1.95–2.01 (m, 1H, C*H*); 2.57 (dd, 1H, C*H*, *J*′ = 6.1 Hz, *J*″ = 14.6 Hz); 2.83 (dd, 1H, C*H*, *J*′ = 6.2 Hz, *J*″ = 14.6 Hz); 3.35 (d, 1H, C*H*_,_ *J* = 14.4 Hz); 3.52 (d, 1H, C*H*_,_ *J* = 14.3 Hz); 3.58 (dd, 2H, C*H*_2_, *J*′ = 2.6 Hz, *J*″ = 6.4 Hz); 6.71 (d, 1H, aryl, *J* = 8.2 Hz); 6.84 (t, 1H, aryl, *J* = 7.4 Hz); 7.10–7.21 (m, 6H, aryl); 7.39 (d, 1H, aryl, *J* = 7.4 Hz). ^13^C NMR (100 MHz, CD_3_OD) δ: 19.4, 28.4, 33.8, 39.5, 40.6, 58.5, 74.2, 110.8, 120.3, 126.1, 127.8, 128.3, 128.7, 129.1, 139.9, 156.4. HR-MS *m*/*z* calcd for C_20_H_25_NO [(M + H)]^+^: 296.2009; found 296.2014.

##### Synthesis of 2-((*tert*-Butoxycarbonyl)­amino)­benzoic Acid (**15**)

2-Aminobenzoic acid (1 equiv) and di-*tert*-butyl dicarbonate (1.43 equiv) were added to a solution of NaOH (0.5 M, 20.0 mL), 1,4-dioxane (10.0 mL), and ACN (2.0 mL) at 0 °C. The cold bath was removed, and the reaction mixture was stirred at room temperature for 16 h before quenching with 10% aqueous citric acid (30 mL). The mixture was diluted with H_2_O (50 mL) and extracted with DCM (3 × 20 mL). The organic phases were dried on Na_2_SO_4_, filtered, and concentrated in vacuo. Intermediate **15** was obtained without further purification in 98% yield as a white solid.

^1^H NMR (400 MHz, CDCl_3_) δ: 1.58 (s, 9H, C*H*_3_); 7.08 (t, 1H, aryl, *J* = 6.1 Hz); 8.14 (d, 1H, aryl, *J* = 6.2 Hz); 8.51 (d, 1H, aryl, *J* = 6.8 Hz); 10.07 (bs, 1H, N*H*). HR-MS *m*/*z* calcd for C_12_H_15_NO_4_ [(M + H)]^+^: 238.1074; found 238.1088.

##### Methyl (2-((*tert*-Butoxycarbonyl)­amino)­benzoyl)-*L*-phenylalaninate (**16**)

Intermediate **16** was synthesized from **15** and *L*-phenylalanine methyl ester following general procedure C as a yellowish oil in 80% of yield. FC in *n*-hexane/ethyl acetate 7/3, *R*_f_: 0.40.

^1^H NMR (400 MHz, CDCl_3_) δ: 1.54 (s, 9H, C*H*_3_); 3.22 (dd, 1H, C*H*, *J*′ = 5.8 Hz, *J*″ = 13.8 Hz); 3.32 (dd, 1H, C*H*, *J*′ = 5.6 Hz, *J*″ = 13.8 Hz); 3.79 (s, 3H, C*H*_3_); 5.07 (dd, 1H, C*H*, *J*′ = 5.8 Hz, *J*″ = 13.2 Hz); 6.66 (d, 1H, N*H*, *J* = 7.4 Hz); 7.28–7.36 (m, 4H, aryl); 7.44 (t, 1H, aryl, *J* = 7.2 Hz); 8.38 (d, 1H, aryl, *J* = 8.2 Hz); 10.03 (s, 1H, N*H*). HR-MS *m*/*z* calcd for C_22_H_26_N_2_O_5_ [(M + H)]^+^: 399.1914; found 399.1948.

##### Methyl (2-((*tert*-Butoxycarbonyl)­amino)­benzoyl)-*L*-leucinate (**17**)

Intermediate **17** was synthesized from **15** and *L*-leucine methyl ester following general procedure C as a yellowish oil in 90% of yield. FC in *n*-hexane/ethyl acetate 7/3, *R_f_*: 0.38.

^1^H NMR (400 MHz, CDCl_3_) δ: 0.98 (t, 6H, C*H*_3_, *J* = 5.8 Hz); 1.53 (s, 9H, C*H*_3_); 1.66–1.82 (m, 3H, C*H* and C*H*_2_); 3.76 (s, 3H, C*H*_3_); 4.84–4.90 (m, 1H, C*H*); 6.71 (d, 1H, N*H*, *J* = 8.1 Hz); 6.98 (t, 1H, aryl, *J* = 7.8 Hz); 7.44 (t, 1H, aryl, *J* = 7.2 Hz); 7.50 (d, 1H, aryl, *J* = 7.9 Hz); 8.39 (d, 1H, aryl, *J* = 8.0 Hz), 10.01 (s, 1H, N*H*). HR-MS *m*/*z* calcd for C_19_H_28_N_2_O_5_ [(M + H)]^+^: 365.2071; found 365.2094.

##### Isobutyl (2-((*tert*-Butoxycarbonyl)­amino)­benzoyl)-*L*-phenylalaninate (**18**)

Intermediate **18** was synthesized starting from 2-methylpropan-1-ol and **16**, which was previously subjected to general procedure B, using general procedure H. FC in *n*-hexane/ethyl acetate 8/2, *R_f_*: 0.47. Yellowish oil (65%).

^1^H NMR (400 MHz, CDCl_3_) δ: 0.95 (d, 6H, C*H*_3_, *J* = 7.9 Hz); 1.54 (s, 9H, C*H*_3_); 1.94–2.00 (m, 1H, C*H*); 3.21–3.34 (m, 2H, C*H*_2_); 3.91–4.01 (m, 2H, C*H*_2_); 5.07 (dd, 1H, C*H*, *J*′ = 5.8 Hz, *J*″ = 13.3 Hz); 6.63 (d, 1H, N*H*, *J* = 7.4 Hz); 6.98 (t, 1H, aryl, *J* = 7.8 Hz); 7.16–7.18 (m, 2H, aryl); 7.28–7.35 (m, 4H, aryl); 7.45 (t, 1H, aryl, *J* = 7.2 Hz); 7.38 (d, 1H, C*H*, *J* = 7.9 Hz); 10.03 (s, 1H, N*H*). HR-MS *m*/*z* calcd for C_25_H_32_N_2_O_5_ [(M + H)]^+^: 441.2384; found 441.2362.

##### Benzyl (2-((*tert*-Butoxycarbonyl)­amino)­benzoyl)-*L*-leucinate (**19**)

Intermediate **19** was synthesized starting from benzyl alcohol and **17**, which was previously subjected to general procedure B, using general procedure H. FC in *n*-hexane/ethyl acetate 9/1, *R*_f_: 0.48. Yellowish oil (58%).

^1^H NMR (400 MHz, CDCl_3_) δ: 0.97 (t, 6H, C*H*_3_, *J* = 5.6 Hz); 1.55 (s, 9H, C*H*_3_); 1.65–1.85 (m, 3H, C*H* and C*H*_2_); 4.86–4.92­(m, 1H, C*H*); 5.22 (s, 2H, C*H*_2_); 6.76 (d, 1H, N*H*, *J* = 8.0 Hz); 7.00 (t, 1H, aryl, *J* = 7.8 Hz); 7.35–7.39 (m, 4H, aryl); 7.42 (t, 1H, aryl, *J* = 7.2 Hz); 7.54 (d, 1H, aryl, *J* = 7.8 Hz); 8.42 (d, 1H, aryl, *J* = 8.1 Hz), 10.00 (s, 1H, N*H*). HR-MS *m*/*z* calcd for C_25_H_32_N_2_O_5_ [(M + H)]^+^: 441.2384; found 441.2397.

##### Isobutyl (2-Aminobenzoyl)-*L*-phenylalaninate (**20**)

Intermediate **20** was synthesized starting from **18** according to general procedure I as an off-white powder (93% yield).

Precipitated from DCM/diethyl ether. ^1^H NMR (400 MHz, CDCl_3_) δ: 0.90 (d, 6H, C*H*_3_, *J* = 7.8 Hz); 1.84–1.94 (m, 1H, C*H*); 3.14 (dd, 1H, C*H*, *J*′ = 8.8 Hz, *J*″ = 13.8 Hz); 3.27 (dd, 1H, C*H*, *J*′ = 8.8 Hz, *J*″ = 13.8 Hz); 3.90 (d, 2H, C*H*_2_, *J* = 5.7 Hz); 4.84 (dd, 1H, C*H*, *J*′ = 6.1 Hz, *J*″ = 8.8 Hz); 4.87 (bs, 2H, N*H*_2_); 6.60 (t, 1H, aryl, *J* = 7.0 Hz); 6.73 (d, 1H, aryl, *J* = 8.2 Hz); 7.15–7.23 (m, 2H, aryl); 7.26–7.31 (m, 4H, aryl); 7.39 (d, 1H, aryl, *J* = 6.6 Hz). HR-MS *m*/*z* calcd for C_20_H_24_N_2_O_3_ [(M + H)]^+^: 341.1860; found 341.1882.

##### Benzyl (2-Aminobenzoyl)-*L*-leucinate (**21**)

Intermediate **21** was synthesized starting from **19** according to general procedure I as an off-white powder (95% yield).

Precipitated from DCM/diethyl ether. ^1^H NMR (400 MHz, CD_3_OD) δ: 0.95 (dd, 6H, C*H*_3_, *J*′ = 6.1 Hz, *J*″ = 9.4 Hz); 1.69–1.82 (m, 3H, C*H* and C*H*_2_); 4.73 (t, 1H, C*H, J* = 4.9 Hz); 5.17 (dd, 2H, C*H*_2_, *J*′ = 12.3 Hz, *J*″ = 19.4 Hz); 6.63 (t, 1H, aryl, *J* = 8.1 Hz); 6.76 (d, 1H, aryl, *J* = 8.9 Hz); 7.19 (t, 1H, aryl, *J* = 7.2 Hz); 7.28–7.37 (m, 4H, aryl); 7.54 (d, 1H, aryl, *J* = 9.2 Hz). HR-MS *m*/*z* calcd for C_20_H_24_N_2_O_3_ [(M + H)]^+^: 341.1860; found 341.1888.

##### Isobutyl (*S*)-2-(2,4-Dioxo-1,4-dihydroquinazolin-3­(2*H*)-yl)-3-phenylpropanoate (**22**)

Synthesized from **20** as an off-white powder in 42% yield using general procedure J. FC in *n*-hexane/ethyl acetate 2/1, *R*_f_: 0.49. [α]^25^_D_: −55.42 ± 0.28 (c = 0.10, MeOH).

^1^H NMR (400 MHz, CD_3_OD) δ: 0.99 (dd, 6H, C*H*_3_, *J*′ = 2.4 Hz, *J*″ = 6.6 Hz); 1.86–1.96 (m, 1H, C*H*); 3.49 (dd, 1H, C*H*, *J*′ = 10.2 Hz, *J*″ = 14.0 Hz); 3.57 (dd, 1H, C*H*, *J*′ = 5.6 Hz, *J*″ = 14.0 Hz); 3.91–4.00 (m, 2H, C*H*_2_); 5.89 (dd, 1H, C*H*, *J*′ = 5.6 Hz, *J*″ = 10.2 Hz); 7.09–7.22 (m, 7H, aryl); 7.62 (t, 1H, aryl, *J* = 6.9 Hz); 7.93 (d, 1H, aryl, *J* = 8.0 Hz). ^13^C NMR (100 MHz, CD_3_OD) δ: 17.89, 17.91, 27.4, 34.1, 54.5, 71.2, 113.5, 114.7, 122.8, 126.2, 127.5, 127.9, 128.9, 135.2, 137.2, 139.3, 150.4, 162.4, 169.9. HR-MS *m*/*z* calcd for C_21_H_22_N_2_O_4_ [(M + Na)]^+^: 389.1472; found 389.1454.

##### Benzyl (*S*)-2-(2,4-Dioxo-1,4-dihydroquinazolin-3­(2*H*)-yl)-4-methylpentanoate (**23**)

Synthesized from **21** as an off-white powder in 44% yield using general procedure J. FC in *n*-hexane/ethyl acetate 2/1, *R*_f_: 0.48. [α]^25^_D_: −30.12 ± 0.34 (c = 0.10, MeOH).

^1^H NMR (400 MHz, CD_3_OD) δ: 0.93 (d, 3H, C*H*_3_, *J* = 6.7 Hz); 0.99 (d, 3H, C*H*_3_, *J*″ = 6.5 Hz); 1.51–1.58 (m, 1H, C*H*); 2.10–2.14 (m, 2H, C*H*_2_); 5.16 (dd, 2H, C*H*_2_, *J*′ = 12.4 Hz, *J*″ = 20.0 Hz); 5.66 (dd, 1H, C*H*, *J*′ = 6.2 Hz, *J*″ = 8.1 Hz); 7.17 (d, 1H, aryl, *J* = 8.2 Hz); 7.23–7.29 (m, 5H, aryl); 7.67 (t, 1H, aryl, *J* = 8.5 Hz); 8.03 (d, 1H, aryl, *J* = 9.2 Hz). ^13^C NMR (100 MHz, CD_3_OD) δ: 21.0, 22.1, 25.1, 37.6, 52.2, 66.7, 113.7, 114.8, 122.9, 127.60, 127.65, 127.8, 128.0, 135.3, 135.8, 139.4, 150.5, 162.6, 170.3. HR-MS *m*/*z* calcd for C_21_H_22_N_2_O_4_ [(M + Na)]^+^: 389.1472; found 389.1556.

##### Methyl (2-Aminobenzoyl)-*L*-phenylalaninate (**24**)

Intermediate **24** was synthesized following general procedure I, starting from intermediate **16**.

White powder (90% yield).

Precipitated from DCM/diethyl ether. ^1^H NMR (400 MHz, CD_3_OD) δ: 3.12 (dd, 1H, C*H*, *J*′ = 9.1 Hz, *J*″ = 13.8 Hz); 3.28 (dd, 1H, C*H*, *J*′ = 5.6 Hz, *J*″ = 13.8 Hz); 3.74 (s, 3H, C*H*_3_); 4.80–4.84 (m, 1H, C*H*); 6.61 (t, 1H, aryl, *J* = 7.9 Hz); 6.73 (d, 1H, aryl, *J* = 8.2 Hz); 7.18 (t, 1H, aryl, *J* = 7.0 Hz); 7.23–7.32 (m, 5H, aryl); 7.36 (d, 1H, aryl, *J* = 8.9 Hz). HR-MS *m*/*z* calcd for C_17_H_18_N_2_O_3_ [(M + H)]^+^: 299.1390; found 299.1399.

##### Methyl (2-Aminobenzoyl)-*L*-leucinate (**25**)

Intermediate **24** was synthesized following general procedure I, starting from intermediate **17**.

White powder (93% yield).

Precipitated from DCM/diethyl ether. ^1^H NMR (400 MHz, CD_3_OD) δ: 0.99 (t, 6H, C*H*_3_, *J* = 6.4 Hz); 1.68–1.82 (m, 3H, C*H* and C*H*_2_); 3.79 (s, 3H, CH_3_); 4.64–4.68 (m, 1H, C*H*); 6.64 (t, 1H, aryl, *J* = 7.7 Hz); 6.76 (d, 1H, aryl, *J* = 8.1 Hz); 7.20 (t, 1H, aryl, *J* = 7.2 Hz); 7.53 (d, 1H, aryl, *J* = 8.5 Hz). HR-MS *m*/*z* calcd for C_14_H_20_N_2_O_3_ [(M + H)]^+^: 265.1547; found 265.1557.

##### (*S*)-3-Benzyl-3,4-dihydro-1*H*-benzo­[*e*]­[1,4]­diazepine-2,5-dione (**26**)

Intermediate **26** was synthesized from intermediate **24** following general procedure K. FC in *n*-hexane/ethyl acetate 1/1, *R*_f_: 0.39. Off-white powder (20% yield).

^1^H NMR (400 MHz, CD_3_OD) δ: 3.10 (dd, 1H, C*H*, *J*′ = 8.5 Hz, *J*″ = 14.4 Hz); 3.46 (dd, 1H, C*H*, *J*′ = 6.4 Hz, *J*″ = 14.2 Hz); 4.06–4.4.12 (m, 1H, C*H*); 7.06–7.09 (m, 2H, aryl); 7.29–7.31 (m, 5H, aryl); 7.53 (t, 1H, aryl, *J* = 7.6 Hz); 7.94 (d, 1H, aryl, *J* = 7.7 Hz) 8.93 (s, 1H, N*H*). HR-MS *m*/*z* calcd for C_16_H_14_N_2_O_2_ [(M + H)]^+^: 267.1128; found 267.1144.

##### (*S*)-3-Isobutyl-3,4-dihydro-1*H*-benzo­[*e*]­[1,4]­diazepine-2,5-dione (**27**)

Intermediate **27** was synthesized from intermediate **25** following general procedure K. FC in *n*-hexane/ethyl acetate 1/2, *R*_f_: 0.41. Off-white powder (37% yield).

^1^H NMR (400 MHz, CD_3_OD) δ: 0.87 (d, 1H, C*H*, *J* = 6.3 Hz); 0.95 (d, 1H, C*H*, *J* = 6.4 Hz); 1.68–1.83 (m, 3H, C*H* and C*H*_2_); 3.79 (t, 1H, C*H*, *J* = 8.4 Hz); 7.15 (d, 1H, aryl, *J* = 8.1 Hz); 7.31 (t, 1H, aryl, *J* = 6.8 Hz); 7.57 (d, 1H, aryl, *J* = 7.5 Hz); 7.87 (d, 1H, aryl, *J* = 6.3 Hz). HR-MS *m*/*z* calcd for C_13_H_16_N_2_O_2_ [(M + H)]^+^: 233.1285; found 233.1295.

##### (*S*)-3-Benzyl-1-(4-(trifluoromethyl)­benzyl)-3,4-dihydro-1*H*-benzo­[*e*]­[1,4]­diazepine-2,5-dione (**28**)

Synthesized from **26** as an off-white oil in 48% yield using general procedure G. FC in *n*-hexane/ethyl acetate 8/2, *R*_f_: 0.48. [α]^25^_D_: + 133.72 ± 0.45 (c = 0.10, MeOH).

^1^H NMR (400 MHz, CD_3_OD) δ: 2.99 (dd, 1H, C*H*, *J*′ = 7.8 Hz, *J*″ = 14.4 Hz); 3.38 (dd, 1H, C*H*, *J*′ = 6.7 Hz, *J*″ = 14.4 Hz); 4.07–4.12 (m, 1H, C*H*); 5.08 (s, 2H, C*H*_2_); 6.18 (d, 1H, N*H*, *J* = 5.2 Hz); 7.10 (d, 1H, aryl, *J* = 8.2 Hz); 7.15–7.26 (m, 8H, aryl); 7.39–7.47 (m, 3H, aryl); 7.77 (d, 1H, aryl, *J* = 6.5 Hz). ^13^C NMR (100 MHz, CD_3_OD) δ: 34.8, 34.9, 52.0, 53.6, 122.0, 125.8, (q, ^2^ *J*_C–F_= 3.8 Hz), 126.6, 126.7 (q, ^1^ *J*_C–F_= 271.4 Hz), 127.1, 127.3, 128.8, 128.9, 129.3, 130.7, 132.8, 135.9, 139.8, 240.7. HR-MS *m*/*z* calcd for C_24_H_19_F_3_N_2_O_2_ [(M + H)]^+^: 425.1471; found 425.1471.

##### (*S*)-3-Isobutyl-1-(4-(trifluoromethyl)­benzyl)-3,4-dihydro-1*H*-benzo­[*e*]­[1,4]­diazepine-2,5-dione (**29**)

Obtained from **27** as an off-white oil in 45% yield using general procedure G. FC in *n*-hexane/ethyl acetate 9/1, *R*_f_: 0.46. [α]^25^_D_: + 24.62 ± 0.26 (c = 0.10, MeOH).

^1^H NMR (400 MHz, CDCl_3_) δ: 0.90 (d, 1H, C*H*, *J* = 6.4 Hz); 0.97 (d, 1H, C*H*, *J* = 6.4 Hz); 1.72–1.87 (m, 2H, C*H*_2_); 1.93–2.00 (m, 1H, C*H*); 3.91 (dd, 1H, C*H*, *J*′ = 6.0 Hz, *J*″ = 13.9 Hz); 5.16 (dd, 1H, C*H*, *J*′ = 15.9 Hz, *J*″ = 22.0 Hz); 7.00 (d, 1H, N*H*, *J* = 5.7 Hz); 7.21 (d, 1H, aryl, *J* = 8.1 Hz); 7.28–7.36 (m, 3H, aryl); 7.51 (t, 1H, aryl, *J* = 8.3 Hz); 7.57 (d, 1H, aryl, *J* = 8.1 Hz); 7.91 (d, 1H, aryl, *J* = 7.8 Hz). ^13^C NMR (100 MHz, CDCl_3_) δ: 22.1, 22.8, 24.5, 37.4, 50.7, 51.9, 122.0, 124.0 (q, ^1^ *J*_C–F_= 272.1 Hz), 125.78 (q, ^3^ *J*_C–F_= 3.8 Hz), 125.82, 126.5, 127.1, 128.9, 129.6, 129.7 (q, ^2^ *J*_C–F_= 32.5 Hz), 129.9, 130.5, 132.7, 140.1, 141.0, 168.7, 170.4. HR-MS *m*/*z* calcd for C_21_H_21_F_3_N_2_O_2_ [(M + H)]^+^: 391.1628; found 391.1614.

##### (*S*)-3-Benzyl-1,4-bis­(4-(trifluoromethyl)­benzyl)-3,4-dihydro-1*H*-benzo­[*e*]­[1,4]­diazepine-2,5-dione (**30**)

Synthesized from **26** as an off-white powder in 39% yield using general procedure G. FC in *n*-hexane/ethyl acetate 6/4, *R*_f_: 0.42. [α]^25^_D_: −33.62 ± 0.44 (c = 0.10, MeOH).

Spectra are described as two atropisomers in agreement with the literature.^97^

**A** ^1^H NMR (400 MHz, CD_3_OD) δ: 2.55–2.69 (m, 2H, C*H*_2_); 4.36 (d, 1H, C*H*, *J* = 14.9 Hz); 4.65 (t, 1H, C*H*, *J* = 9.3 Hz); 4.94–5.01 (m, 1H, C*H*); 5.08 (d, 2H, C*H*_2_, *J* = 15.9 Hz); 5.31 (d, 1H, C*H*, *J* = 7.5 Hz); 6.93 (d, 1H, aryl, *J* = 3.5 Hz); 7.03 (d, 2H, aryl, J= 8.1 Hz); 7.17–7.22 (m, 4H, aryl); 7.48–7.60 (m, 9H, aryl); 7.88 (dd, 1H, aryl, *J*′ = 1.6 and *J*″ = 7.8 Hz); **B** ^1^H NMR (400 MHz, CD_3_OD) δ: 3.16 (dd, 1H, C*H*, *J*′ = 5.7 and *J*″ = 13.6 Hz); 3.56 (dd, 1H, C*H*, *J*′ = 9.6 and *J*″ = 13.6 Hz); 4.77 (dd, 6H, C*H*_3_, *J*′ = 5.4 and *J*′ = 9.6 Hz); 4.92 (d, 2H, C*H*_2_, *J* = 15.8 Hz); 5.27 (d, 1H, C*H*, *J* = 7.2 Hz); 6.91 (d, 1H, aryl, *J* = 2.1 Hz); 7.13 (d, 2H, aryl, *J* = 8.1 Hz); 7.23–7.27 (m, 4H, aryl); 7.35–7.44 (m, 8H, aryl); 7.69–7.73 (m, 1H, aryl); 7.95 (dd, 1H, aryl, *J*′ = 1.6 and *J*″ = 6.4 Hz). ^13^C NMR (100 MHz, CD_3_OD) (**A** + **B**) δ: 32.5, 34.5, 45.8, 49.6, 51.0, 53.6, 57.5, 67.4, 121.6, 122.4, 124.96, 125.0, (q, ^3^ *J*_C–F_= 3.8 Hz), 125.1 (q, ^3^ *J*_C–F_= 3.8 Hz), 124.1 (q, ^1^ *J*_C–F_= 271.5 Hz), 124.2 (q, ^1^ *J*_C–F_= 271.4 Hz), 124.3 (q, ^1^ *J*_C–F_= 271.4 Hz), 125.11, 126.1, 126.5, 127.0, 127.3, 127.5, 128.1, 128.2, 128.5, 128.6, 128.7, 128.9, 129.4, 129.2 (q, ^2^ *J*_C–F_= 34.1 Hz), 129.5 (q, ^2^ *J*_C–F_= 38.8 Hz), 129.9, 130.1, 130.4, 132.7, 133.0, 135.4, 136.3, 138.6, 139.4, 140.6, 141.2, 141.8, 167.1, 168.4, 169.1, 169.3. HR-MS *m*/*z* calcd for C_32_H_24_F_6_N_2_O_2_ [(M + H)] ^+^: 583.1815; found 583.1803.

##### (*S*)-3-Isobutyl-1,4-bis­(4-(trifluoromethyl)­benzyl)-3,4-dihydro-1*H*-benzo­[*e*]­[1,4]­diazepine-2,5-dione (**31**)

Final derivative **31** was synthesized from **27** as an off-white powder in 42% yield using general procedure G. FC in *n*-hexane/ethyl acetate 1/1, *R*_f_: 0.42. [α]^25^_D_: + 32.42 ± 0.43 (c = 0.10, MeOH).

NMR analysis shows duplication of several ^13^C signals, indicating the presence of two diastereomeric species in solution. This is consistent with the presence of a stereogenic center and a hindered rotation around the benzodiazepindione, leading to a situation of atropisomerism. The resulting atropisomers are diastereomeric due to the fixed stereocenter (3S) and are observed as distinct species on the NMR time scale.^97^

**A** ^1^H NMR (400 MHz, CD_3_OD) δ: 0.77 (d, 3H, CH_3_, *J* = 6.6 Hz); 0.87 (d, 3H, CH_3_, *J* = 6.6 Hz); 1.37–1.43 (m, H, C*H*); 1.48–1.58 (m, H, C*H*); 2.12–2.19 (m, H, C*H*); 4.38 (dd, 1H, C*H*, *J*′ = 4.6 and *J*″ = 7.4 Hz); 4.79 (t, 2H, C*H*_2_, *J* = 14.7 Hz); 5.48 (t, 2H, C*H*_2_, *J* = 17.0 Hz); 7.13 (d, 2H, aryl, *J* = 8.1 Hz); 7.53 (t, 5H, aryl, *J* = 8.1 Hz); 7.56–7.59 (m, 4H, aryl); 7.86 (dd, 1H, aryl, *J*′ = 1.5 and *J*″ = 7.8 Hz); **B** ^1^H NMR (400 MHz, CD_3_OD) δ: 0.72 (d, 3H, CH_3_, *J* = 6.6 Hz); 0.87 (d, 3H, CH_3_, *J* = 6.6 Hz); 1.09–1.23 (m, 2H, C*H*_2_); 1.65–1.72 (m, H, C*H*); 4.40 (t, 1H, C*H*, *J* = 2.6 Hz); 4.87–4.91 (m, 1H, C*H*); 5.01 (dd, 2H, C*H*_2_, *J*′ = 9.6 and *J*″ = 15.6 Hz); 5.12 (d, 1H, C*H*, *J* = 14.8 Hz); 7.21 (d, 2H, aryl, *J* = 8.1 Hz); 7.40 (t, 3H, aryl, *J* = 6.6 Hz); 7.47 (d, 4H, aryl, *J* = 8.2 Hz); 7.63 (t, 2H, aryl, *J* = 7.3 Hz); 7.81 (dd, 1H, aryl, *J*′ = 5.6 and *J*″ = 7.8 Hz). ^13^C NMR (100 MHz, CD_3_OD) (**A** + **B**) δ: 20.8, 20.9, 21.4, 21.6, 24.9, 25.7, 35.2, 38.3, 45.6, 48.5, 49.2, 50.5, 53.3, 54.6, 64.8, 122.0, 122.3, 124.1 (q, ^1^ *J*_C–F_= 271.4 Hz), 124.2 (q, ^1^ *J*_C–F_= 271.4 Hz), 124.9 (q, ^3^ *J*_C–F_= 3.7 Hz), 125.1 (q, ^3^ *J*_C–F_= 3.7 Hz), 125.2, 126.3, 127.6, 127.7, 128.9, 129.2 (q, ^2^ *J*_C–F_= 33.9 Hz), 129.3 (q, ^2^ *J*_C–F_= 33.9 Hz), 129.9, 130.1, 132.7, 132.8, 138.3, 139.2, 140.9, 141.3, 141.9, 167.1, 169.2, 169.5, 170.0. HR-MS *m*/*z* calcd for C_29_H_26_F_6_N_2_O_2_ [(M + H)]^+^: 549.1971; found 549.1959.

##### (*S*)-3-Isobutyl-1,2,3,4-tetrahydro-5*H*-benzo­[*e*]­[1,4]­diazepin-5-one (**32**)

Synthesized from **27** as an off-white powder in 42% yield using general procedure L. FC in *n*-hexane/ethyl acetate 3/2, *R*_f_: 0.44.

^1^H NMR (400 MHz, CDCl_3_) δ: 0.94 (dd, 6H, C*H*_3_, *J*′ = 6.6 Hz, *J*″ = 9.4 Hz); 1.33–1.40 (m, 1H, C*H*); 1.45–1.56 (m, 1H, C*H*); 1.73–1.79 (m, 1H, C*H*); 3.36 (dd, 1H, CH, *J*′ = 7.8 Hz, *J*″ = 12.7 Hz); 3.47 (dd, 1H, CH, *J*′ = 7.7 Hz, *J*″ = 12.5 Hz); 3.60–3.66 (m, 1H, C*H*); 4.46 (bs, 1H, N*H*); 6.47 (s, 1H, N*H*); 6.60 (d, 1H, N*H*, *J* = 8.2 Hz); 6.79 (t, 1H, aryl, *J* = 8.0 Hz); 7.24 (t, 1H, aryl, *J* = 5.7 Hz); 8.05 (d, 1H, aryl, *J* = 6.6 Hz). HR-MS *m*/*z* calcd for C_13_H_18_N_2_O [(M + H)]^+^: 219.1492; found 219.1488.

##### (*S*)-3-Isobutyl-4-(4-(trifluoromethyl)­benzyl)-1,2,3,4-tetrahydro-5*H*-benzo­[*e*]­[1,4]­diazepin-5-one (**33**)

Synthesized from **32** as a whitish oil in 53% yield using the general procedure G. FC in *n*-hexane/ethyl acetate 8/2, *R*_f_: 0.45. [α]^25^_D_: −90.12 ± 0.44 (c = 0.10, MeOH).

^1^H NMR (400 MHz, CDCl_3_) δ: 0.74 (d, 3H, C*H*_3_, *J* = 6.4 Hz); 0.80 (d, 1H, C*H*_3_, *J* = 6.4 Hz); 1.20–1.27 (m, 1H, C*H*); 1.41–1.53 (m, 2H, C*H*_2_); 3.14 (d, 1H, C*H*, *J* = 14.9 Hz); 3.31 (dd, 1H, C*H*, *J*′ = 5.6 Hz, *J*″ = 13.5 Hz); 3.48 (dd, 1H, C*H*, *J*′ = 6.3 Hz, *J*″ = 12.7 Hz); 4.26 (d, 1H, C*H*, *J* = 14.9 Hz); 4.48 (bs, 1H, N*H*); 5.31 (d, 1H, C*H*, *J* = 14.9 Hz); 6.50 (d, 1H, aryl, *J* = 8.2 Hz); 6.68 (t, 1H, aryl, *J* = 8.0 Hz); 7.13 (t, 1H, aryl, *J* = 6.8 Hz); 7.40 (d, 2H, aryl, *J* = 8.0 Hz); 7.50 (d, 2H, aryl, *J* = 8.2 Hz); 8.11 (d, 1H, aryl, *J* = 6.7 Hz). ^13^C NMR (100 MHz, CDCl_3_) δ: 22.0, 23.1, 25.0, 39.2, 48.8, 53.1, 56.4, 116.9, 117.3, 117.6, 124.1 (q, ^1^ *J*_C–F_= 273.4 Hz), 125.5 (q, ^3^ *J*_C–F_= 3.7 Hz), 125.6, 128,6, 129.6, 129.7 (q, ^2^ *J*_C–F_= 32.3 Hz), 129.9, 132.1, 134.5, 141.7, 145.5, 168.5. HR-MS *m*/*z* calcd for C_21_H_23_F_3_N_2_O [(M + H)]^+^: 377.1835; found 377.1835.

##### (*S*)-3-Isobutyl-1,4-bis­(4-(trifluoromethyl)­benzyl)-1,2,3,4-tetrahydro-*5H*-benzo­[*e*]­[1,4]­diazepin-5-one (**34**)

Synthesized from **32** as an off-white powder in 38% yield using general procedure G. FC in *n*-hexane/ethyl acetate 9.5/0.5, *R*_f_: 0.48. [α]^25^_D_: −32.35 ± 0.36 (c = 0.10, MeOH).

^1^H NMR (400 MHz, CDCl_3_) δ: 0.79 (dd, 6H, C*H*_3_, *J*′ = 6.2 Hz, *J*″ = 14.1 Hz); 1.23–1.28 (m, 1H, C*H*); 1.44–1.52 (m, 2H, C*H*_2_); 2.99–3.01 (m, 2H, C*H*_2_); 3.75–3.81 (m, 1H, C*H*); 4.28 (d, 1H, C*H*, *J* = 14.9 Hz); 4.44 (d, 1H, C*H*, *J* = 14.9 Hz); 4.87 (s, 2H, C*H*_2_); 6.87 (d, 1H, aryl, *J* = 8.1 Hz); 7.07 (t, 1H, aryl, *J* = 8.2 Hz); 7.34–7.38 (m, 3H, aryl); 7.50–7.54 (m, aryl, 4H); 7.57 (d, 2H, aryl, *J* = 8.1 Hz); 7.81 (d, 1H, aryl, *J* = 7.7 Hz). ^13^C NMR (100 MHz, CDCl_3_) δ: 22.37, 22.44, 25.2, 39.7, 47.8, 54.8, 56.8, 61.2, 117.8, 121.9, 124.0 (q, ^1^ *J*_C–F_= 271.0 Hz), 125.4 (q, ^3^ *J*_C–F_= 3.8 Hz), 125.5, 125.56, 125.60 (q, ^3^ *J*_C–F_= 3.8 Hz), 128.07, 128.13, 129.1, 129.5, 129.6, 129.6 (q, ^2^ *J*_C–F_= 32.4 Hz), 129.7 (q, ^2^ *J*_C–F_= 32.4 Hz), 129.8, 129.9, 130.8, 132.0, 141.7, 142.5, 146.2, 171.0. HR-MS *m*/*z* calcd for C_29_H_28_F_6_N_2_O [(M + H)]^+^: 535.2179; found 535.2157.

##### (*S*)-3-Benzyl-2,3,4,5-tetrahydro-1*H*-benzo­[*e*]­[1,4]­diazepine (**35**)

Synthesized from **26** as a whitish powder in 55% yield using general procedure L. FC in *n*-hexane/ethyl acetate 1/9, *R*_f_: 0.45.

^1^H NMR (400 MHz, CDCl_3_) δ: 2.70–2.80 (m, 3H, C*H* and C*H*_2_); 3.17–3.27 (m, 1H, C*H*); 3.37 (dd, 1H, CH, *J*′ = 2.4 Hz, *J*″ = 12.9 Hz); 3.92 (dd, 2H, C*H*_2_, *J*′ = 14.5 Hz, *J* = 29.0 Hz); 6.75 (d, 1H, aryl, *J* = 7.4 Hz); 6.84 (t, 1H, aryl, *J* = 7.4 Hz); 7.10 (t, 2H, aryl, *J* = 7.5 Hz); 7.25–7.28 (m, 3H, aryl); 7.33–7.37 (m, 2H, aryl). HR-MS *m*/*z* calcd for C_16_H_18_N_2_ [(M + H)]^+^: 239.1543; found 239.1568.

##### (*S*)-3-Isobutyl-2,3,4,5-tetrahydro-1*H*-benzo­[*e*]­[1,4]­diazepine (**36**)

Synthesized from **27** as a whitish powder in 54% yield using the general procedure L. FC in *n*-hexane/ethyl acetate 2/8, *R*_f_: 0.46.

^1^H NMR (400 MHz, CDCl_3_) δ: 0.96 (d, 6H, C*H*_3_, *J* = 6.6 Hz); 1.16–1.22 (m, 1H, C*H*); 1.28–1.36 (m, 1H, C*H*); 1.74–1.81 (m, 1H, C*H*); 2.63 (dd, 1H, CH, *J*′ = 8.6 Hz, *J*″ = 13.0 Hz); 2.93–2.99 (m, 1H, C*H*); 3.36 (dd, 1H, C*H*, *J*′ = 2.6 Hz, *J* = 13.0 Hz); 3.95 (dd, 4H, C*H*_2_, *J*′ = 14.8 Hz, *J* = 29.2 Hz); 6.75 (d, 1H, aryl, *J* = 7.5 Hz); 6.84 (t, 1H, aryl, *J* = 7.3 Hz); 7.09 (t, 2H, aryl, *J* = 7.5 Hz). HR-MS *m*/*z* calcd for C_13_H_20_N_2_ [(M + H)]^+^: 205.1699; found 205.1680.

##### (*S*)-3-Benzyl-4-isobutyl-2,3,4,5-tetrahydro-1*H*-benzo­[*e*]­[1,4]­diazepine (**37**)

Synthesized from **35** as an off-white powder in 42% yield using general procedure A. FC in *n*-hexane/ethyl acetate 8/2, *R*_f_: 0.45. [α]^25^_D_: −18.36 ± 0.23 (c = 0.10, MeOH).

^1^H NMR (400 MHz, (CD_3_)_2_SO) δ: 0.68 (dd, 6H, C*H*_3_, *J*′ = 6.6 Hz, *J*″ = 11.1 Hz); 1.55–1.64 (m, 1H, C*H*); 2.15–2.26 (m, 2H, C*H*_2_); 2.74–2.83 (m, 2H, C*H*_2_); 2.95–2.98 (m, 2H, C*H*_2_); 3.03–3.09 (m, 1H, C*H*); 3.50 (d, 1H, C*H*, *J* = 15.5 Hz); 4.26 (d, 1H, C*H*, *J* = 15.5 Hz); 5.30 (t, 1H, N*H*, *J* = 3.2 Hz); 6.63 (d, 1H, aryl, *J* = 7.2 Hz); 6.68 (d, 1H, aryl, *J* = 7.5 Hz); 6.94 (d, 2H, aryl, *J* = 7.4 Hz); 7.18 (d, 2H, aryl, *J* = 6.8 Hz); 7.23–7.30 (m, 4H, aryl). ^13^C NMR (100 MHz, (CD_3_)_2_SO) δ: 20.9, 21.0, 26.1, 38.4, 47.4, 53.1, 59.8, 65.7, 117.5, 118.8, 126.1, 127.3, 127.3, 127.6, 128.4, 129.7, 130.8, 140.9, 150.9. HR-MS *m*/*z* calcd for C_20_H_26_N_2_ [(M + H)]^+^: 295.2169; found 295.2171.

##### Synthesis of (*S*)-3-Isobutyl-1,4-bis­(4-(trifluoromethyl)­benzyl)-2,3,4,5-tetrahydro-1*H*-benzo­[*e*]­[1,4]­diazepine (**38**)

To a solution of intermediate **36** (0.1 mmol) dissolved in ACN, 4-(trifluoromethyl)­benzyl bromide (2 equiv), K_2_CO_3_ (2 equiv), and a KI (2 equiv) were added. The mixture was heated into a microwave apparatus at 120 °C for 1 h. After the completion of the reaction, the solvent was evaporated under reduced pressure, and the crude product was reconstituted with DCM and washed with a saturated solution of potassium carbonate. The organic layer was dried over anhydrous Na_2_SO_4_ and filtered. The desired compound was purified by flash chromatography using dichloromethane/methanol 9/1 as the eluent (*R*_f_: 0.45) in 57% of yield (off-white powder). [α]^25^_D_: −44.33 ± 0.24 (c = 0.10, MeOH).

^1^H NMR (400 MHz, CDCl_3_) δ: 0.81 (dd, 6H, C*H*_3_, *J*′ = 6.2 Hz, *J*″ = 14.1 Hz); 0.94–1.01 (m, 1H, C*H*); 1.54–1.64 (m, 2H, C*H*_2_); 2.88 (bs, 1H, C*H*); 3.02–3.12 (m, 2H, C*H*_2_); 3.54 (d, 1H, CH, *J* = 7.7 Hz); 3.66 (d, 1H, C*H*, *J* = 8.9 Hz); 3.76 (d, 1H, C*H*, *J* = 8.9 Hz); 4.21 (d, 1H, C*H*, *J* = 8.5 Hz); 4.49 (dd, 2H, C*H2*, *J*′ = 6.2 Hz and *J*″ = 10.4 Hz); 6.88–6.93 (m, 3H, aryl); 7.19–7.23 (m, 1H, aryl); 7.45 (d, 2H, aryl, *J* = 8.1 Hz); 752 (d, 2H, aryl, *J* = 8.2 Hz); 7.62 (dd, 4H, aryl, *J*′ = 4.7 Hz and *J*″ = 9.2)). ^13^C NMR (100 MHz, CDCl_3_) δ: 22.3, 23.0, 24.7, 41.0, 53.0, 54.1, 57.8, 60.5, 116.4, 120.8, 124.2 (q, 1*J*_C–F_= 271.0 Hz), 124.4 (q, ^1^ *J*_C–F_= 271.0 Hz),125.09, 125.13 (q, ^3^ *J*_C–F_= 3.7 Hz), 125.40 (q, ^3^ *J*_C–F_= 3.7 Hz), 125.43, 128.6, 129.0, 129.6 (q, ^2^ *J*_C–F_= 34.1 Hz), 129.7 (q, ^2^ *J*_C–F_= 34.1 Hz), 129.7, 131.3, 143.4, 144.2, 151.6. HR-MS *m*/*z* calcd for C_29_H_30_F_6_N_2_O [(M + H)]^+^: 521.2386; found 521.2378.

##### Methyl Benzyl-*L*-phenylalaninate (**39**)

Synthesized from benzaldehyde and *L*-Phe-OMe as an off-white oil in 72% yield using general procedure A. FC in *n*-hexane/ethyl acetate 7/3, *R*_f_: 0.47.

^1^H NMR (400 MHz, CDCl_3_) δ: 2.99 (d, 2H, C*H*_2_, *J* = 5.2 Hz); 3.58 (t, 1H, CH, *J* = 6.8 Hz); 3.65–3.68 (m, 4H, C*H* and C*H*_3_); 3.84 (d, 1H, CH, *J* = 13.1 Hz); 7.19 (d, 2H, aryl, *J* = 11.1 Hz); 7.24–7.33 (m, 8H, aryl). HR-MS *m*/*z* calcd for C_17_H_19_NO_2_ [(M + H)]^+^: 270.1489; found 270.1466.

##### Methyl *N*-Benzyl-*N*-((*tert*-butoxycarbonyl)-*L*-leucyl)-*L*-phenylalaninate (**40**)

Synthesized from **39** and Boc-*L*-Leu-OH following the general procedure M in 39% yield. FC dichloromethane/acetate 9/1, *R*_f_: 0.48.

^1^H NMR (400 MHz, CD_3_OD) δ: 0.76 (d, 3H, C*H*_3_, *J* = 6.4 Hz); 0.91 (d, 3H, C*H*_3_, *J* = 6.5 Hz); 1.43 (s, 9H, C*H*_3_); 3.22 (dd, 1H, C*H*, *J*′ = 9.7 Hz, *J*″ = 13.8 Hz); 3.35 (dd, 1H, C*H*, *J*′ = 9.6 Hz, *J*″ = 13.6 Hz); 3.59 (s, 3H, C*H*_3_); 3.90 (d, H, C*H, J* = 16.2 Hz); 4.22 (dd, 1H, C*H, J*′ = 5.6 Hz, *J*″ = 9.6 Hz); 4.59 (d, H, C*H, J* = 16.0 Hz); 7.18 (d, 2H, aryl, *J* = 10.8 Hz); 7.27–7.31 (m, 8H, aryl). HR-MS *m*/*z* calcd for C_28_H_38_N_2_O_5_ [(M + H)]^+^: 483.2853; found 483.2884.

##### (3*S*,6*S*)-1,6-Dibenzyl-3-isobutylpiperazine-2,5-dione (**41**)

Final derivative **41** was synthesized from **40** applying the general procedure I, as a white powder in almost quantitative yield. [α]^25^_D_: −41.60 ± 0.50 (c = 0.10, MeOH).

Precipitated from DCM/diethyl ether. ^1^H NMR (400 MHz, CDCl_3_) δ: 0.03–0.04 (m, 1H, C*H*); 0.85 (d, 3H, C*H*_3_, *J* = 6.6 Hz); 0.90 (d, 3H, C*H*_3_, *J* = 6.6 Hz); 1.18–1.25 (m, 1H, C*H*); 1.48–1.57 (m, 1H, C*H*); 3.35 (d, 2H, C*H*_2_, *J* = 4.2 Hz); 3.94–3.98 (m, 1H, C*H*); 4.01 (d, 1H, C*H*, *J* = 14.7 Hz); 4.26 (t, 1H, C*H*, *J* = 4.2 Hz); 5.74 (d, 1H, C*H*, *J* = 14.7 Hz); 7.18 (bs, 1H, N*H*); 7.22–7.24 (m, 2H, aryl); 7.37–7.48 (m, aryl, 8H). ^13^C NMR (100 MHz, CDCl_3_) δ: 20.5, 22.9, 23.9, 36.3, 43.6, 46.8, 53.5, 59.3, 77.4, 127.7, 128.2, 128.5, 128.9, 129.0, 130.3, 135.1, 166.8, 167.1. HR-MS *m*/*z* calcd for C_22_H_26_N_2_O_2_ [(M + Na)]^+^: 373.1886; found 373.1872.

##### *tert*-Butyl (*S*)-(1-((2-Iodophenyl)­amino)-1-oxo-3-phenylpropan-2-yl)­carbamate (**42**)

Intermediate **42** was synthesized starting from Boc-*L*-Phe-OH and 2-iodoaniline according to general procedure M as a yellowish oil (44% yield). FC in *n*-hexane/ethyl acetate 9/1, *R*_f_: 0.40.

^1^H NMR (400 MHz, CDCl_3_) δ: 1.45 (s, 9H, C*H*_3_); 3.21–3.29 (m, 2H, C*H*_2_); 4.59 (bs, 1H, C*H*); 5.01 (bs, 1H, N*H*); 6.87 (t, 1H, aryl, *J* = 7.8 Hz); 7.26–7.39 (m, 6H, aryl); 7.77 (d, 1H, aryl, *J* = 7.9 Hz); 8.01 (bs, 1H, N*H*); 8.29 (d, 1H, aryl, *J* = 8.0 Hz). HR-MS *m*/*z* calcd for C_20_H_23_IN_2_O_3_ [(M + H)]^+^: 467.0826; found 467.0868.

##### *tert*-Butyl (*S*)-(1-((2-Iodophenyl)­amino)-4-methyl-1-oxopentan-2-yl)­carbamate (**43**)

Intermediate **43** was synthesized starting from Boc-*L*-Leu-OH and 2-iodoaniline according to general procedure M as a yellowish oil (37% yield). FC in *n*-hexane/ethyl acetate 8/2, *R*_f_: 0.45.

^1^H NMR (400 MHz, CD_3_OD) δ: 1.01 (t, 9H, C*H*_3_, *J* = 7.8 Hz); 1.45 (s, 9H, C*H*_3_); 1.64–1.69 (m, 3H, C*H* and C*H*_2_); 4.26 (dd, 1H, C*H*, *J*′ = 6.8 Hz, *J*″ = 13.0 Hz); 6.96 (t, 1H, aryl, *J* = 7.8 Hz); 7.39 (t, 1H, aryl, *J* = 7.5 Hz); 7.80 (d, 1H, aryl, *J* = 7.8 Hz); 7.88 (d, 1H, aryl, *J* = 7.9 Hz). HR-MS *m*/*z* calcd for C_17_H_25_IN_2_O_3_ [(M + H)]^+^: 433.0983; found 433.0944.

##### (*S*)-2-Amino-*N*-(2-iodophenyl)-3-phenylpropanamide (**44**)

Intermediate **44** was synthesized according to the general procedure I, starting from intermediate **42**.

White powder (97% yield).Precipitated from DCM/diethyl ether. ^1^H NMR (400 MHz, CD_3_OD) δ: 2.91 (dd, 1H, C*H*, *J*′ = 8.2 Hz, *J*″ = 13.6 Hz); 3.24 (dd, 1H, C*H*, *J*′ = 4.9 Hz, *J*″ = 13.6 Hz); 3.77 (dd, 1H, C*H*, *J*′ = 4.9 Hz, *J*″ = 8.3 Hz); 6.92 (t, 1H, aryl, *J* = 8.9 Hz); 7.22–7.39 (m, 6H, aryl); 7.85 (d, 1H, aryl, *J* = 8.0 Hz); 7.93 (d, 1H, aryl, *J* = 8.1 Hz). HR-MS *m*/*z* calcd for C_15_H_15_IN_2_O [(M + H)]^+^: 367.0302; found 367.0347.

##### (*S*)-2-Amino-*N*-(2-iodophenyl)-4-methylpentanamide (**45**)

Intermediate **45** was synthesized according to general procedure I, starting from intermediate **43**.

White powder (96% yield).^1^H NMR (400 MHz, CD_3_OD) δ: 1.11 (t, 9H, C*H*_3_, *J* = 6.4 Hz); 1.83–1.99 (m, 3H, C*H* and C*H*_2_); 4.12 (dd, 1H, C*H*, *J*′ = 7.3 Hz, *J*″ = 13.0 Hz); 7.07 (t, 1H, aryl, *J* = 9.0 Hz); 7.44 (t, 1H, aryl, *J* = 8.0 Hz); 7.52 (d, 1H, aryl, *J* = 9.5 Hz); 7.95 (d, 1H, aryl, *J* = 8.0 Hz). HR-MS *m*/*z* calcd for C_12_H_17_IN_2_O [(M + H)]^+^: 333.0458; found 333.0488.

##### (*S*)-3-Benzyl-3,4-dihydroquinoxalin-2­(1*H*)-one (**46**)

Intermediate **46** was synthesized starting from **44** according to general procedure N as a yellowish oil in 45% of yield. FC in ethyl acetate/methanol 9.4/0.6, *R*_f_: 0.37.

^1^H NMR (400 MHz, CD_3_OD) δ: 2.92 (dd, 1H, C*H*, *J*′ = 7.2 Hz, *J*″ = 13.3 Hz); 3.09 (dd, 1H, C*H*, *J*′ = 6.6 Hz, *J*″ = 13.3 Hz); 3.70 (t, 1H, C*H*, *J* = 6.8 Hz); 7.10 (t, 1H, aryl, *J* = 7.4 Hz); 7.21–7.32 (m, 6H, aryl); 7.49 (d, 2H, aryl, *J* = 7.6 Hz). HR-MS *m*/*z* calcd for C_15_H_14_N_2_O [(M + H)]^+^: 239.1179; found 239.1148.

##### (*S*)-3-Isobutyl-3,4-dihydroquinoxalin-2­(1*H*)-one (**47**)

Intermediate **47** was synthesized starting from **45** according to general procedure N as a yellowish oil in 32% of yield. FC in ethyl acetate/methanol 9.6/0.4, *R*_f_: 0.39.

^1^H NMR (400 MHz, CD_3_OD) δ: 1.00 (dd, 6H, C*H*_3,_ *J*′ = 6.6, *J*″ = 8.8 Hz); 1.47–1.54 (m, 1H, C*H*); 1.62–1.69 (m, 1H, C*H*); 1.74–1.84 (m, 1H, C*H*); 3.52 (bs, 1H, C*H*); 7.12 (t, 1H, aryl, *J* = 7.4 Hz); 7.33 (t, 1H, aryl, *J* = 8.4 Hz); 7.59 (d, 2H, aryl, *J* = 9.5 Hz). HR-MS *m*/*z* calcd for C_12_H_16_N_2_O [(M + H)]^+^: 205.1335; found 205.1377.

##### (*S*)-3-Benzyl-4-isobutyl-3,4-dihydroquinoxalin-2­(1*H*)-one (**48**)

Final derivative **48** was synthesized from **46** as an off-white powder in 38% yield using general procedure G. FC in dichloromethane/ethyl acetate 9.8/0.2, *R*_f_: 0.42. [α]^25^_D_: −44.53 ± 0.31 (c = 0.10, MeOH).

^1^H NMR (400 MHz, CD_3_OD) δ: 1.07 (dd, 6H, C*H*_3,_ *J*′ = 3.0, *J*″ = 11.9 Hz); 2.02–2.11 (m, 1H, C*H*); 2.80–2.85 (m, 1H, C*H*); 2.93–2.99 (m, 1H, C*H*); 3.20–3.25 (m, 1H, C*H*); 3.36–3.40 (m, 1H, C*H*); 4.11 (bs, 1H, C*H*); 7.14 (t, 1H, aryl, *J* = 7.3 Hz); 7.28–7.38 (m, 8H, aryl); ^13^C NMR (100 MHz, CD_3_OD) δ: 18.8, 19.0, 26.1, 36.5, 53.9, 62.9, 120.1, 124.8, 127.4, 128.5, 128.6, 129.2, 134.0, 136.8, 154.4. HR-MS *m*/*z* calcd for C_19_H_22_N_2_O [(M + H)]^+^: 295.1805; found 295.1835.

##### (*S*)-4-Benzyl-3-isobutyl-3,4-dihydroquinoxalin-2­(1*H*)-one (**49**)

Final derivative **49** was synthesized from **47** as an off-white powder in 44% yield using general procedure G. FC in dichloromethane/ethyl acetate 9.6/0.4, *R*_f_: 0.44. [α]^25^_D_: −8.72 ± 0.56 (c = 0.10, MeOH).

^1^H NMR (400 MHz, CD_3_OD) δ: 0.76 (d, 3H, C*H*_3_, *J* = 6.6 Hz); 0.86 (d, 3H, C*H*_3_, *J* = 6.6 Hz); 1.41–1.46 (m, 2H, C*H*_2_); 1.61–1.69 (m, 1H, C*H*); 3.18 (d, 1H, C*H, J* = 6.6 Hz); 3.58 (d, 1H, C*H, J* = 13.1 Hz); 3.71 (d, 1H, C*H, J* = 13.1 Hz); 7.01 (t, 1H, aryl, *J* = 7.4 Hz); 7.13 (t, 1H, aryl, *J* = 7.0 Hz); 7.19–7.26 (m, 5H, aryl); 7.45 (d, 2H, aryl, *J* = 8.6 Hz). ^13^C NMR (100 MHz, CD_3_OD) δ: 20.9, 22.2, 24.6, 42.8, 52.3, 60.5, 90.6, 123.1, 126.4, 126.9, 128.1, 128.3, 128.6, 138.5, 139.0, 139.4. HR-MS *m*/*z* calcd for C_19_H_22_N_2_O [(M + H)]^+^: 295.1805; found 295.1837.

##### *tert*-Butyl (*S*)-(1-((2-(Hydroxymethyl)­phenyl)­amino)-4-methyl-1-oxopentan-2-yl)­carbamate (**50**)

Synthesized from 2-aminobenzyl alcohol and Boc-*L*-Leu-OH as a whitish oil in 66% yield following general procedure C. FC *n*-hexane/ethyl acetate, 1/1, *R*_f_: 0.45.

^1^H NMR (400 MHz, CD_3_OD) δ: 1.01 (dd, 1H, C*H*, *J*′ = 6.4 Hz; *J*″ = 9.5 Hz); 1.49 (s, 9H, C*H*_3_); 1.61–1.81 (m, 3H, C*H* and C*H*_2_); 4.19 (bs, 1H, C*H*); 4.64 (dd, 1H, C*H*, *J*′ = 13.0 Hz; *J*″ = 16.2 Hz); 7.18 (t, 1H, aryl, *J* = 7.3 Hz); 7.30 (t, 1H, aryl, *J* = 8.6 Hz); 7.35 (d, 1H, aryl, *J* = 7.9 Hz); 7.76 (d, 1H, aryl, *J* = 8.4 Hz). HR-MS *m*/*z* calcd for C_18_H_28_N_2_O_4_ [(M + H)]^+^: 337.2122; found 337.2145.

##### (*S*)-2-Amino-*N*-(2-(hydroxymethyl)­phenyl)-4-methylpentanamide (**51**)

Synthesized from **50** as an off-white powder in 97% yield using general procedure I.

Precipitated from DCM/diethyl ether. ^1^H NMR (400 MHz, CD_3_OD) δ: 1.08 (dd, 1H, C*H*, *J*′ = 6.2 Hz; *J*″ = 7.6 Hz); 1.79–1.91 (m, 3H, C*H* and C*H*_2_); 4.11 (t, 1H, C*H, J* = 6.2 Hz); 4.67 (dd, 1H, C*H*, *J*′ = 13.1 Hz; *J*″ = 16.4 Hz); 7.26 (t, 1H, aryl, *J* = 8.7 Hz); 7.33 (t, 1H, aryl, *J* = 9.4 Hz); 7.45 (d, 1H, aryl, *J* = 8.6 Hz); 7.62 (d, 1H, aryl, *J* = 8.8 Hz). HR-MS *m*/*z* calcd for C_13_H_20_N_2_O_2_ [(M + H)]^+^: 237.1598; found 237.1566.

##### (*S*)-3-Isobutyl-1,3,4,7-tetrahydrobenzo­[*g*]­[1,3,6]­oxadiazonine-2,5-dione (**52**)

Final compound **52** was synthesized from **51** as an off-white powder in 44% yield using general procedure D. FC in dichloromethane/ethyl acetate 9.6/0.4, *R*_f_: 0.44. [α]^25^_D_: −54.96 ± 0.20 (c = 0.10, MeOH).

^1^H NMR (400 MHz, CD_3_OD) δ: 1.03 (dd, 6H, C*H*_3_, *J*′ = 6.4 Hz and *J*″ =14.5 Hz); 0.95 (dd, 6H, C*H*_3_, *J*′ = 6.5 Hz and *J*″ = 11.8 Hz); 1.71–1.79 (m, 1H, C*H*); 1.82–1.86 (m, 2H, C*H*_2_); 4.59–4.67 (m, 3H, C*H* and *CH*_2_); 7.21 (t, 1H, aryl, *J* = 7.4 Hz); 7.31 (t, 1H, aryl, *J* = 6.3 Hz); 7.39 (d, 1H, aryl, *J* = 7.5 Hz); 7.65 (d, 1H, aryl, *J* = 7.2 Hz). ^13^C NMR (100 MHz, CD_3_OD) δ: 20.4, 21.9, 24.7, 39.8, 53.0, 61.5, 123.7, 125.4, 127.6, 127.7, 133.7, 135.3, 170.5. HR-MS *m*/*z* calcd for C_14_H_18_N_2_O_3_ [(M – H)]^−^: 261.1245; found 261.1235.

##### Synthesis of *tert*-Butyl (2-(Hydroxymethyl)­phenyl)­carbamate (**53**)

To a solution of 2-aminobenzyl alcohol (1 mmol) in THF, di-*tert*-butyl dicarbonate (1.2 equiv) and DIPEA (1.2 equiv) were added. The mixture was heated under microwave irradiation at 120 °C for 1 h. The solvent was evaporated under vacuum; the residue was reconstituted with DCM and washed with a saturated aqueous solution of sodium bicarbonate (3 × 50 mL). The combined organic layers were dried over Na_2_SO_4_, filtered, and concentrated under vacuum. The crude product was purified by flash chromatography using *n*-hexane/ethyl acetate 8/2 as the mobile phase (*R*_f_: 0.45) affording a pale-yellow oil in 88% of yield.

^1^H NMR (400 MHz, CDCl_3_) δ: 1.57 (s, 9H, C*H*_3_); 4.42 (s, 2H, C*H*_2_); 6.46 (bs, 1H, N*H*); 7.06 (t, 1H, aryl, *J* = 8.6 Hz); 7.28–7.34 (m, 2H, aryl); 7.78 (d, 1H, aryl, *J* = 8.0 Hz). HR-MS *m*/*z* calcd for C_12_H_17_NO_3_ [(M + H)]^+^: 224.1281; found 224.1299.

##### Synthesis of *tert*-Butyl (2-(Iodomethyl)­phenyl)­carbamate (**54**)

To a solution of triphenylphosphine (1.2 equiv) and imidazole (1.2 equiv) in DCM dry in a round-bottom flask under nitrogen stream, iodine (1.2 equiv) was added, and the mixture was stirred at room temperature for 5 min. Then, a solution of alcoholic intermediate **53** (0.1 mmol) in DCM dry was added. After complete conversion of the alcohol intermediate (monitored by TLC), the reaction mixture was quenched with an aqueous solution of sodium thiosulfate. The organic layer was separated, dried over Na_2_SO_4_, filtered, and concentrated under vacuum. The residue was purified by flash chromatography using a mixture of *n*-hexane/ethyl acetate 9.5/5 (*R*_f_: 0.45) affording a whitish powder in 90% of yield.

^1^H NMR (400 MHz, CDCl_3_) δ: 1.58 (s, 9H, C*H*_3_); 4.22 (s, 2H, C*H*_2_); 7.24 (t, 1H, aryl, *J* = 8.6 Hz); 7.24–7.29 (m, 2H, aryl); 7.82 (d, 1H, aryl, *J* = 8.0 Hz). HR-MS *m*/*z* calcd for C_12_H_16_INO_2_ [(M + H)]^+^: 334.0298; found 334.0292.

##### Methyl (2-((*tert*-Butoxycarbonyl)­amino)­benzyl)-*L*-leucinate (**55**)

Synthesized from **54** and *L*-Leu-OMe whitish oil in 55% yield using general procedure O. FC in *n*-hexane/ethyl acetate 7/3, *R*_f_: 0.44.

^1^H NMR (400 MHz, CD_3_OD) δ: 0.96 (d, 3H, C*H*_3_, *J* = 6.5 Hz) 1.01 (d, 3H, C*H*_3_, *J* = 6.4 Hz); 1.58 (s, 9H, C*H*_3_); 1.62–1.72 (m, 1H, C*H*); 1.76–1.92 (m, 1H, C*H*_2_); 3.57 (t, 1H, C*H, J* = 7.0 Hz); 3.76 (s, 3H, C*H*_3_); 4.08 (d, 1H, C*H*, *J* = 12.6 Hz); 4.22 (d, 1H, C*H*, *J* = 12.6 Hz); 7.30–7.37 (m, 2H, aryl); 7.44 (t, 1H, aryl, *J* = 6.5 Hz); 7.56 (d, 1H, aryl, *J* = 6.3 Hz). HR-MS *m*/*z* calcd for C_19_H_30_N_2_O_4_ [(M + H)]^+^: 351.2278; found 351.2287.

##### Methyl (2-((*tert*-Butoxycarbonyl)­amino)­benzyl)-*L*-phenylalaninate (**56**)

Obtained from **54** and *L*-Phe-OMe as an off-white oil in 68% yield using general procedure O. FC in *n*-hexane/ethyl acetate 7/3, *R*_f_: 0.45.

^1^H NMR (400 MHz, CDCl_3_) δ: 1.57 (s, 9H, C*H*_3_); 3.01 (t, 2H, C*H*_2_, *J* = 6.9 Hz); 3.55 (t, 1H, C*H, J* = 6.2 Hz); 3.63 (d, 1H, C*H*, *J* = 12.9 Hz); 3.75 (s, 3H, C*H*_3_); 3.92 (d, 1H, C*H*, *J* = 12.9 Hz); 7.93 (t, 1H, aryl, *J* = 7.4 Hz); 7.02 (d, 1H, aryl, *J* = 6.2 Hz); 7.14–7.16 (m, 2H, aryl); 7.25–7.32 (m, 4H, aryl); 8.00 (d, 1H, aryl, *J* = 8.0 Hz). HR-MS *m*/*z* calcd for C_22_H_28_N_2_O_4_ [(M + H)]^+^: 385.2122; found 385.2137.

##### (2-((*tert*-Butoxycarbonyl)­amino)­benzyl)-*L*-leucine (**57**)

Synthesized from **55** as an off-white powder in 94% yield using general procedure B.

^1^H NMR (400 MHz, CD_3_OD) δ: 0.98 (d, 3H, C*H*_3_, *J* = 6.5 Hz); 1.03 (d, 3H, C*H*_3_, *J* = 6.4 Hz); 1.57 (s, 9H, C*H*_3_), 1.65–1.76 (m, 1H, C*H*); 1.80–1.95 (m, 1H, C*H*_2_); 3.63 (t, 1H, C*H, J* = 7.0 Hz); 4.11 (d, 1H, C*H*, *J* = 12.4 Hz); 4.24 (d, 1H, C*H*, *J* = 12.4 Hz); 7.31–7.36 (m, 2H, aryl); 7.44 (t, 1H, aryl, *J* = 6.5 Hz); 7.60 (d, 1H, aryl, *J* = 6.5 Hz). HR-MS *m*/*z* calcd for C_18_H_28_N_2_O_4_ [(M + H)]^+^: 337.2122; found 337.2148.

##### (2-((*tert*-Butoxycarbonyl)­amino)­benzyl)-*L*-phenylalanine (**58**)

Synthesized from **56** as an off-white powder in 95% yield using general procedure B.

^1^H NMR (400 MHz, CDCl_3_) δ: 1.43 (s, 9H, C*H*_3_); 3.12 (dd, 1H, C*H, J*′ = 8.7 Hz and *J*″ = 14.5 Hz); 3.36 (dd, 1H, C*H, J*′ = 8.7 Hz and *J*″ = 14.5 Hz); 3.81 (dd, 1H, C*H, J*′ = 4.7 Hz and *J*″ = 8.7 Hz); 3.95 (d, 1H, C*H, J* = 12.7 Hz); 4.23 (d, 1H, C*H, J* = 12.7 Hz); 7.23 (d, 1H, aryl, *J* = 7.4 Hz); 7.28–7.36 (m, 5H, aryl); 7.42 (t, 1H, aryl, *J* = 7.6 Hz); 7.51 (d, 1H, aryl, *J* = 7.5 Hz). HR-MS *m*/*z* calcd for C_21_H_26_N_2_O_4_ [(M + H)]^+^: 371.1965; found 371.1988.

##### Benzyl (2-((*tert*-Butoxycarbonyl)­amino)­benzyl)-*L*-leucinate (**59**)

Synthesized from **57** and benzyl alcohol as an off-white oil in 52% yield using general procedure F. FC in *n*-hexane/ethyl acetate 8/2, *R*_f_: 0.47.

^1^H NMR (400 MHz, CD_3_OD) δ: 0.89 (d, 3H, C*H*_3_, *J* = 6.6 Hz); 0.95 (d, 3H, C*H*_3_, *J* = 6.4 Hz); 1.48–1.52 (m, 2H, C*H*_2_); 1.55 (s, 9H, C*H*_3_); 1.88–1.95 (m, 1H, C*H*); 3.35 (dd, 1H, C*H, J*′ = 5.6 Hz and *J*″ = 8.8 Hz); 3.64 (d, 1H, C*H*, *J* = 12.5 Hz); 3.86 (d, 1H, C*H*, *J* = 12.5 Hz); 5.20 (s, 2H, C*H*_2_); 6.94 (t, 1H, aryl, *J* = 7.4 Hz); 7.28 (t, 1H, aryl, *J* = 7.4 Hz); 7.38–7.44 (m, 6H, aryl); 8.04 (d, 1H, aryl, *J* = 8.0 Hz). HR-MS *m*/*z* calcd for C_25_H_34_N_2_O_4_ [(M + H)]^+^: 427.2591; found 427.2577.

##### Isobutyl (2-((*tert*-Butoxycarbonyl)­amino)­benzyl)-*L*-phenylalaninate (**60**)

Synthesized from **58** and 2-methylpropan-1-ol as an off-white powder in 48% yield using general procedure F. FC in *n*-hexane/ethyl acetate 8/2, *R*_f_: 0.45.

^1^H NMR (400 MHz, CDCl_3_) δ: 0.91 (d, 6H, C*H*_3_, *J* = 6.5 Hz); 1.57 (s, 9H, C*H*_3_); 1.84–1.94 (m, 3H, C*H* and C*H*_2_); 3.12 (dd, 1H, C*H, J*′ = 6.2 Hz and *J*″ = 8.9 Hz); 3.44 (dd, 1H, C*H, J*′ = 6.2 Hz and *J*″ = 8.9 Hz); 3.80 (dd, 1H, C*H, J*′ = 6.2 Hz and *J*″ = 8.9 Hz); 3.87 (d, 1H, C*H*, *J* = 12.2 Hz); 3.95 (d, 1H, C*H*, *J* = 12.2 Hz); 6.93 (t, 1H, aryl, *J* = 7.4 Hz); 7.01 (d, 1H, aryl, *J* = 7.4 Hz); 7.15 (d, 2H, aryl, *J* = 8.0 Hz); 7.23–7.30 (m, 4H, aryl); 8.00 (d, 1H, aryl, *J* = 8.1 Hz). HR-MS *m*/*z* calcd for C_25_H_34_N_2_O_4_ [(M + H)]^+^: 427.2591; found 427.2544.

##### Benzyl (2-Aminobenzyl)-*L*-leucinate (**61**)

Synthesized from **59** as an off-white powder in 94% yield using general procedure I.

Precipitated from DCM/diethyl ether. ^1^H NMR (400 MHz, CD_3_OD) δ: 0.78 (d, 6H, C*H*_3_, *J* = 6.1 Hz); 1.28–1.35 (m, 2H, C*H*_2_); 1.69–1.78 (m, 1H, C*H*); 3.39 (dd, 1H, C*H, J*′ = 6.2 Hz and *J*″ = 8.9 Hz); 3.84 (dd, 1H, C*H, J*′ = 6.6 Hz and *J*″ = 14.0 Hz); 4.29 (d, 2H, C*H*_2_, *J* = 6.4 Hz); 6.94 (t, 1H, aryl, *J* = 8.4 Hz); 7.00 (t, 1H, aryl, *J* = 8.2 Hz); 7.25–7.39 (m, 7H, aryl); 8.04 (d, 1H, aryl, *J* = 8.0 Hz). HR-MS *m*/*z* calcd for C_20_H_26_N_2_O_2_ [(M + H)]^+^: 327.2067; found 327.2088.

##### Isobutyl (2-Aminobenzyl)-*L*-phenylalaninate (**62**)

Synthesized from **60** as an off-white powder in 96% yield using the general procedure I.

Precipitated from DCM/diethyl ether. ^1^H NMR (400 MHz, CD_3_OD) δ: 0.77 (d, 6H, C*H*_3_, *J* = 6.8 Hz); 1.26–1.35 (m, 2H, C*H*_2_); 1.67–1.76 (m, 1H, C*H*); 3.16 (dd, 1H, C*H, J*′ = 9.1 Hz and *J*″ = 13.7 Hz); 3.42 (dd, 1H, C*H, J*′ = 5.7 Hz and *J*″ = 13.8 Hz); 4.29 (d, 2H, C*H*_2_, *J* = 4.8 Hz); 4.35 (dd, 1H, C*H, J*′ = 5.7 Hz and *J*″ = 9.1 Hz); 6.94 (t, 1H, aryl, *J* = 7.6 Hz); 7.00 (d, 1H, aryl, *J* = 8.2 Hz); 7.25–7.38 (m, 7H, aryl). HR-MS *m*/*z* calcd for C_20_H_26_N_2_O_2_ [(M + H)]^+^: 327.2067; found 327.2088.

##### Benzyl (*S*)-4-Methyl-2-(2-oxo-1,4-dihydroquinazolin-3­(2*H*)-yl)­pentanoate (**63**)

Final derivative **63** was synthesized from **61** as an off-white oil in 55% yield using the general procedure P. FC in dichloromethane/ethyl acetate 9.6/0.4, *R*_f_: 0.44. [α]^25^_D_: + 6.05 ± 0.22 (c = 0.10, MeOH).

^1^H NMR (400 MHz, CD_3_OD) δ: 0.76 (d, 3H, C*H*_3_, *J* = 6.6 Hz); 0.95 (dd, 6H, C*H*_3_, *J*′ = 6.5 Hz and *J*″ = 11.8 Hz); 1.52–1.61 (m, 1H, C*H*); 1.74–1.82 (m, 1H, C*H*); 1.91–1.98 (m, 1H, C*H*); 4.42 (dd, 2H, C*H*_3_, *J*′ = 14.4 Hz and *J*″ = 16.1 Hz); 5.08 (dd, 1H, C*H, J*′ = 4.9 Hz and *J*″ = 11.1 Hz); 5.19 (dd, 2H, C*H*_2_, *J*′ = 12.3 Hz and *J*″ = 20.6 Hz); 6.82 (d, 1H, aryl, *J* = 7.4 Hz); 6.95 (t, 1H, aryl, *J* = 6.4 Hz); 7.08 (d, 1H, aryl, *J* = 7.5 Hz);); 7.18 (t, 1H, aryl, *J* = 8.0 Hz); 7.29–7.35 (m, 5H, aryl). ^13^C NMR (100 MHz, CD_3_OD) δ: 20.3, 22.1, 24.7, 36.4, 55.0, 66.5, 113.3, 118.0, 121.9, 125.2, 127.7, 127.8, 127.9, 128.2, 135.9, 136.7, 155.8, 171.7. HR-MS *m*/*z* calcd for C_21_H_24_N_2_O_3_ [(M + Na)]^+^: 375.1679; found 375.1667.

##### Isobutyl (*S*)-2-(2-Oxo-1,4-dihydroquinazolin-3­(2*H*)-yl)-3-phenylpropanoate (**64**)

Synthesized from **62** whitish oil in 60% yield using general procedure P. FC in dichloromethane/ethyl acetate 9.8/0.2, *R*_f_: 0.42. [α]^25^_D_: −15.88 ± 0.42 (c = 0.10, MeOH).

^1^H NMR (400 MHz, CD_3_OD) δ: 0.80 (d, 6H, C*H*_3_, *J* = 6.7 Hz); 1.77–1.87 (m, H, C*H*); 3.14 (dd, 1H, C*H, J*′ = 10.8 Hz and *J*″ = 14.3 Hz); 3.28 (dd, 1H, C*H, J*′ = 5.4 Hz and *J*″ = 14.4 Hz); 3.80–3.88 (m, 2H, C*H*_2_); 4.10 (d, 1H, C*H*_2_, *J* = 14.1 Hz); 4.40 (d, 1H, C*H*_2_, *J* = 14.1 Hz); 4.82 (dd, 1H, C*H, J*′ = 5.4 Hz and *J*″ = 10.8 Hz); 6.63 (d, 1H, aryl, *J* = 7.9 Hz); 6.78 (t, 1H, aryl, *J* = 7.4 Hz); 6.86 (d, 1H, aryl, *J* = 6.8 Hz); 6.99–7.05 (m, 2H, aryl); 7.08–7.15 (m, 4H, aryl). ^13^C NMR (100 MHz, CD_3_OD) δ: 18.0, 27.5, 34.0, 59.7, 71.0, 113.2, 117.7, 121.7, 125.0, 126.2, 127.7, 128.1, 128.6, 136.6, 137.5. HR-MS *m*/*z* calcd for C_21_H_24_N_2_O_3_ [(M + Na)]^+^: 375.1679; found 375.1674.

### Inverse Virtual Screening

All the compounds used were built using the Maestro 2D Sketcher tool and prepared for the IVS with LigPrep.^98^ The IVS experiments were carried out on an in-house panel containing the structures of proteins involved in cancer. The panel was built using PDB Cl.O.E.,^99^ an automatic workflow that generates the input macromolecule files required for molecular docking. The final target panel contained ∼6.7 × 10^3^ entries corresponding to the PDB list reported in Table S5. For each PDB file, more than one protein entry can be generated (see reference 99 for further details). The panel build in this way was subjected to molecular docking experiments using Glide^100–103^ in XP mode and enhanced sampling, keeping 10,000 poses for each ligand in the initial evaluation step. The top 800 poses were selected for energy reduction, considering a van der Waals radii scaling factor of 0.8 and a partial charge cutoff of 0.15. Following this procedure, the top 50 poses for each ligand were retained. The same procedure was carried out for ten decoy molecules chosen for **31** and **63**, which were used to normalize the binding affinities calculated for the query compounds. In detail, the decoy molecules have the same properties as the query compounds (e.g., MW, hydrogen bond donor, and acceptor) but different core structures. Then, for each target, the calculated binding affinities are divided by the average binding affinity of the decoys, resulting in an adimensional number called “V value”. In this way, false-positive results are limited. After the normalization step, a series of filters were applied to narrow down the list of putative targets (Figure 2). In the first stage of analysis, only targets with a V value greater than 1.0 and for which at least six decoys gave a predicted binding affinity were considered. This criterion was applied because not all decoys may interact effectively with each target and, as a result, the average binding affinities used to calculate the V value may not always include data from ten distinct decoys. Subsequently, we selected only those protein–ligand complexes that exhibited a favorable predicted binding affinity, specifically lower than −6.2 kcal/mol. Finally, after significantly reducing the number of potential targets compared to the initial data set, a visual inspection of each protein–ligand complex was performed to identify interactions between the investigated compounds and key amino acids within the receptor’s binding cavity. The final list of selected targets for compounds **31** and **63** is provided in Tables 2 and 3.

### Molecular Docking on TRPA1

The molecular structure of TRPA1 (PDB ID: 6X2J)^104^ was retrieved from the Protein Data Bank. The structure was prepared using the Protein Preparation Wizard tool of the Schrödinger Suite to fix common structure mistakes like atom partial charges, bond orders, etc. The molecular docking was carried out with Glide^100–103^ following the same parameters applied in the Inverse Virtual Screening procedure. The docking was performed in XP mode and enhanced sampling, initially generating 10,000 poses per ligand. From these, the top 800 poses were submitted to further refinement through energy minimization, applying a van der Waals radii scaling factor of 0.8 and a partial charge cutoff of 0.15.

### Proteomics Analysis

Protein pellet digestion and cleanup was performed as reported previously.^105^ Proteomic analysis was performed by nLC-HRMS using an Ultimate 3000 nanoLC (Thermo Fisher Scientific, Bremen, Germany) coupled to an Orbitrap Lumos tribrid mass spectrometer (Thermo Fisher Scientific) with an Easy nano electrospray ion source (Thermo Fisher Scientific). Peptides were trapped for 1 min in a PepMap trap-Cartridge, 100 Å, 5 μm, 0.3 × 5 mm (Thermo Fisher), and separated onto a C18-reversed phase column (250 mm × 75 μm I.D, 2.0 μm, 100Å, Thermo). Mobile phases were A): 0.1% HCOOH in water v/v; B): 0.1% HCOOH in ACN/water v/v 80/20. Peptides were separated using a linear gradient of 90 min. HRMS analysis was performed in data-dependent acquisition (DDA), with MS1 range 400–1500 *m*/*z*, HCD fragmentation was used with normalized collision energy setting 27. Resolution was set at 120.000 for MS1 and 15.000 for MS/MS. Single charge and unassigned charge peptides were excluded. Quadrupole isolation was set to 3 Da. Maximum ion injection times for MS (OT) and the MS/MS (OT) scans were set to auto and 50 ms respectively, and ACG values were set to standard. Dynamic exclusion: 30 s. For data processing, raw MS data were analyzed using Proteome Discoverer v 2.5 (Thermo Fisher). The following parameters were used: enzyme trypsin, missed cleavages max 1, mass accuracy tolerance 10 ppm and 0.6 Da for precursors and fragments, respectively. Sequest search and Percolator algorithm were used. Carbamidomethylcysteine was used as fixed modification while methionine oxidation as variable. Proteins were considered identified with at least one unique peptide, using a false discovery rate (FDR) threshold of 0.1.

### Target Prediction with Publicly Available Tools

Two target prediction tools that are available as web servers were used, STP (http://www.swisstargetprediction.ch/),^106–108^ and SP (http://prediction.charite.de).^107,109^ SP functions as a 2D similarity ensemble approach using ECFP4 fingerprints. Bioactivities that were used to build reference target sets in SP were derived from ChEMBL,^110^ Binding DB,^111^ and SuperTarget.^112,113^ SP is maintained by the structural bioinformatics group of the Charité (University Medicine Berlin in Germany). In contrast, STP uses a hybrid approach combining 2D and 3D ligand-based similarity. It calculates 2D similarity via Tanimoto scores using FP2 fingerprints and 3D similarity as the Manhattan distance between electroshape vectors.^114^ A logistic regression model then classifies the input ligand based on both 2D and 3D similarity. STP also draws bioactivity data from ChEMBL and was developed by the Swiss Institute of Bioinformatics (SIB).

### Cell Culture

The human hepatoma HEPG2 cell line was obtained from American Type Culture Collection (ATCC, Rockville, MD, USA). Cells were grown in Eagle’s Minimum Essential Medium (EMEM) supplemented with 10% (v/v) fetal bovine serum (FBS), 2 mM l-glutamine, 1% (v/v) nonessential amino acids, 100 U/mL penicillin, and 0.1 mg/mL streptomycin.

The human neuroblastoma SHSY5Y, melanoma A375, human breast cancer MCF7, nonsmall lung cancer A549, and human epidermal keratinocyte HaCaT cell lines were obtained from ATCC and were grown in Dulbecco’s Modified Eagle Medium (DMEM) supplemented with 10% (v/v) FBS, 2 mM l-glutamine, 100 U/mL penicillin, and 0.1 mg/mL streptomycin.

Cells were routinely grown in culture dishes (Corning, Corning, New York) in an environment containing 5% CO_2_ at 37 °C and passaged at confluence using a solution of 0.025% trypsin and 0.01% EDTA. They were subcultured every 2 days with growth and viability monitored using phase-contrast microscopy and trypan blue staining.^115^ In each experiment, cells were placed in a fresh medium, cultured in the presence of synthesized compounds, and followed for further analyses. All experiments were performed in triplicate.

### Cell Viability Assays

Cell viability was established by measuring mitochondrial metabolic activity with MTT. In brief, HEPG2 (1 × 10^4^ cells/well), A375 (2 × 10^3^ cells/well), HaCaT (3 × 10^3^ cells/well), A549 (3 × 10^3^ cells/well), SHSY5Y (2 × 10^4^ cells/well), and MCF 7 (5 × 10^3^ cells/well) were plated into 96-well plates for 24 h, then the compounds (0.01–40 μM) were added. After 24h, cells were replaced with a fresh medium containing 0.5 mg/mL MTT. Cells were incubated 1–3 h at 37 °C, then the medium was discarded, and formazan blue crystals in the cells were dissolved in 100 μL per well of a solution containing isopropanol/HCl 0.1 M. The absorbance was measured at 570 nm using a microplate reader (Multiskan Go, Thermo Scientific, Waltham, MA, USA). Cell viability was expressed as a percentage relative to the untreated cells cultured in medium with 0.1% DMSO and set to 100%, whereas 10% DMSO was used as positive control and set to 0% of viability. The EC_50_ values were calculated using GraphPad Prism 8.0 software by nonlinear regression of dose–response inhibition.

### RNA Data Acquisition and Analysis

RNA expression data were obtained from “The Human Protein Atlas” (https://www.proteinatlas.org/). This resource provides an extensive collection of gene expression data acquired through RNA sequencing (RNA-seq) across various human tissues and cell types. Specifically, the data were used to compare expression levels with those found in the cell lines used in the MTT assays. The analysis focused on correlating the RNA expression profiles from “The Human Protein Atlas” with the expression observed in healthy HaCaT and A375 melanoma cells, providing a reference framework for interpreting the MTT assay results.

### Measurement of Intracellular ROS

The intracellular ROS generation was detected using 10 μM DCFH-DA. This compound is oxidized by ROS to fluorescent carboxydichlorofluorescein (DCF) inside the cells. A375 cells were seeded (5 × 10^3^ cells/well) in a volume of 100 μL per well in black 96-well OptiPlate (PerkinElmer, USA), allowing them to adhere for 24 h. After, the cells were incubated for 2, 4, and 8 h with compounds (10, 5, and 2.5 μM). Subsequently, the cells were washed twice with PBS and a staining solution containing DCFH-DA in serum-free medium without phenol red was added for 30 min at 37 °C in the dark. The stained cells were washed, and the fluorescence signals (excitation/emission 485 nm/535 nm) were read using a PerkinElmer EnSpire multimode plate reader and expressed as DCFH fluorescence intensity.^116^

### Determination of Protein Misfolding

Protein misfolding was analyzed using ThT, (Sigma-Aldrich, St. Louis, MO, USA) staining. A375 cells (5 × 10^3^ cells/well) were grown in 96-well plates and allowed to adhere for 24 h. Later the medium was replaced, and cells were treated with compounds (**31**: 10, 5, and 2.5 μM; **63**: 20, 10, and 5 μM), for 24 h. After treatments, the culture medium was replaced, cells washed twice with PBS and then suspended in 100 μL ThT (20 μM). After washing, a staining solution containing 20 μM ThT in PBS was added for 30 min at 37 °C in the dark. The stained cells were washed, and representative images were acquired using ZOE Fluorescent Cell Imaging System (Magnification, 20×). Quantitative analyses were performed reading the fluorescence signals (excitation/emission 450 nm/482 nm) using a PerkinElmer EnSpire multimode plate reader (PerkinElmer, Waltham, MA, USA).^117^

### Annexin V-FITC Staining

Apoptosis of the cells was assessed using the Annexin V-FITC/PI reagents. A375 cells (3 × 10^4^ cells/well) were seeded into 24-well plates and incubated for 72 h with compounds (5, 2.5, and 1.25 μM). After treatment, the collected cells were resuspended in a 100 μL assay buffer, then 5 μL Annexin V-FITC and 1 μL PI reagents were added following an incubation for 20 min at room temperature according to the manufacturer’s protocol (Dead Cell Apoptosis Kits with Annexin V for Flow Cytometry, Thermo Fisher Scientific). Cells were analyzed with a Becton Dickinson FACScan flow cytometer using the Cell Quest software, version 4 (Franklin Lakes, NJ, USA).^118^

### Lipid and Metabolite Extraction

A375 cells were seeded (2 × 10^6^ cells) in 100 mm culture dishes. After treatment with the test compounds (2.5 μM) for 72 h, the conditioned medium from the cells was collected after centrifugation (1000 *g*, 5 min) to remove cell debris. The cells were washed twice and collected with 500 μL of ice-cold MeOH/H_2_O (80:20 v/v). Then the cells were centrifuged at 1000 *g* for 10 min to isolate the cell pellet.

226 μL of ice-cold MeOH containing a blend of deuterated standards was spiked to cell pellets, kept at–30 °C for 1 min and then transferred to an ultrasonic bath for 10 min. Following the addition of 750 μL of ice-cold methyl *tert*-butyl ether (MTBE), cells were incubated in a Thermomixer (Eppendorf) for 1 h at 4 °C and 550 rpm. 188 μL of water was added to the samples, and after centrifugation, the upper (for lipids) and lower (for metabolites) layers were collected and evaporated using a SpeedVac (Savant, Thermo Scientific, Milan, Italy). Dried extracts were dissolved in 150 μL of CHCl_3_/MeOH/IPA (1/2/4) and 50 μL of H_2_O/MeOH 90:10 (v/v %), respectively, for lipidomics and metabolomics analyses.

### Lipidomics Profiling

Lipidome has been profiled using a Thermo Ultimate UHPLC system (Thermo Scientific, Bremen, Germany) connected online to a TimsTOF Pro Quadrupole Time-of-Flight (Q-TOF) mass spectrometer (Bruker Daltonics, Bremen, Germany) equipped with an Apollo II electrospray ionization (ESI) source.

Lipids separation was performed using a Waters Acquity UPLC CSH C18 column (50 × 2.1 mm; 1.7 μm, 130 Å) paired with a VanGuard CSH precolumn (5.0 × 2.1 mm; 1.7 μm, 130 Å) (Waters, Milford, MA, USA). The column oven temperature was maintained at 65 °C, with a flow rate of 0.55 mL min^–1^. The mobile phases were composed of: (A) ACN/H_2_O 60:40 (v/v %) and (B) IPA/ACN 90:10 (v/v %), both buffered with 10 mM HCOONH_4_ and 0.1% HCOOH (v/v %) for the positive ionization mode and with 10 mM CH_3_COONH_4_ and 0.1% CH_3_COOH (v/v %) for the negative ionization mode. The following gradient was applied: 0 min, 40% B; 0.4 min, 43% B; 0.425 min, 50% B; 0.9 min, 57% B; 2.0 min, 70% B; 2.950 min, 99% B; 3.3 min, 99% B; 3.31 min, 40% B, followed by a 0.7 min re-equilibration of the column.

At the start of each run, mass and mobility accuracy were recalibrated by injecting a mixture (1:1 v/v %) of 10 mM sodium formate calibrant solution and ESI-L Low Concentration Tuning Mix. Data-dependent parallel accumulation serial fragmentation (DDA-PASEF) acquisition mode was employed. Both positive and negative ESI ionization were used in separate runs, with each sample analyzed in duplicate. The injection volume was set at 2 and 3 μL, respectively, for positive and negative ionization mode. ESI source parameters were set as follows: nebulizer gas (N_2_) pressure at 4.0 bar, dry gas (N_2_) flow rate at 10 L/min, and dry temperature at 280 °C. Mass spectra were collected over the *m*/*z* range of 50–1500, with accumulation and ramp times of 100 ms each. Ion mobility was scanned from 0.55 to 1.80 Vs/cm^2^. Precursors were isolated within ± 2 *m*/*z* and fragmented using ion mobility-dependent collision energies MS/MS stepping mode: 1/K0 (Vs/cm^2^) 0.55–1.8; CE [eV] #1:20–40 and CE [eV] #2:35–50. The total acquisition cycle lasted 0.32 s, consisting of one PASEF ramp. Exclusion time was set to 0.1 min, and ion charge control (ICC) was configured to 7.5 million.

Data alignment, filtering, and annotation were carried out using MetaboScape 2023b (Bruker) that uses a feature-detection algorithm (T-Rex 4D). The feature detection threshold was set to 250 counts for both positive and negative ionization modes, with a minimum of 75 data points in the 4D-TIMS space. Lipidomics data were deconvoluted in positive mode using [M + H]^+^, [M + Na]^+^, [M + K]^+^, [M + H – H_2_O]^+^ and [M + NH_4_]^+^, ions, and in negative mode using [M – H] ^–^ [M + Cl] ^–^, [M + CH_3_COO]^−^ and [M – H_2_O] ^–^ ions. Lipids were annotated through a rule-based approach and the LipidBlast spectral library in MS-DIAL. Molecular formulas were assigned using SmartFormula (SF). Compound annotation has been performed setting the following parameters: mass accuracy (narrow: 2 ppm, wide: 10 ppm), mSigma (narrow: 30, wide: 250), MS/MS score (narrow: 800, wide: 150), and Collision Cross-Section (CCS) % (narrow: 2, wide: 3.5). CCS values were compared with predictions from the CCSbase platform.

After the annotation process, all spectra were manually curated and examined. Lipids missing in more than 75% of real samples and 50% of QC samples were excluded. Additionally, lipids with a Coefficient of Variation (CV) above 30% among QCs were discarded. Lipid intensities were normalized using the corresponding deuterated internal standard. The data set was then log-transformed and autoscaled for multivariate data analysis.

### Metabolomics Profiling

Metabolome analyses were performed on a Thermo Vanquish Flex UHPLC (2.1 mm I.D setup) coupled online to a hybrid quadrupole Orbitrap Exploris 120 mass spectrometer (Thermo Fisher Scientific, Bremen, Germany) equipped with a heated electrospray ionization probe (HESI II). The MS was daily calibrated by Pierce FlexMix calibration solutions in both polarities. Metabolites separation was performed with an Acquity UPLC HSS T3 (150 × 2.1 mm; 1.8 μm, 100Å) protected with a VanGuard HSS T3 precolumn (5.0 × 2.1 mm; 1.8 μm, 100Å) (Waters, Milford, MA, U.S.A). The column temperature was set at 45 °C, a flow rate of 0.4 mL/min was used, the mobile phase consisting of (A): H_2_O + 0.1% HCOOH and (B): ACN + 0.1% HCOOH was used for positive ionization, while that with (A): H_2_O + 1 mM NH_4_F and (B): ACN was used for negative ionization mode. The following gradient has been used: 0–1 min, 0% B; 1.5 min, 25% B; 6 min, 70% B; 8 min, 80% B, 9 min, 98%; 10 min 98% B and 4 min for column re-equilibration.

The ESI source parameters were sheath gas pressure, 40 a.u; aux gas flow, 15 au; sweep gas flow, 0 au Spray voltages were set to 3.3 kV and 3.0 kV for ESI (+) and ESI (−) respectively; the Ion Transfer Tube (ITT) and Vaporizer temperature were set to 280 and 300 °C. MS data acquisition was performed in full scan-data dependent acquisition (FS-DDA) in the *m*/*z* 70–800, MS1 resolution was set to 60000, the AGC target was set to auto with a maximum injection time at 100 ms. MS/MS was employed with an isolation window of 1–5 Da, dynamic exclusion of 10s, resolution was set to 15.000, and HCD was used with normalized collision energies of 20, 40, and 60. The instrument was externally calibrated daily with FlexMix solution (Thermo Fisher) while at the beginning of every LC run the internal calibrant was injected (IC run start mode).

FreeStyle (Thermo Fisher Scientific) was used to visualize and perform qualitative analysis on the raw data, which were then imported to Compound Discoverer v.3.3 (Thermo Fisher Scientific) to normalize, align, detect and identify compounds. Features were extracted from 0 to 14 min chromatography runs, in the *m*/*z* = 70–800 mass range. Data were aligned according to an adaptive curve alignment model. Compounds were detected using the following parameters settings: mass tolerance was set to 5 ppm, while retention time tolerance was set to 0.2 min; minimum peak intensity was set to 100,000 AU and the signal-to-noise threshold for compound detection was set to 5. The peak rating filter was set to 3. To perform blank subtraction, we maintained max sample/max blank >5. To predict elemental compositions of the compounds, the relative intensity tolerance was set to 30% for isotope pattern matching. For the mzCloud database search, both the precursor and fragment mass tolerance were set to 5 ppm. The databases used for matching compounds in ChemSpider for structural search were BioCyc, the Human Metabolome Database and KEGG, and the mass tolerance in ChemSpider Search was set to 5 ppm. The mass tolerance for matching compounds in Metabolika pathways was set to 5 ppm. Compounds were assigned by comparing annotations using the following nodes in order of priority: (1) mzCloud (2) Predicted Compositions; (3) MassList search; (4) ChemSpider Search; (5) Metabolika search.

### Competition Binding Assay on Cannabinoid Receptors

Binding assays at equilibrium were conducted as described in Di Micco et al., 2024. In summary, membranes from human embryonic kidney 293 (HEK-293) cells overexpressing the human recombinant CB1R (Bmax = 5.7 pmol/mg protein) and CB2R (Bmax = 11.4 pmol/mg protein) were incubated with [^3^H]-CP-55,940, a high-affinity radioligand ([^3^H]-CP-55,940:0.4 nM, KD = 3.08 nM for CB1R and 0.53 nM, KD = 5.89 nM for CBR2). Competition binding assays were performed by displacing [^3^H]-CP-55,940 with increasing concentrations of test compounds to determine binding affinities. Nonspecific binding was defined in the presence of 10 μM WIN55,212–2, used as a heterologous competitor (*K*_i_ = 8.8 nM for CB1R and *K*_i_ = 0.89 nM for CB2R). All compounds were evaluated following the manufacturer’s protocol (PerkinElmer distributed by Revvity, Italy). Displacement assays were carried out by incubating the test compounds with [^3^H]-CP-55,940 for 90 min at 30 °C. The *K*_i_ values were calculated using the Cheng-Prusoff equation, based on the IC_50_ values obtained through GraphPad PRISM software (version 10.2.2). The results are expressed as *K*_i_ (μM), represented as mean ± SEM, and reflect the average of three independent experiments performed in duplicate.

### In Vitro Functional Activity at CB2Rs

The functional activity at CB2Rs was evaluated using the cAMP Hunter assay with an enzyme fragment complementation chemiluminescent detection kit, as previously reported.^119^ Following the manufacturer’s instructions, this assay measured G_i_-coupled cAMP modulation in CB2R-expressing cell lines (DiscoveRx, Fremont, CA). Briefly, CHO-K1 cells overexpressing the human CB2Rs were plated in a 96-well plate at a density of 10,000 cells per well and incubated overnight at 37 °C in 5% CO_2_. Cells were treated with dose–response solutions of the test compound, prepared in cellulo assay buffer containing 25 μM NKH-477, a water-soluble forskolin analog, to stimulate adenylate cyclase and elevate basal cAMP levels. In a different experimental setup, cells were pretreated with a dose–response solution of the compound for 15 min at 37 °C and subsequently challenged with a known CB2R agonist (JWH-133, 4 μM) in the presence of cell assay buffer containing 25 μM NKH-477. Following stimulation, the cells were lysed, and cAMP levels were detected according to the manufacturer’s protocol. Luminescence was measured using the GloMax Multi-Detection System (Promega, Italy). Results were expressed as mean ± SEM and derived from three independent experiments conducted in triplicate. Data were normalized to the response elicited by NKH477 alone, set as 100%. Statistical analyses were performed using GraphPad PRISM software (version 10.1.2, GraphPad Software Inc., San Diego, CA).

### In Vitro Functional Activity at TRP Channels

HEK-293 cells, stably overexpressing recombinant rat TRPM8 or rat TRPA1, were cultured as monolayers in 100 mm Petri dishes using Minimum Essential Media supplemented with 2 mM glutamine, 1% nonessential amino acids, 10% FBS, and maintained at 37 °C under 5% CO_2_. On the day of the experiment, both nontransfected and transfected HEK-293 cells were loaded for 1 h at room temperature in the dark with the calcium-sensitive fluorescent probe Fluo-4 AM (4 μM, dissolved in dimethyl sulfoxide with 0.02% Pluronic F-127). After loading, cells were washed twice with Tyrode’s solution (145 mM NaCl, 2.5 mM KCl, 1.5 mM CaCl_2_, 1.2 mM MgCl_2_, 10 mM *D*-glucose, 10 mM HEPES, pH 7.4) and transferred to a quartz cuvette (approximately 100,000 cells) in a spectrofluorometer (PerkinElmer LS50B; λ_EX_ = 488 nm, λ_EM_ = 516 nm) under continuous stirring. Fluorescence was recorded before and after the addition of various concentrations of test compounds. The observed effects were normalized to the response elicited by ionomycin (4 μM). Baseline fluorescence values from nontransfected HEK-293 cells were subtracted from the measurements obtained in transfected cells. The potency of the test compounds was expressed as the concentration required to elicit a half-maximal agonist effect (EC_50_), measured as half-maximal increases in intracellular calcium concentration ([Ca^2+^]­i). The efficacy of TRPA1 agonists was reported as a percentage of the maximal effect induced by allyl isothiocyanate (AITC, 100 μM). Antagonistic or desensitizing effects were assessed by preincubating cells with the test compounds in the quartz cuvette for 5 min before stimulating them with the specific TRP agonist (AITC, 100 μM for TRPA1, or icilin, 0.25 μM for TRPM8). IC_50_ values were determined as the concentration required to produce a half-maximal inhibition of the agonist effect, with the agonist-induced [Ca^2+^]­i response set at 100%. Measurements of [Ca^2+^]­i in TRPM8-expressing cells were conducted at 22 °C using a Fluorescence Peltier System (PTP-1, PerkinElmer). All experiments were performed in triplicate or greater. Curve fitting (sigmoidal concentration–response with variable slope) and parameter estimation were conducted using GraphPad Prism 10 (GraphPad Software Inc., San Diego, CA).

### IDH1 Enzymatic Assay

The assay was performed in a volume of 100 μL in a 96-well black plate. The IDH1 enzyme assay (R132H; BPS Bioscience, USA) was designed to measure IDH1 enzyme activity by measuring NADPH consumption. For activity measurement, the enzyme (final concentration, 2.5 ng per reaction), α-ketoglutarate (IDH1 substrate; final concentration, 4 mM), NADPH (final concentration, 500 μM), and compounds were incubated for 1 h at room temperature. The compounds were added at different concentrations in the reaction buffer, starting from a final concentration of 30 μM. Buffer with the same amount of DMSO (1%) was used as a negative control. After the incubation time, NADPH detection reagent was added for 5 min at room temperature. The fluorescence signals (excitation/emission, 544 nm/600 nm) were read using a PerkinElmer EnSpire multimode plate reader (PerkinElmer, Waltham, MA, USA). The experiments were performed in triplicate. The IC_50_ values were calculated using GraphPad Prism 8.0 software by nonlinear regression of dose–response inhibition.

### B-Raf Kinase Assay

The assay was performed in a volume of 50 μL in a 96-well white plate. The B-Raf kinase assay (V600E; BPS Bioscience, USA) was designed to measure B-Raf kinase activity by the use of Kinase-Glo MAX (Promega, USA), which detects ATP residual following the kinase reaction. For activity measurement, the kinase (final concentration, 2 ng per reaction), Raf substrate and ATP (final concentration, 500 μM), and compounds were incubated for 45 min at 30 °C. The compounds were added at different concentrations in the reaction buffer, starting from a final concentration of 30 μM. Buffer with the same amount of DMSO (1%) was used as a negative control. After the incubation time, Kinase-Glo MAX detection reagent was added for 15 min at room temperature. The generated luminescent signal was read in end point mode using a PerkinElmer AlphaScreen multimodal plate reader (PerkinElmer, Waltham, MA, USA). Experiments were performed in triplicate. IC_50_ values were calculated with GraphPad Prism 8.0 software by nonlinear dose–response inhibition regression.

### BRK Kinase Assay

The assay was performed in a volume of 50 μL in a 96-well white plate. The BRK kinase assay (BPS Bioscience, USA) was designed to measure BRK kinase activity by the use of ADP-Glo Kinase (Promega, USA), which reveals the ADP produced following the kinase reaction. For activity measurement, the kinase (final concentration, 10 ng per reaction), PTK (Poly-Glu, Tyr 4:1; BRK substrate), ATP (final concentration, 500 μM) and compounds were incubated for 45 min at 30 °C. The compounds were added at different concentrations in the reaction buffer, starting from a final concentration of 30 μM. Buffer with the same amount of DMSO (1%) was used as a negative control. After the incubation time, ADP-Glo Kinase detection reagent was added for 45 min at room temperature. The generated luminescent signal was read in end point mode using a PerkinElmer AlphaScreen multimodal plate reader (PerkinElmer, Waltham, MA, USA). Experiments were performed in triplicate. IC_50_ values were calculated with GraphPad Prism 8.0 software by nonlinear dose–response inhibition regression.

### TNKS1 Assay

The assay was performed in a volume of 50 μL in a 96-well transparent plate. The TNKS1 assay kit (PARP5A; BPS Bioscience, USA) was performed by first coating the 96-well plate with histone proteins, then left at 4 °C overnight. Next, Poly [ADP-ribose] polymerase tankyrase-1 (TNKS1) enzyme (amino acids 1001–1323; final concentration, 1.5 ng per reaction), a mixture of biotinylated NAD^+^ (PARP substrate) and compounds were incubated for 1 h at room temperature. The compounds were added at different concentrations in the reaction buffer, starting from a final concentration of 30 μM. Buffer with the same amount of DMSO (1%) was used as a negative control. After incubation, the plate was treated with streptavidin-HRP followed by the addition of the colorimetric HRP substrate generating a colored solution. The generated signal was read at an absorbance of 450 nm using a microplate reader with UV/vis spectrophotometer (Multiskan Go, Thermo Fisher Scientific, Waltham, MA, USA). Experiments were performed in triplicate. IC_50_ values were calculated with GraphPad Prism 8.0 software by nonlinear dose–response inhibition regression.

### PPARγ-Ligand Binding Domain assay

The assay was performed in a volume of 50 μL in a 96-well plate. In the PPARγ-Ligand Binding Domain screening assay (Cayman, USA), a ligand of Peroxisome proliferator-activated receptor gamma (PPARγ) is conjugated to fluorescein and is used as a displacement probe. To evaluate the binding ability to the target, PPARγ-Ligand Binding Domain (human recombinant), PPARγ Probe – Green and the compounds were incubated in the dark for 1 h at room temperature. The compounds were added at different concentrations in the reaction buffer, starting from a final concentration of 30 μM. Buffer with the same amount of DMSO (1%) was used as a negative control. The fluorescence signals (excitation/emission, 490 nm/520 nm) were read using a PerkinElmer EnSpire multimode plate reader (PerkinElmer, Waltham, MA, USA). The experiments were performed in triplicate. The IC_50_ values were calculated using GraphPad Prism 8.0 software by nonlinear regression of dose–response inhibition.

### PARP1 Assay

The assay was performed in a volume of 50 μL in 96-well transparent plates. The PARP1 assay kit (BPS Bioscience, USA) was performed by first coating the 96-well plate with histone proteins, then left at 4 °C overnight. Next, Poly [ADP-ribose] polymerase 1 (PARP1) enzyme (final concentration, 0.33 ng per reaction), a mixture of biotinylated NAD^+^ (PARP substrate) and compounds were incubated for 1 h at room temperature. The compounds were added at different concentrations in the reaction buffer, starting from a final concentration of 30 μM. Buffer with the same amount of DMSO (1%) was used as a negative control. After incubation, the plate was treated with streptavidin-HRP followed by the addition of the colorimetric HRP substrate generating a colored solution. The generated signal was read at an absorbance of 450 nm using a microplate reader with UV/vis spectrophotometer (Multiskan Go, Thermo Fisher Scientific, Waltham, MA, USA). Experiments were performed in triplicate. IC_50_ values were calculated with GraphPad Prism 8.0 software by nonlinear dose–response inhibition regression.

### CTSK Enzymatic Assay

The assay was performed in a volume of 50 μL in a 96-well black plate. The Cathepsin K inhibitor assay (BPS Bioscience, USA) was designed to measure protease activity by the presence of an internally quenched fluorogenic substrate. Upon cleavage by Cathepsin K (CTSK), the fluorescence of the substrate increases dramatically. For the activity measurement, protease (final concentration, 0.5 ng per reaction), the Fluorogenic Cathepsin K Substrate (final concentration, 70 μM), E-64 (known inhibitor, as positive control) or compounds were incubated for 1 h at room temperature. The compounds were added at different concentrations in the reaction buffer, starting with a final concentration of 30 μM. Buffer with the same amount of DMSO (1%) was used as a negative control. After the incubation time, the fluorescence signals (excitation/emission, 360 nm/460 nm) were read with a PerkinElmer EnSpire multimode plate reader (PerkinElmer, Waltham, MA, USA). Experiments were performed in triplicate. IC_50_ values were calculated with GraphPad Prism 8.0 software by nonlinear dose–response inhibition regression.^120^

### AlphaScreen Assay on BRD4

The activity of synthesized compounds against BRD4 was measured by AlphaScreen assay using BRD4­(BD1) Inhibitor Screening Assay Kit (BSP-32514) as reported before.^121,122^

Compounds were first tested in triplicate at a concentration of 10 μM, using (+)-JQ1 as reference compound. After detecting a promising ability to displace the natural binding (H4Ac) to the protein counterpart (residual BRD4 binding to acetylated H4:6.52 ± 2.96% for compound **63** and 24.17 ± 0.03% for compound **31**), the IC_50_ values were determined.

Briefly, compounds **31**, **63,** or (+)-JQ1 (as reference compound) or DMSO (2%) were preincubated with BRD4­(BD1) protein and histone H4(1–21)­K5/8/12/16Ac-BiotinOH at room temperature for 30 min. After that, 10 μL of acceptor and donor beads (250-fold diluted) were added, protecting them from light. The results were measured 60 min later using the Enspire microplate analyzer (PerkinElmer, California, USA). Each compound was tested in triplicate, and GraphPad Prism (version 8.0) was used to analyze the data, employing the equation log­(inhibitor) vs normalized response-variable slope

### Binding Assays Edited by Eurofins

For displacement assays on G-protein coupled receptor 55 (GPR55), Vasopressin V2 receptor (V2R), Adenosine A1 receptor (A1AR), Fibroblast growth factor receptor 1 (FGFR1), Vascular endothelial growth factor receptor 2 (VEGFR2), Platelet-derived growth factor receptor beta (PDGFRB), Vascular endothelial growth factor receptor 1 (VEGFR1), Thromboxane Synthase (TXS) and Phosphodiesterase 10A (PDE10A), we commissioned the Eurofins company which employed its specific experimental methods, accessible at the link: https://www.eurofins.com/.

## Supplementary Material







## Notes

Figures S1–S60: NMR spectra and HPLC traces of synthesized compounds; Figure S61: Predicted binding between 31 and 63 and TRPA1; Figure S62-S63: Binding of 31 and 63 with the other targets highlighted by IVS; Table S1: Identified targets for 31 cross the bioinformatics tool Super Pred (SP) and transcriptomics data (Protein Atlas); Table S2: Identified targets for 31 cross the bioinformatics tool Swiss Target Prediction (STP) and transcriptomics data (Protein Atlas); Table S3: Identified targets for 63 cross the bioinformatics tool Super Pred (SP) and transcriptomics data (Protein Atlas); Table S4: Identified targets for 63 cross the bioinformatics tool Swiss Target Prediction (STP) and transcriptomics data (Protein Atlas); Figure S64: (A) Principal component analysis score plot showing clustering of 63 vs CTRL, (B) Differential analysis reporting significant proteins, (C) String network, and (D) Enrichment analysis; Table S5: PDB list of the structures contained in the panel used for IVS; references (PDF)
Atomic coordinates of the proteins and of the predicted ligand/protein complexes (ZIP)
Molecular formula strings (CSV)

## List of Abbreviations

ACN: acetonitrile
Boc: *tert*-Butyloxycarbonyl
BRD4: bromodomain-containing protein 4
BRK: protein-tyrosine kinase 6
CB2R: cannabinoid receptor 2
DCM: dichloromethane
DG: diacylglycerol
DMAP: 4-(dimethylamino)­pyridine
DMF: dimethylformamide
DOS: diversity-oriented synthesis
ESI: electrospray ionization
FBS: fetal bovine serum
FC: flash chromatography
GPCR: G protein-coupled receptor
HMDB: Human Metabolome Database
HPLC: high-performance liquid chromatography
HR-MS: high-resolution mass spectrometry
IVS: inverse virtual screening
LD: lipid droplet
LFQ: label-free quantification
MeOH: methanol
MTA: 5′-S-methyl-5′-thioadenosine
MTT: (3-(4, 5-dimethylthiazolyl-2)-2, 5-diphenyltetrazolium bromide
NMR: nuclear magnetic resonance
PCA: principal component analysis
Rf: retention factor
ROS: reactive oxygen species
SI: selectivity index
SMPDB: small molecule pathway database
SP: Super Pred
STP: Swiss Target Prediction
TEA: triethylamine
TFA: trifluoroacetic acid
THF: tetrahydrofuran
ThT: thioflavin T
TIS: triisopropylsilane
TLC: thin layer chromatography
TRPA1: transient receptor potential cation channel subfamily A member 1
TRPM8: transient receptor potential cation channel subfamily M member 8
UHPLC: ultra-high performance liquid chromatography
UnDOS: untargeted diversity-oriented synthesis

## References

[1] Berdigaliyev N., Aljofan M. (2020). An Overview of Drug Discovery and Development. Future Medicinal Chemistry.

[2] Schenone M., Dančík V., Wagner B. K., Clemons P. A. (2013). Target identification
and mechanism of action in chemical biology and drug discovery. Nat. Chem. Biol..

[3] Sinha S., Vohora D. (2018). Drug Discovery and
Development. In Pharmaceutical Medicine and
Translational Clinical Research.

[4] Lood C. S., Koskinen A. M. P. (2015). Harmicine, a Tetracyclic Tetrahydro-β-Carboline:
From the First Synthetic Precedent to Isolation from Natural Sources
to Target-Oriented Synthesis (Review)*. Chem.
Heterocycl. Compd..

[5] Korolchuk A. M., Zolottsev V. A., Misharin A. Y. (2023). Conjugates of Tetrapyrrolic Macrocycles
as Potential Anticancer Target-Oriented Photosensitizers. Top. Curr. Chem..

[6] Magalhães C. M., González-Berdullas P., Duarte D., Correia A. S., Rodríguez-Borges J. E., Vale N., Esteves
da Silva J. C. G., Pinto da Silva L. (2021). Target-Oriented Synthesis of Marine
Coelenterazine Derivatives with Anticancer Activity by Applying the
Heavy-Atom Effect. Biomedicines.

[7] Dincel E., Guzeldemirci N. (2020). Synthesis
and computer - aided drug design studies
of novel thiosemicarbazide derivatives as potent and target - oriented
anti - cancer agents. Med. Sci. Int. Med. J..

[8] Burke M. D., Schreiber S. L. (2004). A Planning
Strategy for Diversity-Oriented Synthesis. Angew.
Chem., Int. Ed..

[9] Schreiber S. L. (2000). Target-Oriented
and Diversity-Oriented Organic Synthesis in Drug Discovery. Science.

[10] O’Connell K. M.
G., Galloway W. R. J. D., Spring D. R. (2013). The Basics of Diversity-Oriented
Synthesis. In Diversity-Oriented Synthesis.

[11] Werner S., Turner D. M., Chambers P. G., Brummond K. M. (2008). Skeletal and appendage
diversity as design elements in the synthesis of a discovery library
of nonaromatic polycyclic 5-iminooxazolidin-2-ones, hydantoins, and
acylureas. Tetrahedron.

[12] Hudson L., Mason J. W., Westphal M. V., Richter M. J. R., Thielman J. R., Hua B. K., Gerry C. J., Xia G., Osswald H. L., Knapp J. M. (2023). Diversity-oriented synthesis
encoded by deoxyoligonucleotides. Nat. Commun..

[13] Spandl R. J., Bender A., Spring D. R. (2008). Diversity-oriented
synthesis; a spectrum
of approaches and results. Org. Biomol. Chem..

[14] Scott K. A., Ropek N., Melillo B., Schreiber S. L., Cravatt B. F., Vinogradova E. V. (2022). Stereochemical
diversity as a source
of discovery in chemical biology. Curr. Res.
Chem. Biol..

[15] Arya P., Joseph R., Gan Z., Rakic B. (2005). Exploring New Chemical
Space by Stereocontrolled Diversity-Oriented Synthesis. Chemistry & Biology.

[16] Taylor M. S., Jacobsen E. N. (2004). Asymmetric catalysis
in complex target synthesis. Proc. Natl. Acad.
Sci. U. S. A..

[17] Balskus E. P., Jacobsen E. N. (2007). Asymmetric Catalysis
of the Transannular Diels-Alder
Reaction. Science.

[18] Spandl R. J., Díaz-Gavilán M., O’Connell K. M. G., Thomas G. L., Spring D. R. (2008). Diversity-oriented
synthesis. Chem. Rec..

[19] Oguri H., Schreiber S. L. (2005). Skeletal
Diversity via a Folding Pathway: Synthesis
of Indole Alkaloid-Like Skeletons. Org. Lett..

[20] Galloway W.
R. J. D., Diáz-Gavilán M., Isidro-Llobet A., Spring D. R. (2009). Synthesis of Unprecedented Scaffold
Diversity. Angew. Chem., Int. Ed..

[21] Rolfe A., Lushington G. H., Hanson P. R. (2010). Reagent based DOS: A “Click,
Click, Cyclize” strategy to probe chemical space. Org. Biomol. Chem..

[22] Sen S., Kamma S. R., Gundla R., Adepally U., Kuncha S., Thirnathi S., Prasad U. V. (2013). A reagent based DOS strategy via
Evans chiral auxiliary: highly stereoselective Michael reaction towards
optically active quinolizidinones, piperidinones and pyrrolidinones. RSC Adv..

[23] Yang S.-J., Choe J.-H., Abdildinova A., Gong Y.-D. (2015). A Highly Efficient
Diversification of 2-Amino/Amido-1,3,4-oxadiazole and 1,3,4-Thiadiazole
Derivatives via Reagent-Based Cyclization of Thiosemicarbazide Intermediate
on Solid-Phase. ACS Comb. Sci..

[24] Evans B. E., Rittle K. E., Bock M. G., DiPardo R. M., Freidinger R. M., Whitter W. L., Lundell G. F., Veber D. F., Anderson P. S., Chang R. S. (1988). Methods for drug discovery: development of
potent, selective, orally effective cholecystokinin antagonists. J. Med. Chem..

[25] Welsch M. E., Snyder S. A., Stockwell B. R. (2010). Privileged
scaffolds for library
design and drug discovery. Curr. Opin. Chem.
Biol..

[26] Zhao H., Dietrich J. (2015). Privileged scaffolds in lead generation. Expert Opinion on Drug Discovery.

[27] Davison E. K., Brimble M. A. (2019). Natural product
derived privileged scaffolds in drug
discovery. Curr. Opin. Chem. Biol..

[28] Ng P. Y., Tang Y., Knosp W. M., Stadler H. S., Shaw J. T. (2007). Synthesis
of Diverse Lactam Carboxamides Leading to the Discovery of a New Transcription-Factor
Inhibitor. Angew. Chem..

[29] Koehler A. N., Shamji A. F., Schreiber S. L. (2003). Discovery
of an Inhibitor of a Transcription
Factor Using Small Molecule Microarrays and Diversity-Oriented Synthesis. J. Am. Chem. Soc..

[30] Kuruvilla F. G., Shamji A. F., Sternson S. M., Hergenrother P. J., Schreiber S. L. (2002). Dissecting glucose signalling with
diversity-oriented
synthesis and small-molecule microarrays. Nature.

[31] Narayan R., Potowski M., Jia Z.-J., Antonchick A. P., Waldmann H. (2014). Catalytic Enantioselective 1,3-Dipolar
Cycloadditions
of Azomethine Ylides for Biology-Oriented Synthesis. Acc. Chem. Res..

[32] Basu S., Ellinger B., Rizzo S., Deraeve C., Schürmann M., Preut H., Arndt H.-D., Waldmann H. (2011). Biology-oriented synthesis
of a natural-product inspired oxepane collection yields a small-molecule
activator of the Wnt-pathway. Proc. Natl. Acad.
Sci. U. S. A..

[33] Marcaurelle L. A., Comer E., Dandapani S., Duvall J. R., Gerard B., Kesavan S., Lee M. D., Liu H., Lowe J. T., Marie J.-C. (2010). An Aldol-Based Build/Couple/Pair
Strategy for
the Synthesis of Medium- and Large-Sized Rings: Discovery of Macrocyclic
Histone Deacetylase Inhibitors. J. Am. Chem.
Soc..

[34] Mahnashi M., Elgazwi S. M., Ahmed M. S., Halaweish F. T. (2019). Cucurbitacins
inspired organic synthesis: Potential dual inhibitors targeting EGFR–MAPK
pathway. Eur. J. Med. Chem..

[35] Zhao X., Zhang J., Zheng Z., Xu R. (2017). Facile Synthesis for
Benzo-1,4-Oxazepine Derivatives by Tandem Transformation of C-N Coupling/C-H
Carbonylation. Molecules.

[36] Sapegin A., Krasavin M. (2019). Convenient Assembly
of Privileged (Hetero)­Arene-Fused
Benzo[1.4]­oxazepines via Two Tandem SNAr Events. Part 1–On
the Importance of the Intermittent Smiles Rearrangement. Eur. J. Org. Chem..

[37] Gupta N., Saini V., Basavarajaiah S. M., Dar M. O., Das R., Dahiya R. S. (2023). 1,3-Oxazine as a
Promising Scaffold for the Development
of Biologically Active Lead Molecules. ChemistrySelect.

[38] Tang Z., Tan Y., Chen H., Wan Y. (2023). Benzoxazine: A Privileged Scaffold
in Medicinal Chemistry. Curr. Med. Chem..

[39] Alsibaee A. M., Al-Yousef H. M., Al-Salem H. S. (2023). Quinazolinones, the Winning Horse
in Drug Discovery. Molecules.

[40] Gheidari D., Mehrdad M., Maleki S. (2022). Recent advances
in synthesis of quinazoline-2,4­(1H,3H)-diones:
Versatile building blocks in N-heterocyclic compounds. Appl. Organomet. Chem..

[41] Arora N., Dhiman P., Kumar S., Singh G., Monga V. (2020). Recent advances
in synthesis and medicinal chemistry of benzodiazepines. Bioorg. Chem..

[42] Joseph C. G., Wilson K. R., Wood M. S., Sorenson N. B., Phan D. V., Xiang Z., Witek R. M., Haskell-Luevano C. (2008). The 1,4-Benzodiazepine-2,5-dione
Small Molecule Template Results in Melanocortin Receptor Agonists
with Nanomolar Potencies. J. Med. Chem..

[43] Stewart C.
D., White N. G., Barrow R. A., Reekie T. A. (2024). Synthesis and spectroscopic
investigation of substituted piperazine-2,5-dione derivatives. Tetrahedron.

[44] Lattanzi A. (2022). 3,4-Dihydroquinoxalin-2-one
privileged motif: A journey from classical chiral tools based synthesis
to modern catalytic enantioselective strategies. Tetrahedron Chem..

[45] Haghighijoo Z., Zamani L., Moosavi F., Emami S. (2022). Therapeutic
potential
of quinazoline derivatives for Alzheimer’s disease: A comprehensive
review. Eur. J. Med. Chem..

[46] Lauro G., Romano A., Riccio R., Bifulco G. (2011). Inverse Virtual Screening
of Antitumor Targets: Pilot Study on a Small Database of Natural Bioactive
Compounds. J. Nat. Prod..

[47] Lauro G., Masullo M., Piacente S., Riccio R., Bifulco G. (2012). Inverse Virtual
Screening allows the discovery of the biological activity of natural
compounds. Biorg. Med. Chem..

[48] Laezza C., Pagano C., Navarra G., Pastorino O., Proto M. C., Fiore D., Piscopo C., Gazzerro P., Bifulco M. (2020). The Endocannabinoid System: A Target
for Cancer Treatment. Int. J. Mol. Sci..

[49] Aviz-Amador A., Contreras-Puentes N., Mercado-Camargo J. (2021). Virtual screening using docking and
molecular dynamics of cannabinoid analogs against CB1 and CB2 receptors. Comput. Biol. Chem..

[50] Xing C., Zhuang Y., Xu T.-H., Feng Z., Zhou X. E., Chen M., Wang L., Meng X., Xue Y., Wang J. (2020). Cryo-EM
Structure of the Human Cannabinoid Receptor
CB2-Gi Signaling Complex. Cell.

[51] Palchevskyi S., Czarnocki-Cieciura M., Vistoli G., Gervasoni S., Nowak E., Beccari A. R., Nowotny M., Talarico C. (2023). Structure
of human TRPM8 channel. Commun. Biol..

[52] Vershinin Z., Feldman M., Werner T., Weil L. E., Kublanovsky M., Abaev-Schneiderman E., Sklarz M., Lam E. Y. N., Alasad K., Picaud S. (2021). BRD4 methylation by the methyltransferase SETD6 regulates
selective transcription to control mRNA translation. Science Advances.

[53] Hu J., Pan D., Li G., Chen K., Hu X. (2022). Regulation of programmed
cell death by Brd4. Cell Death & Disease.

[54] Dong J., Wang X. (2023). Identification of novel
BRD4 inhibitors by pharmacophore screening,
molecular docking, and molecular dynamics simulation. J. Mol. Struct..

[55] Gfeller D., Grosdidier A., Wirth M., Daina A., Michielin O., Zoete V. (2014). SwissTargetPrediction: a web server for target prediction of bioactive
small molecules. Nucleic Acids Res..

[56] Gallo K., Goede A., Preissner R., Gohlke B.-O. (2022). SuperPred 3.0: drug
classification and target prediction—a machine learning approach. Nucleic Acids Res..

[57] Cruz A. L. S., Barreto E. D. A., Fazolini N. P. B., Viola J. P. B., Bozza P. T. (2020). Lipid droplets: platforms with multiple
functions in
cancer hallmarks. Cell Death Dis..

[58] Lumaquin-Yin D., Montal E., Johns E., Baggiolini A., Huang T.-H., Ma Y., LaPlante C., Suresh S., Studer L., White R. M. (2023). Lipid droplets are
a metabolic vulnerability
in melanoma. Nat. Commun..

[59] Dadsena S., Bockelmann S., Mina J. G. M., Hassan D. G., Korneev S., Razzera G., Jahn H., Niekamp P., Müller D., Schneider M. (2019). Ceramides bind VDAC2 to trigger mitochondrial
apoptosis. Nat. Commun..

[60] Lu S. C., Mato J. M. (2008). S-Adenosylmethionine
in cell growth, apoptosis and
liver cancer. J. Gastroenterol. Hepatol..

[61] Redondo-Muñoz M., Rodriguez-Baena F. J., Aldaz P., Caballé-Mestres A., Moncho-Amor V., Otaegi-Ugartemendia M., Carrasco-Garcia E., Olias-Arjona A., Lasheras-Otero I., Santamaria E. (2023). Metabolic rewiring induced
by ranolazine improves melanoma responses
to targeted therapy and immunotherapy. Nature
Metabolism.

[62] Andreu-Pérez P., Hernandez-Losa J., Moliné T., Gil R., Grueso J., Pujol A., Cortés J., Avila M. A., Recio J. A. (2010). Methylthioadenosine
(MTA) inhibits melanoma cell proliferation and in vivotumor growth. BMC Cancer.

[63] Yee N. (2015). Roles of TRPM8
Ion Channels in Cancer: Proliferation, Survival, and Invasion. Cancers (Basel).

[64] Ciaglia T., Vestuto V., Bertamino A., González-Muñiz R., Gómez-Monterrey I. (2023). On the modulation
of TRPM channels:
Current perspectives and anticancer therapeutic implications. Front. Oncol..

[65] Ochoa S. V., Casas Z., Albarracín S.
L., Sutachan J. J., Torres Y. P. (2023). Therapeutic potential of TRPM8 channels in cancer treatment. Front. Pharmacol..

[66] Moccia F., Montagna D. (2023). Transient Receptor Potential Ankyrin
1 (TRPA1) Channel
as a Sensor of Oxidative Stress in Cancer Cells. Cells.

[67] Faris P., Rumolo A., Pellavio G., Tanzi M., Vismara M., Berra-Romani R., Gerbino A., Corallo S., Pedrazzoli P., Laforenza U. (2023). Transient receptor potential ankyrin 1 (TRPA1)
mediates reactive oxygen species-induced Ca2+ entry, mitochondrial
dysfunction, and caspase-3/7 activation in primary cultures of metastatic
colorectal carcinoma cells. Cell Death Discovery.

[68] Wu Y.-T., Yen S.-L., Li C.-F., Chan T.-C., Chen T.-J., Lee S.-W., He H.-L., Chang I. W., Hsing C.-H., Shiue Y.-L. (2016). Overexpression of
Transient Receptor Protein Cation
Channel Subfamily A Member 1, Confers an Independent Prognostic Indicator
in Nasopharyngeal Carcinoma. Journal of Cancer.

[69] Francesconi O., Corzana F., Kontogianni G.-I., Pesciullesi G., Gualdani R., Supuran C. T., Angeli A., Kavasi R. M., Chatzinikolaidou M., Nativi C. (2022). Lipoyl-Based Antagonists of Transient
Receptor Potential Cation A (TRPA1) Downregulate Osteosarcoma Cell
Migration and Expression of Pro-Inflammatory Cytokines. ACS Pharmacology & Translational Science.

[70] Lee H. J., Chen Z., Collard M., Chen F., Chen J. G., Wu M., Alani R. M., Cheng J.-X. (2021). Multimodal Metabolic Imaging Reveals
Pigment Reduction and Lipid Accumulation in Metastatic Melanoma. BME Front..

[71] Carrasco C., Naziroglu M., Pecze L., Pariente J. A. (2018). Editorial: Involvements
of TRP Channels and Oxidative Stress in Pain. Front. Physiol..

[72] Vestuto V., Di Sarno V., Musella S., Di Dona G., Moltedo O., Gomez-Monterrey I. M., Bertamino A., Ostacolo C., Campiglia P., Ciaglia T. (2023). New Frontiers on ER Stress Modulation: Are TRP Channels
the Leading Actors?. Int. J. Mol. Sci..

[73] De
Logu F., de Araujo D. S. M., Ugolini F., Iannone L. F., Vannucchi M., Portelli F., Landini L., Titiz M., De Giorgi V., Geppetti P. (2021). The TRPA1 Channel Amplifies
the Oxidative Stress Signal in Melanoma. Cells.

[74] Parra V., Moraga F., Kuzmicic J., López-Crisosto C., Troncoso R., Torrealba N., Criollo A., Díaz-Elizondo J., Rothermel B. A., Quest A. F. G. (2013). Calcium and mitochondrial
metabolism in ceramide-induced cardiomyocyte death. Biochimica et Biophysica Acta (BBA) - Molecular Basis of Disease.

[75] Martínez-Martínez E., Martín-Ruiz A., Martín P., Calvo V., Provencio M., García J. M. (2016). CB2 cannabinoid receptor activation promotes colon
cancer progression via AKT/GSK3β signaling pathway. Oncotarget.

[76] Bachari A., Piva T. J., Salami S. A., Jamshidi N., Mantri N. (2020). Roles of Cannabinoids
in Melanoma: Evidence from In Vivo Studies. Int. J. Mol. Sci..

[77] Xu S., Ma H., Bo Y., Shao M. (2019). The oncogenic role of CB2 in the
progression of non-small-cell lung cancer. Biomed.
Pharmacother..

[78] Muller C., Morales P., Reggio P. H. (2019). Cannabinoid Ligands
Targeting TRP
Channels. Front. Mol. Neurosci..

[79] Zhao H., Zhao Z., Yang J., Fang X., Li H. (2012). Cannabinoid
receptor 2 is upregulated in melanoma. Journal
of Cancer Research and Therapeutics.

[80] Marzęda P., Drozd M., Wróblewska-Łuczka P., Łuszczki J. J. (2021). Cannabinoids and their derivatives in struggle against
melanoma. Pharmacological Reports.

[81] Bachari A., Nassar N., Telukutla S., Zomer R., Piva T. J., Mantri N. (2024). Evaluating the Mechanism of Cell Death in Melanoma
Induced by the Cannabis Extract PHEC-66. Cells.

[82] Altendorfer E., Mochalova Y., Mayer A. (2022). BRD4: a general regulator of transcription
elongation. Transcription.

[83] Wang R., Li Q., Helfer C. M., Jiao J., You J. (2012). Bromodomain Protein
Brd4 Associated with Acetylated Chromatin Is Important for Maintenance
of Higher-order Chromatin Structure. J. Biol.
Chem..

[84] Lu L., Chen Z., Lin X., Tian L., Su Q., An P., Li W., Wu Y., Du J., Shan H. (2020). Inhibition of BRD4 suppresses the malignancy of breast cancer cells
via regulation of Snail. Cell Death & Differentiation.

[85] Floyd S. R., Pacold M. E., Huang Q., Clarke S. M., Lam F. C., Cannell I. G., Bryson B. D., Rameseder J., Lee M. J., Blake E. J. (2013). The bromodomain protein
Brd4 insulates chromatin from DNA damage signalling. Nature.

[86] Uppal S., Gegonne A., Chen Q., Thompson P. S., Cheng D., Mu J., Meerzaman D., Misra H. S., Singer D. S. (2019). The Bromodomain
Protein 4 Contributes to the Regulation of Alternative Splicing. Cell Rep..

[87] Lam F. C., Kong Y. W., Huang Q., Vu Han T.-L., Maffa A. D., Kasper E. M., Yaffe M. B. (2020). BRD4 prevents the
accumulation of
R-loops and protects against transcription–replication collision
events and DNA damage. Nat. Commun..

[88] Marié I. J., Chang H.-M., Levy D. E. (2018). HDAC stimulates
gene expression through
BRD4 availability in response to IFN and in interferonopathies. Journal of Experimental Medicine.

[89] Omidkhah N., Hadizadeh F., Ghodsi R. (2021). Dual HDAC/BRD4 inhibitors against
cancer. Medicinal Chemistry Research.

[90] Moreno N., Holsten T., Mertins J., Zhogbi A., Johann P., Kool M., Meisterernst M., Kerl K. (2017). Combined BRD4 and CDK9
inhibition as a new therapeutic approach in malignant rhabdoid tumors. Oncotarget.

[91] Liu R. (2023). Brd4-dependent
CDK9 expression induction upon sustained pharmacological inhibition
of P-TEFb kinase activity. Biochem. Biophys.
Res. Commun..

[92] Huang M., Zhu L., Garcia J. S., Li M. X., Gentles A. J., Mitchell B. S. (2018). Brd4 regulates
the expression of essential autophagy genes and Keap1 in AML cells. Oncotarget.

[93] Sakamaki J.-I., Wilkinson S., Hahn M., Tasdemir N., O’Prey J., Clark W., Hedley A., Nixon C., Long J. S., New M. (2017). Bromodomain Protein BRD4 Is a Transcriptional Repressor
of Autophagy and Lysosomal Function. Mol. Cell.

[94] Ouyang L., Zhang L., Liu J., Fu L., Yao D., Zhao Y., Zhang S., Wang G., He G., Liu B. (2017). Discovery of a Small-Molecule Bromodomain-Containing
Protein 4 (BRD4)
Inhibitor That Induces AMP-Activated Protein Kinase-Modulated Autophagy-Associated
Cell Death in Breast Cancer. J. Med. Chem..

[95] Liu S., Cadoux-Hudson T., Schofield C. J. (2020). Isocitrate dehydrogenase variants
in cancer — Cellular consequences and therapeutic opportunities. Curr. Opin. Chem. Biol..

[96] Kudchadkar R., Paraiso K. H. T., Smalley K. S. M. (2012). Targeting Mutant
BRAF in Melanoma. Cancer Journal.

[97] Blackburn B. K., Lee A., Baier M., Kohl B., Olivero A. G., Matamoros R., Robarge K. D., McDowell R. S. (1997). From Peptide to Non-Peptide. 3. Atropisomeric
GPIIbIIIa Antagonists Containing the 3,4-Dihydro-1H-1,4-benzodiazepine-2,5-dione
Nucleus. J. Med. Chem..

[98] Schrödinger Release 2021-1: Ligprep; Schrödinger LLC: New York, NY, 2021.

[99] Friesner R. A., Banks J. L., Murphy R. B., Halgren T. A., Klicic J. J., Mainz D. T., Repasky M. P., Knoll E. H., Shelley M., Perry J. K. (2004). Glide: a new approach for rapid, accurate docking
and scoring. 1. Method and assessment of docking accuracy. J. Med. Chem..

[100] Schrödinger Release 2021-1: Glide; Schrödinger LLC: New York, NY, 2021.

[101] De Vita S., Lauro G., Ruggiero D., Terracciano S., Riccio R., Bifulco G. (2019). Protein Preparation Automatic Protocol
for High-Throughput Inverse Virtual Screening: Accelerating the Target
Identification by Computational Methods. J.
Chem. Inf. Model..

[102] Friesner R. A., Murphy R. B., Repasky M. P., Frye L. L., Greenwood J. R., Halgren T. A., Sanschagrin P. C., Mainz D. T. (2006). Extra precision glide: docking and scoring incorporating
a model of hydrophobic enclosure for protein-ligand complexes. J. Med. Chem..

[103] Halgren T. A., Murphy R. B., Friesner R. A., Beard H. S., Frye L. L., Pollard W. T., Banks J. L. (2004). Glide:
a new approach
for rapid, accurate docking and scoring. 2. Enrichment factors in
database screening. J. Med. Chem..

[104] Liu C., Reese R., Vu S., Rougé L., Shields S. D., Kakiuchi-Kiyota S., Chen H., Johnson K., Shi Y. P., Chernov-Rogan T., Greiner D. M. Z. (2021). A Non-covalent Ligand Reveals Biased Agonism
of the TRPA1 Ion Channel. Neuron..

[105] Trentini M., Zanolla I., Tiengo E., Zanotti F., Sommella E., Merciai F., Campiglia P., Licastro D., Degasperi M., Lovatti L. (2024). Link between
organic nanovescicles from vegetable kingdom and human cell physiology:
intracellular calcium signalling. J. Nanobiotechnol..

[106] Gfeller D., Michielin O., Zoete V. (2013). Shaping the interaction
landscape of bioactive molecules. Bioinformatics.

[107] Dunkel M., Gunther S., Ahmed J., Wittig B., Preissner R. (2008). SuperPred:
drug classification and target prediction. Nucleic
Acids Res..

[108] Daina A., Michielin O., Zoete V. (2019). SwissTargetPrediction:
updated data and new features for efficient prediction of protein
targets of small molecules. Nucleic Acids Res..

[109] Nickel J., Gohlke B.-O., Erehman J., Banerjee P., Rong W. W., Goede A., Dunkel M., Preissner R. (2014). SuperPred:
update on drug classification and target prediction. Nucleic Acids Res..

[110] Gaulton A., Bellis L. J., Bento A. P., Chambers J., Davies M., Hersey A., Light Y., McGlinchey S., Michalovich D., Al-Lazikani B. (2012). ChEMBL: a large-scale
bioactivity database for drug discovery. Nucleic
Acids Res..

[111] Liu T., Lin Y., Wen X., Jorissen R. N., Gilson M. K. (2007). BindingDB:
a web-accessible database of experimentally determined protein-ligand
binding affinities. Nucleic Acids Res..

[112] Gunther S., Kuhn M., Dunkel M., Campillos M., Senger C., Petsalaki E., Ahmed J., Urdiales E. G., Gewiess A., Jensen L. J. (2007). SuperTarget and Matador:
resources for exploring drug-target relationships. Nucleic Acids Res..

[113] Hecker N., Ahmed J., von Eichborn J., Dunkel M., Macha K., Eckert A., Gilson M. K., Bourne P. E., Preissner R. (2012). SuperTarget
goes quantitative: update
on drug-target interactions. Nucleic Acids Res..

[114] Armstrong M. S., Morris G. M., Finn P. W., Sharma R., Moretti L., Cooper R. I., Richards W. G. (2010). ElectroShape: fast
molecular similarity calculations incorporating shape, chirality and
electrostatics. Journal of Computer-Aided Molecular
Design.

[115] Covelli V., Cozzolino A., Rizzo P., Rodriquez M., Vestuto V., Bertamino A., Daniel C., Guerra G. (2023). Salicylic
Acid Release from Syndiotactic Polystyrene Staple Fibers. Molecules.

[116] Aquino G., Basilicata M. G., Crescenzi C., Vestuto V., Salviati E., Cerrato M., Ciaglia T., Sansone F., Pepe G., Campiglia P. (2023). Optimization
of microwave-assisted extraction of antioxidant compounds from spring
onion leaves using Box–Behnken design. Sci. Rep..

[117] Ciaglia T., Miranda M. R., Di Micco S., Vietri M., Smaldone G., Musella S., Di Sarno V., Auriemma G., Sardo C., Moltedo O. (2024). Neuroprotective Potential
of Indole-Based Compounds: A Biochemical Study on Antioxidant Properties
and Amyloid Disaggregation in Neuroblastoma Cells. Antioxidants.

[118] Soldani C., Croce A. C., Bottone M. G., Fraschini A., Biggiogera M., Bottiroli G., Pellicciari C. (2007). Apoptosis
in tumour cells photosensitized with Rose Bengal acetate is induced
by multiple organelle photodamage. Histochemistry
and Cell Biology.

[119] Mugnaini C., Brizzi A., Paolino M., Scarselli E., Castelli R., de Candia M., Gambacorta N., Nicolotti O., Kostrzewa M., Kumar P. (2024). Novel
Dual-Acting Hybrids Targeting Type-2 Cannabinoid Receptors and Cholinesterase
Activity Show Neuroprotective Effects In Vitro and Amelioration of
Cognitive Impairment In Vivo. ACS Chem. Neurosci..

[120] Di Micco S., Rahimova R., Sala M., Scala M. C., Vivenzio G., Musella S., Andrei G., Remans K., Mammri L., Snoeck R. (2022). Rational design of the
zonulin inhibitor AT1001 derivatives as potential anti SARS-CoV-2. Eur. J. Med. Chem..

[121] Colarusso E., Gazzillo E., Boccia E., Giordano A., Chini M. G., Bifulco G., Lauro G. (2022). 6-Methylquinazolin-4­(3H)-one
Based Compounds as BRD9 Epigenetic Reader Binders: A Rational Combination
of in silico Studies and Chemical Synthesis. Eur. J. Org. Chem..

[122] Colarusso E., Gazzillo E., Boccia E., Terracciano S., Bruno I., Bifulco G., Chini M. G., Lauro G. (2024). Identification
of Novel Bromodomain-Containing Protein 4 (BRD4) Binders through 3D
Pharmacophore-Based Repositioning Screening Campaign. Molecules.

