# Supplementary material for: Untargeted Diversity-Oriented Synthesis for the Discovery of New Antitumor Agents: An Integrated Approach of Inverse Virtual Screening, Bioinformatics, and Omics for Target Deconvolution
Source: J Med Chem. 2025 Jul 24;68(15):16483–517. doi: 10.1021/acs.jmedchem.5c01344 (PMC12362587; doi:10.1021/acs.jmedchem.5c01344)
Supplement: Supplementary file 1 [file jm5c01344_si_001.pdf]

SUPPLEMENTARY INFORMATION

Untargeted Diversity-Oriented Synthesis (UnDOS) for the discovery of new antitumor agents: an integrated approach of IVS, bioinformatics and omics for targets deconvolution.

Tania Ciaglia,[¥,ǁ] Valeria Napolitano,[¥,ǁ] Maria Rosaria Miranda,[¥,ǁ,§] Danila La Gioia,[¥,§] Simona Musella,[¥] Aniello Schiano Moriello[ᵀ], Fabrizio Merciai,[¥] Veronica Di Sarno,[¥] Simona De Vita,[¥] Ester Colarusso,[¥] Gerardina Smaldone,[¥] Francesca Di Matteo, [¥] Eduardo Maria Sommella,[¥] Poulami Kumar,[ᵀ] Marco Allarà,[ᵀ] Alessia Ligresti,[ᵀ] Isabel M. Gomez-Monterrey,[#] Giuseppe Bifulco,[¥] Gianluigi Lauro,[¥] Pietro Campiglia,[¥] Carmine Ostacolo,[¥] Vincenzo Vestuto, [¥,*] Alessia Bertamino.[¥,*]

[¥] Department of Pharmacy, University of Salerno, Via G. Paolo II 132, 84084, Fisciano, Salerno, Italy.

[ᵀ] Institute of Biomolecular Chemistry, Consiglio Nazionale delle Ricerche, Comprensorio Olivetti, Via Campi Flegrei, 34, 80078 Pozzuoli, Naples, Italy.

[#] Department of Pharmacy, University Federico II of Naples, Via D. Montesano 49, 80131, Naples, Italy.

[§]PhD Program in Drug Discovery and Development, University of Salerno,Via G. Paolo II 132, 84084, Fisciano, SA, Italy.

Table of contents:

1. **Figures S1-S60:** NMR spectra and HPLC traces of synthesized compounds **S2-S61**
2. **Figure S61:** Predicted binding between **31** and **63** and TRPA1 **S62**
3. Interacting targets of compounds **31** and **63** predicted by IVS **S63-S66**
4. **Table S1**. Identified targets for **31** cross the bioinformatics tool Super Pred (SP) and transcriptomics data (Protein Atlas) **S67**
5. **Table S2**. Identified targets for **31** cross the bioinformatics tool Swiss Target Prediction (STP) and transcriptomics data (Protein Atlas) **S67-S68**
6. **Table S3**. Identified targets for **63** cross the bioinformatics tool Super Pred (SP) and transcriptomics data (Protein Atlas) **S68-S69**
7. **Table S4**. Identified targets for **63** cross the bioinformatics tool Swiss Target Prediction (STP) and transcriptomics data (Protein Atlas) **S69-S71**
8. **Figure S64:** A) Principal component analysis score plot showing clustering of **63** vs CTRL, B) Differential analysis reporting significant proteins, C) String network, and D) Enrichment analysis **S72**
9. **Table S5.** PDB list of the structures contained in the panel used for IVS **S73-S82**
10. **References** **S83**

NMR spectra and HPLC traces of synthesized compounds

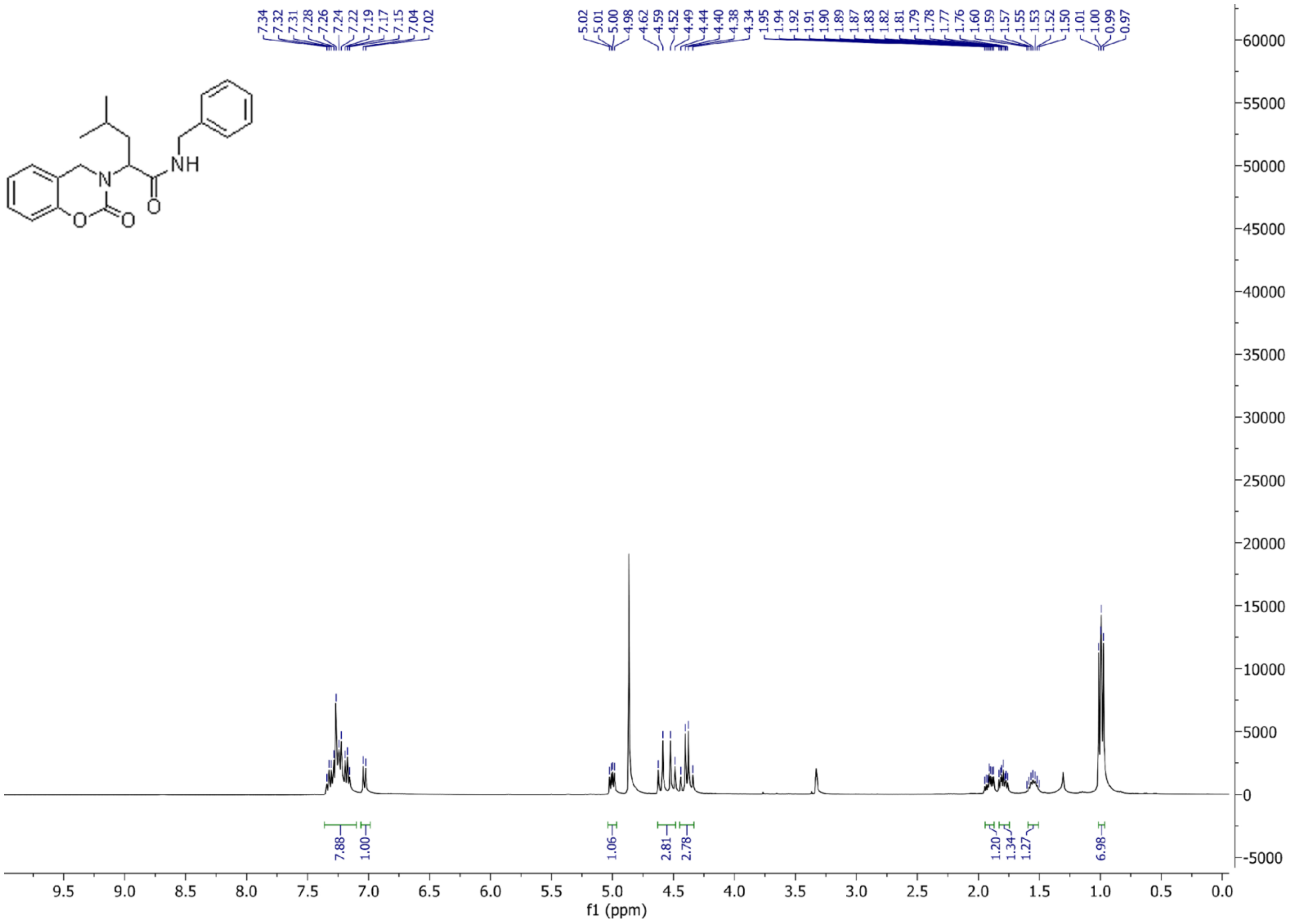

Figure S1: 1H NMR spectra of compound **7**

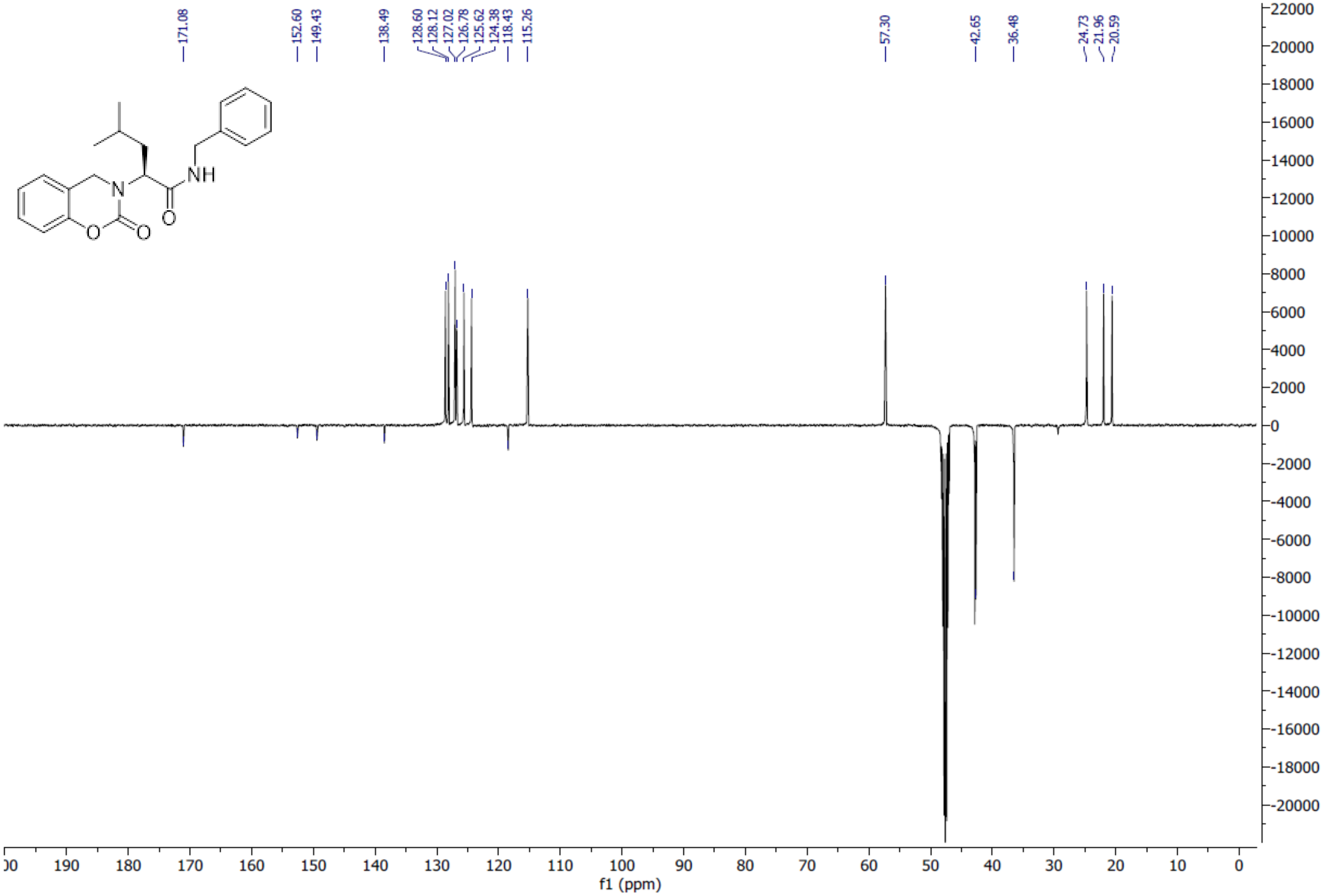

Figure S2: APTspectra of compound **7**

RT	% AREA
10.1	95

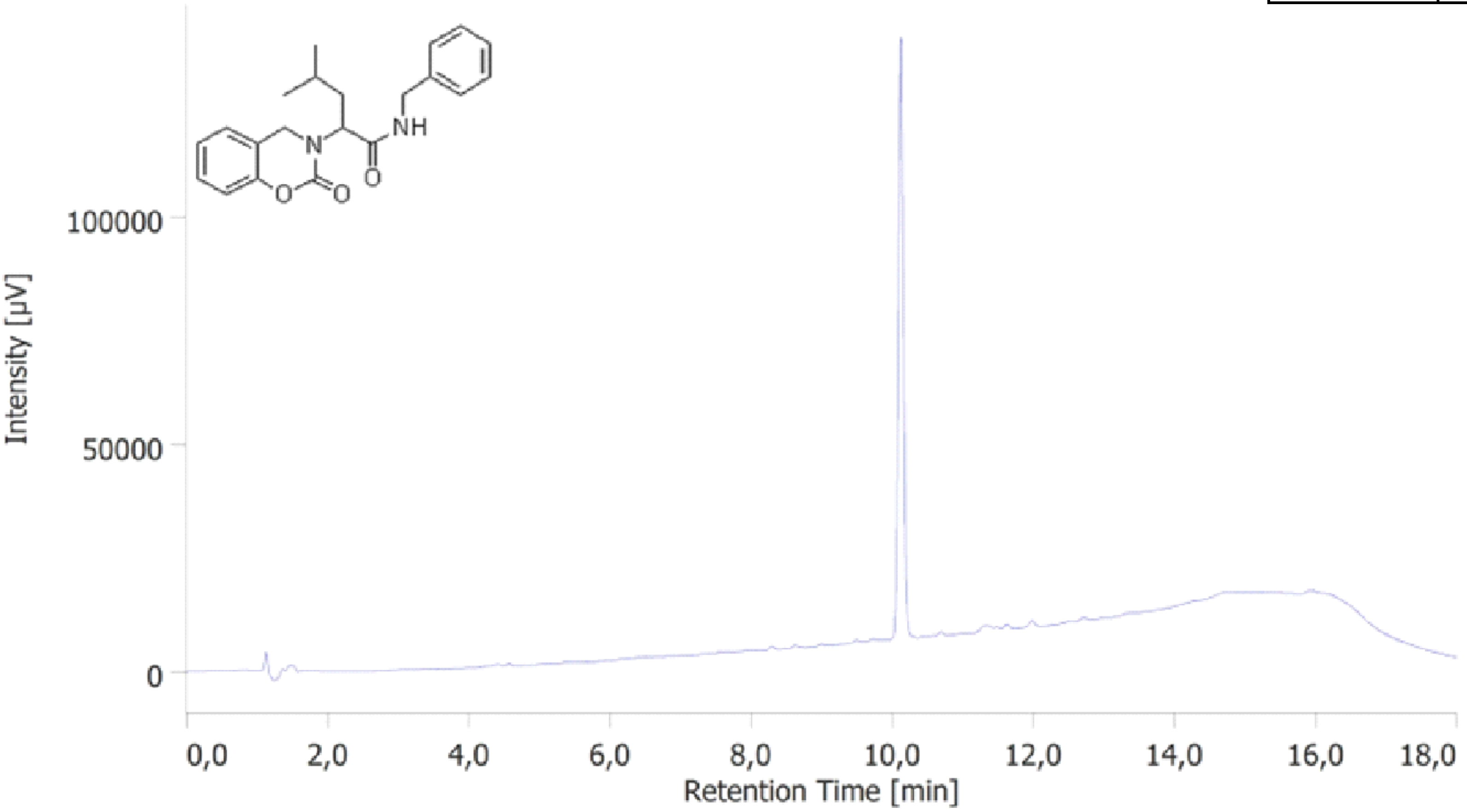

Figure S3: HPLC trace of compound **7**

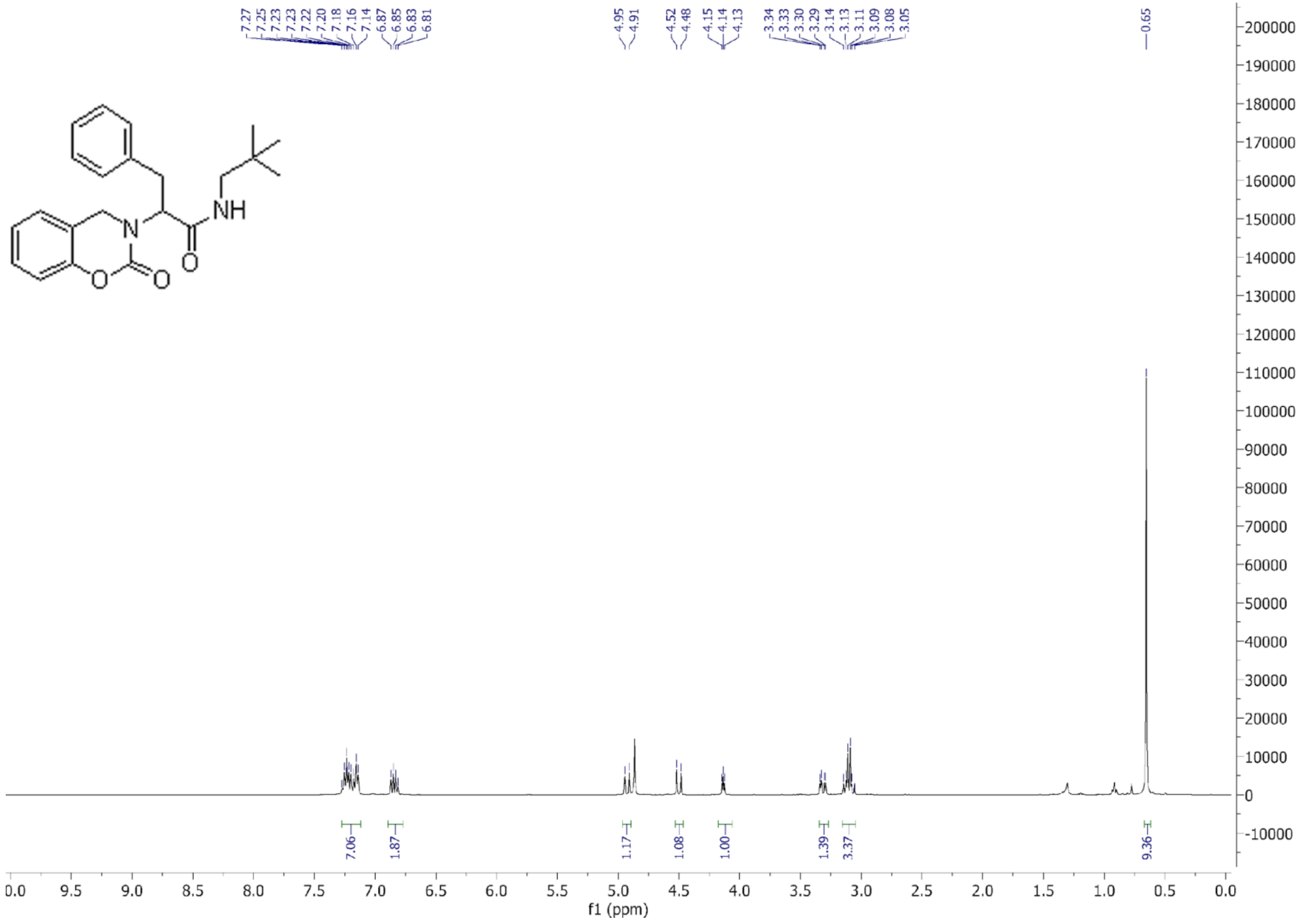

Figure S4: 1H NMR spectra of compound **8**

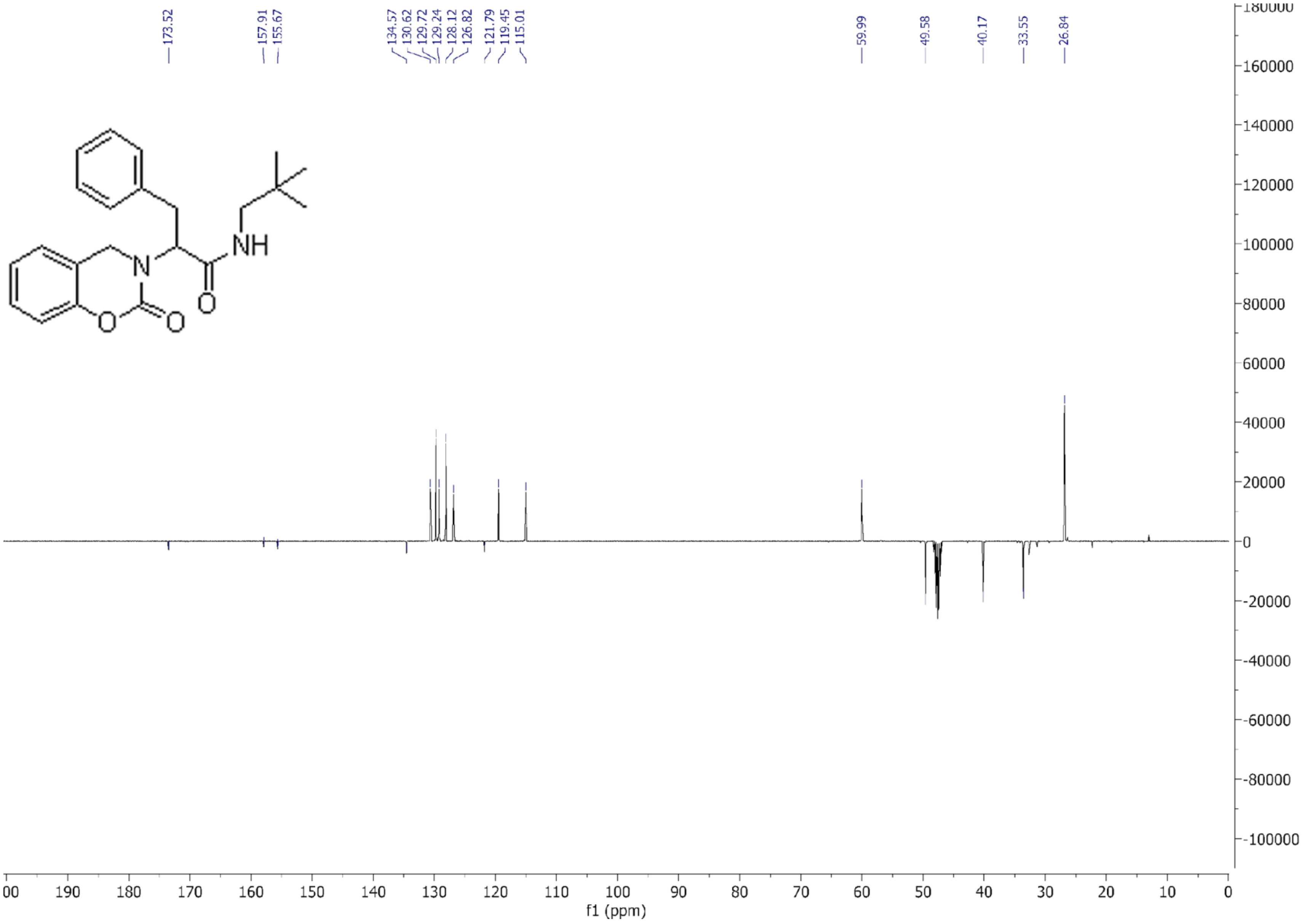

Figure S5: APTspectra of compound **8**

RT	% AREA
10.5	97

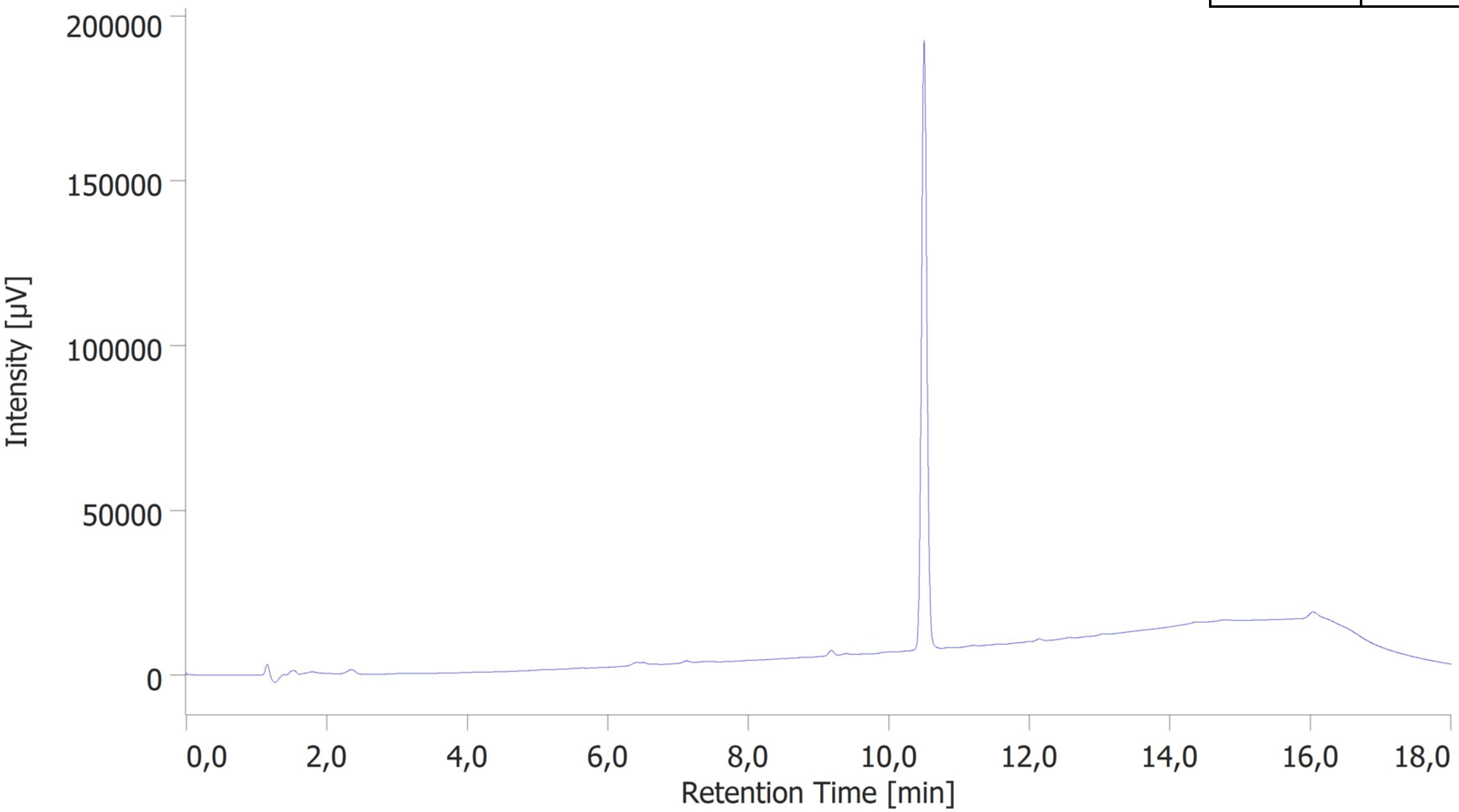

Figure S6: HPLC trace of compound **8**

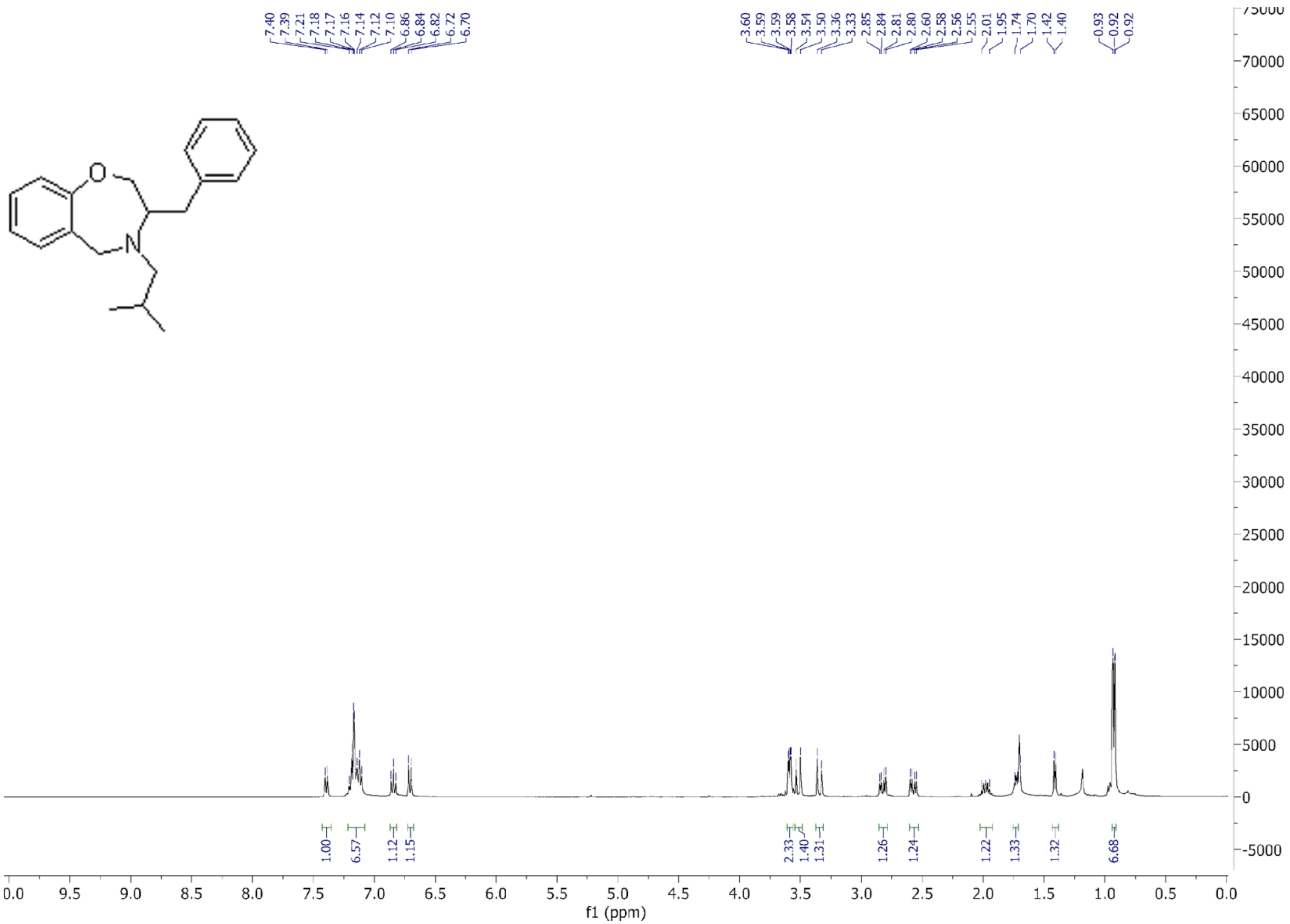

Figure S7: 1H NMR spectra of compound **13**

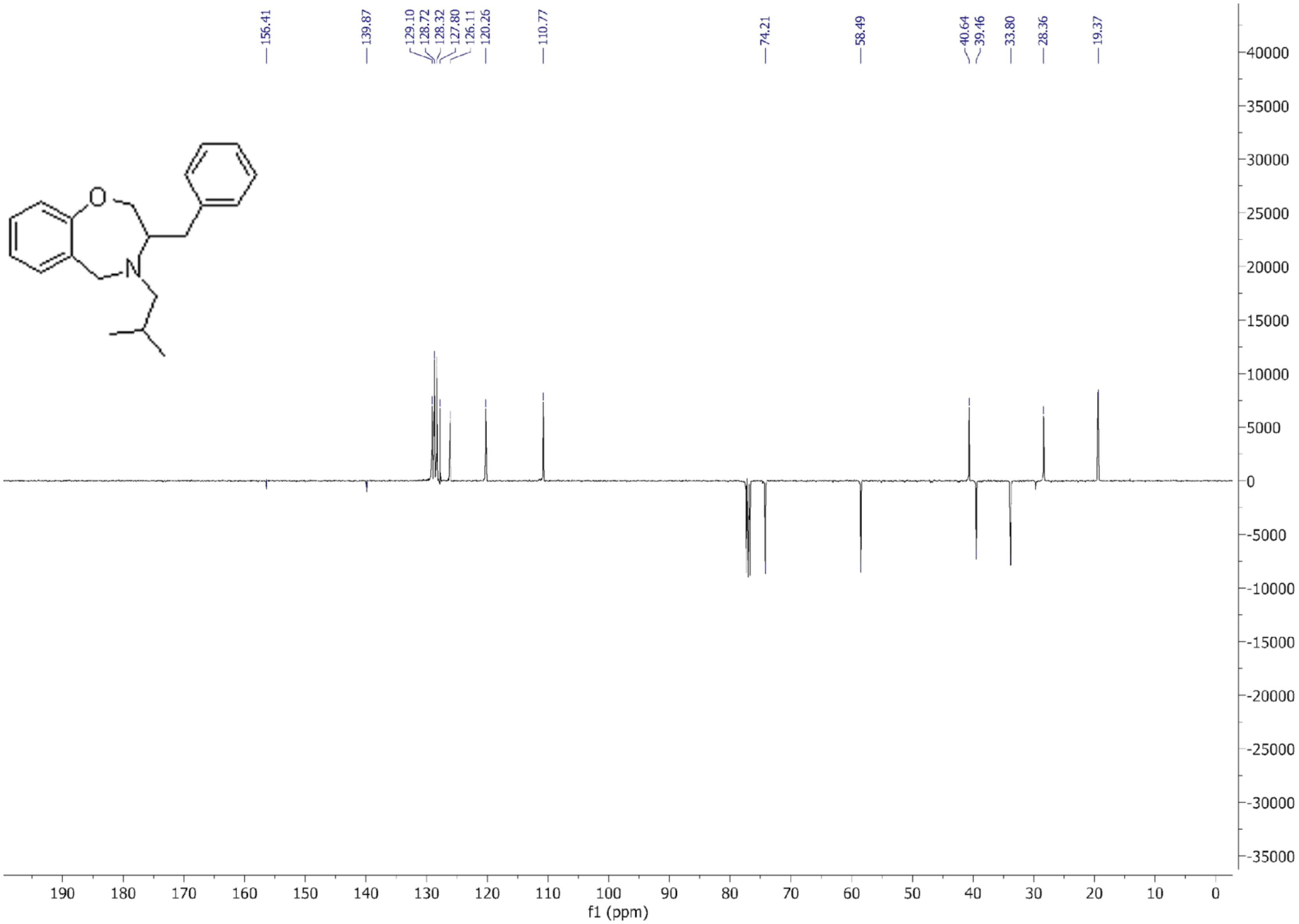

Figure S8: APTspectra of compound **13**

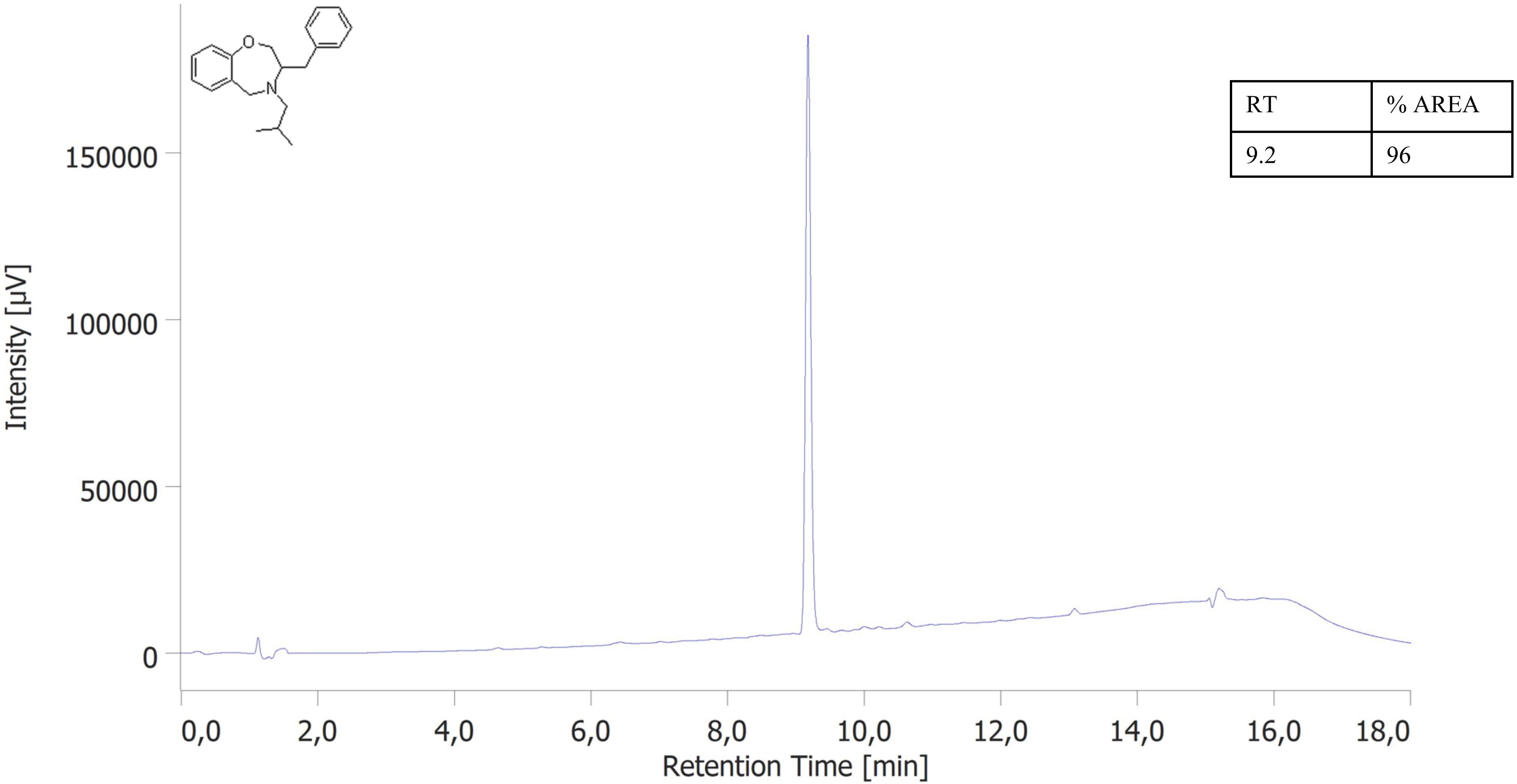

RT	% AREA
9.2	96

Figure S9: HPLC trace of compound **13**

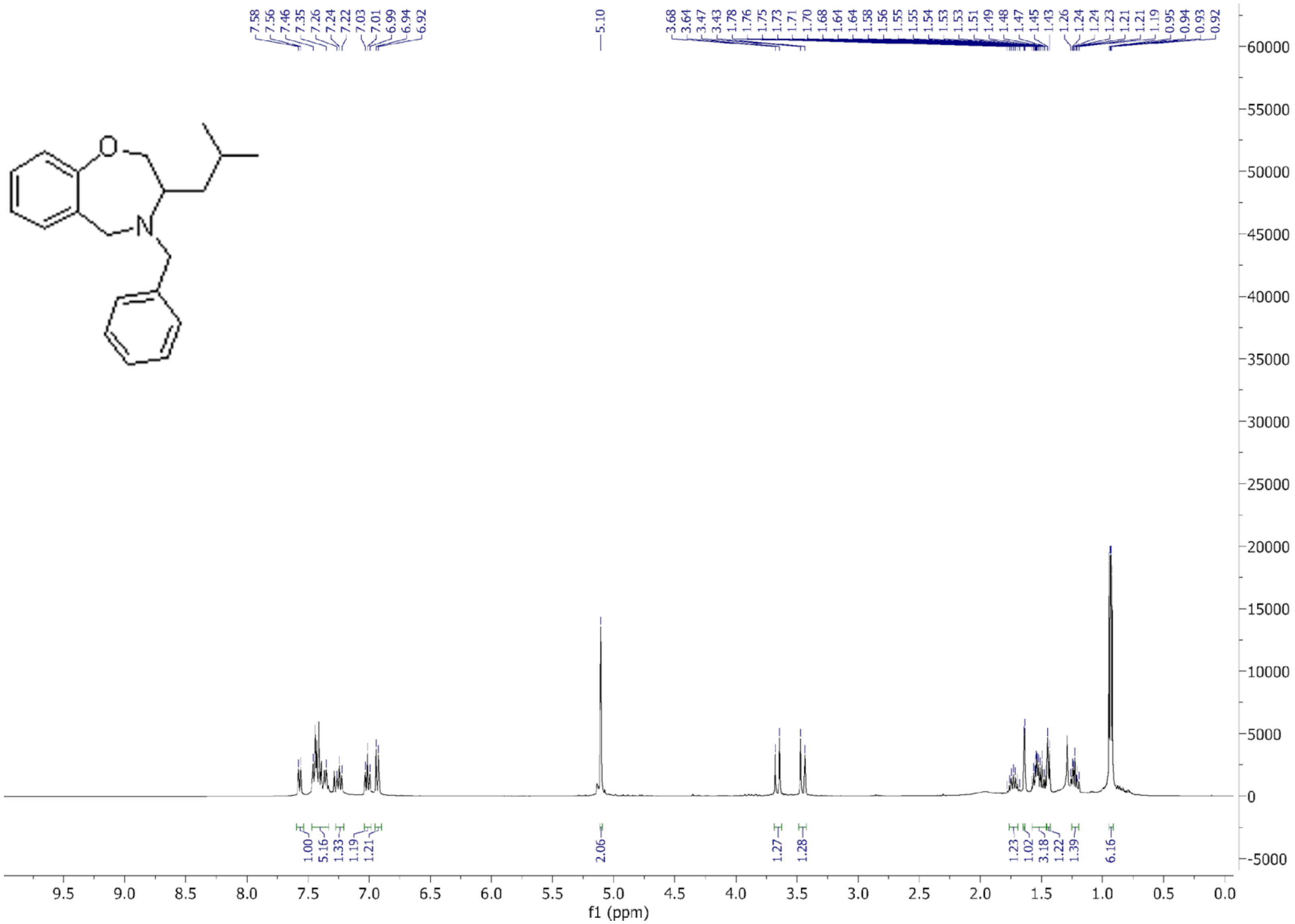

Figure S10: 1H NMR spectra of compound **14**

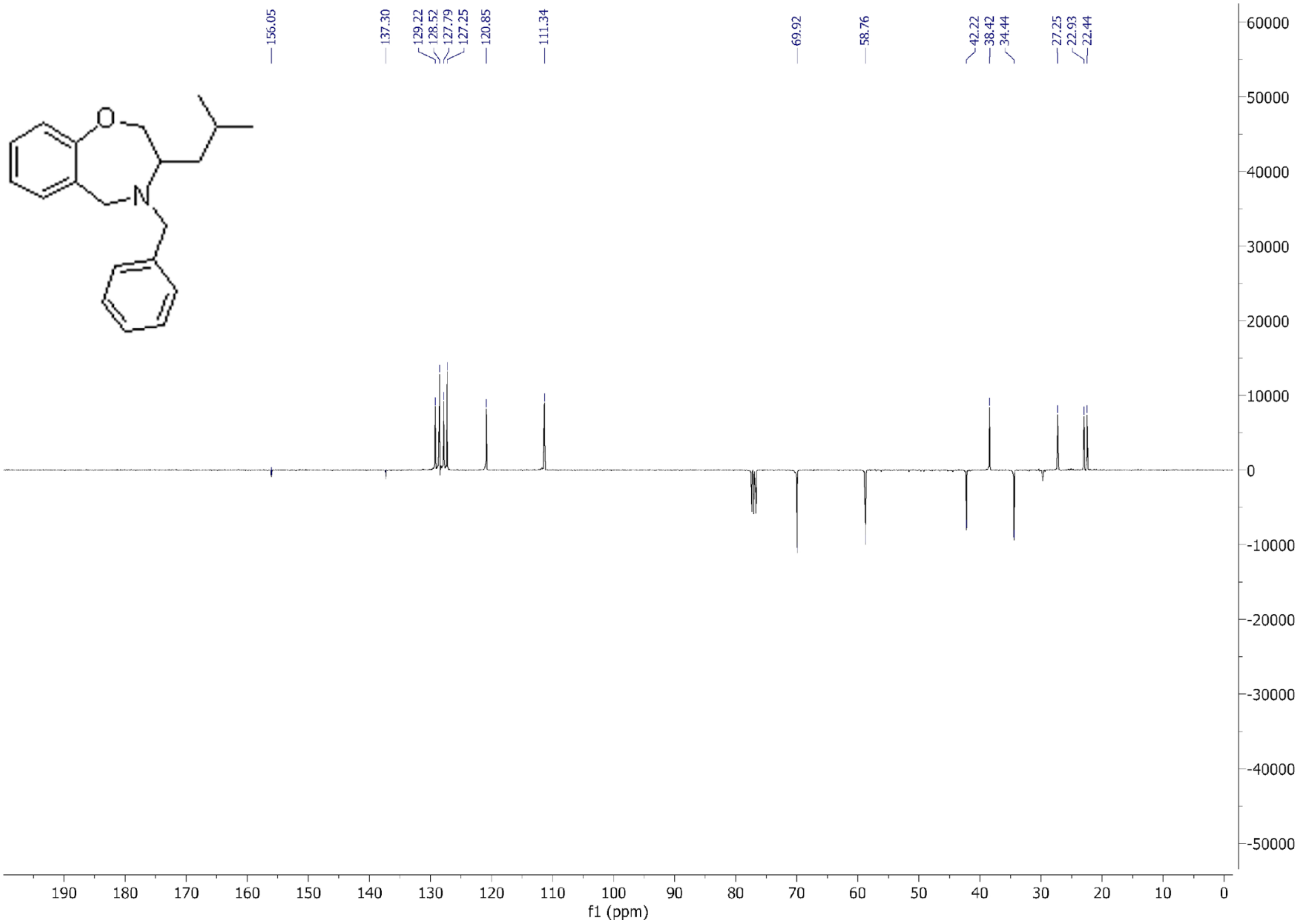

Figure S11: APTspectra of compound **14**

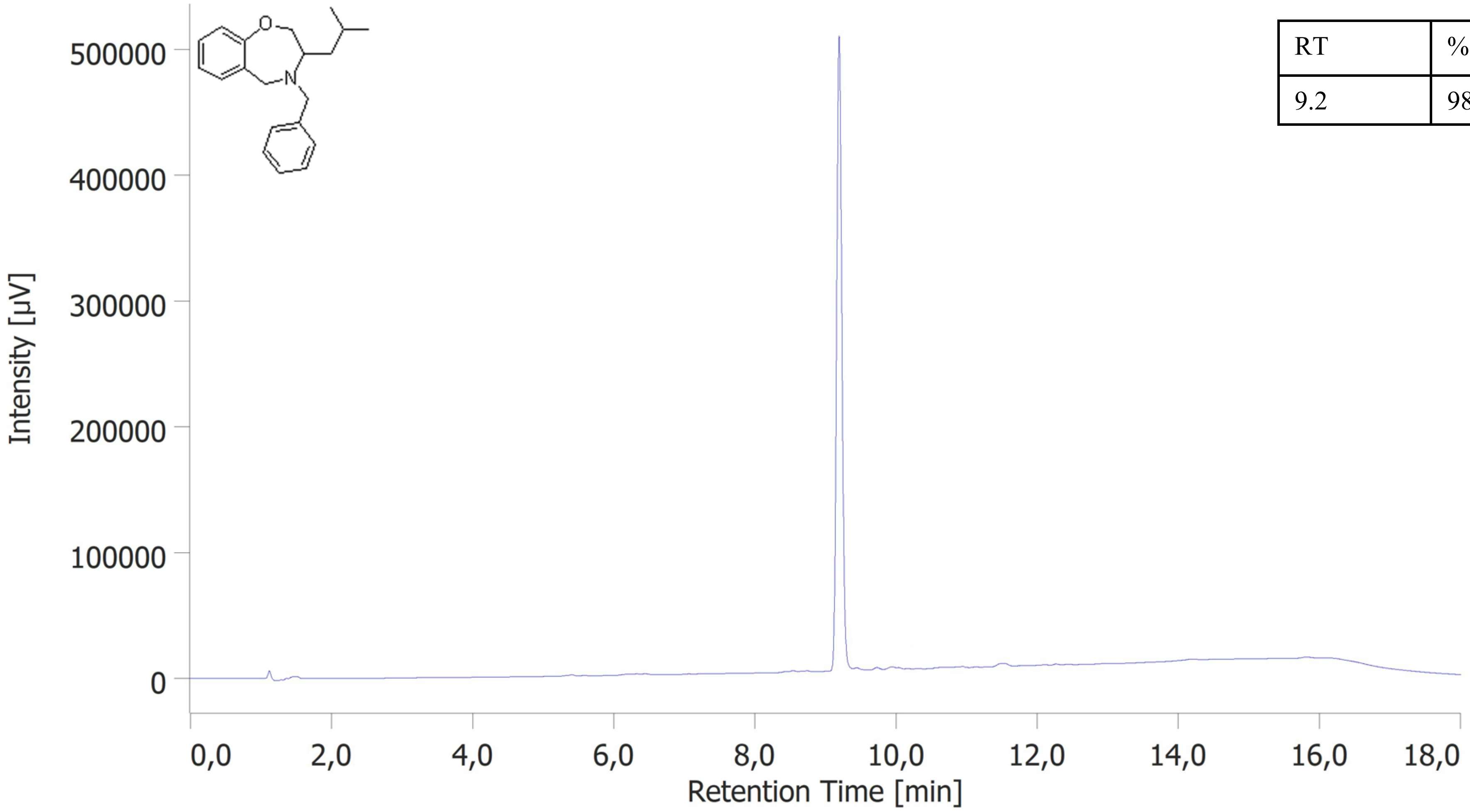

RT	% AREA
9.2	98

Figure S12: HPLC trace of compound **14**

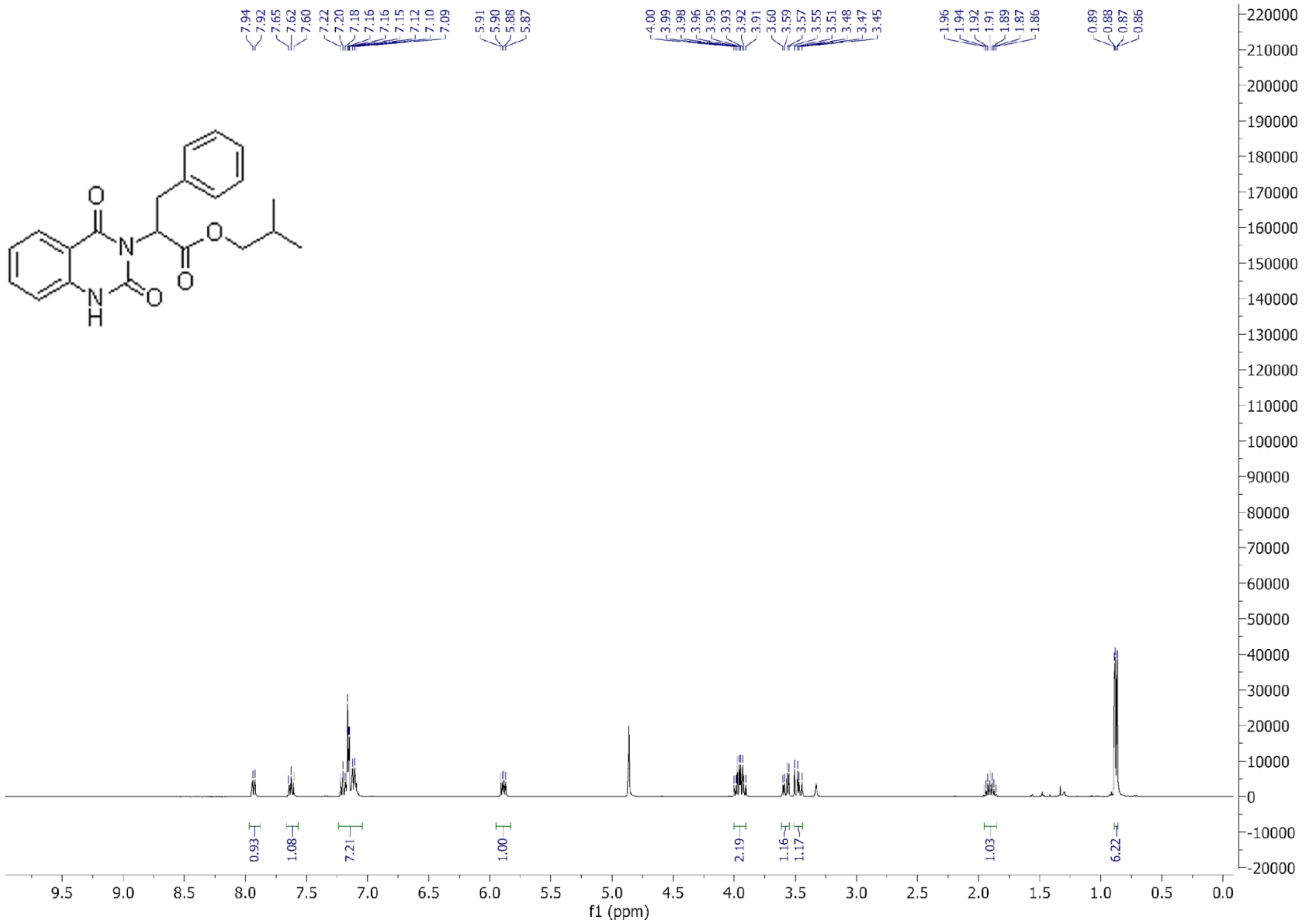

Figure S13: 1H NMR spectra of compound **22**

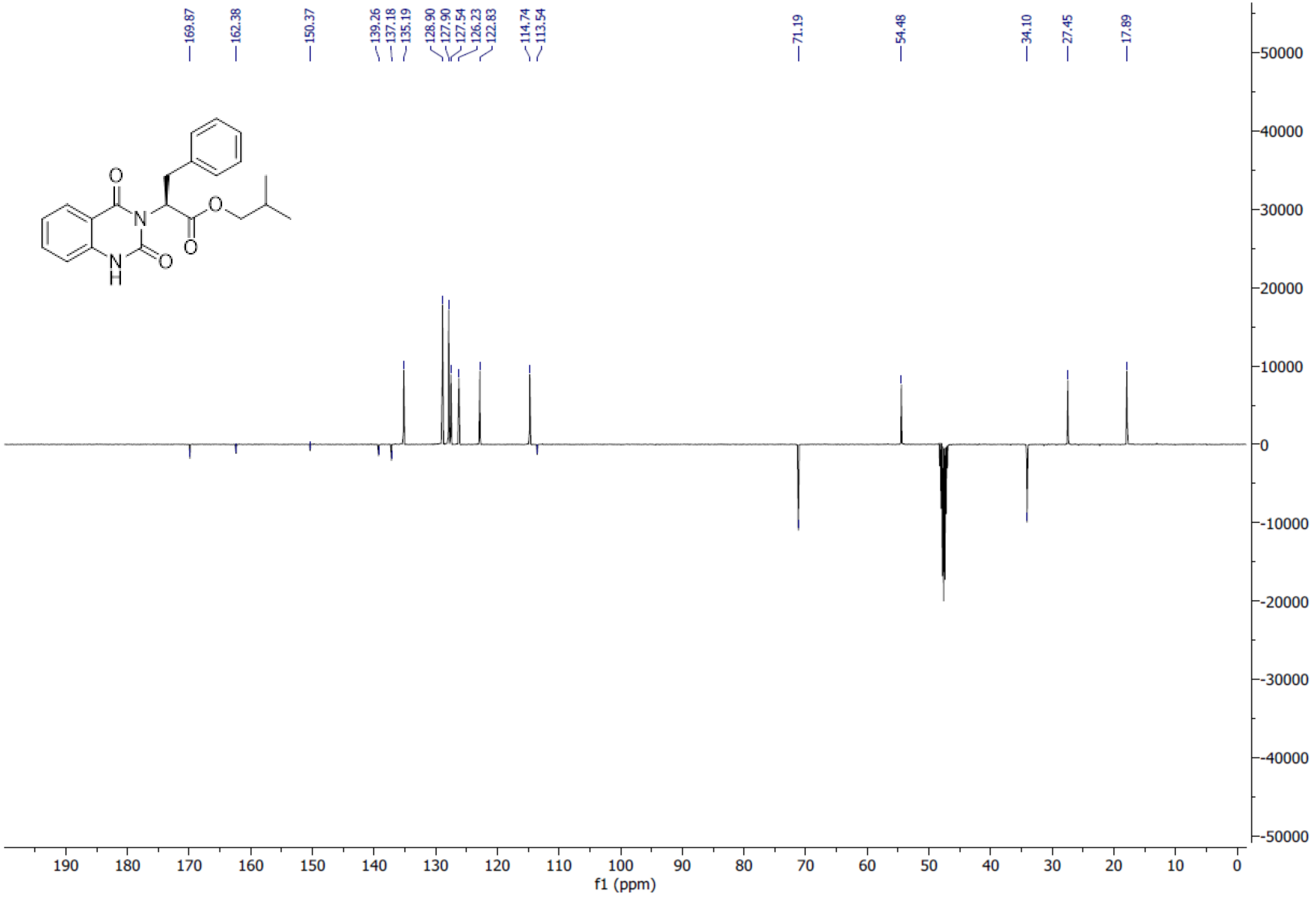

Figure S14: APTspectra of compound **22**

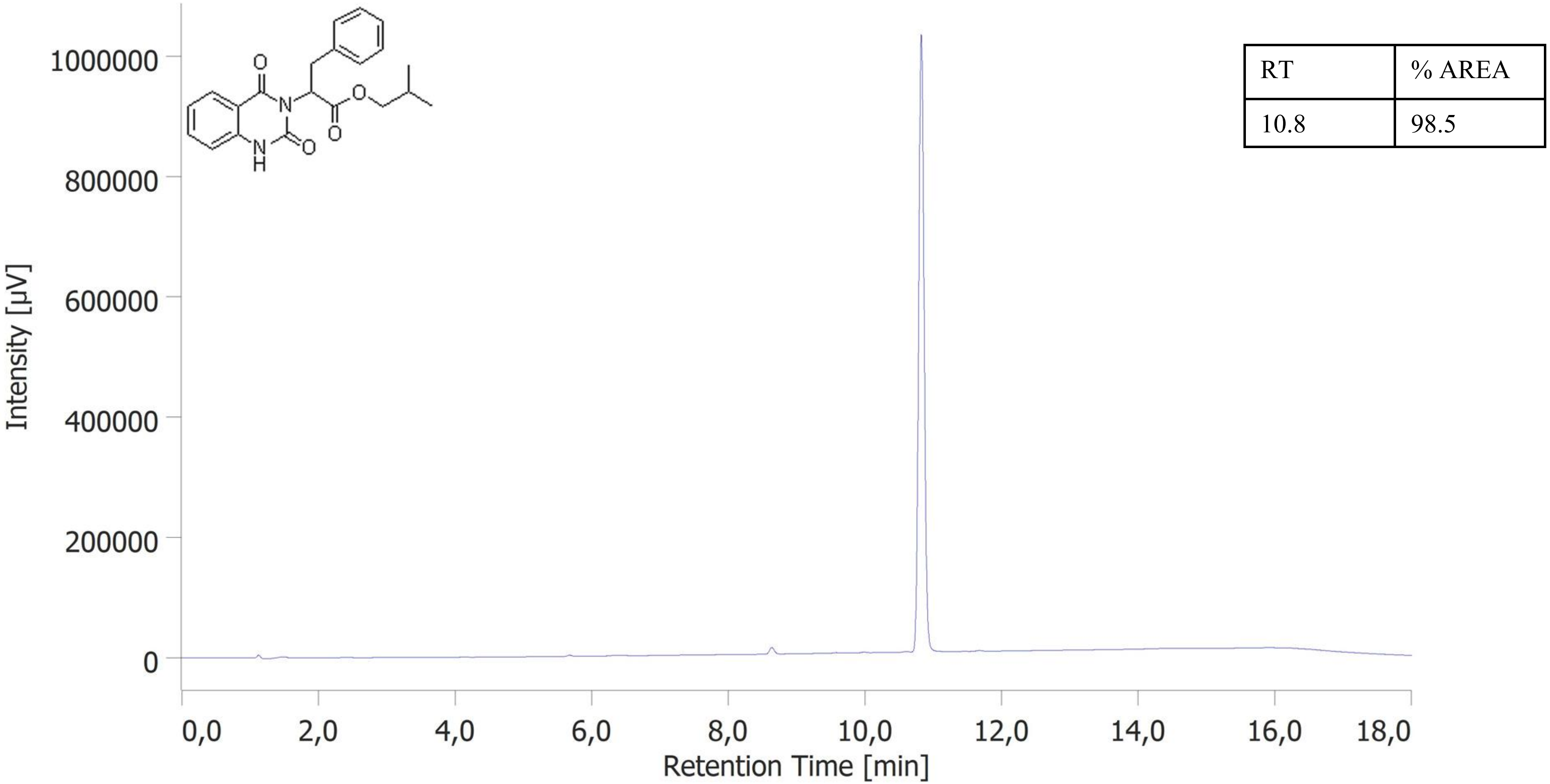

RT	% AREA
10.8	98.5

Figure S15: HPLC trace of compound **22**

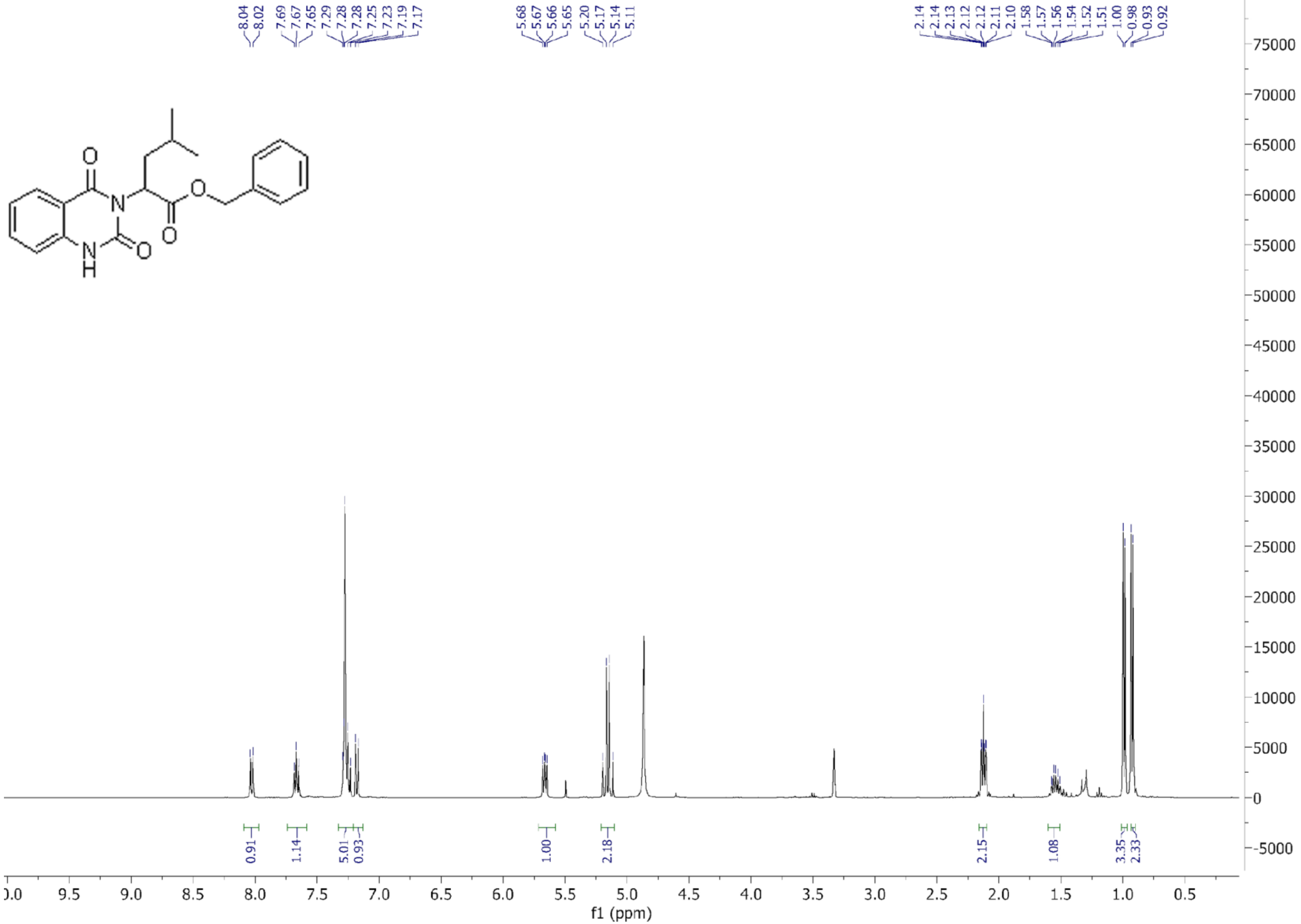

Figure S16: 1H NMR spectra of compound **23**

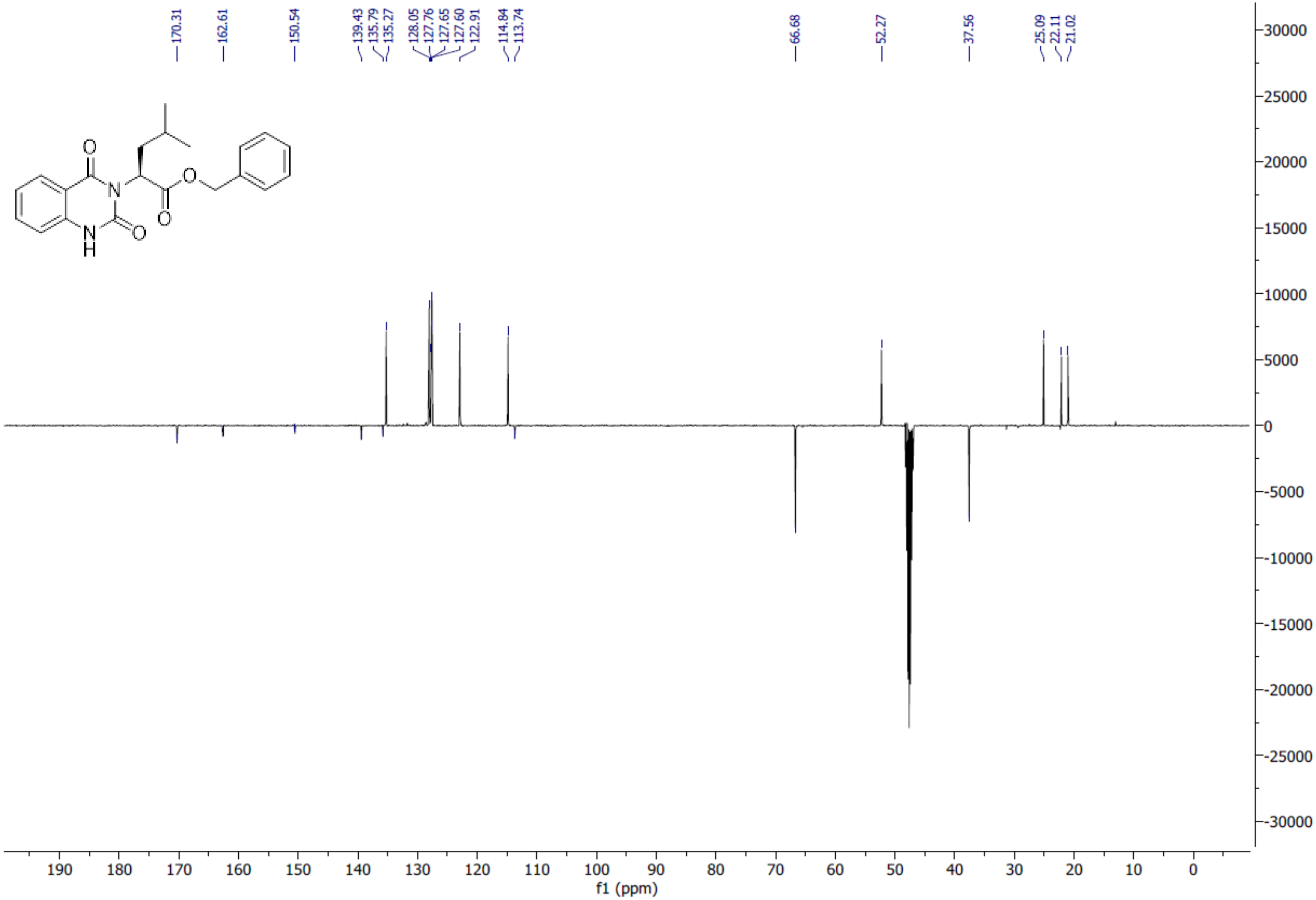

Figure S17: APTspectra of compound **23**

RT	% AREA
10.4	98.5

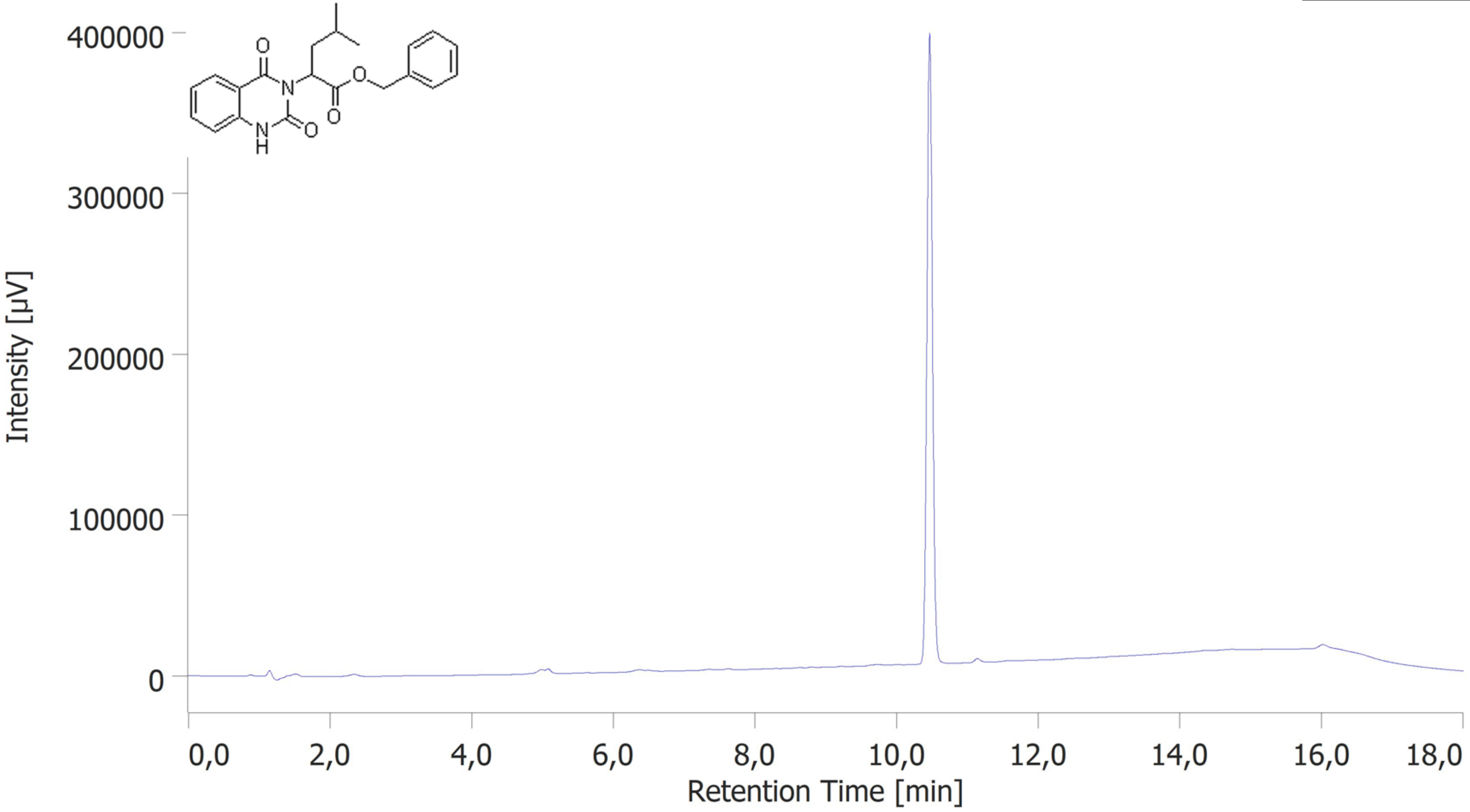

Figure S18: HPLC trace of compound **23**

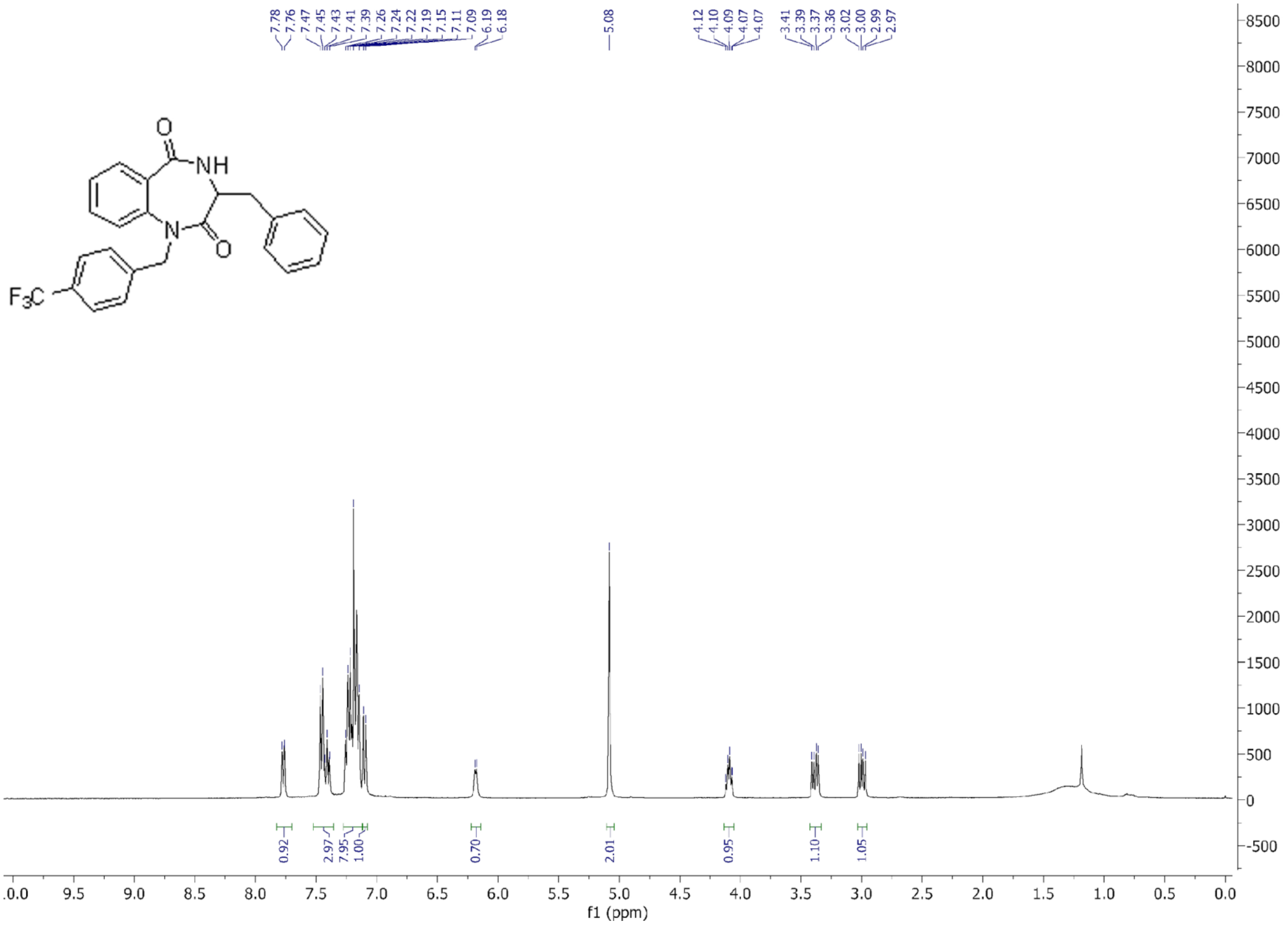

Figure S19: 1H NMR spectra of compound **28**

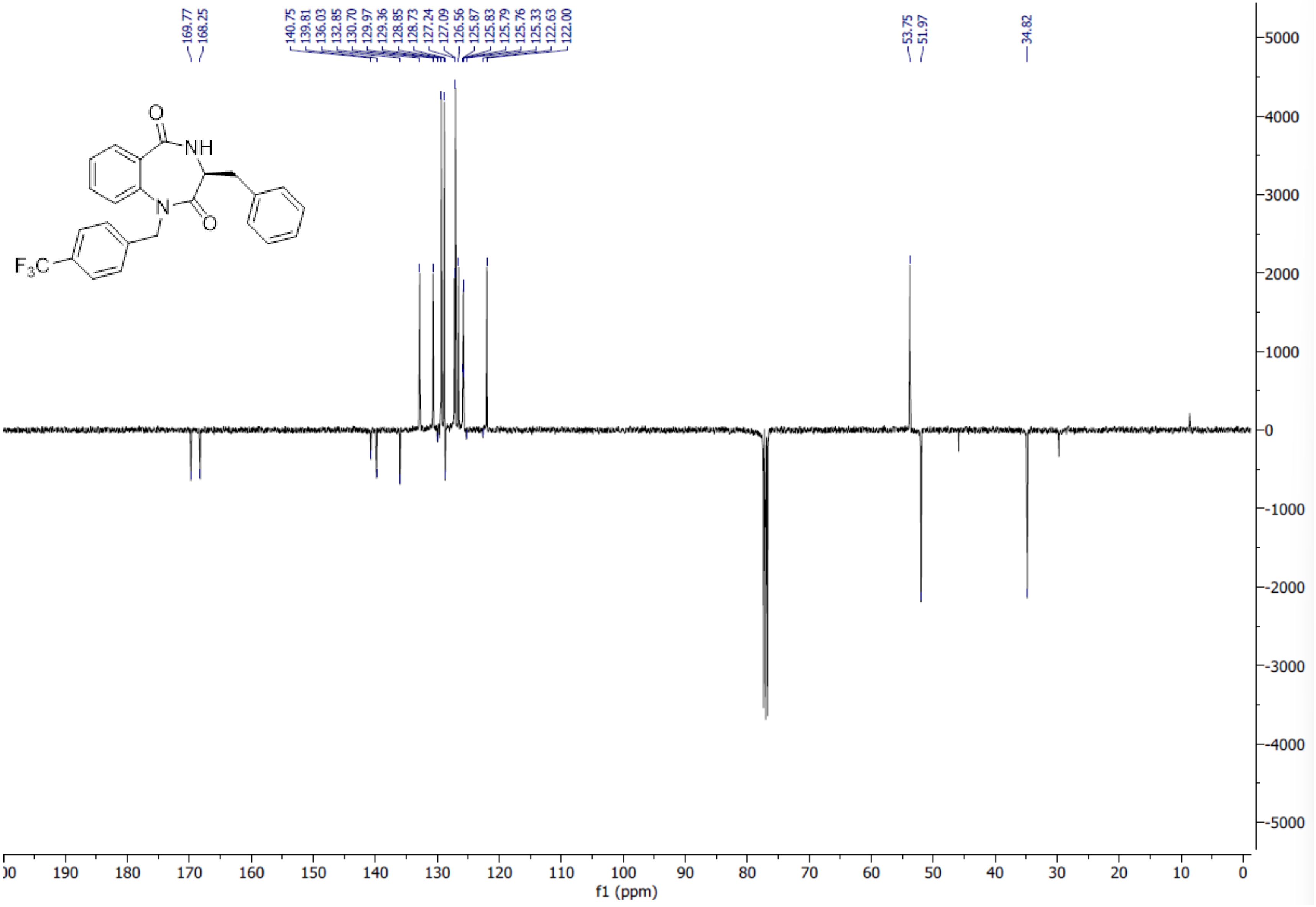

Figure S20: APT spectrum of compound **28**

RT	% AREA
10.2	99.5

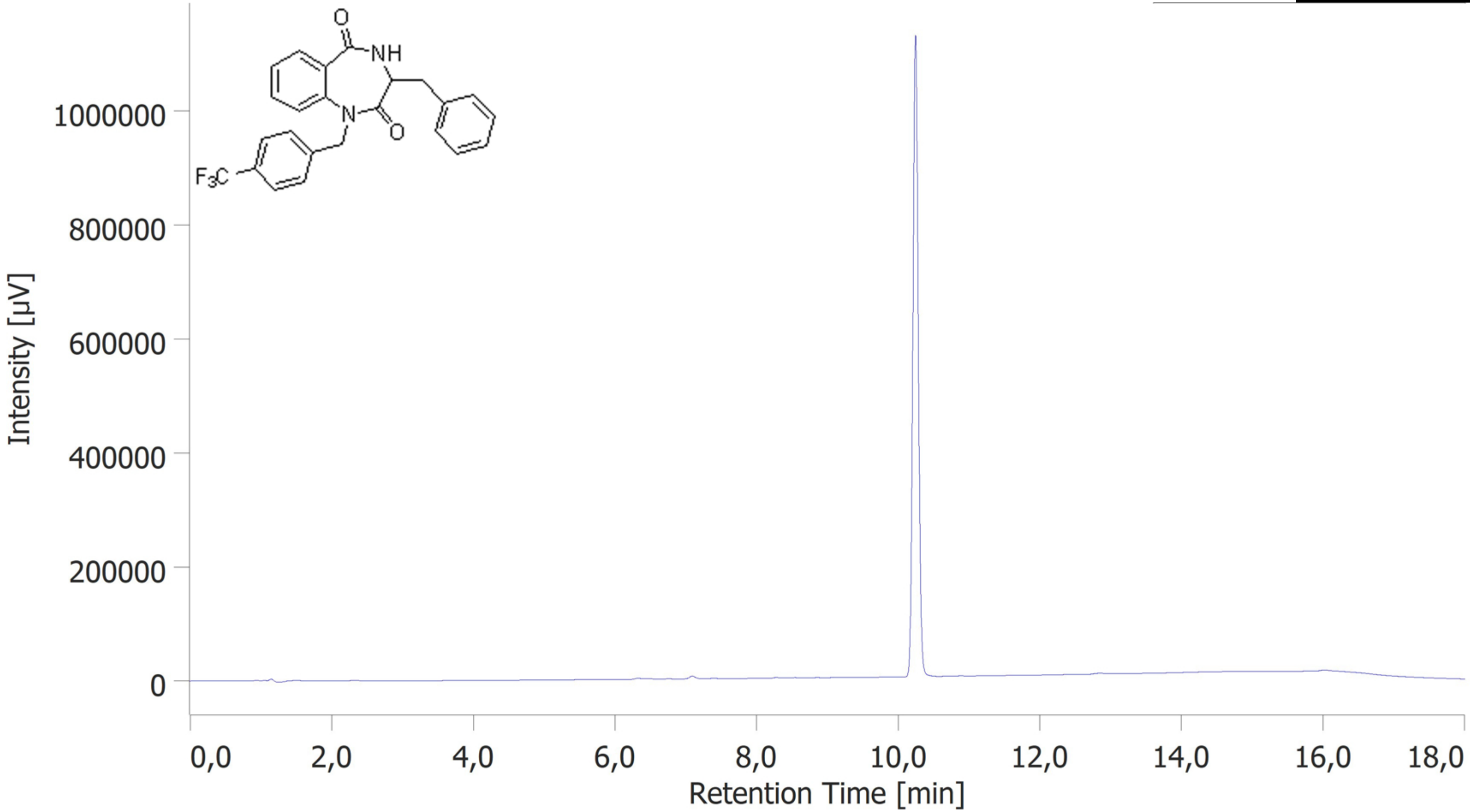

Figure S21: HPLC trace of compound **28**

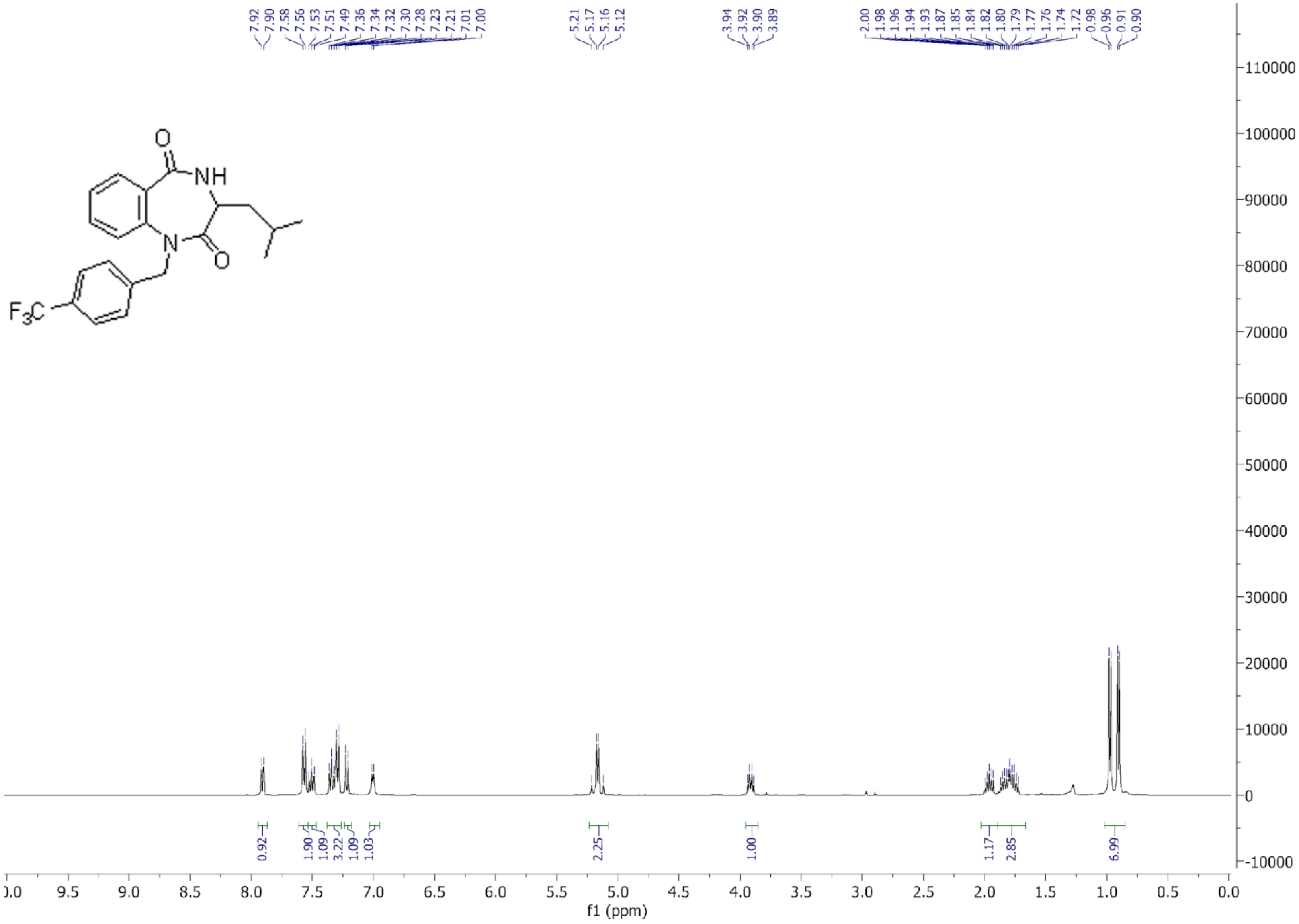

Figure S22: 1H NMR spectrum of compound **29**

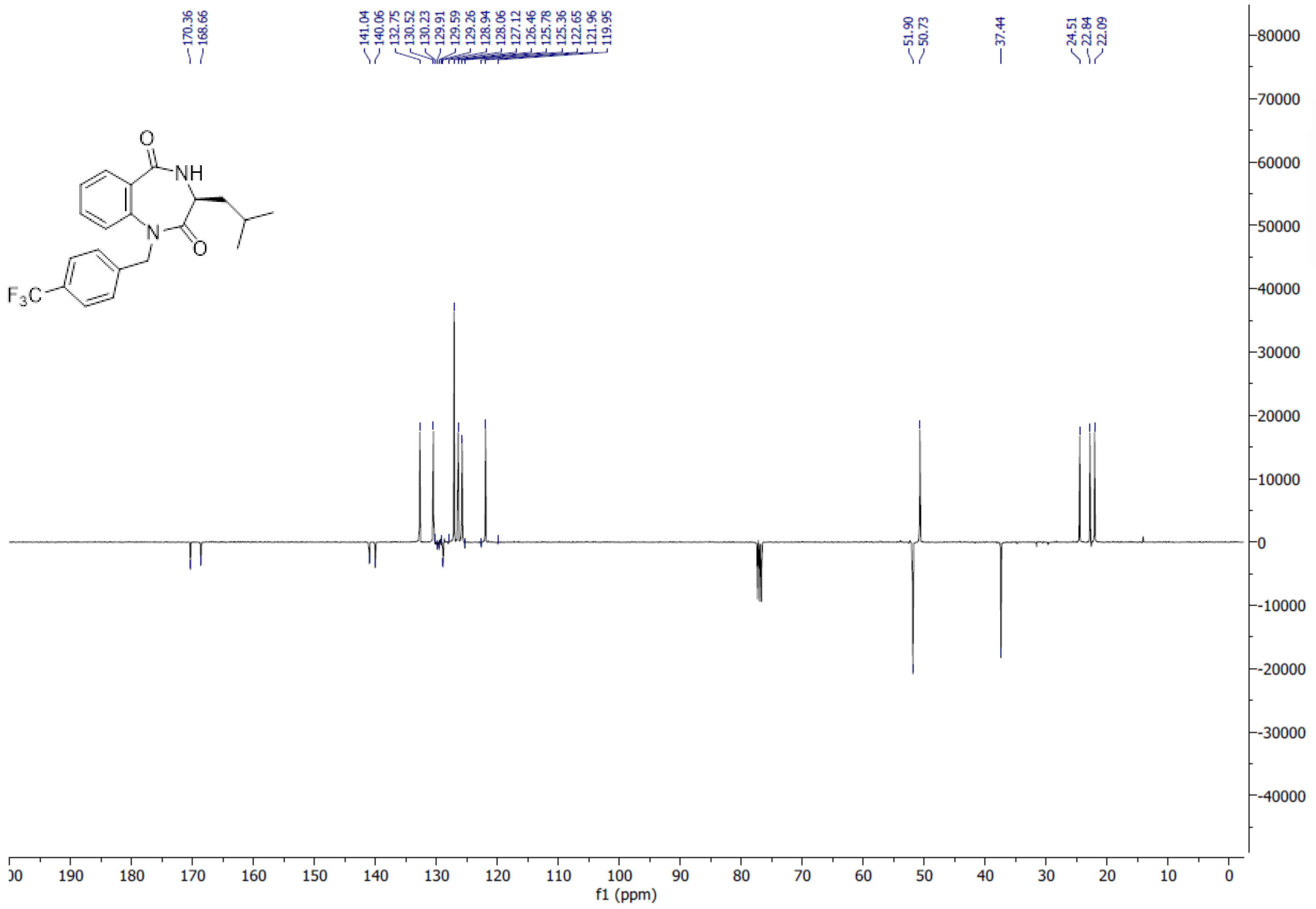

Figure S23: APT spectrum of compound **29**

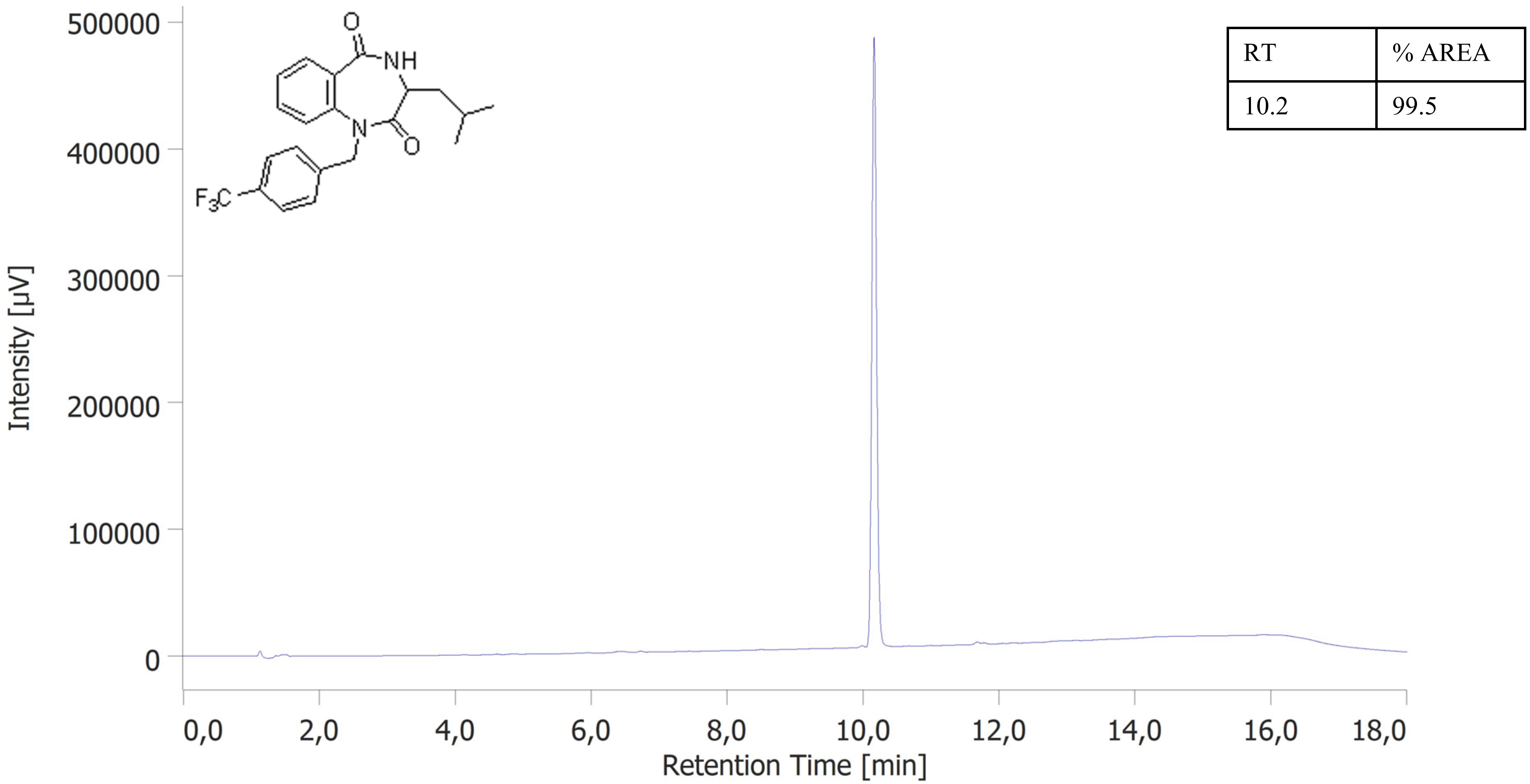

RT	% AREA
10.2	99.5

Figure S24: HPLC trace of compound **29**

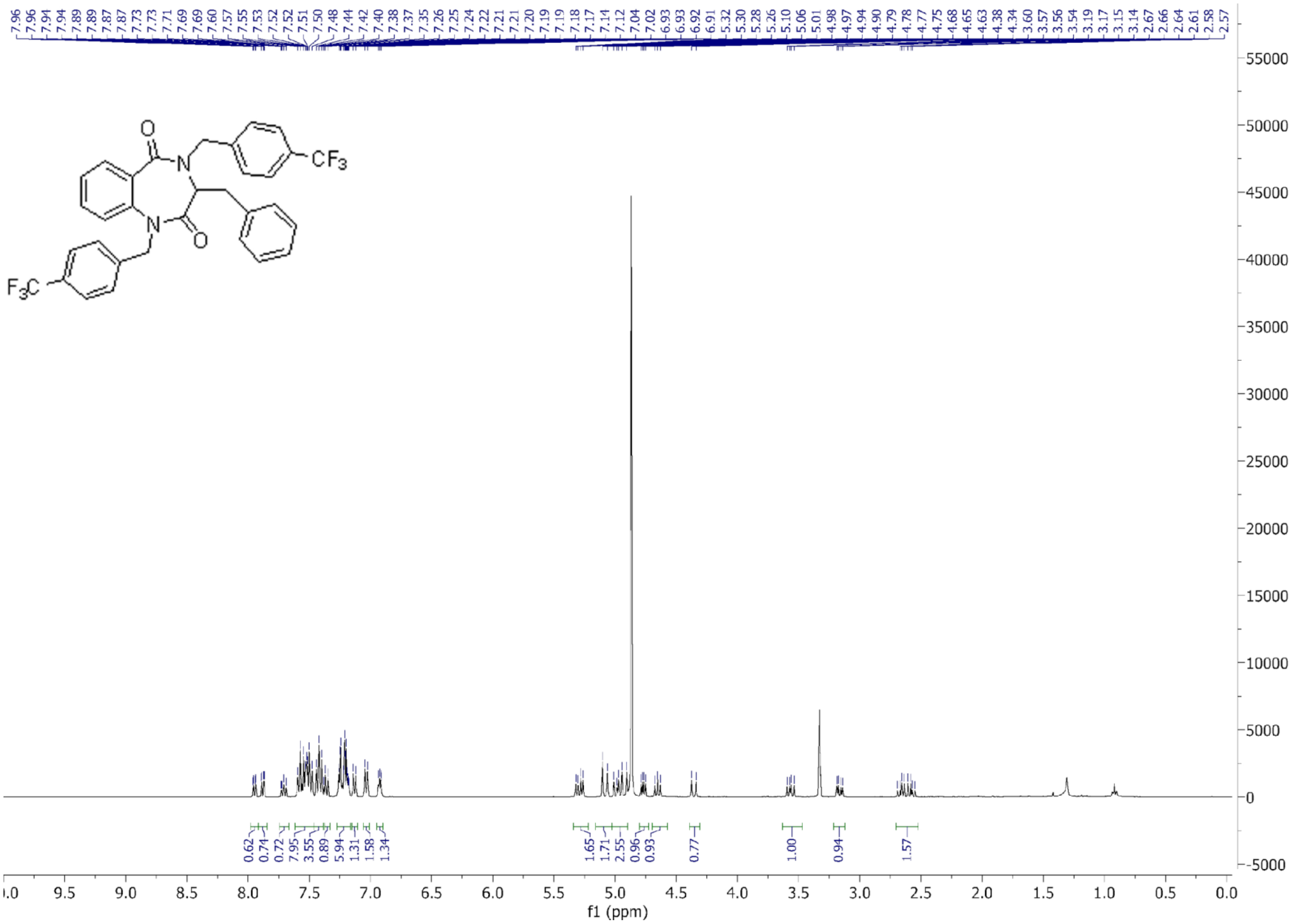

Figure S25: 1H NMR spectrum of compound **30**

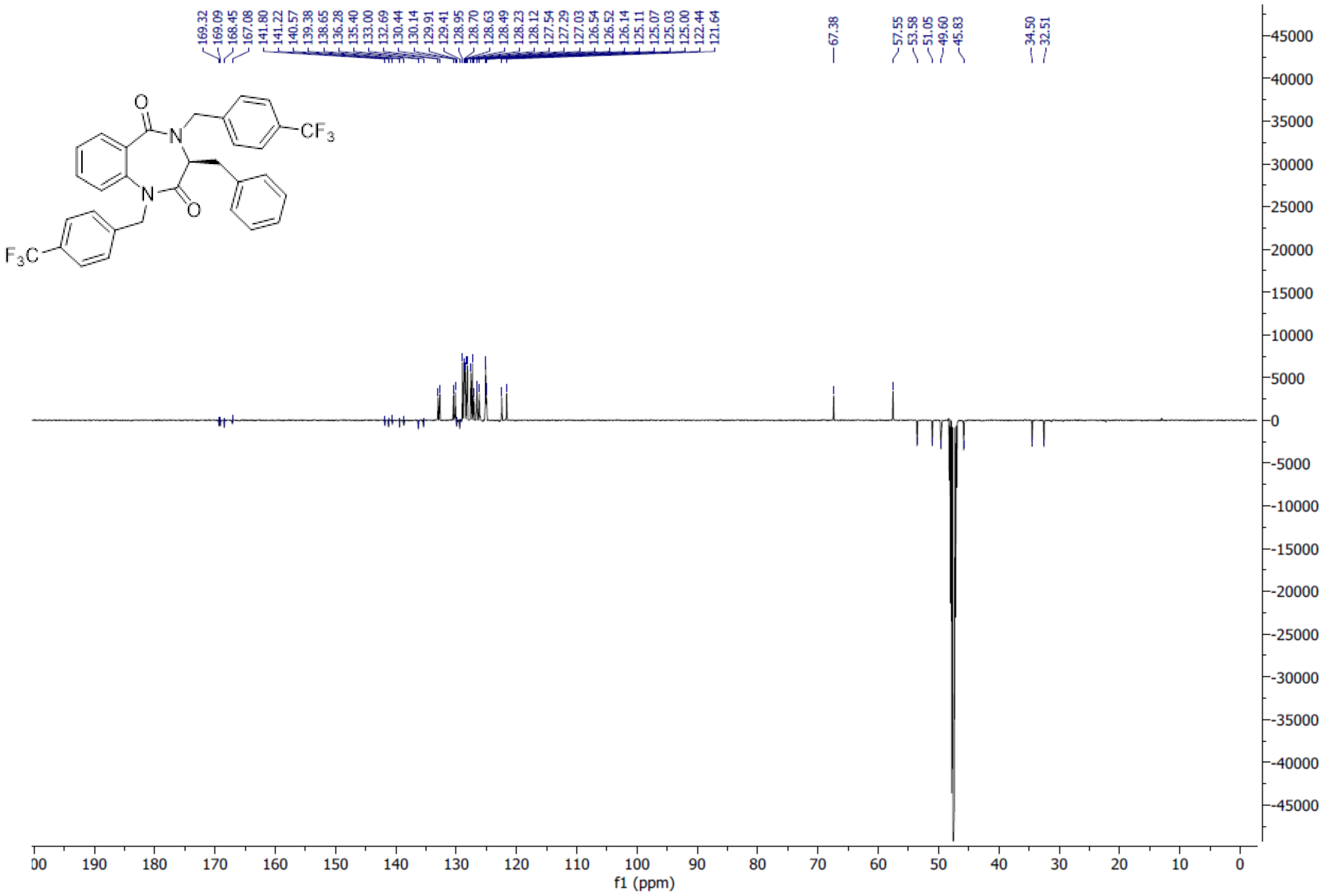

Figure S26: APT spectrum of compound **30**

RT	% AREA
12.4	98

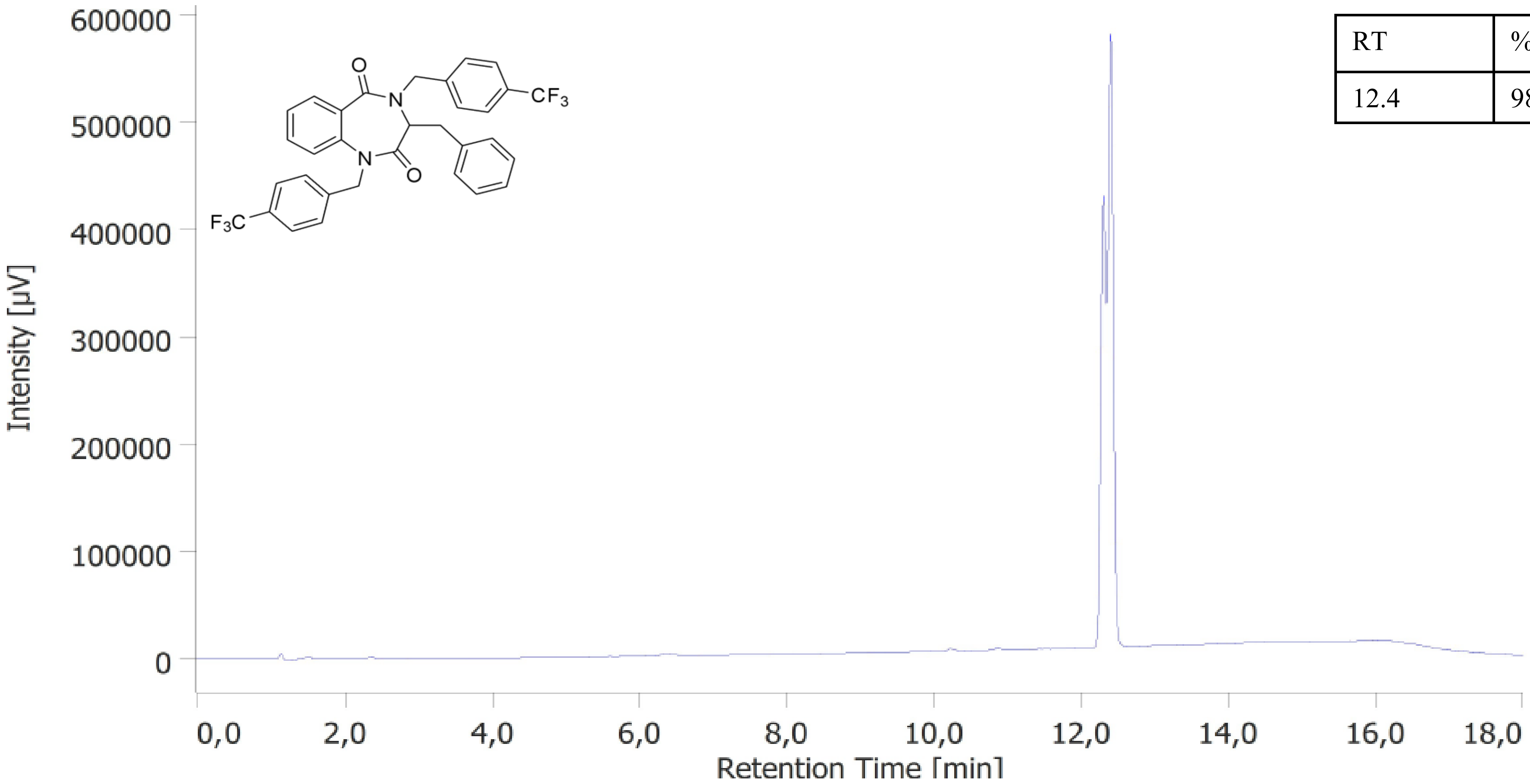

Figure S27: HPLC trace of compound **30**

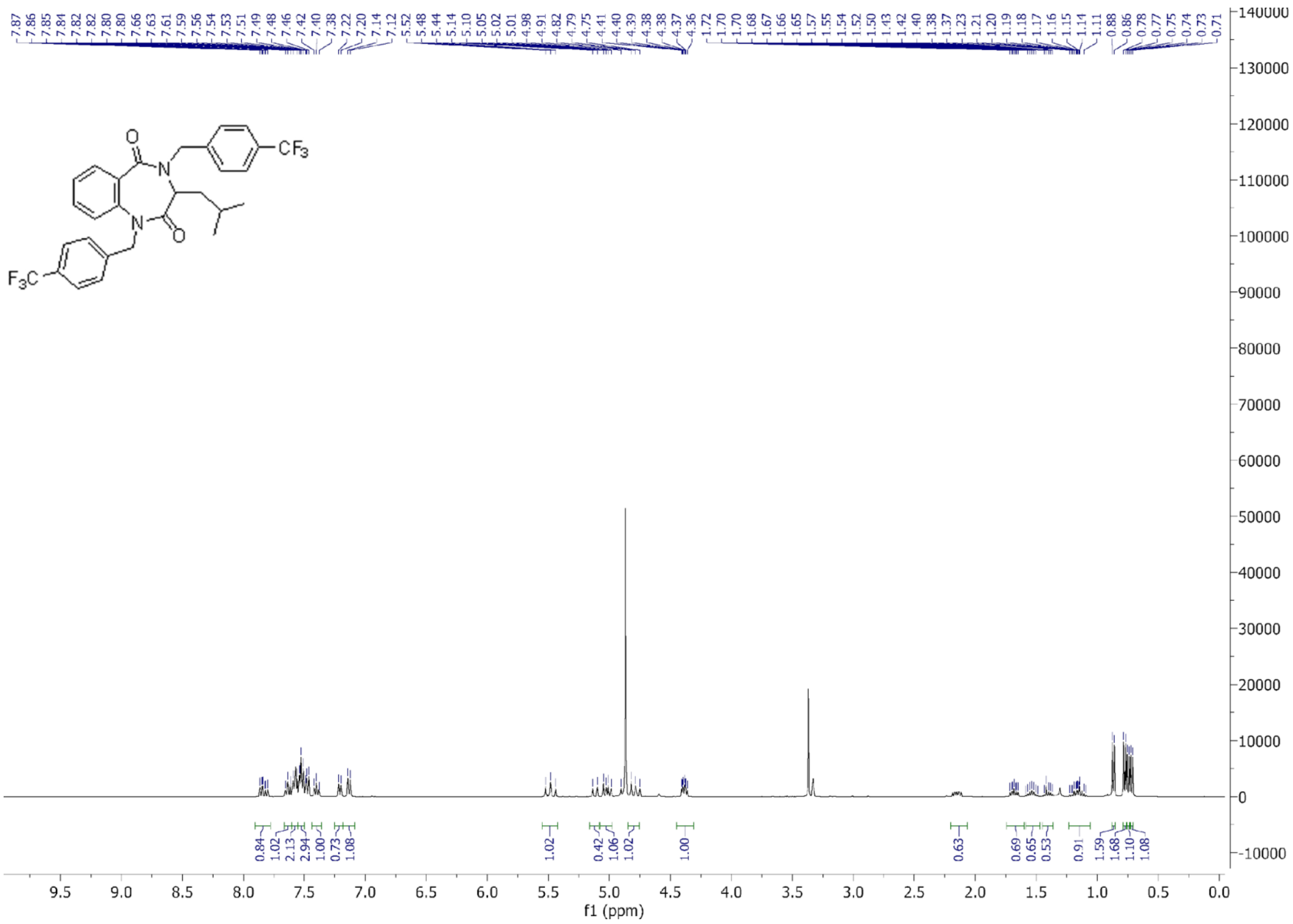

Figure S28: 1H NMR spectrum of compound **31**

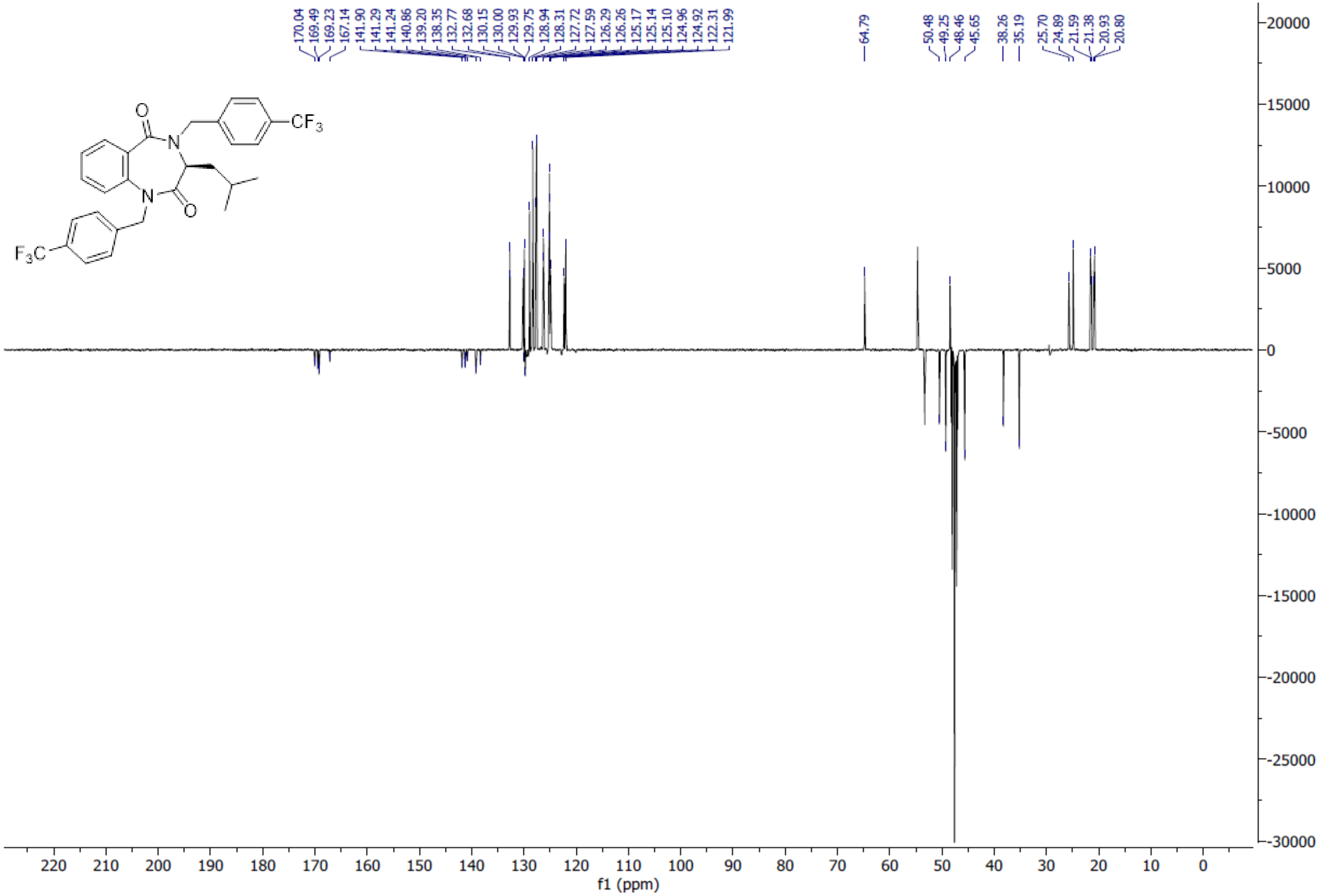

Figure S29: APT spectrum of compound **31**

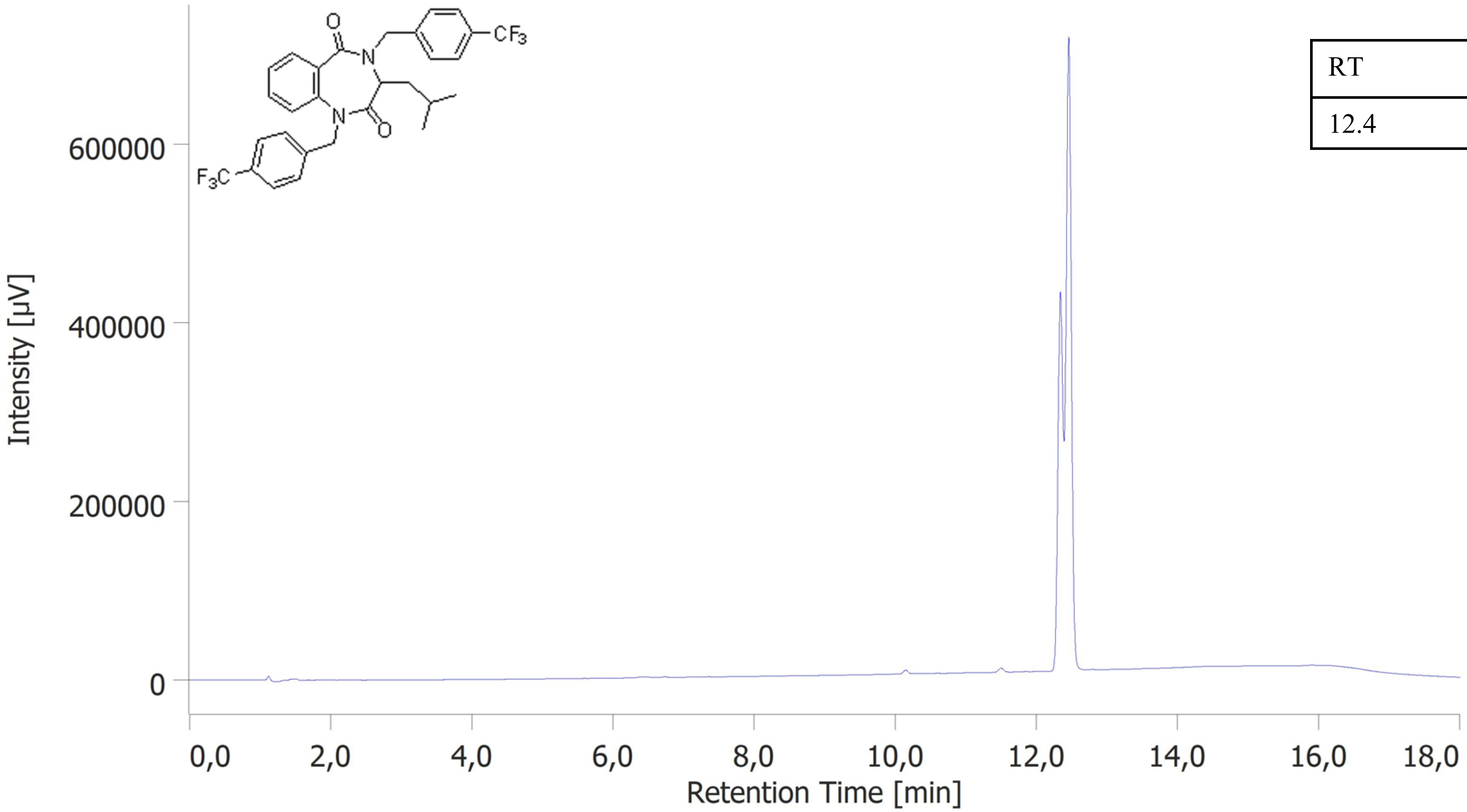

RT	% AREA
12.4	99.5

Figure S30: HPLC trace of compound **31**

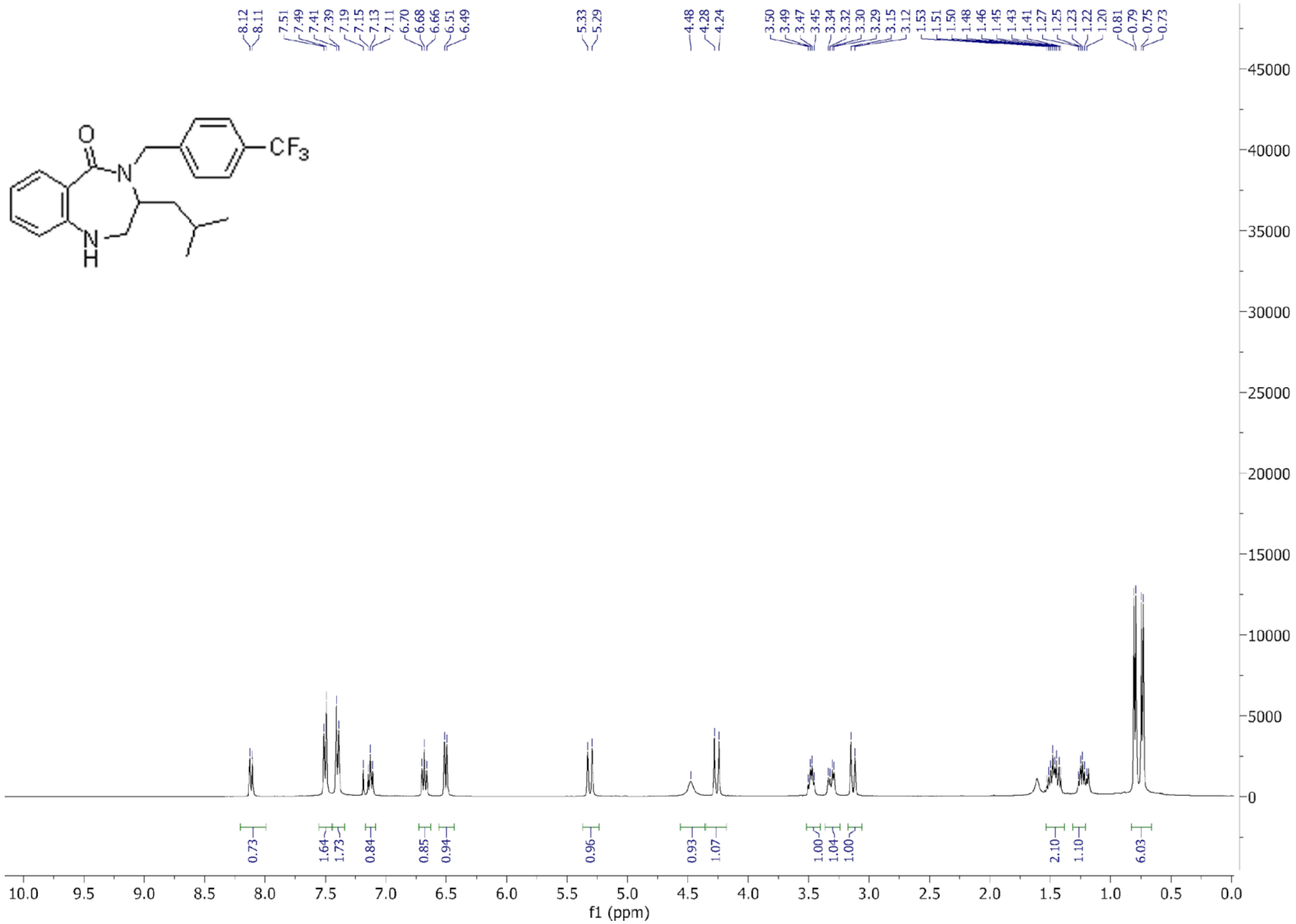

Figure S31: 1H NMR spectrum of compound **33**

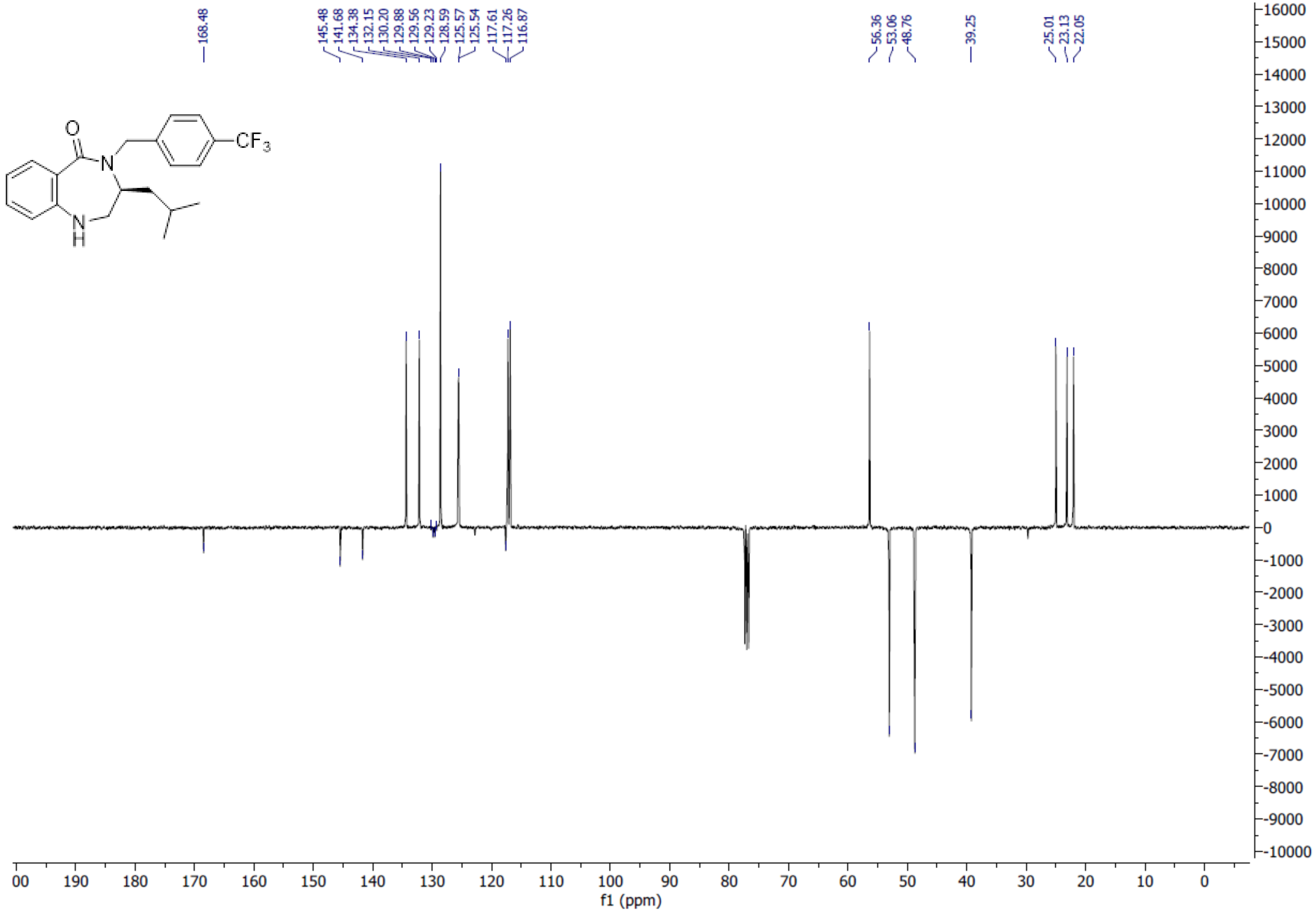

Figure S32: APT spectrum of compound **33**

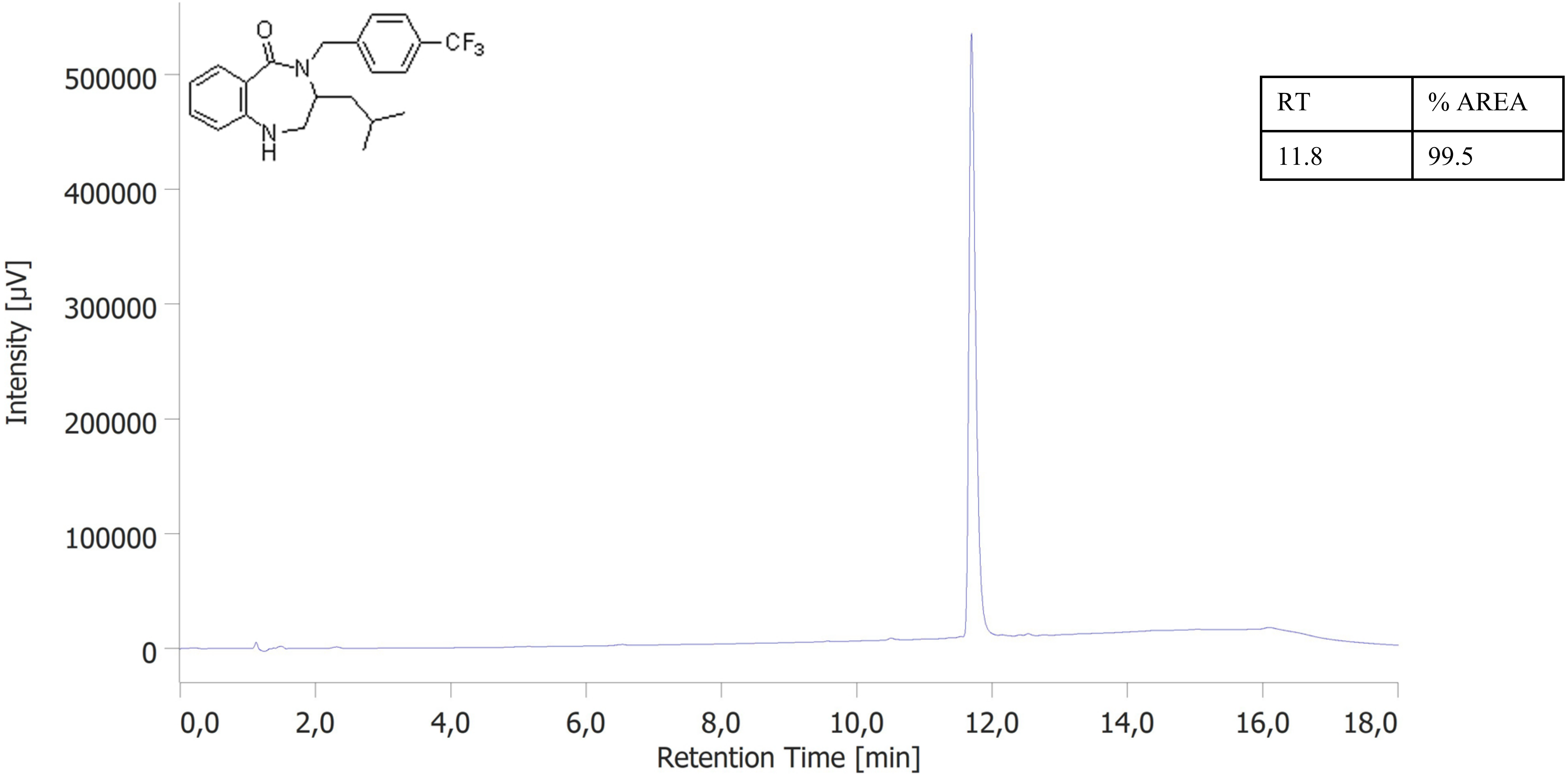

RT	% AREA
11.8	99.5

Figure S33: HPLC trace of compound **33**

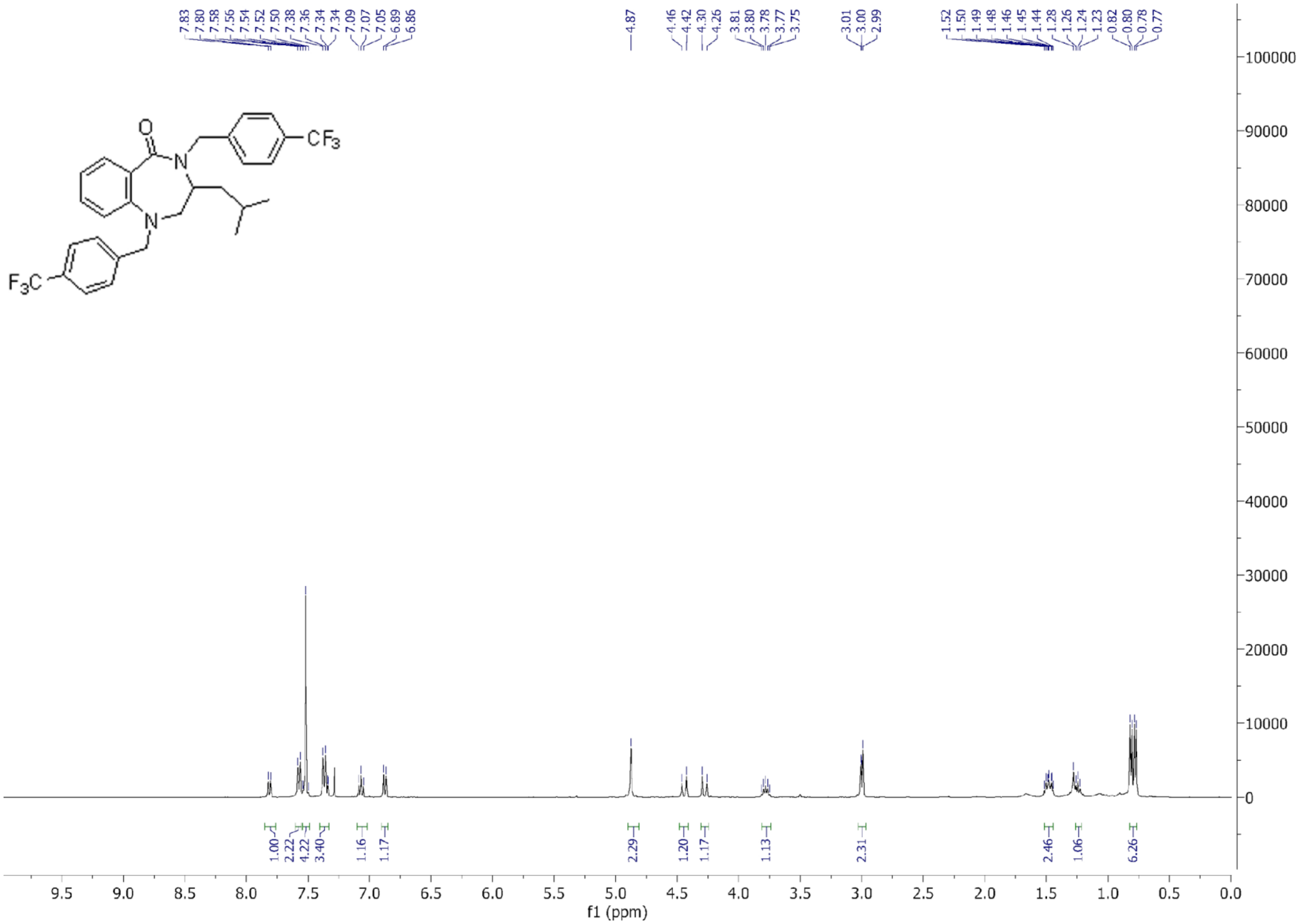

Figure S34: 1H NMR spectrum of compound **34**

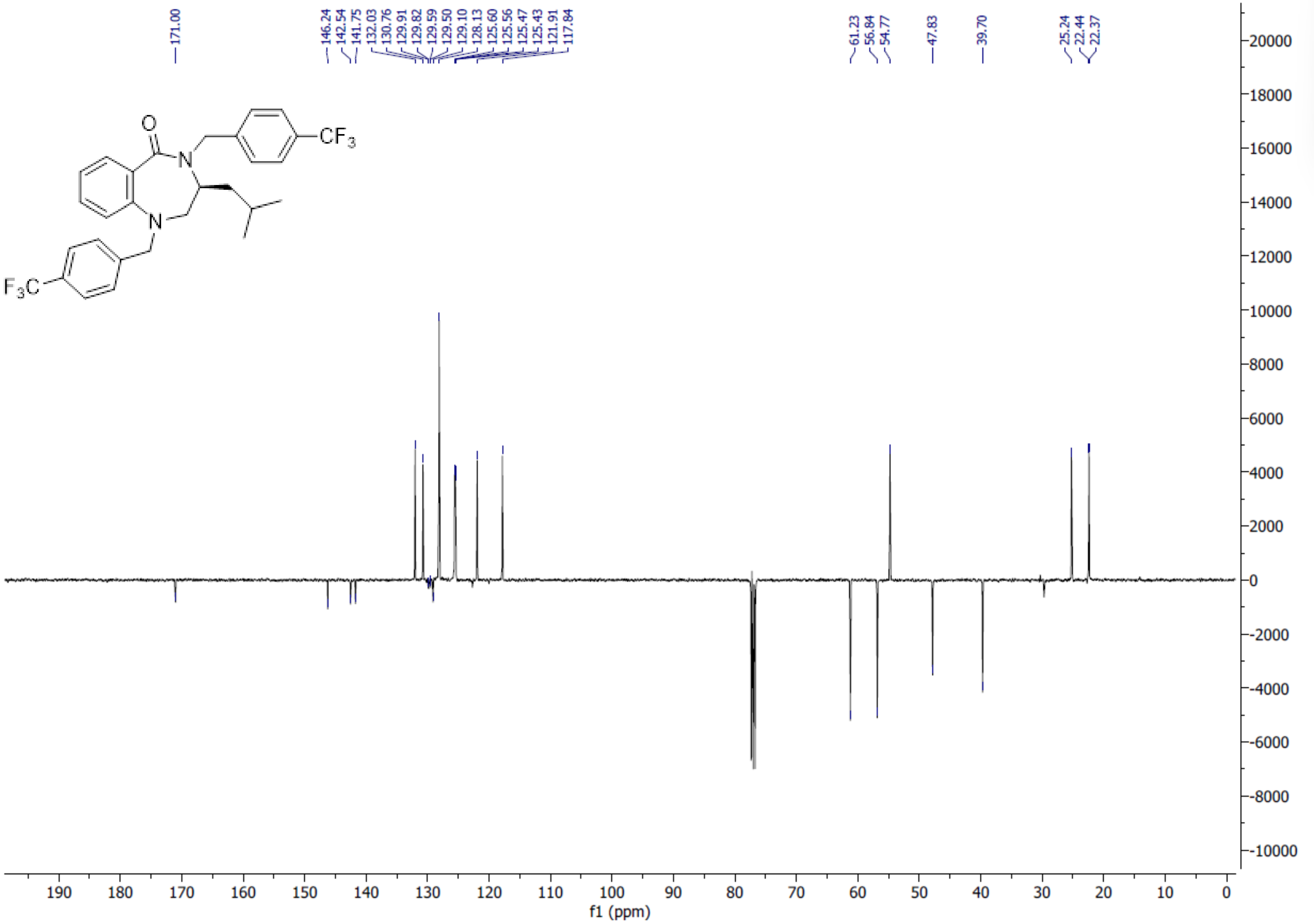

Figure S35: APT spectrum of compound **34**

RT	% AREA
13.3	98

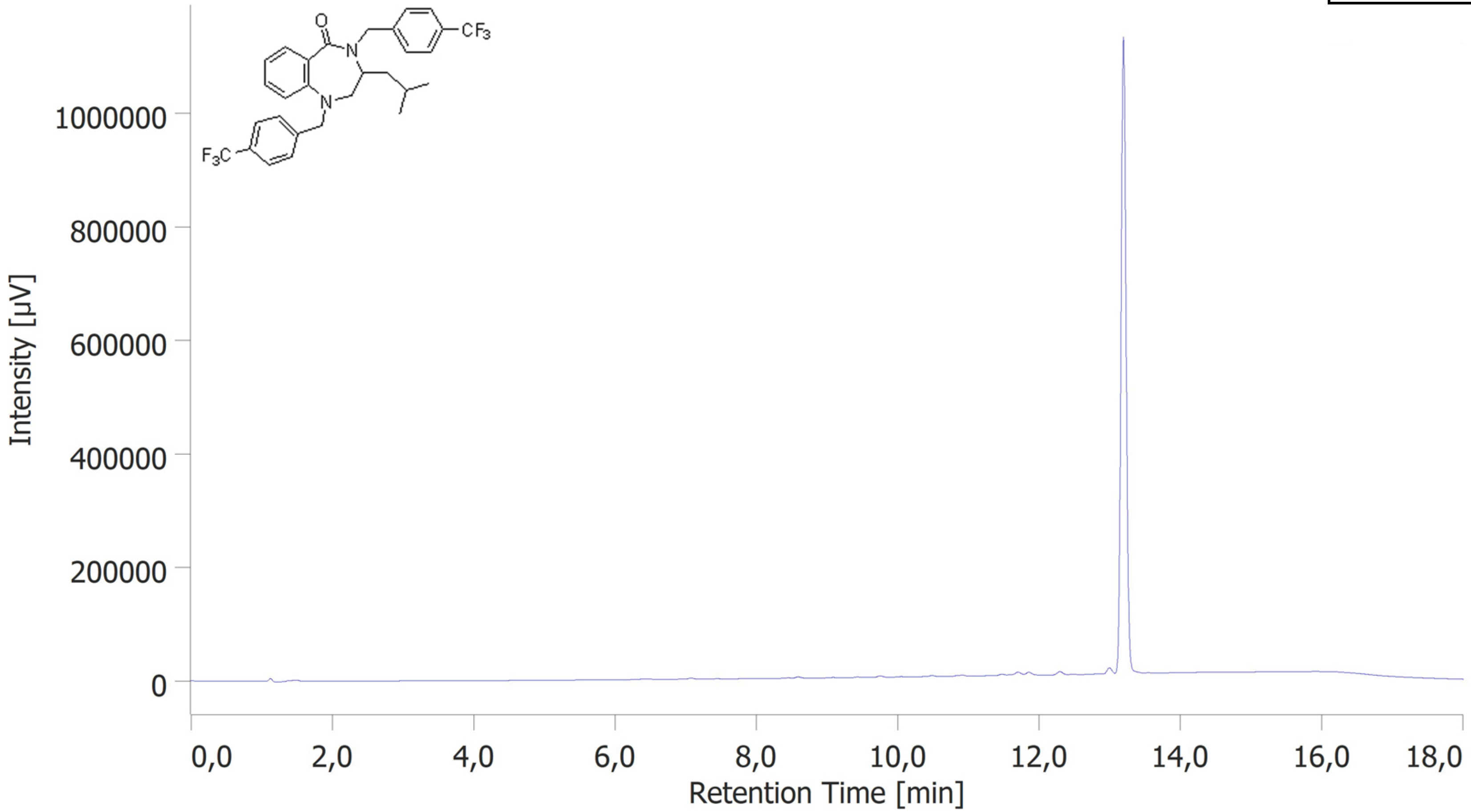

Figure S36: HPLC trace of compound **34**

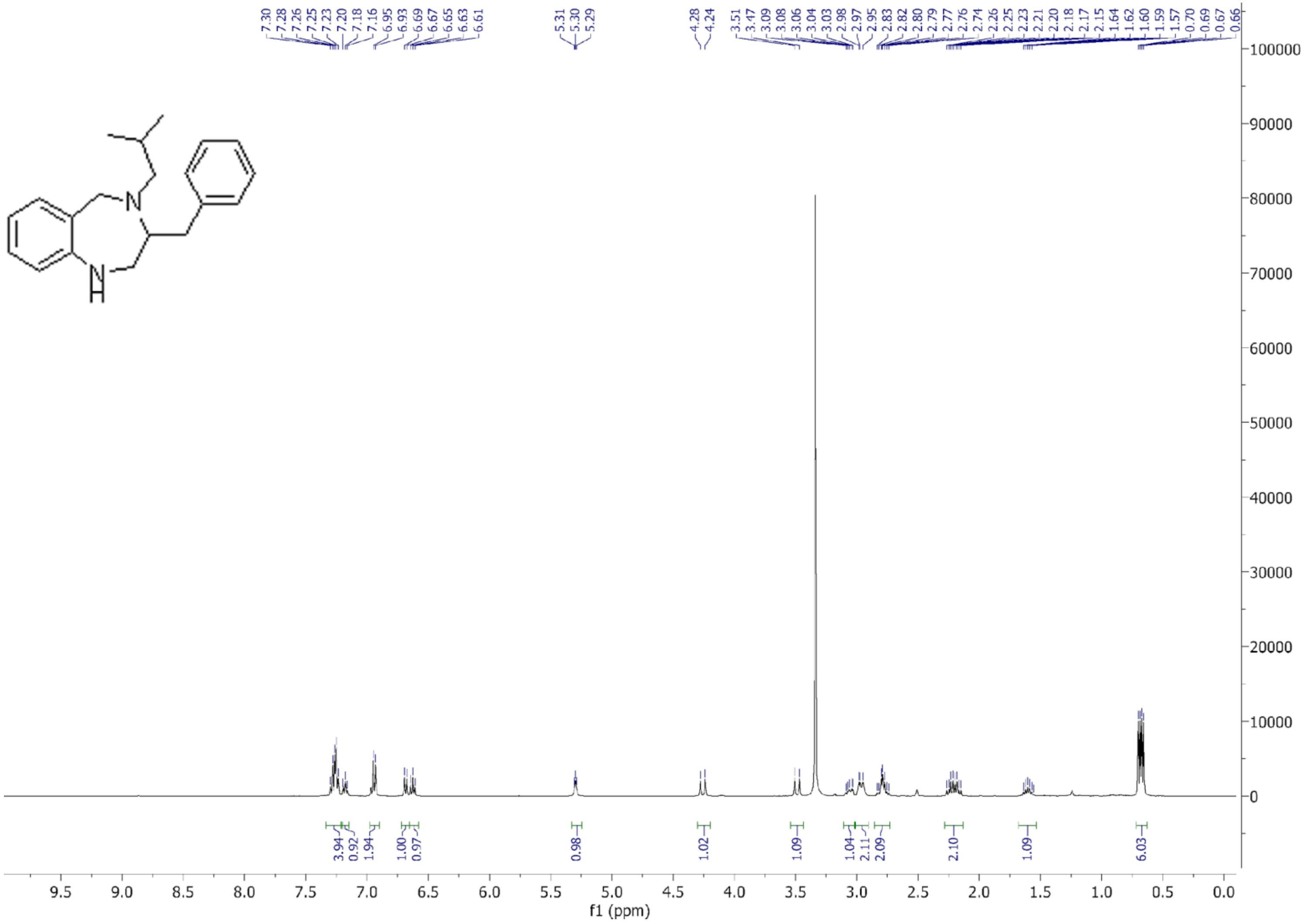

Figure S37: 1H NMR spectrum of compound **37**

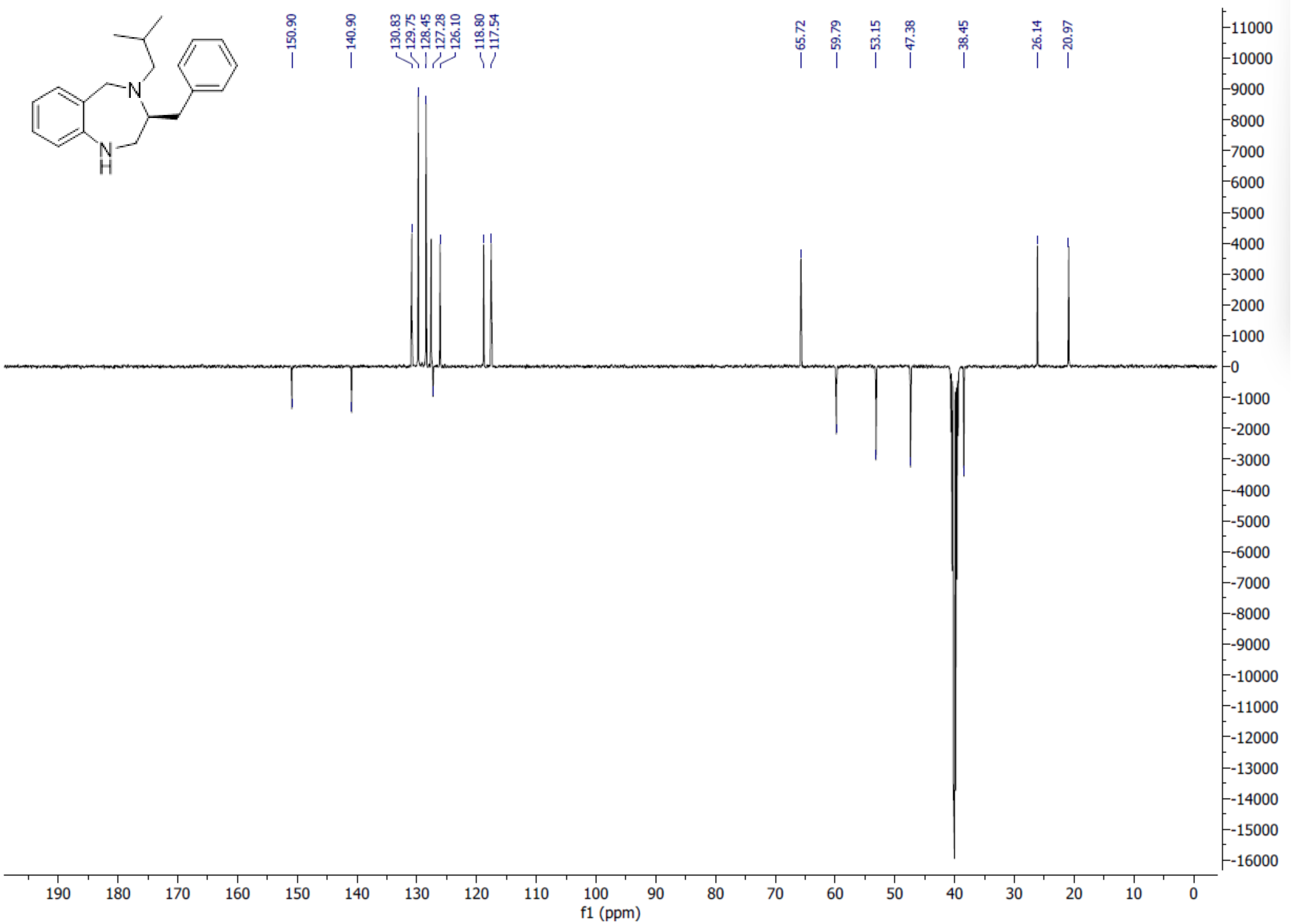

Figure S38: APT spectrum of compound **37**

RT	% AREA
7.8	97.5

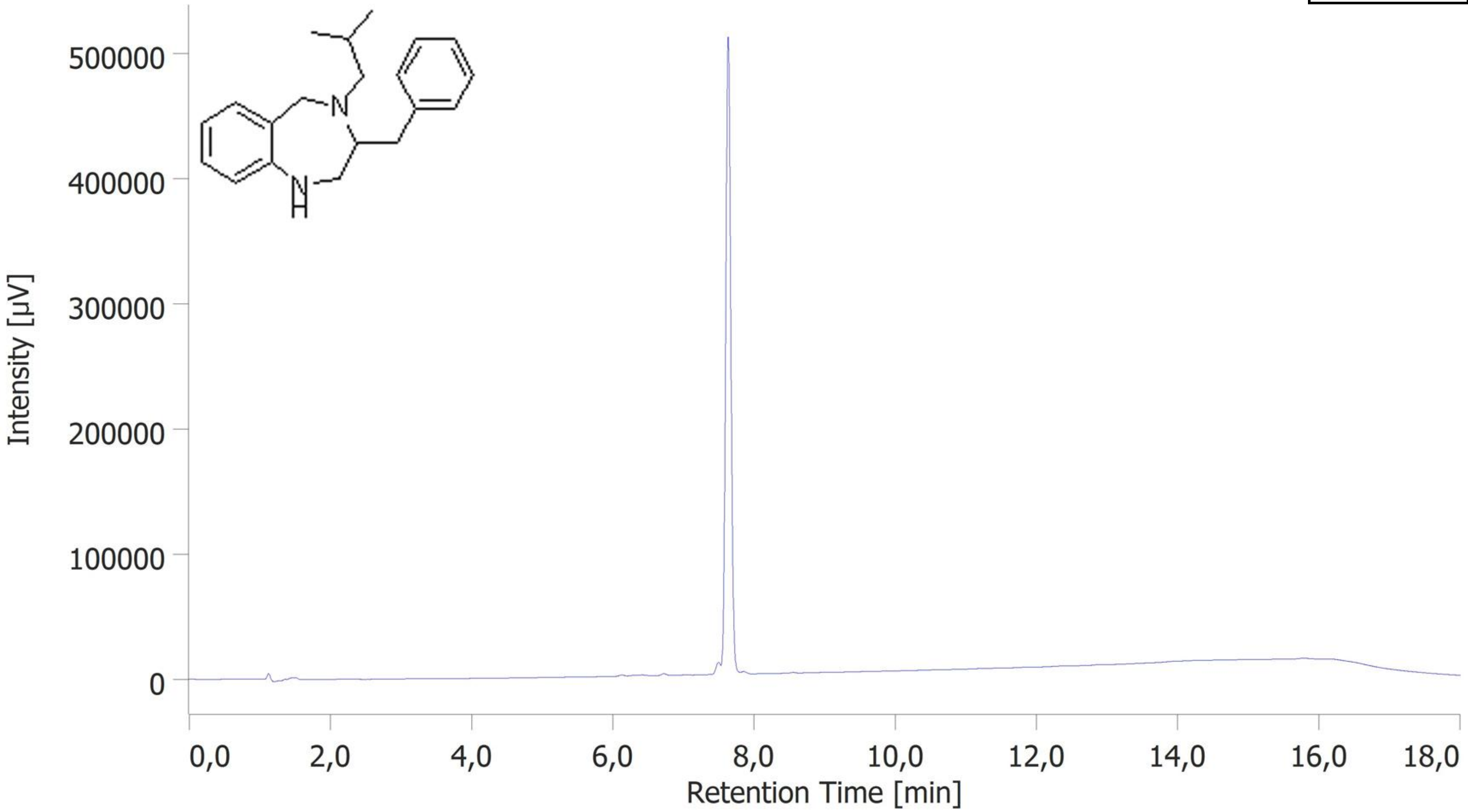

Figure S39: HPLC trace of compound **37**

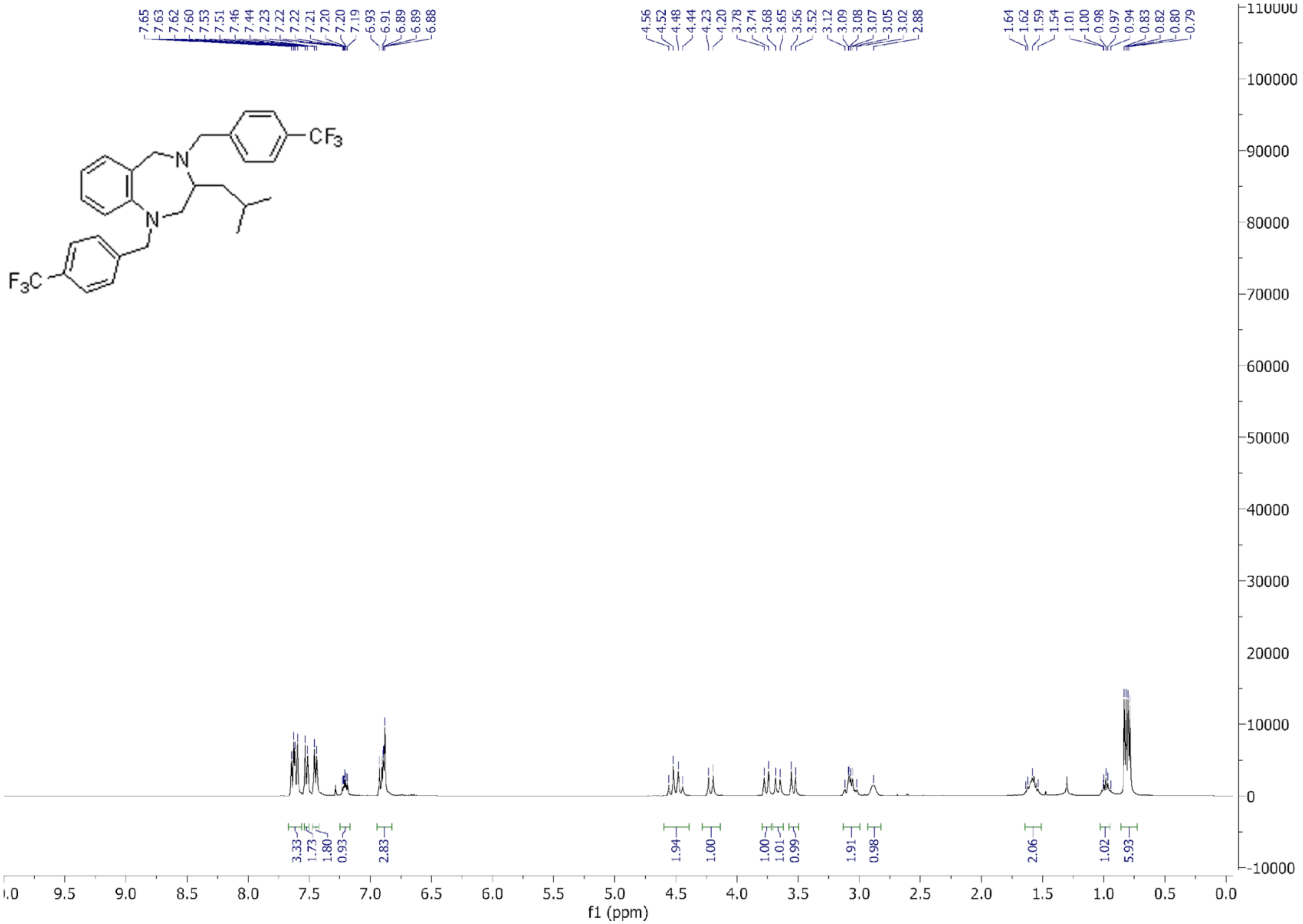

Figure S40: 1H NMR spectrum of compound **38**

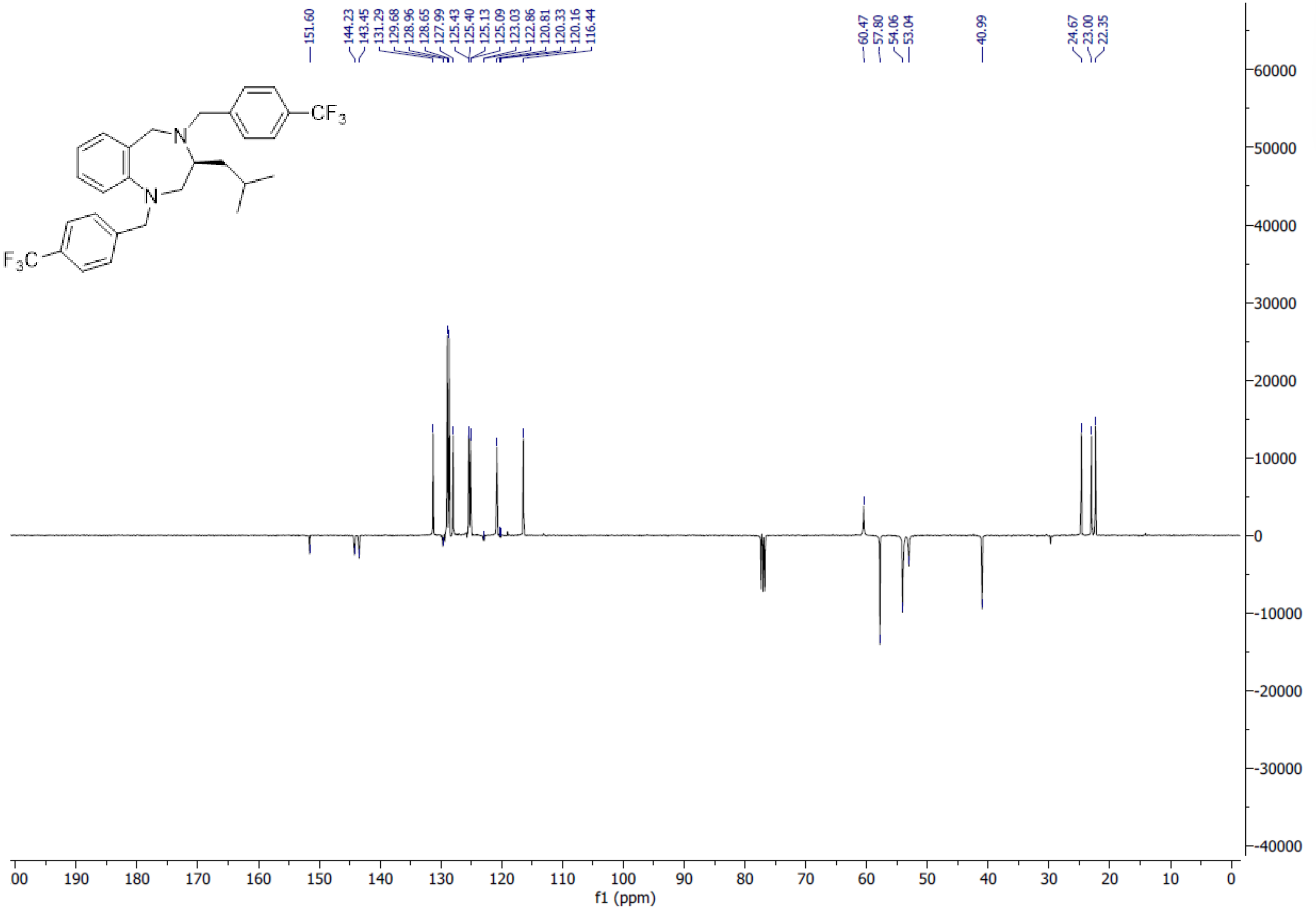

Figure S44: APT spectrum of compound **38**

RT	% AREA
6.9	99.5

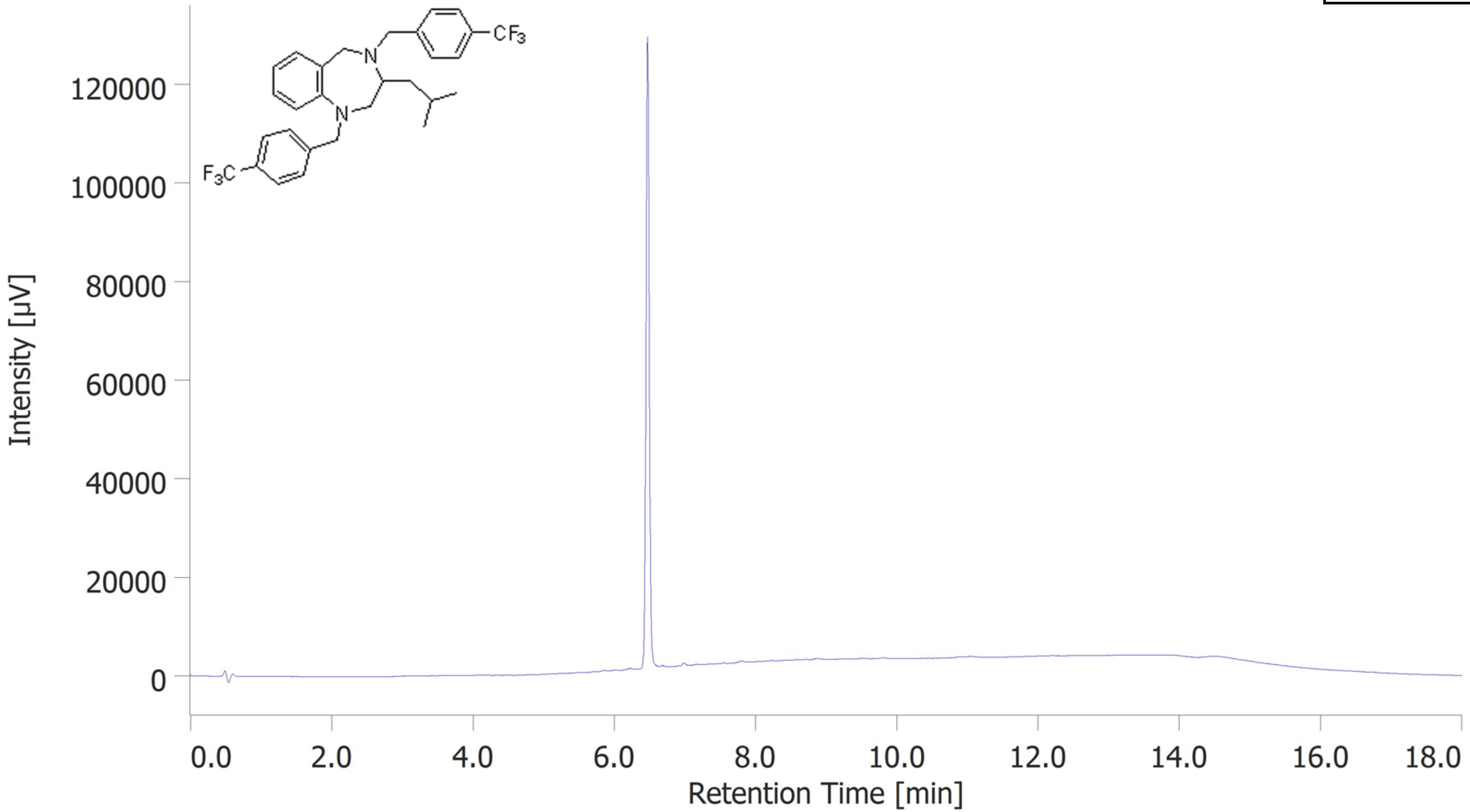

Figure S42: HPLC trace of compound **38**

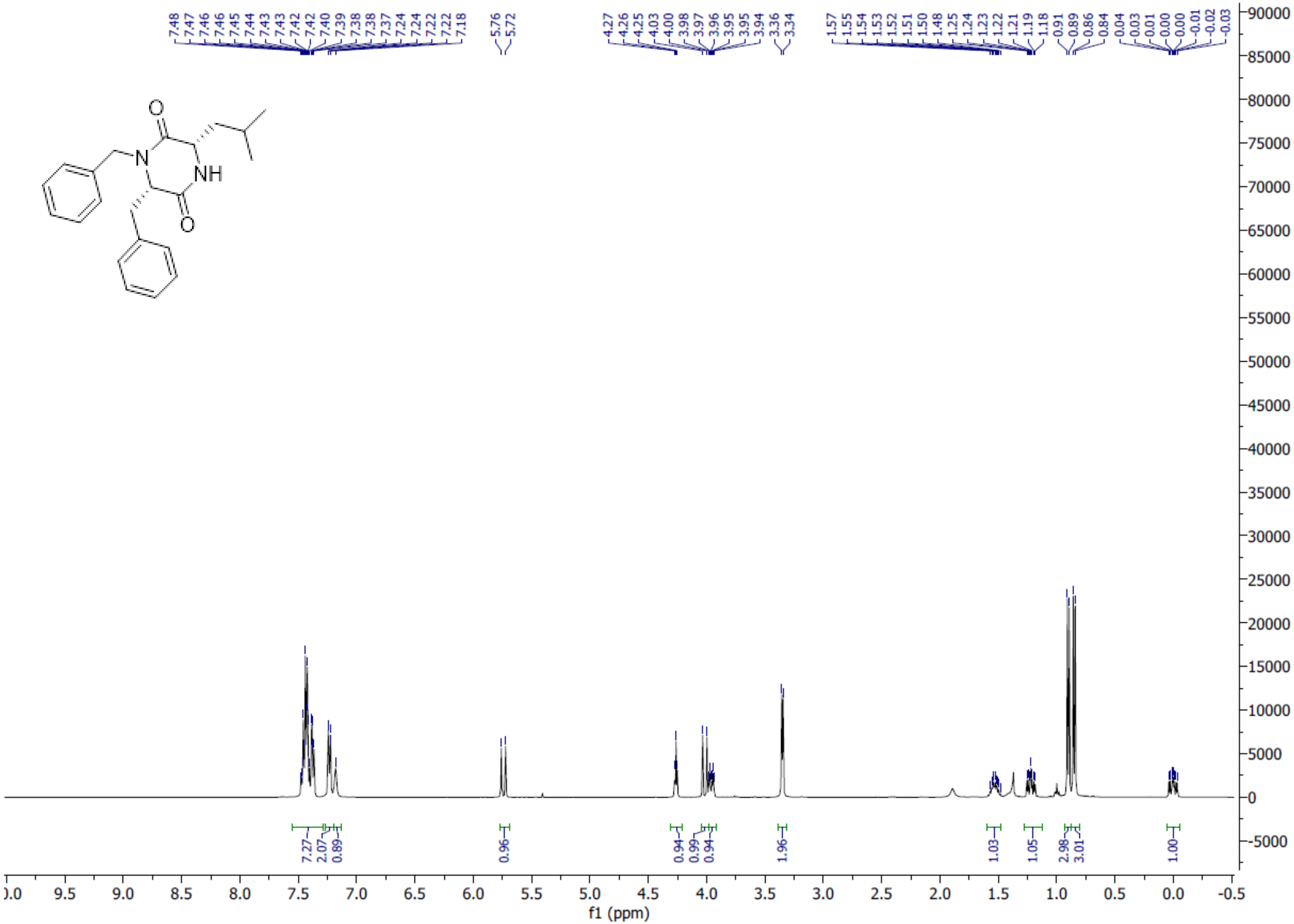

Figure S43: 1H NMR spectrum of compound **41**

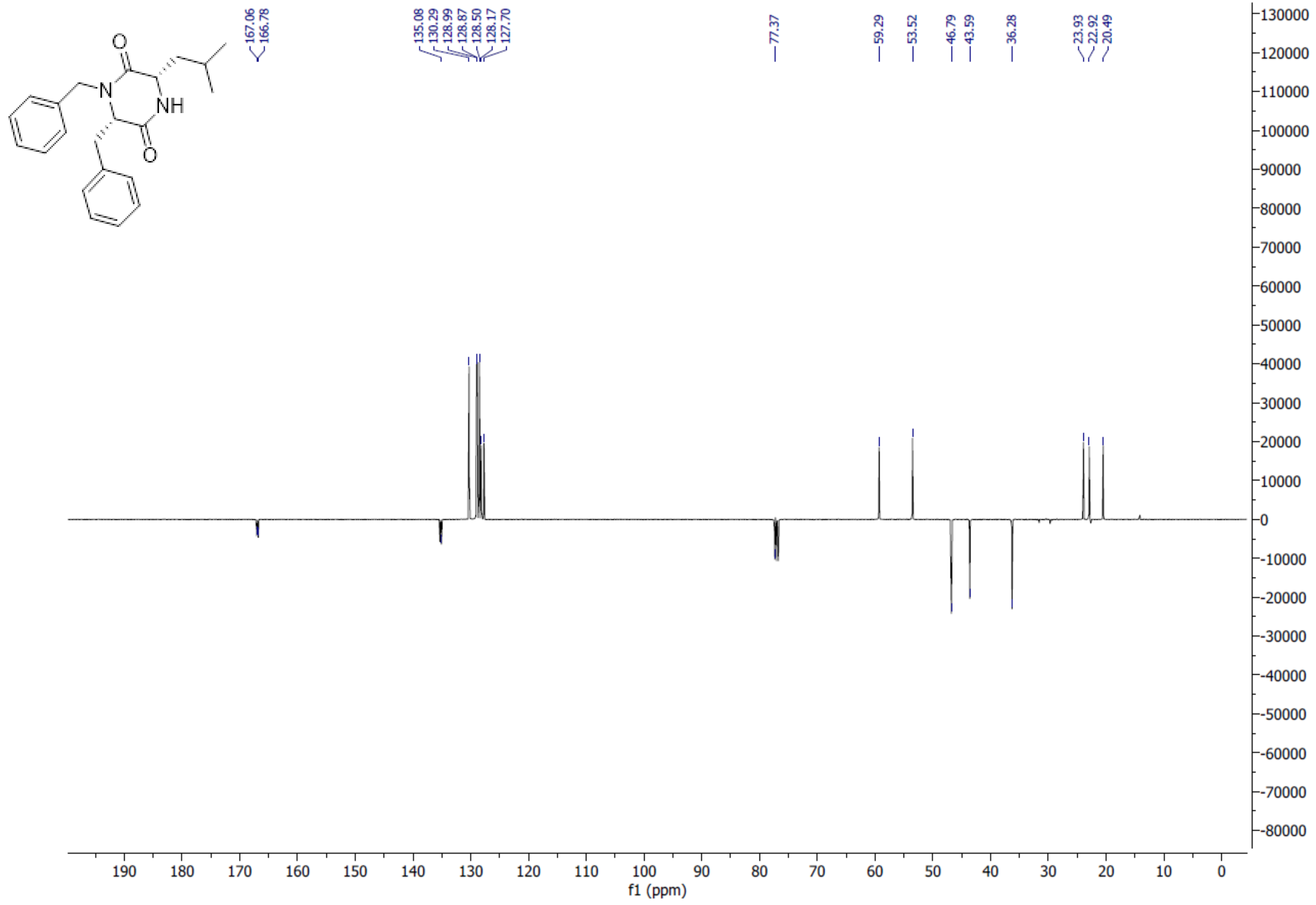

Figure S44: APT spectrum of compound **41**

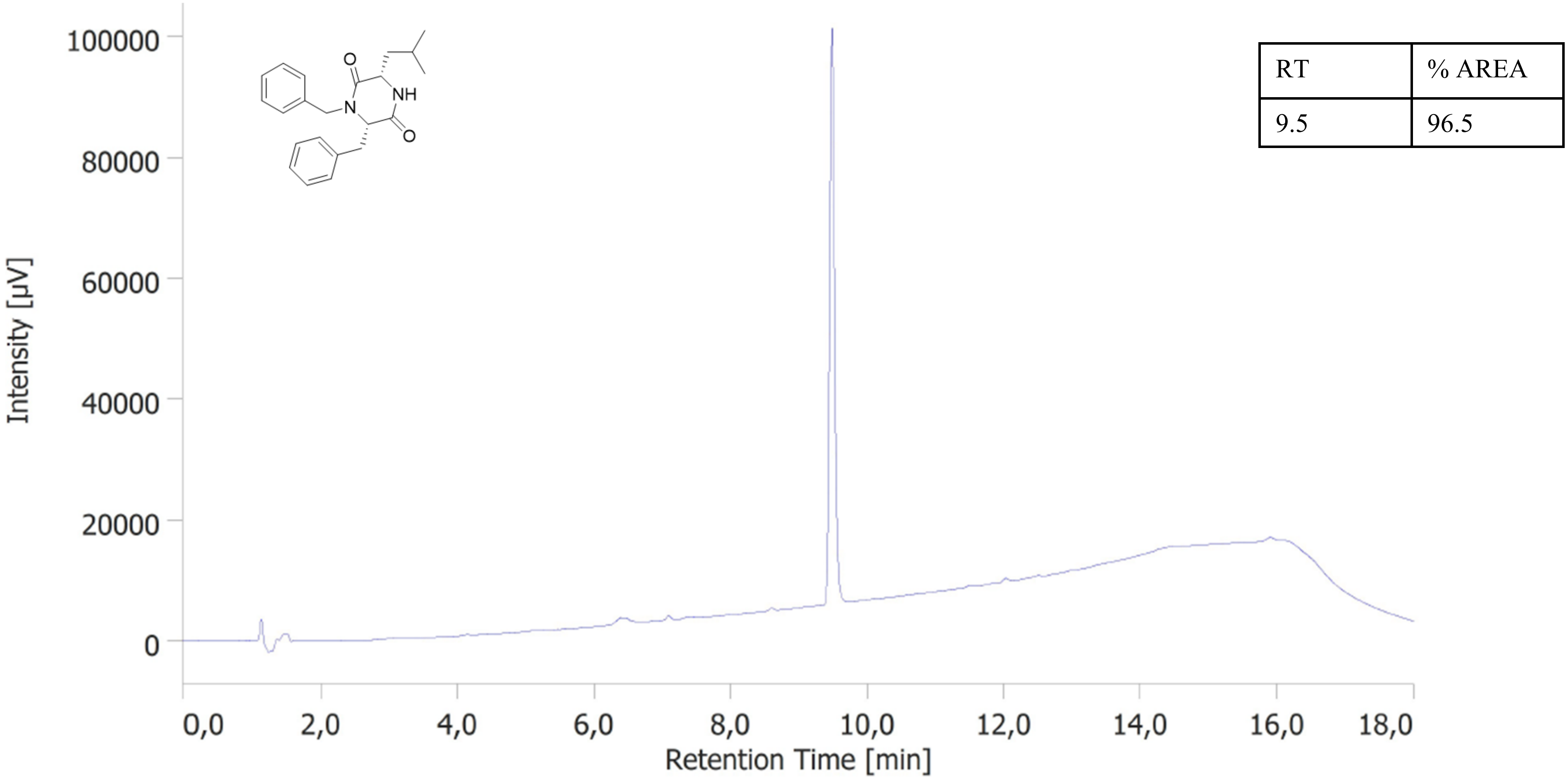

RT	% AREA
9.5	96.5

Figure S45: HPLC trace of compound **41**

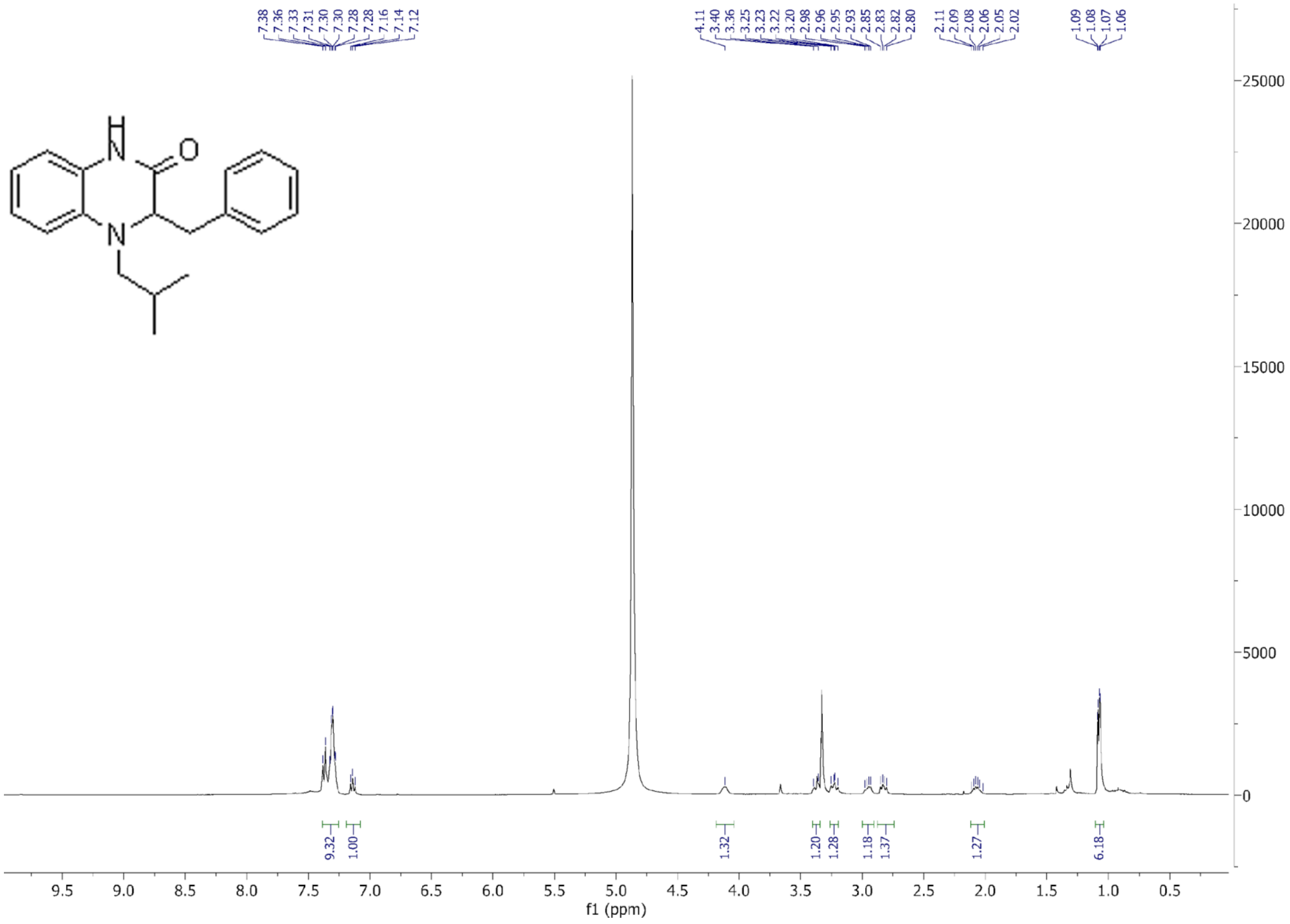

Figure S46: 1H NMR spectrum of compound **48**

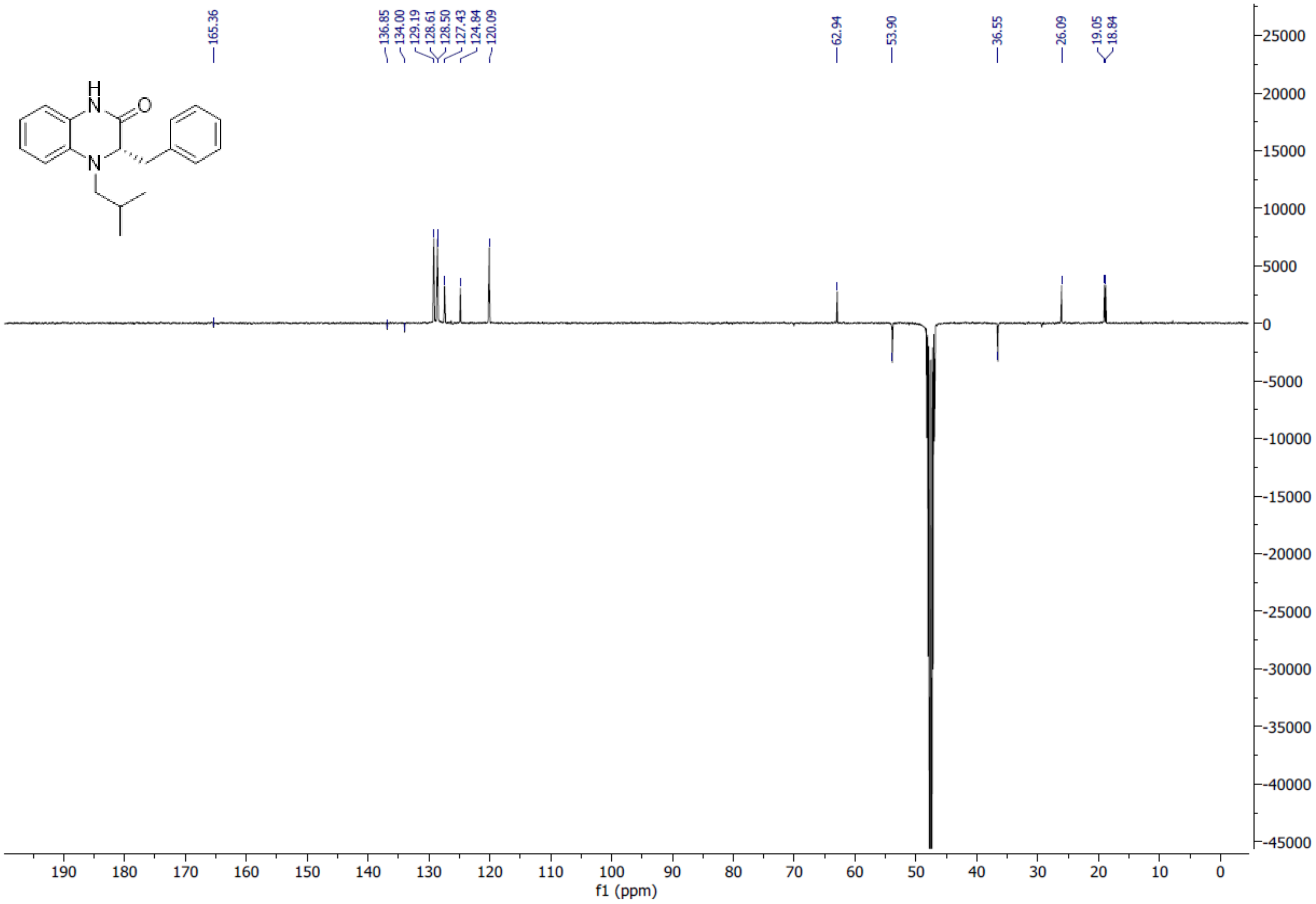

Figure S47: APT spectruma of compound **48**

RT	% AREA
11.8	99.5

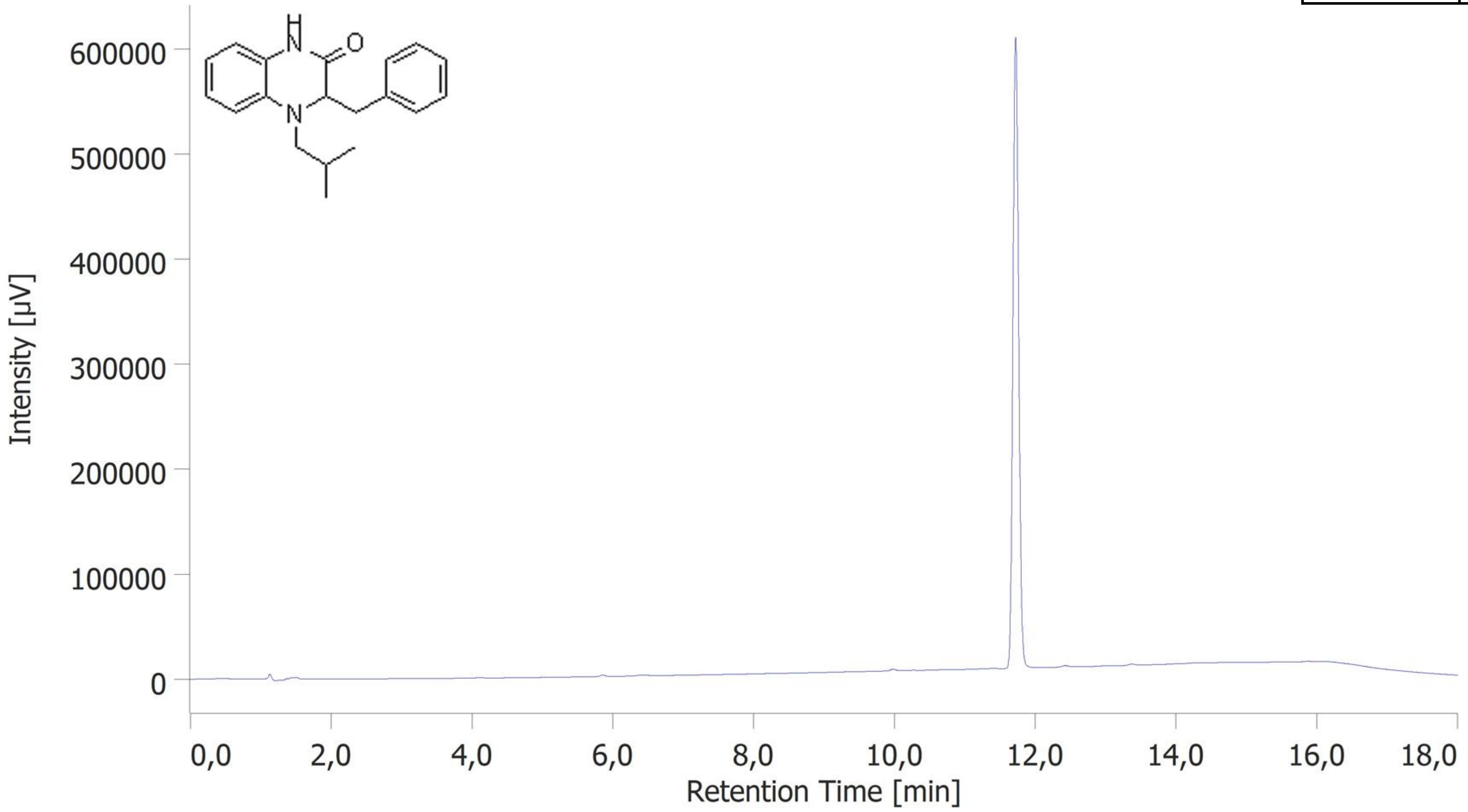

Figure S48: HPLC trace of compound **48**

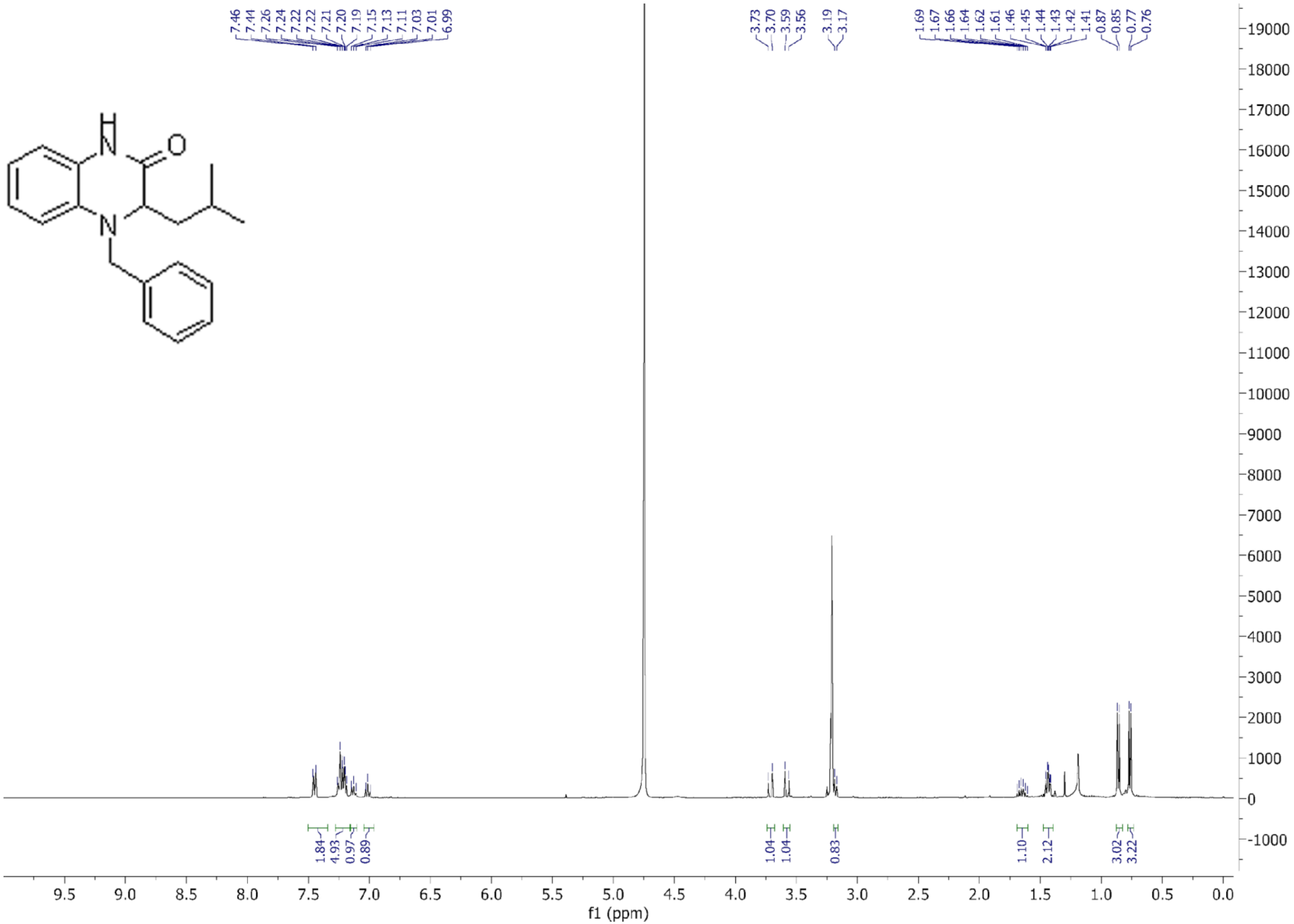

Figure S49: 1H NMR spectrum of compound **49**

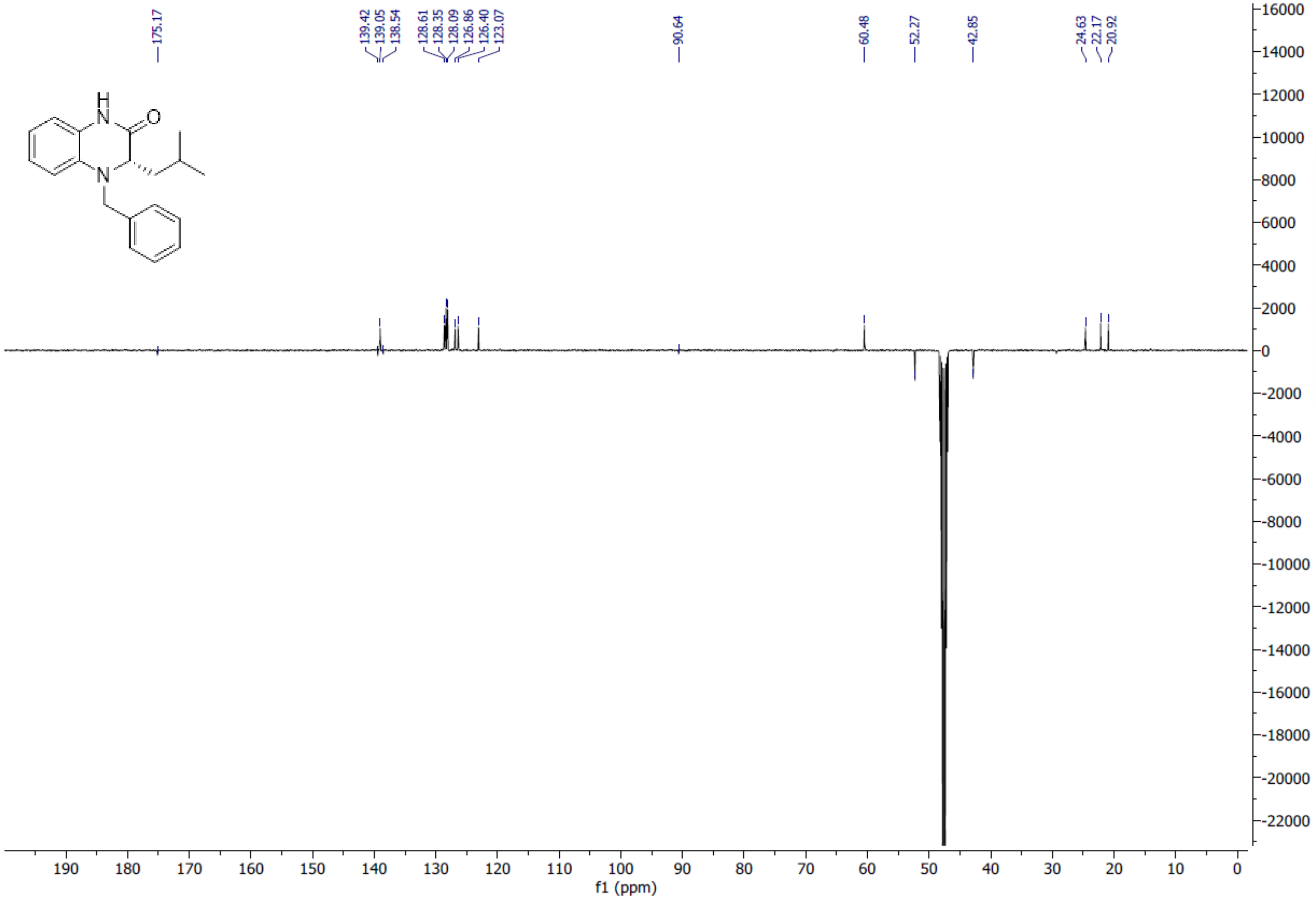

Figure S50: APT spectrum of compound **49**

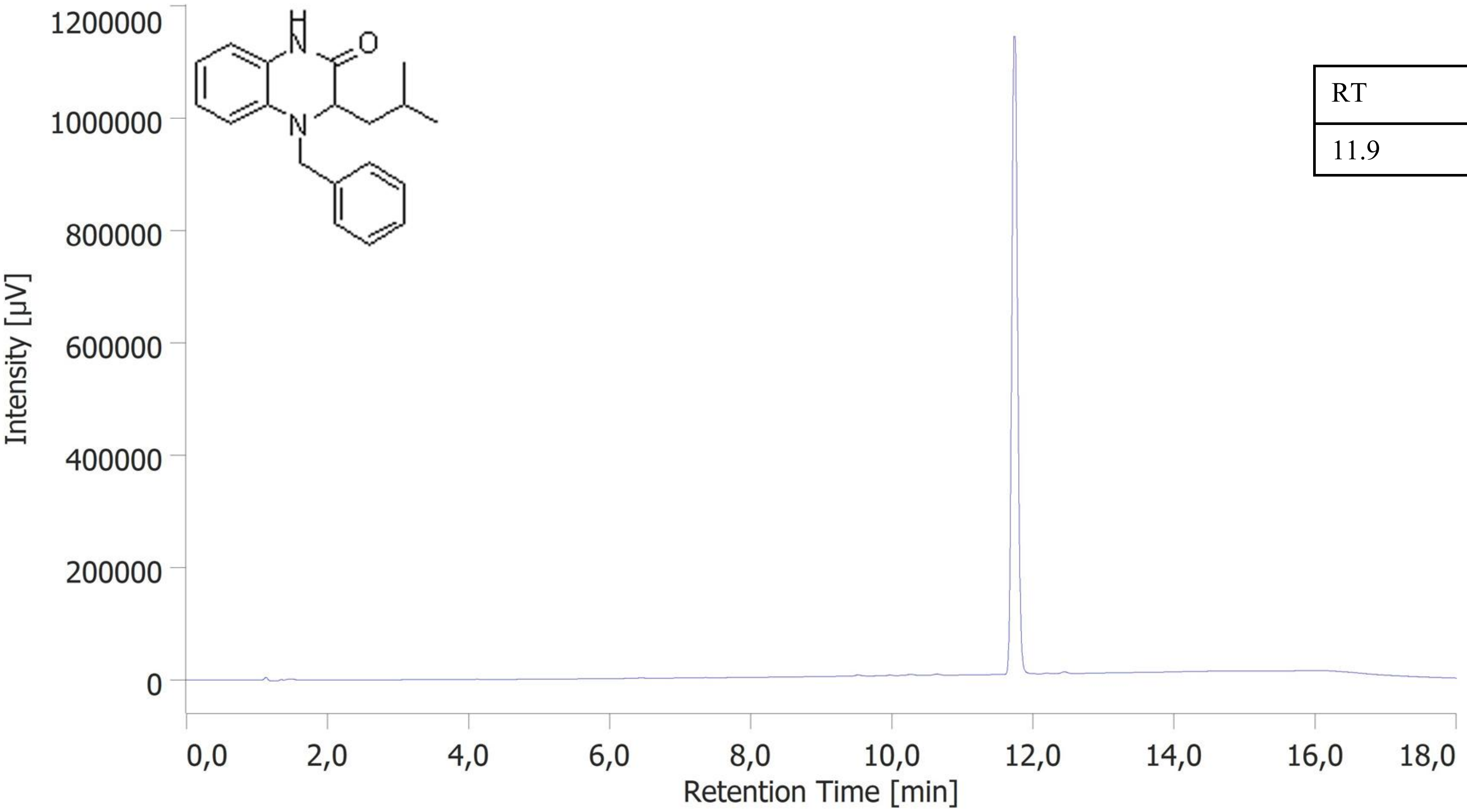

RT	% AREA
11.9	99.5

Figure S51: HPLC trace of compound **49**

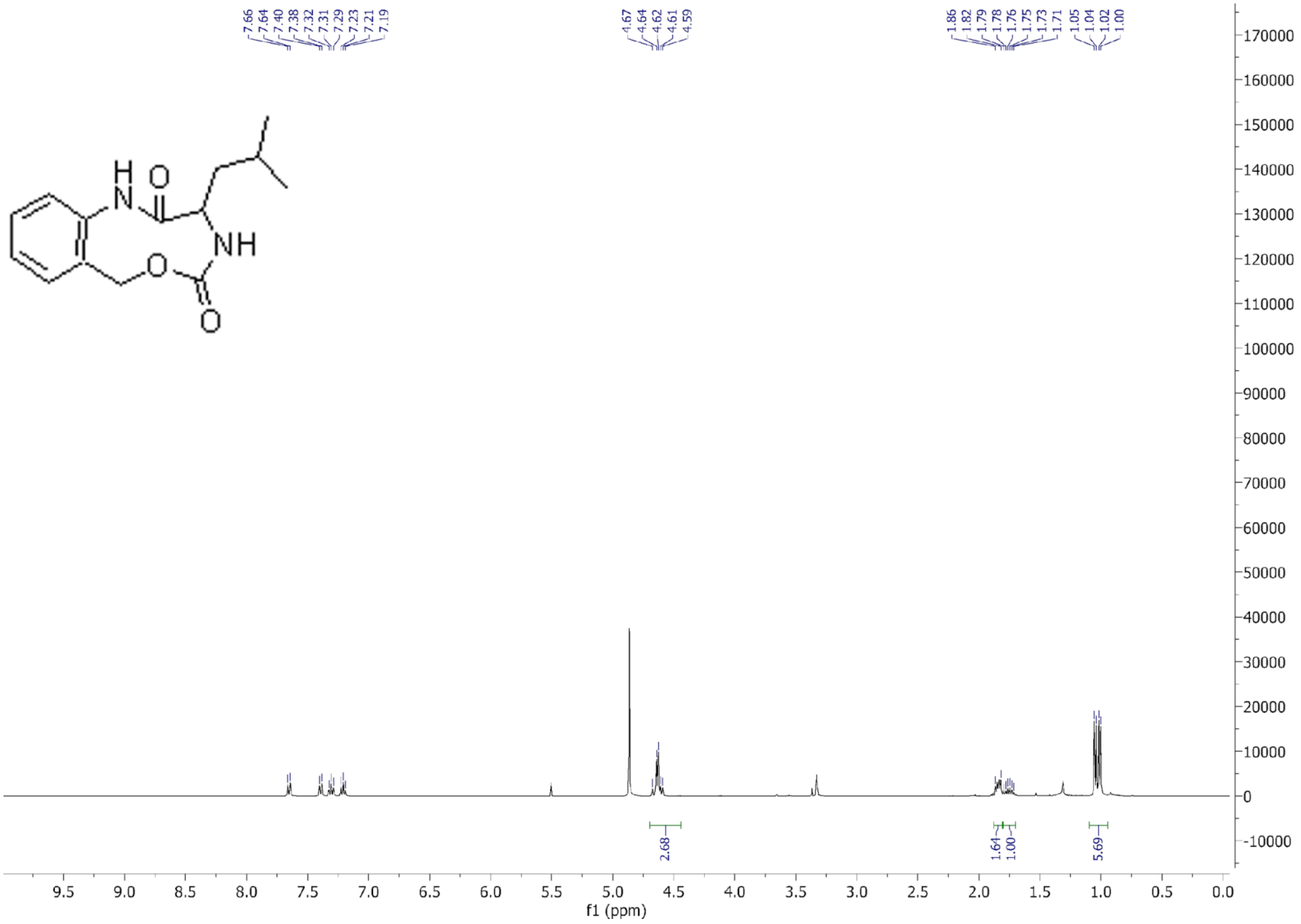

Figure S52: 1H NMR spectrum of compound **52**

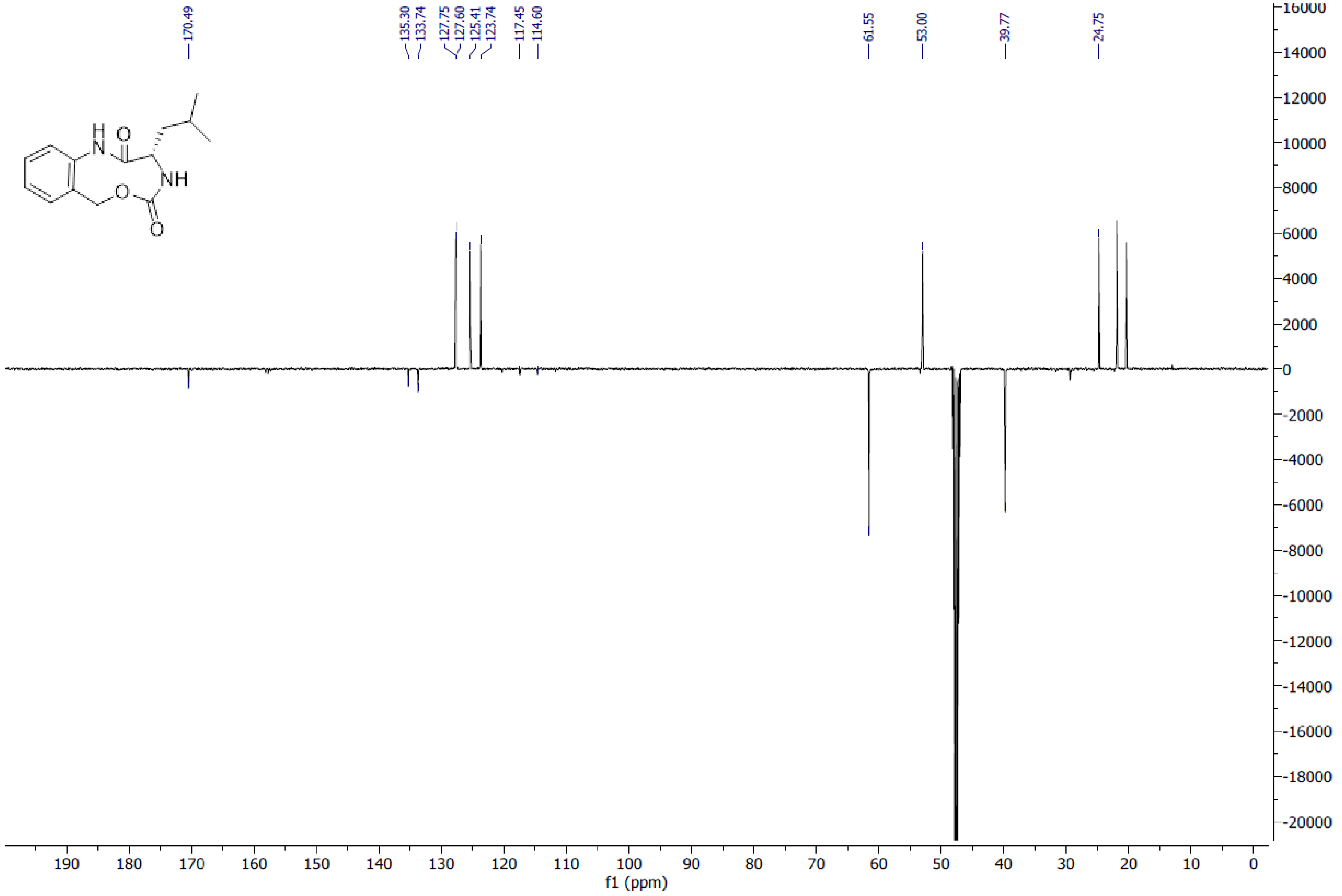

Figure S53: APT spectrum of compound **52**

RT	% AREA
8.1	99.8

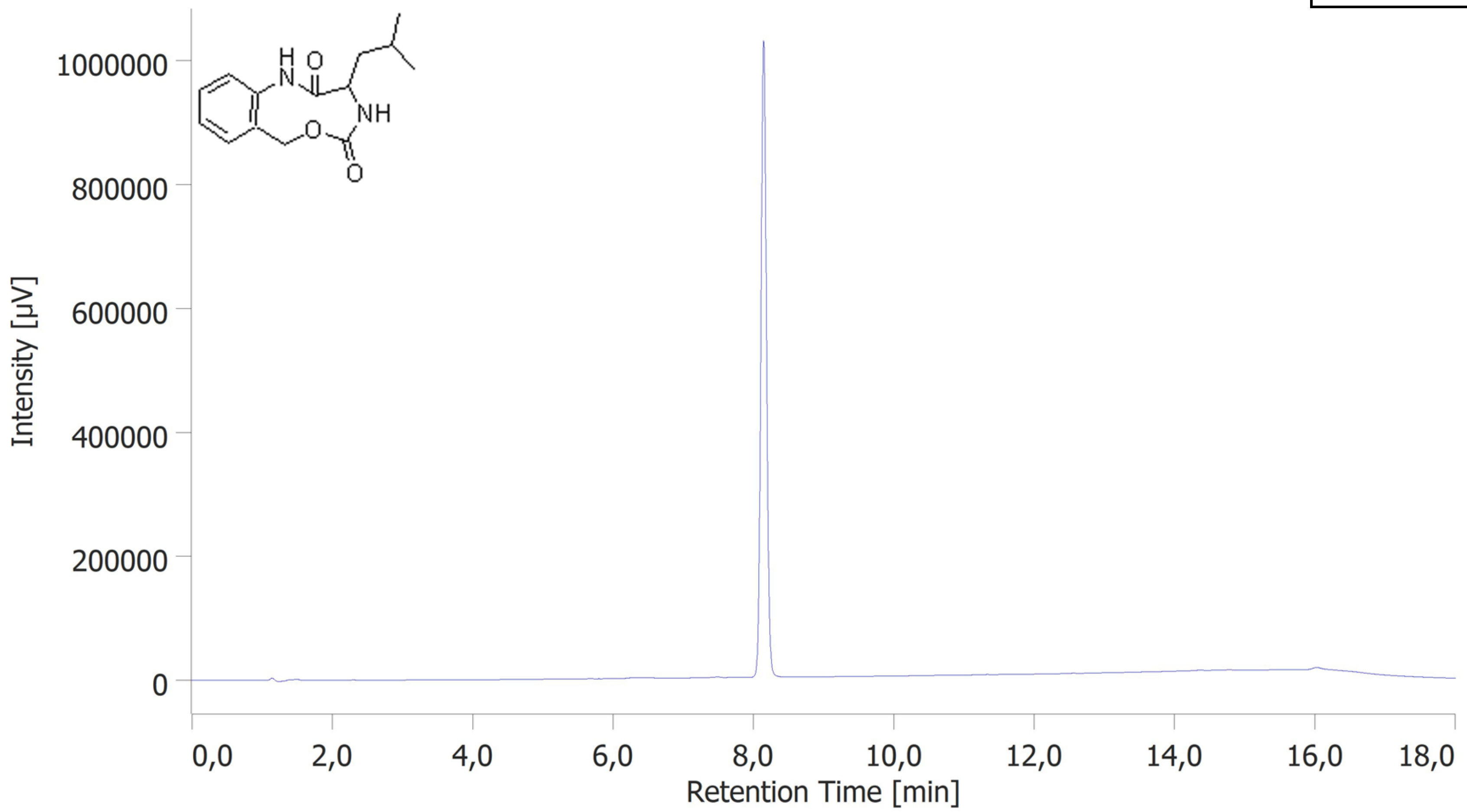

Figure S54: HPLC trace of compound **52**

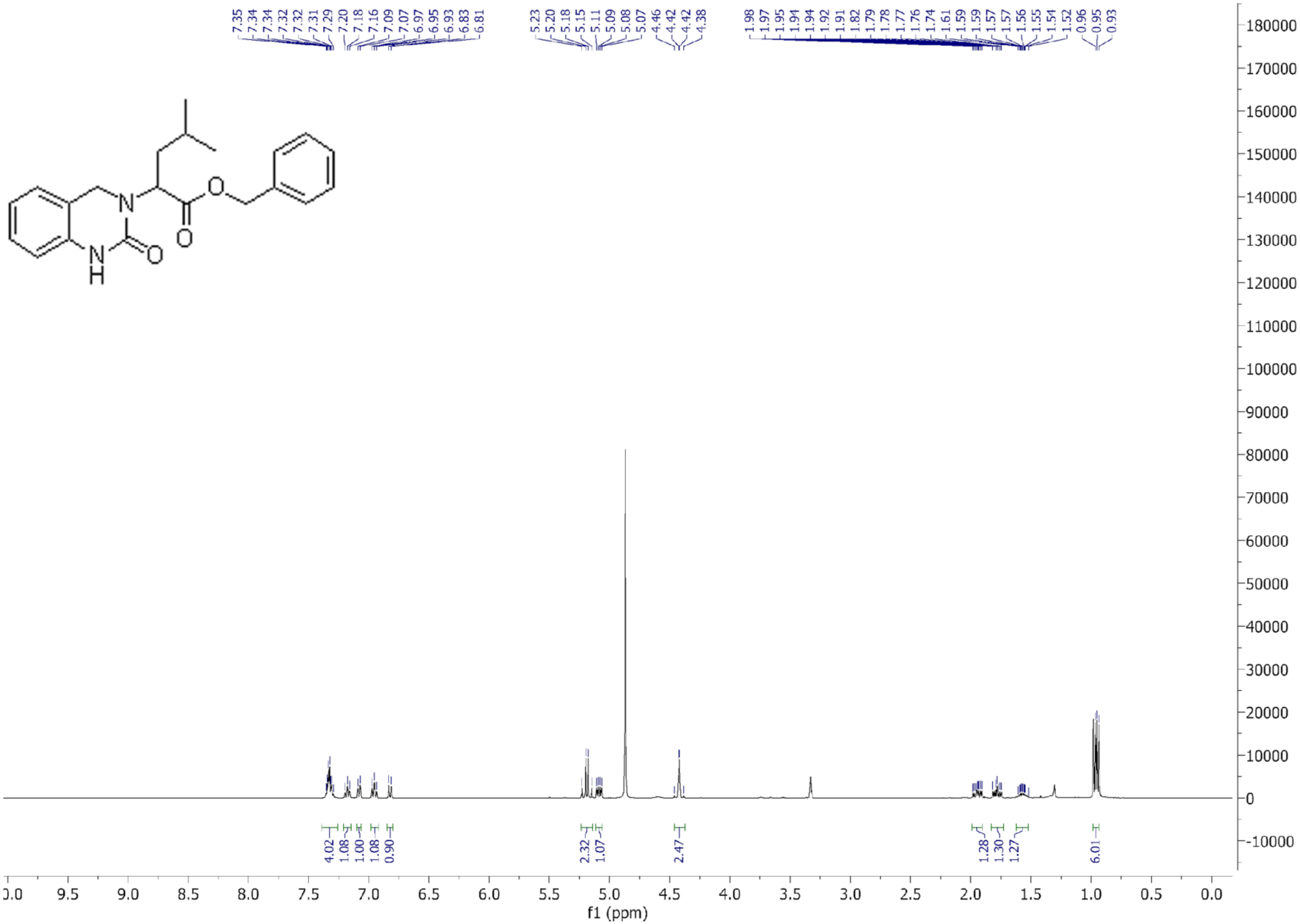

Figure S55: 1H NMR spectrum of compound **63**

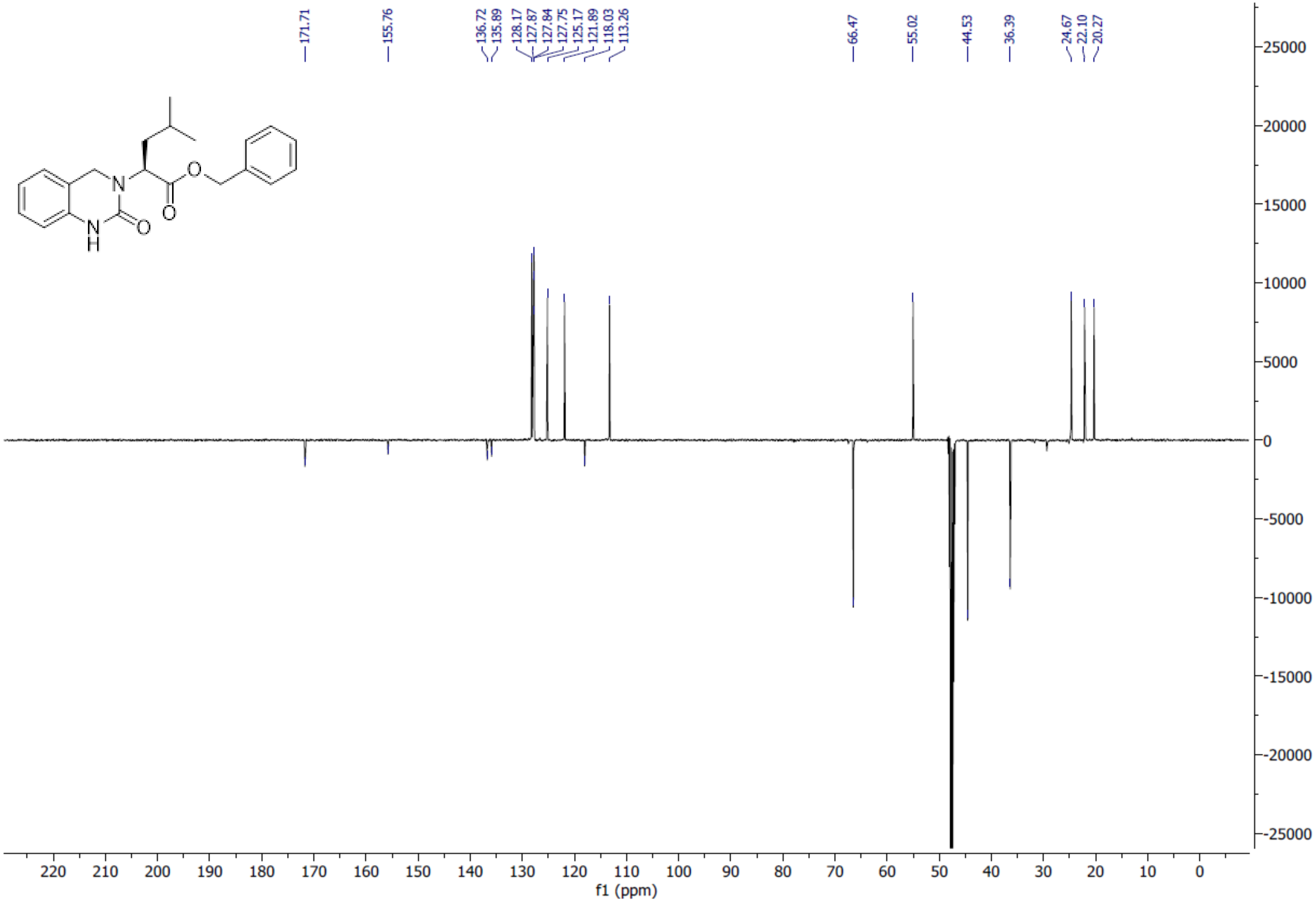

Figure S56: APT spectrum of compound **63**

RT	% AREA
10.9	99.5

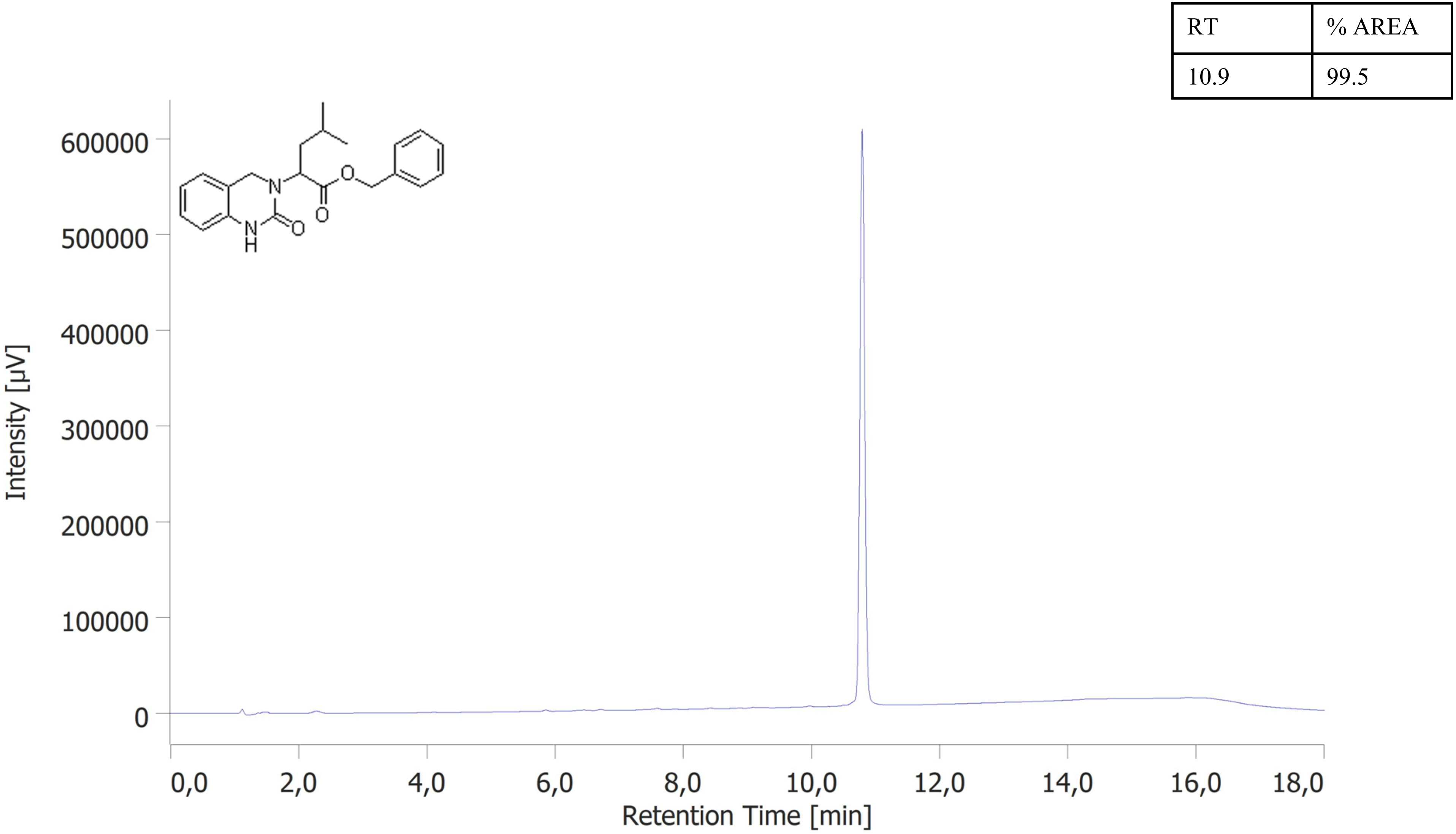

Figure S57: HPLC trace of compound **63**

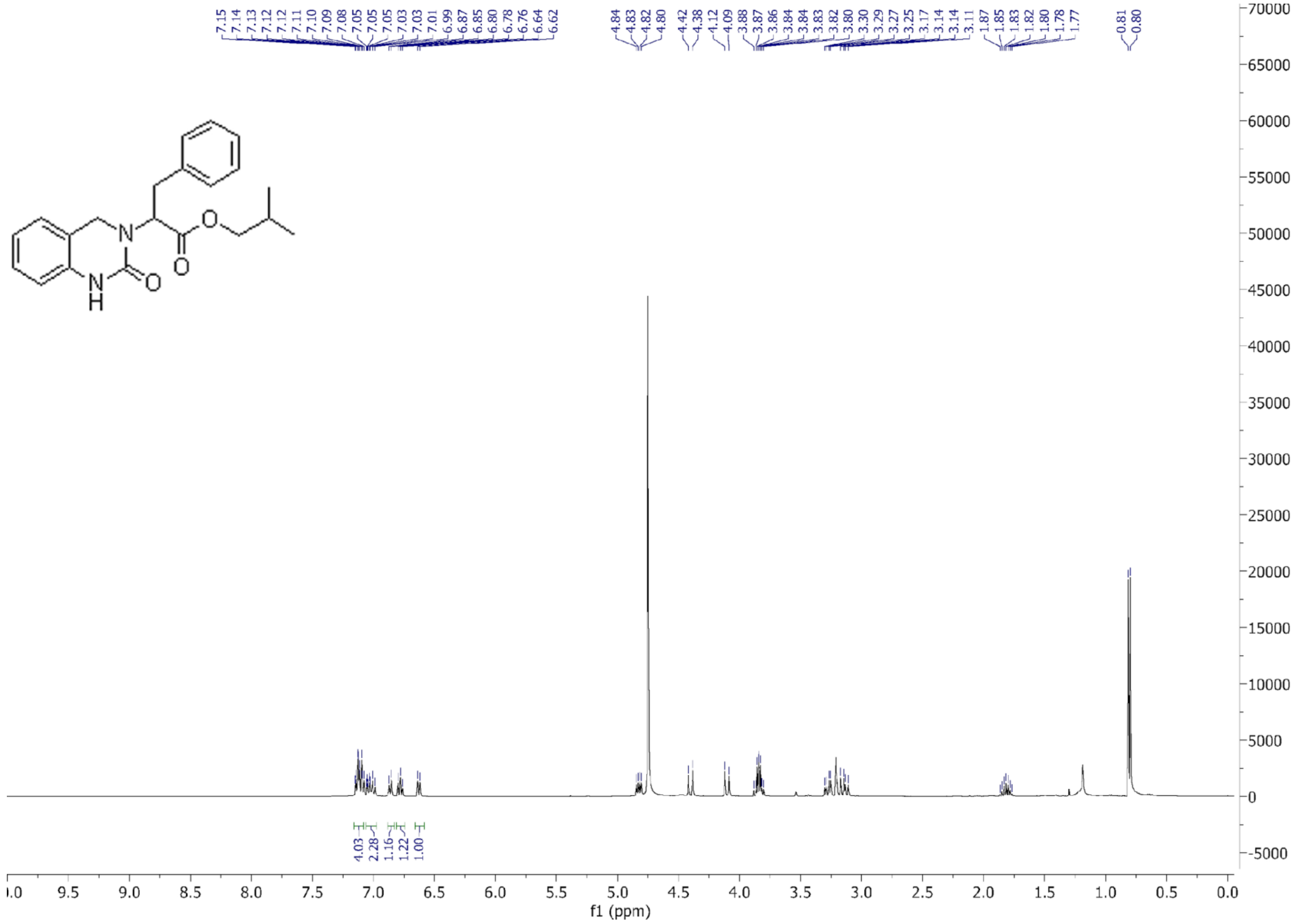

Figure S58: 1H NMR spectrum of compound **64**

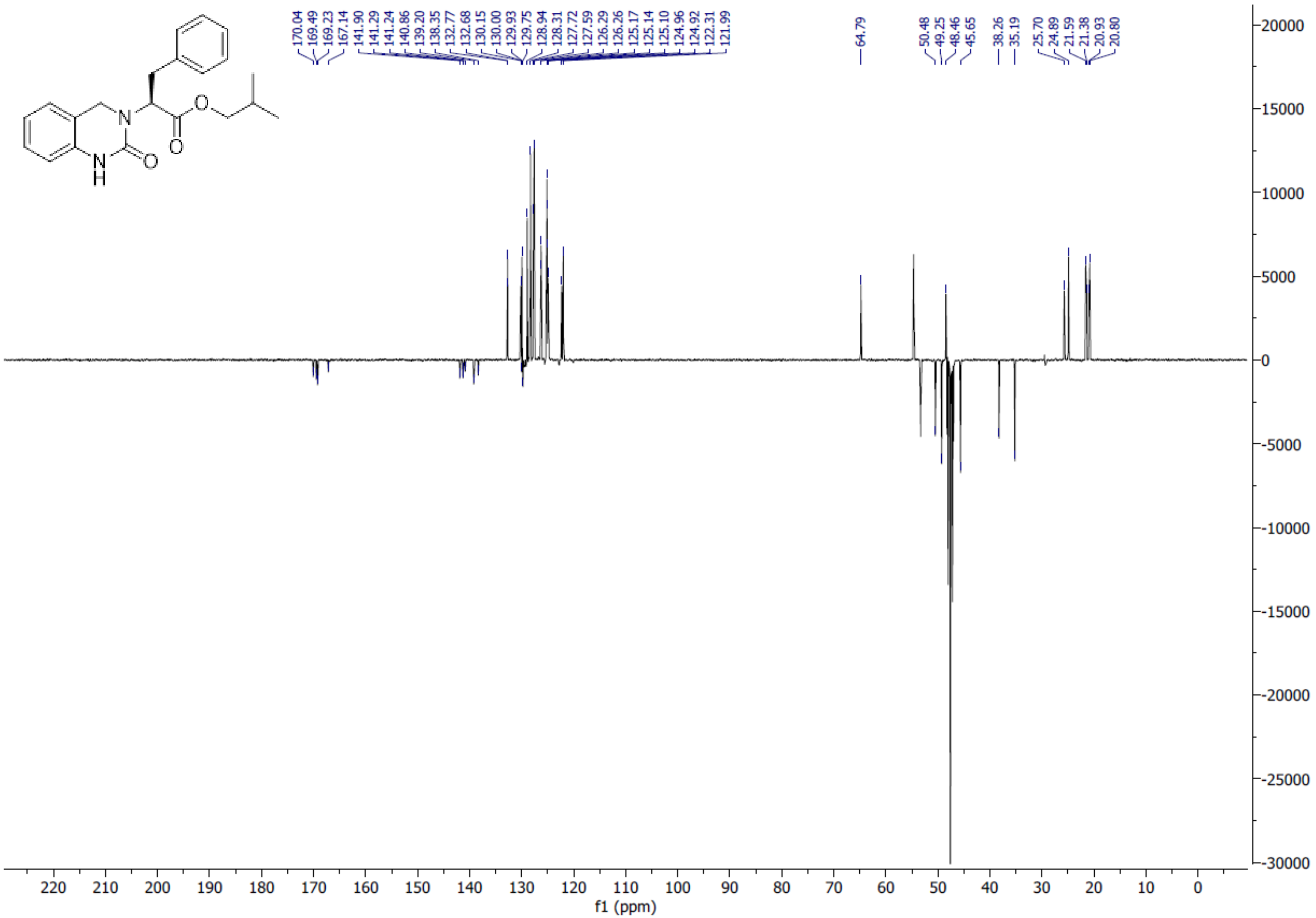

Figure S59: APT spectrum of compound **64**

RT	% AREA
10.6	99.5

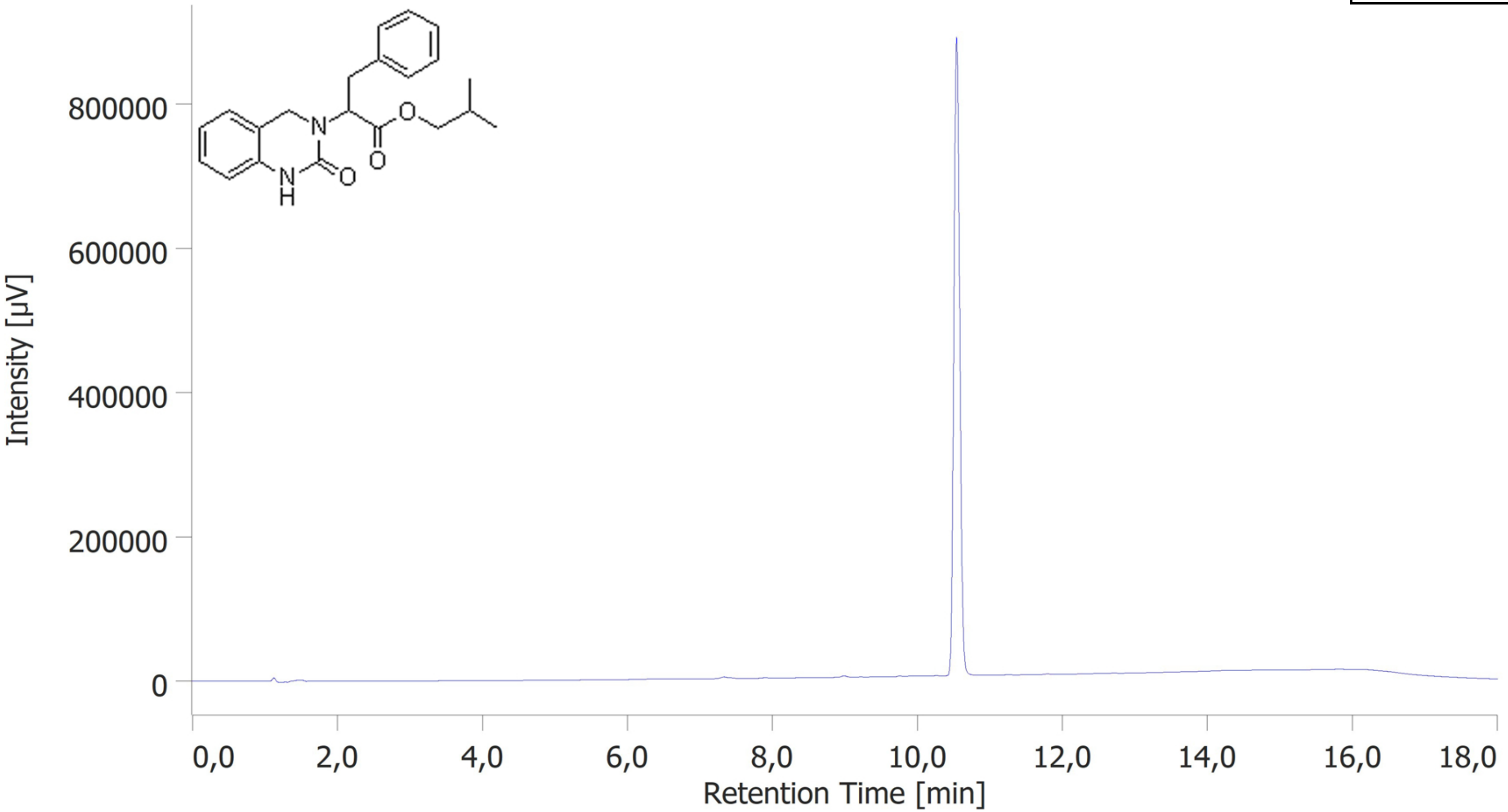

Figure S60: HPLC trace of compound **64**

Predicted binding between 31 and 63 and TRPA1

Based on the promising results obtained from the *in vitro* assays, we decided to further rationalize the activity of **31** and **63** through a molecular docking experiment involving Transient Receptor Potential Cation Channel Subfamily A Member 1 (TRPA1). TRPA1 is a cation channel expressed in sensory neurons. It is known to have a role in inflammatory diseases like arthritis, chronic obstructive pulmonary disease, chronic cough, and asthma besides pain and itch in general.

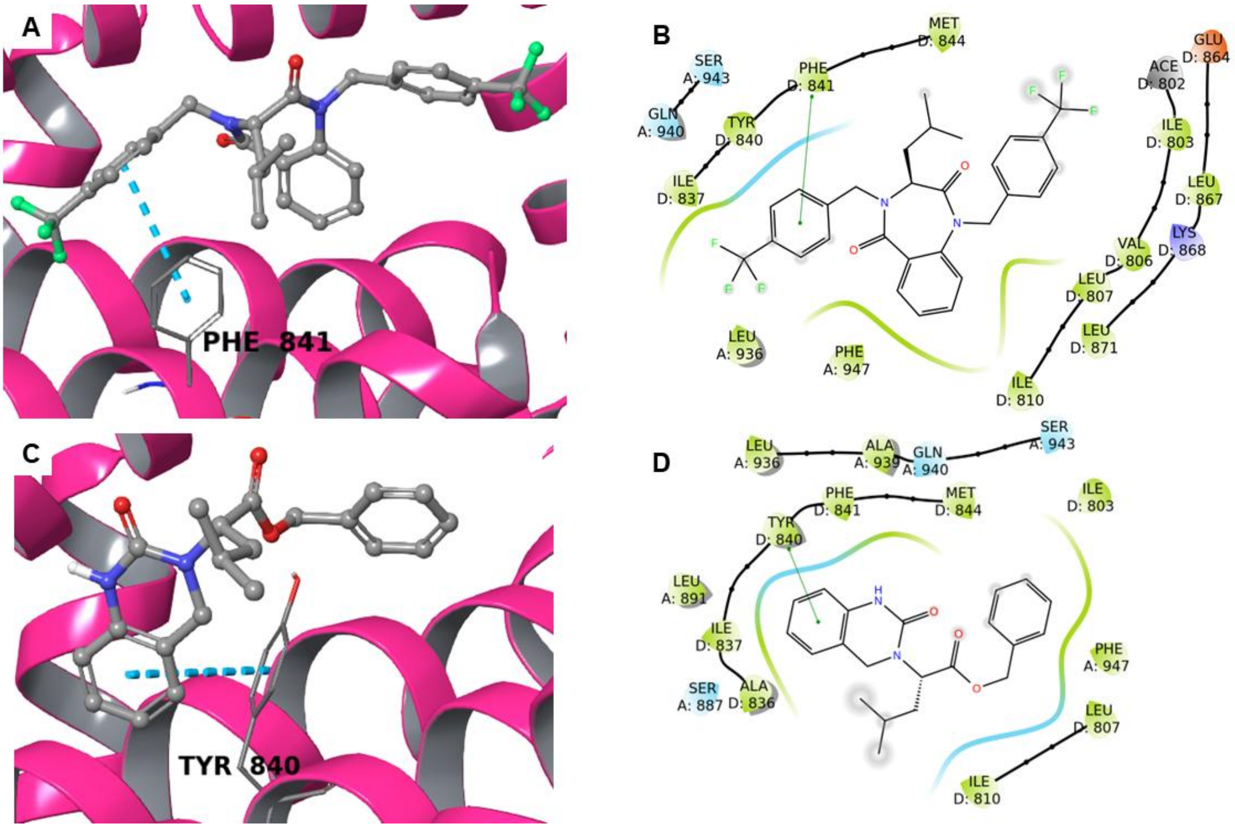

Figure S61. Predicted binding between **31** and **63** and TRPA1 (pink ribbon, PDB ID: 6X2J). **A-C**) 3D view. Cyan dotted lines indicate π-π stacking and the interacting amino acids are labelled. **B-D**) 2D view. Green lines represent π-π stacking. Polar amino acids are in cyan, hydrophobic ones are in green, and negatively charged residues are in red. The most important interactions with aromatic residues, such as Tyr840 and Phe841, which are crucial in the binding of known non-covalent inhibitors of TRPA1, are highlighted.

Interacting targets of compounds 31 and 63 predicted by IVS

In addition to the protein targets in common for compounds **31** and **63** (see main text) identified by IVS, further not shared proteins were selected and are analyzed and discussed below.

In detail, for compound **31**, the IVS experiments highlighted the Receptor tyrosine-protein kinase erbB-2 (ERBB2), the 17-beta-hydroxysteroid dehydrogenase 13 (DHB13), the E3 ubiquitin-protein ligase Mdm2 (MDM2), the Retinoic acid receptor RXR-alpha (RXRA), the Poly [ADP-ribose] polymerase 1 (PARP1, and the Serine/threonine-protein kinase B-raf (B-raf) as probable interacting targets (Figure S62).

ERBB2 (also known as HER2) is a receptor tyrosine kinase belonging to the EGFR family that is involved in cell growth and differentiation. Some aggressive cancer types, like breast and gastric cancer, are often associated with an overexpression of ERBB2. Compound **31** establishes a hydrogen bond with Asp863 (Asp165 in the PDB 7JXH),[1] like the known HER2 inhibitor Lapatinib.[2] DHB13, an enzyme mainly expressed in the liver, is associated with non-alcoholic steatohepatitis (NASH), fibrosis, and neoplasms like breast, prostate, or polycystic kidney cancer.[3] The Phe220, involved in a hydrogen bond with the carbonyl moiety of **31**, is reported to be an important residue in the binding cavity of this enzyme.[3]

MDM2 is a negative regulator of the tumor suppressor protein p53, controlling its degradation through the ubiquitin-proteasome pathway. Overexpression of MDM2 can reduce p53 activity, promoting tumor progression and making it a potential target for cancer therapies aimed at restoring p53 function. The binding site is found at the interface between two monomers with the trifluoromethyl moiety well inserted in the hydrophobic area of the binding pocket and one of the carbonyls of **31** engaging a hydrogen bond with His96 (referred to PDB ID: 1RV1). [4, 5]

RXRA is a nuclear receptor that regulates gene expression by forming heterodimers with other nuclear receptors, such as RARs, PPARs, FXRs, LXRs, and VDR. It plays a crucial role in cellular differentiation, metabolism, and immune response.[6] The reference PDB for this protein is 4N8R, [7] which highlighted a novel binding site located at the interface of the tetramer composed by the RXRA ligand binding domain composed of several hydrophobic residues like Tyr305 and Phe439 with which **31** interacts through π-π stacking.

PARP1 is an enzyme that detects damaged DNA and starts DNA repair mechanisms. Inhibitors of PARP1 have been developed as targeted cancer therapies, especially for tumors with BRCA1/2 mutations, as it has been demonstrated that inactivation of both PARP1 and BRCA1/2 induces cell death.[8] Here, **31** interacts with Tyr907 (Tyr166 in the reference PDB 6NTU), [9] a key interactor in the ligand binding cavity.[8] Eventually, B-Raf is a kinase that is part of the MAPK/ERK signaling pathway, regulating cell growth, differentiation, and survival. Compound **31** makes proficient contacts with Ser536.

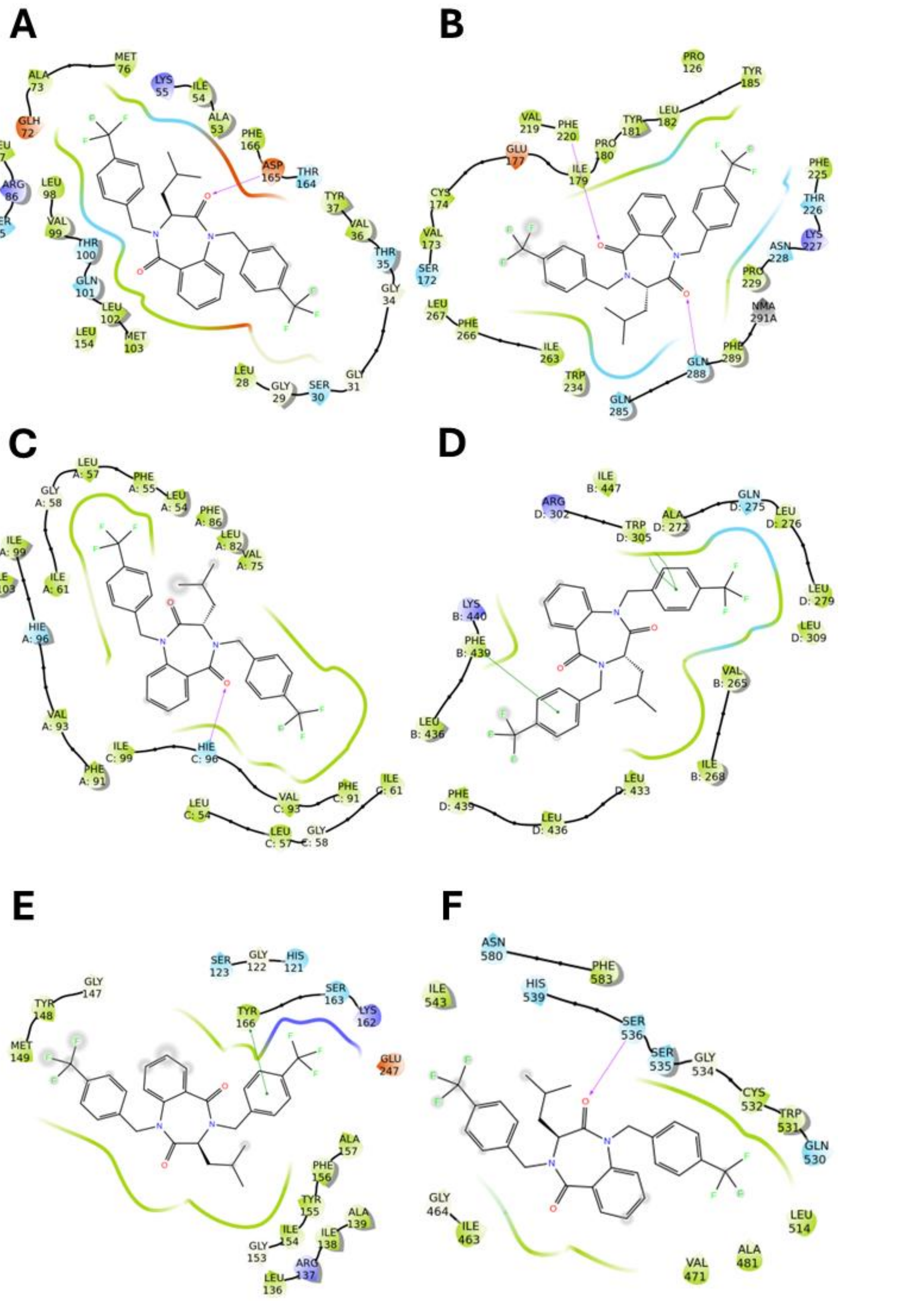

Figure S62. 2D view of the interactions between **31** and **A)** Receptor tyrosine-protein kinase erbB-2 (ERBB2, PDB ID: 7JXH), **B)** 17-beta-hydroxysteroid dehydrogenase 13 (DHB13, PDB ID: 8G9V), **C)** E3 ubiquitin-protein ligase Mdm2 (MDM2, PDB ID: 1RV1), **D)** Retinoic acid receptor RXR-alpha (RXRA, PDB ID: 4N8R), **E)** Poly [ADP-ribose] polymerase 1 (PARP1, PDB ID: 6NTU), and **F)** Serine/threonine-protein kinase B-raf (B-Raf, PDB ID: 5HIE). Green lines represent π-π stacking,

and pink lines are hydrogen bonds. Polar amino acids are in cyan, hydrophobic ones are in green, positively charged residues are in blue, and glycine residues are in white.

Concerning **63** (Figure S63), IVS highlighted the Aurora kinase A (AURKA), the Protein-tyrosine kinase 6 (BRK), and the GTPase Kras (RASK) as putative targets.
AURKA, BRK), and RASK are key regulators of intracellular signaling and cell cycle progression, with distinct but interconnected roles in oncogenesis.
AURKA is a serine/threonine kinase that orchestrates mitotic progression, and its overexpression has been connected to tumorigenesis.[10] Molecular docking calculations highlighted the ability of compound **63** in making hydrogen bonds with Ala213, a key residue of AURKA.
BRK, a non-receptor tyrosine kinase, plays a dual role in cell signaling, promoting proliferation and survival in epithelial cancers while exhibiting tumor-suppressive functions in normal tissues. Its catalytic triad is composed of Lys219, Asp330, and Glu235.[11] Compound **63** makes hydrogen bonds with Asp330, a key residue of the BRK binding site.
In contrast, KRAS is a small GTPase that controls downstream signaling pathways such as MAPK/ERK and PI3K/AKT, converting GTP into GDP.[12] While AURKA and BRK function as kinases that directly phosphorylate substrates, KRAS exerts its effects by activating downstream kinases. In this case, **63** interacts with Glu63 and Arg68 through three hydrogen bonds.

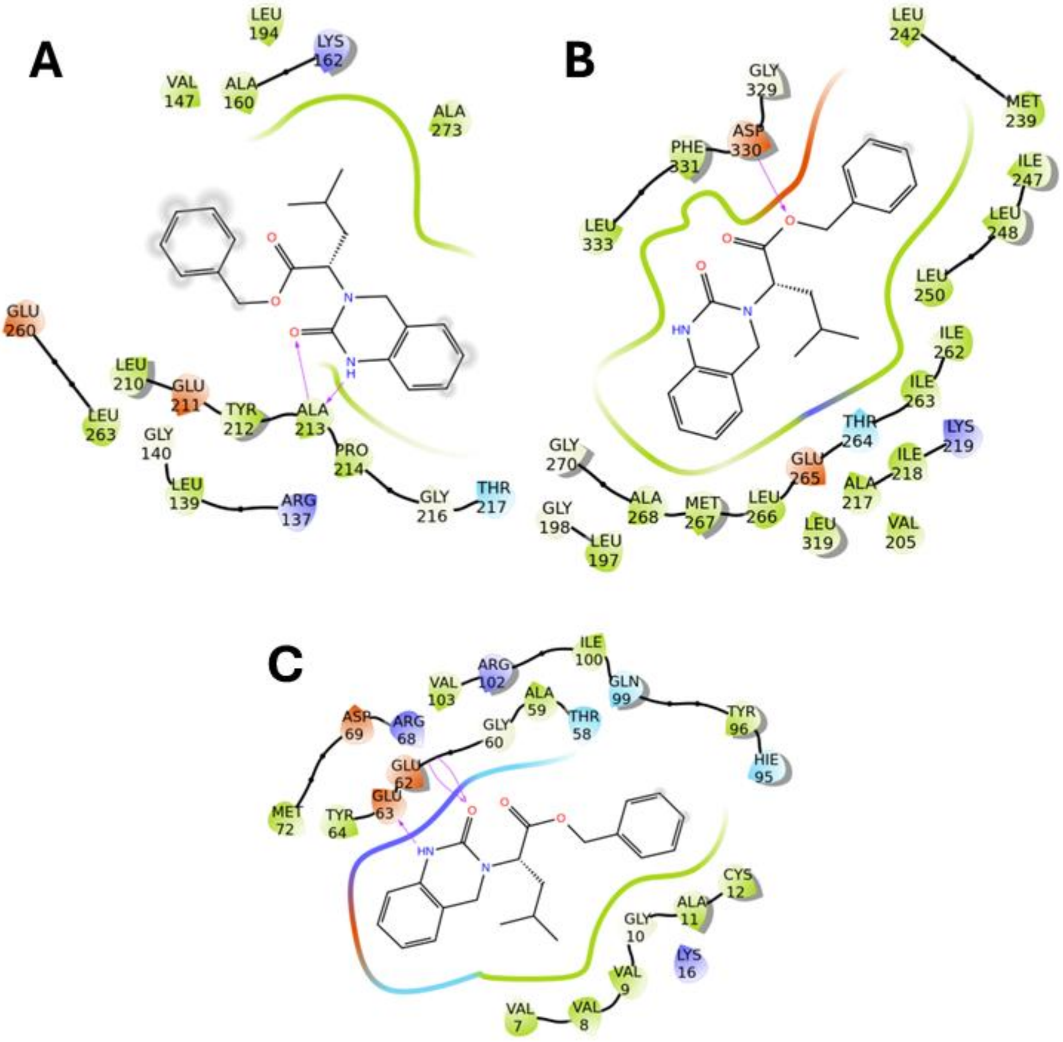

Figure S63. 2D view of the interactions between **63** and **A)** Aurora kinase A (AURKA, PDB ID: 4UYN), **B)** Protein-tyrosine kinase 6 (BRK, PDB ID: 6CZ4), and **C)** GTPase Kras (RASK, PDB ID: 8AFC). Pink lines are hydrogen bonds. Polar amino acids are in cyan, hydrophobic ones are in green, positively charged residues are in blue, and glycine residues are in white.

Bioinformatic studies for targets identification

Table S1. Identified targets for **31** cross the bioinformatics tool Super Pred (SP) and transcriptomics data (Protein Atlas). nTPM, normalized Transcripts Per Million.

**31**			
**Target Name (SP)**	**Probability (SP)**	**nTPMA375 (Protein Atlas)**	**nTPM HaCaT (Protein Atlas)**
Cathepsin D	99%	141.7	1014.8
Cannabinoid CB2 receptor	99%	0	0
Nuclear factor NF-kappa-B p105 subunit	97%	23.8	24.5
Cannabinoid CB1 receptor	95%	3.4	0
Signal transducer and activator of transcription 3	94%	35.1	84.4
Transcription intermediary factor 1-alpha	93%	11.9	7.5
Voltage-gated N-type calcium channel alpha-1B subunit	91%	0	0
G-protein coupled bile acid receptor 1	91%	0	0
Ribosomal protein S6 kinase alpha 1	90%	22.2	29.3
Bcr/Abl fusion protein	90%	30.2	31.8
Kruppel-like factor 5	89%	26.3	122
Histamine H3 receptor	88%	0	0
Acyl coenzyme A:cholesterol acyltransferase	88%	0	0
Transient receptor potential cation channel subfamily A member 1	87%	1.2	0
Glucose transporter	87%	59.9	41.4
DNA-(apurinic or apyrimidinic site) lyase	85%	174.9	248.3
PI3-kinase p110-delta subunit	83%	6.7	2.2
Beta-glucuronidase	83%	40.8	25.9
Glycine receptor subunit alpha-1	83%	0.1	0
Macrophage migration inhibitory factor	83%	1099.2	744.3
IgG receptor FcRn large subunit p51	82%	0	8.1
Lysosomal Pro-X carboxypeptidase	81%	43.5	50.5
Aminopeptidase N	81%	58.1	0
T-cell protein-tyrosine phosphatase	81%	25.7	19.3
Egl nine homolog 1	80%	28.4	23.8
5'-nucleotidase	80%	45.3	23.2

Table S2. Identified targets for **31** cross the bioinformatics tool Swiss Target Prediction (STP) and transcriptomics data (Protein Atlas). nTPM, normalized Transcripts Per Million.

**31**			
**Target (STP)**	**Probability (STP)**	**nTPM A375 (Protein Atlas)**	**nTPM HaCaT (Protein Atlas)**
Cannabinoid receptor 1	0.0956237870388	3.4	0
Beta amyloid A4 protein	0.0956237870388	300	241.5
Neurokinin 1 receptor	0.0956237870388	0	0
Neurokinin 2 receptor	0.0956237870388	0.4	0.3
Bile acid receptor FXR	0.0956237870388	0.1	0
Vasopressin V1a receptor	0.0956237870388	0	0
Histone deacetylase 1	0.0956237870388	146.6	163.9

Transient receptor potential cation channel subfamily M member 8	0.0956237870388	5.4	0
p53-binding protein Mdm-2	0.0956237870388	41.9	10
Bombesin receptor subtype-3	0.0956237870388	0	0
Vasopressin V2 receptor	0.0956237870388	1.4	0
Voltage-gated potassium channel subunit Kv1.5	0.0956237870388	0	0
Apolipoprotein B-100	0.0956237870388	0	0
Adenosine A1 receptor	0.0956237870388	10.5	0.1
Dipeptidyl peptidase IV	0.0956237870388	0.4	1.0
C-C chemokine receptor type 2	0.0956237870388	0	0.3
Pregnane X receptor	0.0956237870388	0	0
Tachykinin-3	0.0956237870388	0	0
P-glycoprotein 1	0.0956237870388	1.5	0

Table S3. Identified targets for **63** cross the bioinformatics tool Super Pred (SP) and transcriptomics data (Protein Atlas). nTPM, normalized Transcripts Per Million.

**63**				
**Target Name (SP)**	**UniProt ID**	**Probability (SP)**	**nTPM A375 (Protein Atlas)**	**nTPM HaCaT (Protein Atlas)**
Cathepsin D	P07339	99%	141.7	1014.8
Nuclear factor NF-kappa-B p105 subunit	P19838	99%	23.8	24.5
Cyclooxygenase-1	P23219	97%	1.5	6.7
Dual specificity protein kinase CLK4	Q9HAZ1	94%	5.7	9.2
Transcription intermediary factor 1-alpha	O15164	93%	11.9	7.5
Muscarinic acetylcholine receptor M5	P08912	91%	0.2	0
Cathepsin K	P43235	91.2%	15.8	0
Muscarinic acetylcholine receptor M4	P08173	90%	0.1	0.1
Cannabinoid CB2 receptor	P34972	89%	0	0
Kruppel-like factor 5	Q13887	88%	26.3	122
Cystinyl aminopeptidase	Q9UIQ6	87%	7.1	12.7
DNA-(apurinic or apyrimidinic site) lyase	P27695	87%	174.9	248.3
Hypoxia-inducible factor 1 alpha	Q16665	86%	116.6	68
Beta-glucocerebrosidase	P04062	85%	49.7	74.2
G-protein coupled receptor 55	Q9Y2T6	84.8%	1.9	0
GABA-A receptor; alpha-1/beta-2/gamma-2	P14867	85%	0	0.1
Proteasome component C5	P20618	84%	372.9	287.4
LSD1/CoREST complex	O60341	83%	88.8	116.5
Glycine transporter 2	Q9Y345	82%	0	0
Cyclin-dependent kinase 5	Q00535	82%	68.6	22.2

T-cell protein-tyrosine phosphatase	P17706	81.4%	25.7	19.3
Glutamate NMDA receptor; GRIN1/GRIN2B	Q05586	80%	0	0.6

Table S4. Identified targets for **63** cross the bioinformatics tool Swiss Target Prediction (STP) and transcriptomics data (Protein Atlas). nTPM, normalized Transcripts Per Million.

**63**				
**Target (STP)**	**UniProt ID**	**Probability (STP)**	**nTPM A375 (Protein Atlas)**	**nTPM HaCaT (Protein Atlas)**
Cyclooxygenase-1	P23219	0.109339753	1.5	6.7
Nuclear receptor ROR-gamma	P51449	0.109339753	0	0
Metabotropic glutamate receptor 5 (by homology)	P41594	0.109339753	0	0
5-lipoxygenase activating protein	P20292	0.109339753	0	1.1
Sodium channel protein type IX alpha subunit	Q15858	0.109339753	0.1	0
Adenosine A2b receptor	P29275	0.109339753	25.3	56.9
SUMO-activating enzyme	Q9UBE0	0.109339753	95.1	46.7
c-Jun N-terminal kinase 2	P45984	0.109339753	31.8	37.4
Carnitine O-palmitoyltransferase 1, liver isoform	P50416	0.109339753	22.7	88.5
6-phosphofructo-2-kinase/fructose-2,6-bisphosphatase 3	Q16875	0.109339753	54.9	65.3
Neprilysin (by homology)	P08473	0.109339753	0	0
Voltage-gated L-type calcium channel alpha-1C subunit	Q13936	0.109339753	0	0
Norepinephrine transporter	P23975	0.109339753	0.1	8.3
Cytochrome P450 2C19	P33261	0.109339753	0	0
Metabotropic glutamate receptor 2	Q14416	0.109339753	0	0
Adenosine A1 receptor (by homology)	P30542	0.109339753	10.5	0
Puromycin-sensitive aminopeptidase	P55786	0.109339753	32.2	49.2
Cytochrome P450 2C9	P11712	0.109339753	0	0
Fibroblast growth factor receptor 1	P11362	0.109339753	10.2	0
MAP kinase p38 alpha	Q16539	0.109339753	19.7	34
Liver glycogen phosphorylase	P06737	0.109339753	54.7	264.2
Bromodomain-containing protein 3	Q15059	0.109339753	12.6	27.1
Transitional endoplasmic reticulum ATPase	P55072	0.109339753	152.3	65.8
Prostanoid EP1 receptor	P34995	0.109339753	0.1	0
Pyruvate kinase isozymes M1/M2	P14618	0.109339753	1496	2134.1
Multidrug resistance-associated protein 1	P33527	0.109339753	6.9	11.5
Bcl-2-related protein A1	Q16548	0.109339753	6	0.9
Gastrin releasing peptide receptor	P30550	0.109339753	0.2	0.1
Voltage-gated potassium channel, KQT; KCNQ2(Kv7.2)/KCNQ3(Kv7.3)	O43525 O43526	0.109339753	0	0
Translocator protein (by homology)	P30536	0.109339753	132.3	228.9
Delta opioid receptor	P41143	0.109339753	6.8	0.1
Bromodomain-containing protein 2	P25440	0.109339753	24.9	26.6
Adenosine A3 receptor	P0DMS8	0.109339753	0.1	0
Cytochrome P450 3A4	P08684	0.109339753	0.1	0.1
Epidermal growth factor receptor erbB1	P00533	0.109339753	0	185.7
Serine/threonine-protein kinase RAF	P04049	0.109339753	40.4	25.4
LXR-alpha	Q13133	0.109339753	15.2	8.7
Adenosine A2a receptor	P29274	0.109339753	0.2	1
LXR-beta	P55055	0.109339753	79.2	44.3
Vascular endothelial growth factor receptor 2	P35968	0.109339753	2.4	0
Kir3.1/Kir3.2	P48051 P48549	0.109339753	0.4/0.1	0

11-beta-hydroxysteroid dehydrogenase 1	P28845	0.109339753	0	0
Platelet-derived growth factor receptor beta	P09619	0.109339753	1.9	0
Kir3.1/Kir3.4	P48544 P48549	0.109339753	0.1	0.1
Kinesin-1 heavy chain/ Tyrosine-protein kinase receptor RET	P07949	0.109339753	0	0
Corticotropin releasing factor receptor 1	P34998	0.109339753	0	0
Potassium channel, inwardly rectifying, subfamily J, member 11	Q14654	0.109339753	0	0.3
Cathepsin (V and K)	O60911	0.109339753	1.2	63.8
Cyclooxygenase-2	P35354	0.109339753	0	11.6
Heat shock protein HSP 90-alpha	P07900	0.109339753	1280.1	1393
Monoamine oxidase B	P27338	0.109339753	0.7	0
Carboxypeptidase B	P15086	0.109339753	0	0
Bromodomain testis-specific protein	Q58F21	0.109339753	0.2	0.2
GABA-A receptor; alpha-1/beta-2/gamma-2	P14867 P47870 P18507	0.109339753	0	0.1
GABA-A receptor; alpha-5/beta-3/gamma-2	P28472 P18507 P31644	0.109339753		
GABA-A receptor; alpha-3/beta-3/gamma-2	P28472 P34903 P18507	0.109339753		
Isocitrate dehydrogenase [NADP] cytoplasmic	O75874	0.109339753	72.5	96
Hypoxia-inducible factor 1 alpha	Q16665	0.109339753	116.6	68
Glucocorticoid receptor	P04150	0.109339753	17.9	26.6
Elongation of very long chain fatty acids protein 6	Q9H5J4	0.109339753	26.5	23.3
GABA-A receptor; alpha-1/beta-3/gamma-2	P28472 P18507 P14867	0.109339753	0	0
P2X purinoceptor 7 (by homology)	Q99572	0.109339753	0	0.2
Tyrosine-protein kinase JAK3	P52333	0.109339753	0	0
GABA-A receptor; alpha-2/beta-3/gamma-2	P47869 P28472 P18507	0.109339753	0	0
Tyrosine-protein kinase JAK2	O60674	0.109339753	3.8	2.1
Cyclin-dependent kinase 4/cyclin D	P30281 P24385 P11802 P30279	0.109339753	43	38.3
Bradykinin B1 receptor	P46663	0.109339753	0	2.3
Apoptosis regulator Bcl-2	P10415	0.109339753	2	0.4
Protein Mdm4	O15151	0.109339753	16.4	6
Estrogen receptor alpha	P03372	0.109339753	0	0
G protein-coupled receptor 44	Q9Y5Y4	0.109339753	0.1	0
Ghrelin receptor	Q92847	0.109339753	0	0
Muscle glycogen phosphorylase	P11217	0.109339753	0.2	0.1
Cystic fibrosis transmembrane conductance regulator	P13569	0.109339753	0	0
G-protein coupled receptor 55	Q9Y2T6	0.109339753	1.9	0
G-protein coupled receptor 4	P46093	0.109339753	0.1	0
Apoptosis regulator Bcl-W	Q92843	0.109339753	22.1	40.3
3-phosphoinositide dependent protein kinase-1	O15530	0.109339753	12.9	19.1
Tyrosine-protein kinase SRC	P12931	0.109339753	15.4	24
c-Jun N-terminal kinase 1	P45983	0.109339753	11.1	12.4
Casein kinase I delta	P48730	0.109339753	46	42.5
Endothelin-converting enzyme 1	P42892	0.109339753	29.4	36.4
Tyrosine-protein kinase ABL	P00519	0.109339753	30.2	31.8
Bcl2-antagonist of cell death (BAD)	Q92934	0.109339753	60.4	47
Monoamine oxidase A	P21397	0.109339753	2.2	15
Induced myeloid leukemia cell differentiation protein Mcl-1	Q07820	0.109339753	193.5	177

Vascular endothelial growth factor receptor 1	P17948	0.109339753	11.3	0
Beta-chymotrypsin	P17538	0.109339753	0	0
Thromboxane-A synthase	P24557	0.109339753	3	0
Neuronal acetylcholine receptor protein alpha-7 subunit	P36544	0.109339753	0.4	0
Phosphodiesterase 10A	Q9Y233	0.109339753	1.1	0
Glycine transporter 1	P48067	0.109339753	7.9	59
TGF-beta receptor type I	P36897	0.109339753	10.6	28.7
c-Jun N-terminal kinase 3	P53779	0.109339753	3.1	7.2
Serine/threonine-protein kinase B-raf	P15056	0.109339753	6.4	3.2
Cyclin-dependent kinase 5/CDK5 activator 1	Q15078 Q00535	0.109339753	4.5	1.7
Tyrosine-protein kinase LCK	P06239	0.109339753	0	0.1
FK506-binding protein 1A	P62942	0.109339753	335	319.5
Dual specificity phosphatase Cdc25A	P30304	0.109339753	30.9	15.4
Bcl-2-like protein 10	Q9HD36	0.109339753	0	1.6

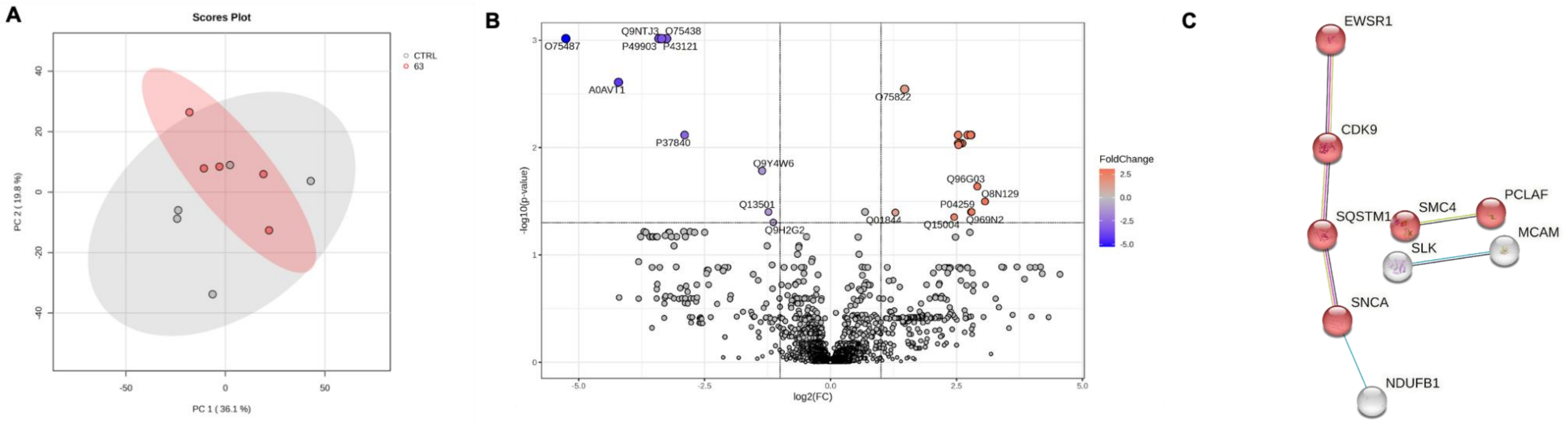

D

Annotated Keywords (UniProt)				
keyword	description	count in network	strength	false discovery rate
KW-0007	Acetylation	14 of 3362	0.52	0.0081

Figure S64 A-D: A) Principal component analysis score plot showing clustering of **63** vs CTRL, B) Differential analysis reporting significant proteins, C) String network and D) Enrichment analysis

Table S5. PDB list of the structures contained in the panel used for IVS.

	1h3x	1klu	1sc8	1xj7	2ado	2eb2	2hzq
1axh	1h3y	1lcl	1shw	1xm2	2ahi	2eb3	2hzr
1b1e	1h7s	1lgv	1si5	1xpa	2ang	2eyv	2i3b
1b1i	1h7u	1lhz	1sj6	1xqg	2ann	2eyw	2i3h
1b1j	1hjb	1ljy	1sm3	1xqj	2anr	2eyx	2i3i
1b3u	1hjc	1luj	1t15	1xtq	2ass	2eyy	2ial
1bbj	1hph	1m5i	1t29	1xtr	2ast	2eyz	2iam
1bd8	1hty	1mp8	1t2u	1xts	2ata	2f4j	2ian
1bj4	1hu9	1mq4	1t2v	1y98	2ayr	2f7e	2ihq
1bor	1hw3	1mqb	1t5z	1ycs	2b1g	2f7x	2ing
1bz7	1hw4	1muo	1t63	1ysg	2b1i	2f7z	2iog
1c5d	1hww	1mzc	1t65	1ysi	2b1v	2fai	2iok
1cl3	1hxk	1n0w	1t73	1ysn	2b9d	2fb8	2itn
1cly	1i37	1n5o	1t74	1ysw	2bdf	2fky	2ito
1clz	1i38	1nh3	1t76	1ysx	2bdg	2fl2	2itp
1cmo	1ig1	1of1	1t79	1ywv	2bdh	2fl5	2itq
1d9j	1igt	1orn	1t7f	1yxs	2bdi	2fl6	2itt
1dr1	1ihb	1orp	1t7m	1yxt	2bdj	2fnb	2itu
1dyr	1io4	1oz2	1t7r	1yxu	2boa	2g15	2itv
1e3g	1iol	1ozo	1t7t	1yxv	2bvb	2g1t	2itw
1e3k	1isn	1p59	1t95	1yxx	2by4	2g2f	2itx
1e3m	1j2l	1p9o	1tfq	1z23	2c6c	2g2h	2ity
1e50	1jf1	1pk5	1tft	1z5m	2c6g	2g2i	2itz
1ea6	1jht	1psk	1tvb	1z8g	2c6p	2g57	2iu0
1ean	1ji7	1q1v	1tvh	1z95	2ceq	2gd8	2iu3
1eao	1jih	1q8f	1uol	1zca	2cer	2ggm	2j2s
1eaq	1jkk	1qrd	1uv0	1zcb	2ces	2ghg	2j6m
1ec6	1jkl	1qym	1uwh	1zck	2cet	2gjj	2jm1
1f08	1jks	1r24	1uwj	1zcl	2cgn	2gv6	2jrw
1fn4	1jkt	1r9x	1v3a	1zky	2cgo	2gv7	2jxd
1gr3	1jm7	1r9y	1vcb	1zms	2cgy	2h8h	2jye
1gs4	1jnx	1r9z	1vj9	1zr0	2cgz	2hd6	2jyt
1gux	1jpp	1ra0	1vja	1ztp	2cw9	2hr9	2jyu
1h0d	1jsg	1ra5	1wch	1zuc	2d1x	2hyy	2jyv
1h3t	1jxv	1rak	1wq1	1zxt	2dvj	2hz0	2kgg
1h3u	1k2a	1rts	1x24	221p	2dx1	2hz4	2kgi
1h3v	1khx	1rv1	1x2j	2a4j	2eak	2hzi	2kkf
1h3w	1klg	1s3k	1x2r	2aab	2eal	2hzn	2knh

2kt4	2oxz	2vth	2ybp	3c4e	3ei4	3l3m	3nal
2kvm	2oy2	2vti	2ybs	3c4f	3eqb	3l3x	3nam
2kyg	2oy4	2vtj	2yc0	3c5r	3eto	3l3z	3nan
2l4s	2pe4	2vtl	2yde	3c7q	3f3v	3l6f	3nax
2l4t	2pkg	2vtm	2yer	3cbm	3f3w	3l8i	3nfk
2l4z	2pnx	2vtn	2yex	3cbo	3f81	3l8j	3nfl
2l7u	2pz1	2vto	2yhd	3cbp	3fc2	3l95	3nzs
2ld1	2q3n	2vtp	2yjw	3cc5	3fcx	3l9l	3nzu
2ldr	2q7i	2vtq	2yjx	3cch	3ffd	3l9m	3o0i
2lwl	2q7j	2vtr	2yk2	3cga	3fw1	3l9n	3o2f
2m1r	2q7k	2vts	2yk9	3ch1	3g0e	3l9p	3o33
2m6y	2q7l	2vtt	2ykb	3cjf	3g0f	3laq	3o34
2m86	2qbx	2vu3	2ykc	3cjg	3g5d	3lbz	3o35
2mbe	2qdj	2vuk	2yke	3cjo	3g77	3lcs	3o36
2mjv	2qic	2vur	2yki	3crv	3gl6	3lct	3o37
2mnw	2qj9	2vxd	2ykj	3crw	3gop	3le6	3o5x
2mqs	2qja	2vxj	2ypr	3cs9	3grw	3lj3	3oaw
2msr	2qjb	2wbs	2zeu	3cth	3guq	3llp	3og7
2mtn	2qms	2wbu	2zev	3ctj	3h3b	3lok	3omv
2mwt	2qp6	2wh6	2zv7	3cwe	3hb4	3lq8	3omz
2n12	2qxs	2wnd	2zv8	3d14	3hb5	3m11	3oni
2nyk	2qzo	2wor	2zv9	3d15	3hg1	3m1j	3os8
2o1y	2rd0	2wos	2zva	3d25	3hkn	3map	3os9
2o21	2rfn	2wqe	3a3z	3dbs	3hkq	3mar	3osa
2o22	2rfs	2wtf	3a40	3ddf	3hkt	3mas	3ow3
2o2f	2rkb	2wxv	3a99	3ddg	3hku	3mfh	3ow4
2o2m	2rku	2x0u	3acl	3dkc	3hl5	3mfi	3owj
2o2n	2rl5	2x0v	3aox	3dkf	3hpj	3mjw	3owk
2o3p	2rrf	2x0w	3b24	3dkg	3i6u	3mr2	3owl
2o63	2tsr	2x4z	3b25	3dvh	3i6w	3mr3	3oxz
2o64	2uzi	2xd6	3b26	3dvp	3iai	3mr5	3oy3
2o65	2uzr	2xef	3b27	3dvt	3iay	3mr6	3oyw
2o8b	2uzs	2xeg	3b28	3dyf	3ik3	3mxf	3p5a
2o8c	2v1y	2xei	3b5h	3dyg	3inm	3myj	3p7z
2o8d	2v6q	2xej	3b9s	3dyh	3iwy	3n1v	3p92
2o8e	2vbj	2xk5	3bcm	3e78	3k5v	3n1w	3p95
2o8f	2vbl	2xmy	3bco	3e79	3kf4	3n3l	3pe1
2obh	2vbn	2xnb	3bcp	3e8n	3kfa	3n45	3pe2
2oh0	2vbo	2xne	3bdy	3eca	3klm	3n46	3peg
2ojf	2vgo	2xng	3be1	3ee5	3kr3	3n49	3pg7
2opm	2vhf	2xpx	3be9	3efq	3kxg	3n5e	3phu
2ovh	2vl7	2xru	3bz3	3egt	3kxh	3n5g	3phw
2ovm	2vr9	2xxn	3c1v	3ei1	3kxm	3n5h	3phx
2oxu	2vra	2yac	3c1x	3ei2	3kxn	3n5j	3pom
2oxw	2vta	2ybk	3c4c	3ei3	3l3l	3n6k	3poz

3pp0	3r4m	3sa0	3vrz	3zjg	4bic	4cqh	4esg
3pp1	3r4n	3sbd	3vs0	3zm0	4bid	4crl	4esr
3pse	3r4o	3sbe	3vs1	3zm1	4bie	4d0w	4eu2
3pt2	3r4p	3sd5	3vs2	3zm2	4bjx	4d0x	4ew1
3puq	3r8u	3si8	3vs3	3zm3	4bnc	4d1s	4ew2
3pur	3r8v	3skj	3vs4	3zp5	4boq	4d8x	4ew3
3pvg	3r8z	3sl9	3vs5	3zyu	4bos	4d8y	4ewr
3pxa	3r92	3sla	3vs6	421p	4bou	4da0	4f0i
3pxb	3r9d	3smb	3vs7	4ag8	4boz	4da3	4f1s
3pxc	3r9h	3smc	3vu7	4agc	4bqg	4da6	4f33
3pxd	3r9n	3sqq	3vuw	4agd	4bqj	4da7	4f3f
3pxe	3r9o	3ss3	3vux	4aif	4by3	4da8	4f52
3q96	3rah	3ss4	3vuy	4ajw	4c13	4dab	4fc0
3qaz	3rak	3ss5	3w32	4anm	4c33	4dae	4fjo
3qb1	3ral	3swz	3w33	4apo	4c34	4dan	4fk3
3qbr	3rev	3sx0	3w8h	4asd	4c35	4daq	4fnw
3qdg	3rew	3t04	3w8i	4ase	4c36	4dar	4fnx
3qdj	3rhk	3t1i	3wa0	4asz	4c37	4dbs	4fny
3qdm	3rjc	3tdd	3wdz	4at3	4c38	4dbu	4fnz
3qeq	3rk5	3tg4	3wgw	4at4	4c6b	4dbw	4fzr
3qeu	3rk7	3tg5	3wha	4at5	4c6c	4dem	4g11
3qfq	3rk9	3th5	3wig	4avp	4c6d	4dl2	4g2t
3qfv	3rkb	3tku	3wme	4aw5	4c6e	4dl3	4g9j
3qi5	3rkz	3tt0	3wmf	4awo	4c6f	4dl4	4gcj
3qqk	3rlj	3twj	3wmg	4awp	4c6i	4dl5	4gg5
3qri	3rll	3tzb	3wmu	4awq	4c6j	4dl6	4gg7
3qrj	3rmf	3u7d	3wmv	4axa	4c6k	4dl7	4gk7
3qrk	3rni	3u9u	3wsq	4b0g	4c6l	4dm4	4gkn
3qti	3rpr	3ue4	3wzz	4b18	4c6m	4dqm	4gks
3qtq	3rpv	3ug1	3x01	4b2z	4c6n	4dxa	4gmb
3qtr	3rpy	3ug2	3x02	4b6u	4c6o	4e26	4gmx
3qts	3rsr	3uli	3x03	4b6v	4c6p	4e28	4gos
3qtu	3rtq	3umw	3x04	4b7p	4c6q	4edu	4gov
3qtw	3ruk	3upa	3x05	4bb4	4c7t	4edw	4goy
3qtx	3rvg	3upc	3x06	4bbe	4c9w	4edx	4gp0
3qtz	3rzb	3uw4	3x07	4bbf	4c9x	4eey	4gp3
3qu0	3s00	3uw5	3x08	4bbg	4cfq	4egc	4gpi
3qum	3s0o	3uyh	3x09	4bcb	4cfr	4eqc	4gpq
3qux	3s1b	3v3v	3x0a	4bcc	4cgb	4ern	4gpt
3quy	3s1h	3v42	3x0b	4bcd	4cgc	4erq	4gpz
3quz	3s1k	3vfg	3x0c	4bf2	4cjx	4ery	4gq3
3qxp	3s7b	3vgo	3zbf	4bfr	4ckr	4erz	4gq4
3r0t	3s7d	3vjn	3zjd	4bgh	4cmh	4es0	4gq6
3r21	3s7f	3vjo	3zje	4bhn	4co8	4es8	4gt4
3r22	3s7j	3vry	3zjf	4bib	4cq0	4es9	4gt5

4gwg	4jfo	4l06	4m9j	4ofu	4q06	4rfw	4uak
4gwk	4jfp	4l3p	4m9l	4ogh	4q07	4rht	4ual
4gzl	4jfq	4l52	4m9n	4ogi	4q08	4rhu	4uaw
4gzm	4jgv	4l53	4ma4	4ogj	4q09	4rhx	4uay
4h58	4ji9	4l8g	4man	4ogn	4q0l	4rhy	4uaz
4hra	4jia	4l9s	4mdr	4ogt	4q18	4riu	4ub1
4hrf	4jol	4l9w	4mi0	4ogv	4q19	4riv	4ub2
4hvs	4jv6	4ldq	4mkc	4oh5	4q1a	4rj3	4ub3
4hw7	4jv8	4ldr	4mx3	4oh6	4q1b	4rj4	4ub4
4i1p	4jvb	4lge	4n0s	4oha	4q1c	4rj5	4ub5
4i1z	4jvf	4lgu	4n1t	4oil	4q1d	4rj6	4ubb
4i20	4jvg	4liq	4n1u	4oiu	4q1e	4rj7	4ubc
4i21	4k3j	4ll0	4n1z	4oj9	4q1f	4rj8	4uf0
4i22	4k94	4lmn	4n4g	4ojb	4q8e	4rlk	4uip
4i23	4k9e	4lmq	4n4h	4ojn	4q8f	4rll	4umx
4i24	4k9g	4ln0	4n4i	4ok1	4q9v	4rmc	4umy
4i51	4kcg	4lo9	4n5g	4okb	4qg6	4rmd	4up4
4ian	4kch	4loe	4n5t	4okn	4qg8	4rme	4uuv
4iea	4kci	4lof	4n7v	4okt	4qg9	4rpp	4uv7
4ifc	4kcj	4lpk	4n7z	4okw	4qgc	4rqk	4uwf
4igu	4kck	4lqg	4n8r	4okx	4ql1	4rqv	4uwg
4ihl	4kcl	4lqm	4n9j	4oli	4ql3	4rrv	4uwh
4iir	4kcm	4lrm	4nrv	4olm	4qqc	4rt7	4uwk
4ijp	4kcn	4lrw	4nrw	4oq3	4qsm	4rvk	4uwl
4ikp	4kco	4lts	4nus	4ove	4qt0	4rvl	4uya
4ipf	4kcp	4luc	4o0r	4oyd	4qxo	4rvm	4uyn
4irj	4kcq	4lv6	4o0t	4p59	4qxp	4s0u	4uzd
4irs	4kcr	4lvt	4o0v	4p7a	4qxq	4tnt	4uzh
4is6	4kcs	4lwt	4o0x	4pbd	4qxr	4tq9	4v25
4isk	4kfp	4lwu	4o0y	4pck	4qxw	4tqa	4v26
4j8m	4kjy	4lww	4o1u	4pdz	4r3p	4tup	4wa7
4j8n	4kk0	4lxd	4o1v	4pe0	4r3r	4tuq	4wa9
4ja8	4kk1	4lyf	4o1x	4pe1	4r5s	4tur	4wb5
4jbo	4knb	4lyh	4o2p	4pe4	4r5y	4tus	4wb6
4jbp	4kpw	4lyj	4o97	4pe7	4r6v	4tv8	4wb7
4jbq	4kv1	4m1o	4o9v	4pgz	4r7a	4tv9	4wb8
4jc3	4kv4	4m1s	4occ	4pl3	4r7h	4tvq	4whf
4jdd	4kvp	4m1t	4od2	4pl4	4r7i	4twp	4whg
4je4	4kwp	4m1w	4ode	4pl5	4ra0	4tyl	4wj5
4jef	4kzi	4m1y	4odf	4pqw	4re1	4tz2	4wj7
4jeg	4kzj	4m21	4oea	4px9	4re8	4tz8	4wn5
4jfd	4kzm	4m22	4oed	4pxa	4ree	4tzi	4wnx
4jfe	4kzo	4m6r	4oey	4pyx	4ref	4u0i	4wq6
4jff	4l03	4m9g	4oez	4pyy	4rfc	4u7l	4wt2
4jfh	4l04	4m9h	4ofr	4pzh	4rfd	4u7m	4wuy

4wv8	4zph	5ap7	5d11	5ew8	5ggr	5hni	5jlb
4wvs	4zpk	5awx	5d12	5eyo	5ggs	5ho6	5jle
4wvt	4zpr	5ax9	5d41	5f1h	5ggt	5hoa	5jlj
4wvu	4zqd	5ayx	5d68	5f1k	5ggu	5hor	5jog
4wwq	4zxt	5ayy	5d7a	5f1l	5ggv	5hu9	5joh
4x1i	4zy3	5ayz	5da3	5f1o	5ggz	5hyn	5jrq
4x1k	4zzx	5b6g	5dag	5f21	5gix	5i4z	5jsb
4x1y	4zzy	5b73	5dah	5f25	5gnk	5i50	5jsm
4x20	4zzz	5bnj	5db0	5f2e	5grj	5i6v	5jsn
4x5u	521p	5bol	5db1	5f2p	5gs4	5i95	5jt2
4x60	5a00	5bom	5db2	5f5z	5gsa	5i96	5jtc
4x61	5a1f	5bpc	5db3	5f60	5gtr	5ibm	5jvd
4x63	5a3n	5c5s	5de1	5f61	5gty	5ibs	5jwm
4x6s	5a3p	5c67	5dg5	5f62	5gwn	5icp	5jyo
4x8y	5a3t	5c8k	5dls	5f63	5gzb	5id7	5jyp
4xcu	5a3w	5c8m	5drw	5f8s	5h08	5id9	5jzj
4xi3	5a4k	5c8n	5drx	5f8w	5h24	5idn	5jzn
4xl1	5a7n	5c9c	5dwr	5f8y	5h25	5idp	5jzv
4xlw	5a7o	5cal	5dx4	5f90	5h2u	5ifh	5k22
4xmo	5a7p	5can	5dzl	5fa5	5h65	5igk	5k4i
4xp7	5a7q	5cao	5e1s	5fed	5h7o	5igl	5k4j
4xyf	5a7s	5cap	5e28	5fee	5ha9	5igm	5k72
4xyj	5a7w	5caq	5e2k	5feq	5hbe	5ij7	5k75
4xyk	5a80	5cas	5e2s	5fgk	5hbh	5ij8	5k76
4y18	5a9u	5cau	5e7i	5fi4	5hbj	5imx	5k7g
4y2g	5aa8	5cav	5e7j	5fkj	5hbt	5in9	5k7i
4y5o	5aa9	5ceh	5e7m	5fl4	5hbv	5ipj	5k7y
4y7r	5aaa	5cma	5e8n	5fl5	5hcx	5iwl	5kcc
4yj2	5aab	5coi	5e8o	5fl6	5hcy	5iz6	5kcd
4yj3	5aac	5cp5	5e8p	5flf	5hcz	5iz8	5kce
4yj4	5aau	5cpe	5ea0	5fpu	5hd4	5iz9	5kcf
4yml	5aav	5cqt	5eam	5fpv	5hd7	5iza	5kct
4yqm	5acc	5crm	5eap	5fto	5hg5	5j17	5kcu
4yqu	5aep	5crz	5ear	5ftq	5hg7	5j18	5kcv
4yqv	5afv	5cs8	5ehr	5fun	5hg8	5j2r	5kcw
4ywp	5afw	5csw	5eob	5fup	5hg9	5j3x	5kd9
4z55	5aiz	5csx	5er4	5fv3	5hi2	5j41	5ke0
4z93	5aj1	5ctl	5er5	5fwj	5hib	5j6r	5kit
4zao	5ap0	5cuh	5ety	5g1d	5hic	5j7f	5kj2
4zau	5ap1	5cwz	5eu1	5g1e	5hid	5j7g	5kkr
4zc9	5ap2	5cy9	5eu3	5g1x	5hie	5j9s	5kl2
4zfo	5ap3	5czv	5eu4	5g55	5hiu	5j9y	5kl3
4zjs	5ap4	5czx	5eu5	5g5j	5hls	5j9z	5kl4
4zjv	5ap5	5d0c	5eu6	5gg4	5hlw	5jjm	5kl5
4zp4	5ap6	5d10	5ew3	5ggq	5hm0	5jjy	5kl6

5kl7	5lge	5n6r	5opk	5tlx	5u2e	5vcj	5xsr
5kpk	5lhd	5n7b	5opl	5tly	5u2f	5veb	5xst
5kpl	5lia	5n7e	5opm	5tm1	5u2m	5vfc	5xsu
5kpm	5lk4	5n91	5opn	5tm2	5u2n	5vkc	5xwr
5kr9	5lof	5n9c	5opo	5tm3	5uab	5vnd	5xxh
5kra	5lqb	5n9p	5opp	5tm4	5uad	5vtb	5y0c
5krc	5lrq	5naj	5oq4	5tm5	5ufp	5vxr	5y25
5krf	5ls6	5nbf	5ote	5tm6	5ufw	5vxz	5y2f
5krh	5lsp	5nbx	5otf	5tm7	5ufx	5w0g	5y3n
5kri	5lxq	5nc2	5sb8	5tm8	5ug8	5w0h	5y3o
5krj	5m4e	5nc7	5sb9	5tm9	5ug9	5w2j	5y4m
5krk	5m4h	5ncf	5sba	5tml	5uga	5w89	5y8c
5krl	5m4k	5ncg	5sbb	5tmm	5ugb	5w8f	5y8w
5krm	5m4m	5ncp	5sbc	5tmo	5ugc	5w9c	5y8y
5kro	5m4n	5nd0	5sbd	5tmq	5ujb	5w9d	5y8z
5kup	5m4p	5ndu	5sbe	5tmr	5upe	5wa1	5y93
5kzi	5m5a	5neg	5sun	5tms	5upf	5wbh	5y94
5l0q	5m6a	5nge	5svf	5tmt	5uqv	5wbi	5y9t
5l3e	5m6i	5ngf	5svi	5tmu	5uqw	5wbj	5yb1
5l3f	5m76	5nhy	5svn	5tmv	5uqx	5wbk	5yde
5l3g	5maf	5njj	5svo	5tmw	5us4	5wbl	5ydf
5l4z	5mag	5njk	5svx	5tmz	5usj	5wbu	5ye3
5l50	5mah	5nnc	5svy	5tn1	5uut	5wby	5ye4
5l57	5mai	5nnd	5t1z	5tn3	5uuy	5wg8	5yql
5l58	5mar	5nne	5t31	5tn4	5uv6	5wj6	5yqm
5l9b	5mhq	5nnf	5t4u	5tn5	5ux6	5wma	5yqn
5l9r	5mkv	5nng	5t4v	5tn6	5uxh	5wmd	5yqo
5l9v	5mle	5nqr	5t7x	5tn7	5uy8	5wmg	5yqx
5la9	5mmz	5nwh	5t92	5tn8	5uy9	5wr7	5yso
5lap	5mo4	5nx1	5t97	5tr6	5uys	5wrv	5yu9
5las	5mq5	5nx3	5tab	5trm	5uz0	5wrw	5yuf
5lat	5mra	5o22	5tbm	5tru	5v21	5wt9	5yvn
5lb6	5mrd	5o28	5tbn	5tt7	5v22	5wuu	5yvo
5lbb	5mtl	5o2a	5th6	5twl	5v37	5wzz	5yx2
5lbc	5mud	5o4g	5th9	5twu	5v3h	5x02	5z2s
5lbe	5muh	5o4o	5tld	5twy	5v3o	5x0i	5z2t
5lbf	5mvg	5o7a	5tlf	5twz	5v3r	5xaf	5za7
5lbq	5my6	5o7i	5tlg	5tx3	5v43	5xag	5za8
5lbw	5n1v	5o7p	5tll	5tz2	5v4e	5xf3	5za9
5lby	5n1x	5oai	5tlm	5tzt	5v5n	5xf4	5zae
5lbz	5n1z	5oba	5tlo	5tzu	5v7i	5xf5	5zaf
5lde	5n20	5obb	5tlp	5u28	5v9u	5xf6	5zag
5lds	5n21	5oc7	5tlt	5u2b	5vaa	5xg5	5zah
5lg6	5n5j	5oc8	5tlu	5u2c	5val	5xgh	5zaj
5lga	5n5k	5ok8	5tlv	5u2d	5vam	5xnv	5zc5

5zev	6axd	6bv2	6d7o	6dxg	6fib	6gqq	6iar
5zgg	6b0f	6bv3	6d7p	6dxk	6fl5	6gva	6ias
5zib	6b0o	6bv4	6d7q	6dxl	6frj	6gvw	6ic2
5zic	6b0p	6bvb	6d7s	6e1a	6fs0	6gy2	6ic3
5zma	6b0q	6bw3	6d7t	6e4f	6fs1	6gzd	6ice
5zr3	6b0r	6bw4	6d7v	6e5n	6fs2	6gzh	6idx
5ztn	6b0v	6byr	6d7x	6e6j	6fwk	6gzm	6ie1
5zv2	6b0y	6bys	6d88	6ef4	6fy4	6h1d	6ie2
5zwj	6b4l	6byy	6d8e	6eny	6fzf	6h1e	6ie3
5zxb	6b4u	6bz1	6d8z	6eqa	6fzg	6h3c	6igd
621p	6b4w	6c2r	6dbp	6eqb	6fzj	6h46	6ige
6a1c	6b7g	6c2t	6dd3	6et4	6fzp	6h47	6igf
6a38	6b9y	6c3l	6ddh	6etj	6fzy	6h4h	6imq
6a3a	6b9z	6c3n	6ddi	6ew6	6g0a	6h4i	6io0
6a3b	6ba2	6c42	6ddj	6ew7	6g0o	6h4j	6itj
6a3c	6ba4	6c48	6ddx	6ew8	6g0p	6h4k	6iuo
6a3e	6bae	6c56	6ddy	6exw	6g0q	6hax	6iup
6a6k	6bah	6c57	6ddz	6ey2	6g0r	6hay	6iur
6a71	6bat	6c59	6de0	6f0f	6g0s	6haz	6iwd
6a72	6bau	6c8c	6de1	6f0g	6g1l	6hby	6iwq
6a76	6bav	6cb8	6de2	6f3d	6g1u	6hcz	6iwr
6a77	6bba	6cbz	6de3	6f3e	6g1v	6hd4	6izq
6a78	6bev	6ccy	6dg5	6f3g	6g1w	6hd6	6j1l
6a79	6bg3	6cdy	6dg6	6f3i	6g2u	6hhf	6j6m
6a8n	6bg5	6cen	6djc	6f57	6g3j	6hho	6j71
6abo	6bit	6cg1	6dl7	6f7b	6g3k	6hig	6jbt
6adg	6blw	6cg2	6dna	6fbn	6g3o	6hk2	6jjp
6adi	6bmi	6chw	6dnb	6fbo	6g40	6hp9	6jke
6afr	6bmr	6chz	6dnd	6fcb	6g6r	6hps	6jn2
6agk	6bmu	6cj9	6dne	6fcd	6g76	6hr2	6jpe
6agp	6bmv	6ckn	6duk	6fek	6g77	6htf	6jpv
6ak2	6bmw	6cko	6dum	6ffj	6g78	6hud	6jq2
6akw	6bmx	6ckx	6dun	6fg6	6gcr	6hxw	6jq3
6amb	6bmy	6cm2	6dw3	6fgb	6gcu	6hyg	6jrp
6arj	6bnk	6co4	6dw4	6fgf	6gcw	6i04	6jtn
6arv	6bnl	6cq1	6dw5	6fgg	6gcx	6i07	6jto
6asr	6bof	6ct2	6dw7	6fgh	6gfa	6i0j	6jtp
6at5	6br1	6cu6	6dwd	6fgi	6gk0	6i0l	6jz0
6at6	6brf	6cxs	6dwj	6fgl	6gou	6i1s	6k04
6att	6brr	6cz2	6dwk	6fgt	6gqj	6i41	6k05
6avf	6bry	6cz3	6dwx	6fgu	6gqk	6i5p	6k0j
6avg	6bs2	6cz4	6dx1	6fgv	6gql	6i68	6k0y
6avj	6buy	6czu	6dx2	6fgw	6gqm	6i7a	6k39
6ax6	6bv0	6czv	6dx3	6fh6	6gqo	6i8a	6k7o
6ax7	6bv1	6d5y	6dx5	6fh7	6gqp	6iap	6k9g

6k9h	6mak	6nsk	6p4y	6rpu	6u6v	6vua	6x5e
6kc5	6mau	6ntc	6p50	6rrc	6u85	6vwu	6x5m
6kc6	6mbw	6ntd	6p5v	6rrk	6ubw	6vzy	6x5n
6kft	6mbz	6ntu	6p5w	6rti	6ud2	6w0q	6x9i
6kg2	6mck	6nui	6p67	6s4a	6udi	6w2p	6x9j
6kla	6md7	6nuq	6p7g	6s4e	6udt	6w3l	6x9k
6kn5	6mdb	6nyb	6pbc	6s4f	6udu	6w3n	6xcg
6knr	6mep	6nyh	6pc4	6s8v	6udv	6w3q	6xcv
6kqp	6mfq	6o0k	6pgo	6s9b	6udx	6w3u	6xer
6kqq	6mnl	6o0l	6pgp	6s9c	6udy	6w43	6xes
6kro	6mnx	6o0m	6pgx	6s9d	6ug7	6w51	6xet
6ktr	6mo7	6o0o	6pkc	6sbo	6ug8	6w7f	6xf3
6kz5	6mo8	6o0p	6pp9	6scm	6ug9	6w7g	6xf4
6kz7	6mo9	6o39	6ppd	6ses	6uga	6wa2	6xfp
6l30	6moa	6o3a	6ppi	6sfr	6ujo	6wak	6xjp
6l4b	6mob	6o3b	6pq3	6slg	6ujq	6wdp	6xjr
6l4g	6mom	6o4y	6psj	6ssh	6uk2	6wdq	6xjs
6l4h	6mp0	6o4z	6ptb	6sua	6uk4	6we2	6xjt
6l8u	6mp1	6o51	6pte	6suu	6urc	6we3	6xju
6l94	6mry	6o53	6pxu	6sxa	6uup	6we4	6xk9
6l9k	6mt0	6o5i	6py8	6sxb	6uy3	6wgh	6xkd
6l9l	6mvl	6o96	6q0j	6szm	6v3w	6whb	6xl4
6l9m	6mwe	6o9x	6q0k	6t2g	6v5l	6wjf	6xlo
6l9n	6mx8	6o9y	6q0t	6t2w	6v65	6wjg	6xlq
6lf1	6n6o	6oa0	6q6f	6t3b	6v6f	6wjh	6xlv
6lf2	6ne5	6oa1	6qb5	6t3c	6v8t	6wjl	6xlw
6lk0	6njs	6oa3	6qbt	6t3i	6vaj	6wmw	6xlx
6lk5	6nlv	6oak	6qbv	6t5b	6vbh	6wnh	6xog
6lk6	6nm0	6oal	6qbw	6t5u	6vc2	6wqu	6xoh
6llc	6nmr	6ob2	6qpo	6t5v	6vc9	6wr0	6xoi
6llx	6nms	6ob3	6qqn	6t6d	6vca	6wr1	6xvt
6lox	6nmt	6oge	6qtg	6t7u	6vil	6wtn	6xxn
6lr9	6nmu	6oia	6qtj	6t9z	6vkk	6wto	6xxo
6ls4	6nmv	6oim	6qu7	6tde	6vko	6wtp	6xxp
6lt8	6nmw	6ony	6r0k	6te5	6vkq	6wtq	6xxr
6lth	6nna	6op9	6r3s	6tgw	6vnd	6wu8	6y90
6ltj	6nng	6opd	6r7o	6th7	6vpf	6wuw	6y92
6lyc	6no9	6oqi	6r9h	6tuy	6vqn	6wvz	6y97
6lzi	6npi	6oql	6rcf	6txz	6vqo	6ww0	6y9a
6lzj	6npm	6oqo	6rcj	6u4j	6vr1	6wxk	6y9b
6lzk	6npp	6osp	6rd2	6u63	6vr5	6wxn	6yaa
6lzp	6nqm	6otn	6rfi	6u64	6vrm	6wyr	6yat
6m7x	6nqu	6ovg	6rfj	6u65	6vrn	6wyt	6yb8
6m8q	6nr5	6p05	6rlc	6u67	6vtc	6wzw	6yb9
6maj	6nsj	6p3d	6rmj	6u6f	6vth	6x0p	6yhk

6yhl	6ztd	7akf	7bir	7df1	7jhd	7khj	7ll8
6yhm	6ztf	7akh	7bit	7dha	7jjl	7khk	7ll9
6yhn	6ztr	7aki	7biv	7dhl	7jkw	7khl	7lqd
6yhr	6zvf	7akl	7bj0	7dhs	7jky	7kib	7lrz
6yi8	721p	7al7	7bj6	7dhy	7jkz	7kic	7ls0
6yid	7a1w	7auv	7bm9	7dhz	7jm5	7kid	7lsz
6yio	7a1x	7avi	7bmc	7dmc	7jni	7kjs	7lt0
6yj3	7a1y	7avl	7bmg	7dt2	7jnr	7kk2	7ltt
6ykc	7a47	7avq	7bxa	7dv4	7jnv	7kk3	7ltx
6ykh	7a4o	7avs	7c06	7dw5	7jnw	7kk4	7lu5
6yo2	7a4r	7avt	7c07	7dy7	7jnx	7kk5	7lxl
6yo4	7a4s	7avu	7c08	7dyt	7jnz	7kk6	7lya
6yo7	7a4w	7avv	7c2z	7dyu	7jo0	7kkh	7lyb
6yoi	7a4z	7awe	7c4o	7dyv	7jo1	7kkm	7lyc
6yok	7a51	7axr	7c6p	7dyw	7jo2	7kkn	7lz4
6yol	7a52	7b46	7c7f	7dyx	7jo3	7kko	7m4t
6ypw	7a53	7b47	7c7g	7e74	7job	7kkp	7mce
6yqn	7a55	7b48	7c7h	7e8d	7joc	7kkq	7mcf
6yqo	7a5b	7b4b	7c88	7e9b	7jto	7knw	7mdn
6yqp	7a5d	7b4c	7cbz	7ear	7jtp	7knx	7mdp
6yr8	7a5l	7b4d	7cc2	7eax	7ju5	7kp6	7mfc
6yxo	7a5m	7b4e	7cfz	7eca	7ju6	7kpg	7mfd
6yxp	7a5n	7b4f	7cgw	7edp	7jvm	7kql	7mfe
6yxq	7a6i	7b4g	7chm	7ef8	7jvn	7kvx	7mff
6yxw	7a6j	7b4h	7chn	7ef9	7jws	7kvz	7mj6
6z1i	7a6k	7b4n	7cht	7efa	7jwt	7kw1	7mj7
6z1o	7a8p	7b7s	7cil	7eif	7jwu	7kxi	7mj8
6z45	7a8r	7b85	7cja	7ejv	7jwv	7l05	7mj9
6zec	7aj2	7bbg	7ckk	7ekn	7jww	7l0f	7mja
6zie	7aj4	7bcu	7clh	7enn	7jx9	7l0g	7mju
6zio	7aj5	7bcx	7cmw	7eod	7jxh	7l1b	7mk7
6zir	7aj7	7bcy	7cnl	7eoy	7jxq	7l1c	7moc
6ziz	7aj8	7bd0	7cqe	7ep6	7k0v	7l1d	7mp5
6zj0	7aja	7bd7	7cqz	7epu	7k1s	7l1x	7mp6
6zl1	7ajm	7bdm	7cro	7eqv	7kbs	7l5e	7mr5
6zl3	7ajs	7be3	7crp	7evj	7kc0	7l9m	7mr6
6zl5	7ajv	7bed	7crq	7ewh	7kcc	7la9	7mr7
6zli	7ajw	7bfz	7crr	7exp	7kce	7law	7mr9
6zmm	7ajy	7bhr	7ctp	7f09	7kcf	7len	7mra
6znc	7ak0	7bhs	7cv3	7f2x	7kda	7lfr	7mrb
6zoq	7ak1	7bht	7cv4	7f7w	7kdb	7lfs	7mrc
6zor	7ak2	7bhu	7czd	7f9w	7kgy	7lg8	7mrd
6zos	7aka	7bhv	7d4b	7fai	7kgz	7lgs	7mrg
6zqk	7akb	7bhw	7d8r	7faj	7khg	7lir	7mrh
6ztb	7ake	7bhx	7d8s	7fdj	7khh	7ljx	7mrj

7msa	7o83	7psj	7r7r	7seg
7msb	7o88	7psk	7ra5	7ser
7msd	7ob9	7puu	7rct	7ses
7mx7	7oba	7puv	7re7	7sfx
7mxa	7obb	7puw	7re8	7sgl
7mxc	7obg	7q0c	7re9	7sj4
7mxn	7obh	7q0d	7ren	7ssm
7n0a	7ocv	7q0e	7rgg	7st8
7n5u	7ok3	7q33	7rgx	7su0
7n5v	7ok4	7q5v	7rgy	7su1
7n5w	7ols	7q5x	7rgz	7su3
7n6z	7olv	7q6t	7rh0	
7n71	7olx	7q6u	7rh1	
7n7j	7om4	7q6v	7rh2	
7n7k	7om5	7q6w	7rh3	
7n7l	7oo7	7q85	7rh4	
7n7m	7opm	7q98	7rjf	
7n7n	7opo	7q99	7rm4	
7n7o	7p3i	7q9a	7rp2	
7n80	7p3v	7qga	7rp3	
7n81	7p9u	7qgl	7rp4	
7n91	7p9v	7qgm	7rrx	
7n93	7pcd	7qgo	7rry	
7n9g	7pdj	7qjg	7rrz	
7ng7	7pdz	7qju	7rs0	
7ni4	7pel	7qk0	7rs1	
7ni5	7pg5	7qk4	7rs2	
7ni6	7pg6	7qk8	7rs3	
7nqq	7phr	7qpj	7rs4	
7nqw	7pjm	7qtw	7rs7	
7nr3	7pjn	7qtx	7rs8	
7nr4	7pll	7qvj	7rs9	
7nr5	7pom	7qvl	7rw5	
7nr8	7pp9	7r03	7rw7	
7nr9	7ppf	7r04	7rwg	
7nsl	7ppg	7r0m	7rwh	
7ny8	7pph	7r0n	7rx9	
7nzn	7ppi	7r0q	7s0u	
7o2i	7pr7	7r3x	7s1p	
7o3i	7pr8	7r3y	7s1q	
7o4p	7pr9	7r75	7s1r	
7o70	7prb	7r7d	7s1s	
7o7i	7prt	7r7i	7s2y	
7o7j	7psh	7r7k	7s4g	
7o7k	7psi	7r7l	7sa9	

References

(1) Son, J.; Jang, J.; Beyett, T. S.; Eum, Y.; Haikala, H. M.; Verano, A.; Lin, M.; Hatcher, J. M.; Kwiatkowski, N. P.; Eser, P. Ö.; et al. A Novel HER2-Selective Kinase Inhibitor Is Effective in HER2 Mutant and Amplified Non–Small Cell Lung Cancer. *Cancer Research* **2022**, *82* (8), 1633-1645. DOI: 10.1158/0008-5472.Can-21-2693.

(2) Sohrab, S. S.; Kamal, M. A. Screening, Docking, and Molecular Dynamics Study of Natural Compounds as an Anti-HER2 for the Management of Breast Cancer. *Life* **2022**, *12* (11), 1729. DOI: 10.3390/life12111729.

(3) Liu, S.; Sommese, R. F.; Nedoma, N. L.; Stevens, L. M.; Dutra, J. K.; Zhang, L.; Edmonds, D. J.; Wang, Y.; Garnsey, M.; Clasquin, M. F. Structural basis of lipid-droplet localization of 17-beta-hydroxysteroid dehydrogenase 13. *Nat. Commun.* **2023**, *14* (1), 5158. DOI: 10.1038/s41467-023-40766-0.

(4) Zhang, X.; Zhou, C.; Yang, Y.; Liu, H.; Wang, S.; Ding, X.; Wang, H. The Discovery of Potential MDM2 Inhibitors: A Combination of Pharmacophore Modeling, Virtual Screening, Molecular Docking Studies, and in vitro/in vivo Biological Evaluation. *ChemMedChem* **2022**, *17* (4), e202100517. DOI: 10.1002/cmdc.202100517 (acccessed 2025/03/18).

(5) Vassilev, L. T.; Vu, B. T.; Graves, B.; Carvajal, D.; Podlaski, F.; Filipovic, Z.; Kong, N.; Kammlott, U.; Lukacs, C.; Klein, C.; et al. In Vivo Activation of the p53 Pathway by Small-Molecule Antagonists of MDM2. *Science* **2004**, *303* (5659), 844-848. DOI: 10.1126/science.1092472 (acccessed 2025/03/18).

(6) Leal, A. S.; Hung, P.-Y.; Chowdhury, A. S.; Liby, K. T. Retinoid X Receptor agonists as selective modulators of the immune system for the treatment of cancer. *Pharmacol. Ther.* **2023**, *252*, 108561. DOI: 10.1016/j.pharmthera.2023.108561.

(7) Chen, L.; Wang, Z.-G.; Aleshin, Alexander E.; Chen, F.; Chen, J.; Jiang, F.; Alitongbieke, G.; Zeng, Z.; Ma, Y.; Huang, M.; et al. Sulindac-Derived RXRα Modulators Inhibit Cancer Cell Growth by Binding to a Novel Site. *Chem. Biol.* **2014**, *21* (5), 596-607. DOI: 10.1016/j.chembiol.2014.02.017.

(8) Shi, D.; Pang, Q.; Qin, Q.; Yao, X.; Yao, X.; Yu, Y. Discovery of novel anti-tumor compounds targeting PARP-1 with induction of autophagy through in silico and in vitro screening. *Front. Pharmacol.* **2022**, *13*, 1026306, Original Research. DOI: 10.3389/fphar.2022.1026306.

(9) Zandarashvili, L.; Langelier, M.-F.; Velagapudi, U. K.; Hancock, M. A.; Steffen, J. D.; Billur, R.; Hannan, Z. M.; Wicks, A. J.; Krastev, D. B.; Pettitt, S. J.; et al. Structural basis for allosteric PARP-1 retention on DNA breaks. *Science* **2020**, *368* (6486), eaax6367. DOI: 10.1126/science.aax6367 (acccessed 2025/03/18).

(10) Du, R.; Huang, C.; Liu, K.; Li, X.; Dong, Z. Targeting AURKA in Cancer: molecular mechanisms and opportunities for Cancer therapy. *Mol. Cancer* **2021**, *20* (1), 15. DOI: 10.1186/s12943-020-01305-3.

(11) Gilic, M. B.; Tyner, A. L. Targeting protein tyrosine kinase 6 in cancer. *Biochim. Biophys. Acta* **2020**, *1874* (2), 188432. DOI: https://doi.org/10.1016/j.bbcan.2020.188432.

(12) Ajmal, A.; Danial, M.; Zulfat, M.; Numan, M.; Zakir, S.; Hayat, C.; Alabbosh, K. F.; Zaki, M. E. A.; Ali, A.; Wei, D. In Silico Prediction of New Inhibitors for Kirsten Rat Sarcoma G12D Cancer Drug Target Using Machine Learning-Based Virtual Screening, Molecular Docking, and Molecular Dynamic Simulation Approaches. *Pharmaceuticals* **2024**, *17* (5), 551. DOI: 10.3390/ph17050551.
